# Coverage and error models of protein-protein interaction data by directed graph analysis

**DOI:** 10.1186/gb-2007-8-9-r186

**Published:** 2007-09-10

**Authors:** Tony Chiang, Denise Scholtens, Deepayan Sarkar, Robert Gentleman, Wolfgang Huber

**Affiliations:** 1 EMBL, European Bioinformatics Institute, Wellcome Trust Genome Campus, Hinxton, Cambridge, CB10 1SD, UK; 2 Fred Hutchinson Cancer Research Center, Computational Biology Group, Fairview Avenue North, Seattle, WA 98109-1024, USA; 3 Northwestern University, Department of Preventive Medicine, N Lake Shore Drive, Chicago, IL 60611-4402, USA

## Abstract

Using a directed graph model for bait to prey systems and a multinomial error model, we assessed the error statistics in all published large-scale datasets for *Saccharomyces cerevisiae *and characterized them by three traits: the set of tested interactions, artifacts that lead to false-positive or false-negative observations, and estimates of the stochastic error rates that affect the data. These traits provide a prerequisite for the estimation of the protein interactome and its modules.

## Abstract 2

Directed graph and multinomial error models were used to assess and characterize the error statistics in all published large-scale datasets for *Saccharomyces cerevisiae*

## Background

Within the past decade a large amount of data on protein-protein interactions in cellular systems has been obtained by the high-throughput scaling of technologies, such as the yeast two-hybrid (Y2H) system and affinity purification-mass spectrometry (AP-MS) [1-15]. This opens the possibility for molecular and computational biologists to obtain a comprehensive understanding of cellular systems and their modules [16]. There are many references in the literature, however, to the apparent noisiness and low quality of high-throughput protein interaction data. Evaluation studies have reported discrepancies between the datasets, large error rates, lack of overlap, and contradictions between experiments [17-30]. The interpretation and integration of these large sets of protein interaction data represents a grand challenge for computational biology.

In essence, inference on the existence of an interaction between two proteins is made based on the measured data, and such inference can either be right or wrong. Most publicly available data are stored as positive measured results, and therefore most analyses have employed the most obvious method to infer interactions; a positive observation indicates an interaction, whereas a negative observation or no observation does not. This method, although useful and sometimes unavoidable, does not make use of other indicators for the presence or absence of interactions.

The most useful and yet seldom used indicator is the information about which set of interactions were tested. As mentioned, most studies report positively measured interactions but few report the negative measurements. It is quite often the case that untested protein pairs and negative measurements are not distinguished. A second indicator of the presence of an interaction is reciprocity. Bait to prey systems allow for the testing of an interaction between a pair of proteins in two directions. If bi-directionally tested, we anticipate the result as both positive or both negative. Failure to attain reciprocity indicates some form of error. A third indicator is the type of interaction being assayed; direct physical interactions must be differentiated from indirect interactions, and this difference plays an important role in inference. In the Y2H system, two proteins are modified so that a physical interaction between the two can reconstitute a functioning transcription factor. In AP-MS, a single protein is chosen and modified, and each pull-down detects proteins that are in some complex with the selected one but may not necessarily directly interact with the chosen protein.

Restricting our attention to bi-directionally tested interactions, we can use a binomial model to identify proteins that either find a disproportionate number of prey relative to the number of baits that find them or *vice versa*. For the AP-MS experiments, there is an association between whether a protein exhibits this discrepancy and its relative abundance in the cell. For the Y2H system, analyses conducted separately by Walhout and coworkers [31], Mrowka and colleagues [19], and Aloy and Russell [32] have reported on this type of artifact and have discussed a relationship between it and some bait proteins' propensity to act alone as activators of the reporter gene. Our methods provide a simple test to identify proteins that are probably affected by such systematic errors. Such diagnostics can aid in the interpretation of the data and in the design of future experiments. By restricting attention to proteins that are not seen to be affected by this artifact, we can refine the error modeling and the subsequent biologic analysis.

## Results and discussion

### Tested interactions and their representations

In the Y2H system, the bait is the protein tagged with the DNA binding domain, and the prey is the hybrid with the activation domain. Only those constructs that result in a functional fusion protein will be tested as bait or as a prey. In AP-MS, a piece of DNA encoding a tag is inserted into a protein-coding gene, so that yeast cells express the tagged protein. These are the baits. The prey are unmodified proteins expressed under the conditions of the experiment. The set of tested baits, even in experiments intended to be genome wide, can be quite restricted. For example, Gavin and coworkers [10] designed their experiment to employ the 6,466 open reading frames that were at that time annotated with the *Saccharomyces cerevisiae *genome, but successfully obtained tandem affinity purifications for 1,993 of those. The remaining 4,473 (69%) failed at various stages, because, for example, the tagged protein failed to express or the bands resulting from the gel electrophoresis were not well separated.

It is difficult to give an accurate enumeration of the sets of tested baits and tested prey in an experiment, and often the published data do not contain sufficient detail to allow identification of these sets. As a proxy, we introduce the concepts of viable baits and viable prey; the first is the set of baits that were reported to have interacted with at least one prey, and the latter is similarly defined. These quantities are unambiguously obtained from the reported data and provide reasonable surrogate estimates for what are the tested baits and tested prey. The set of ordered pairs, one being a viable bait and the other a viable prey, are interactions for which we have a level of confidence that were experimentally tested and could, in principle, have been detected. The failure to detect an interaction between a viable bait and a viable prey is informative, whereas the absence of an observed interaction between an untested bait and prey is not. This approach over-emphasizes positive interactions; potentially, valid data on tested proteins that have truly no interactions with any other tested protein will be discarded.

Protein interactions have been generally modeled by ordinary graphs [33]. The proteins correspond to the nodes of the graph, and edges between protein pairs indicate an interaction (either physical interaction or complex co-membership). For measured data from bait to prey systems, protein pairs are ordered (*b*,*p*) to distinguish a bait *b *from a prey *p*. There are three types of relationships between protein pairs of an experimental dataset: tested with an observed interaction, tested with no observed interaction, and untested. An adequate representation for this type of datum would be a directed graph with edge attributes. A directed edge (*b*,*p*)_+ _signals testing with an observed interaction, whereas a directed edge (*b*,*p*)_- _signals testing without an observed interaction. Interactions between proteins that are not adjacent were not tested. In those cases in which all protein pairs were reciprocally tested, we can suppress the (*b*,*p*)_- _edges, and a directed graph (digraph) is an adequate representation.

**Figure 1:**
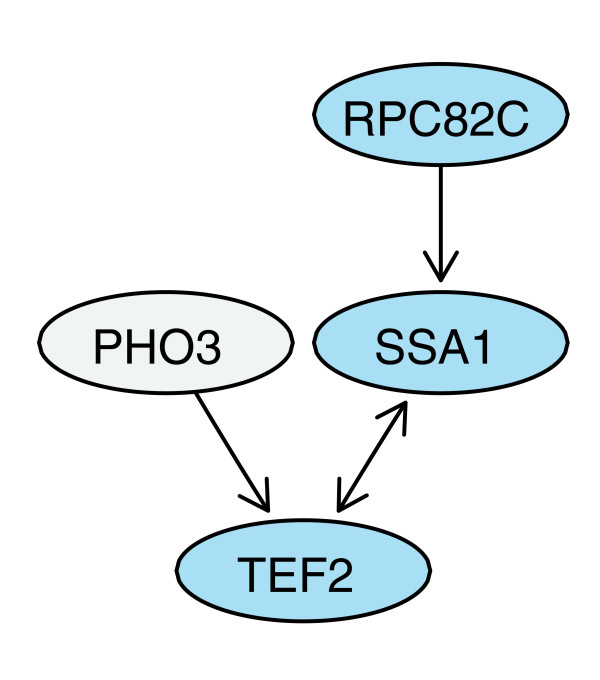
Measured protein interaction data are represented by a directed graph. The graph shows the interaction data between four selected proteins from the report by Krogan and coworkers [11]. The bi-directional edge between the ATPase SSA1 and the translational elongation factor TEF2 indicates that either one as a bait pulled down the other one as a prey. The directed edge from RPC82, a subunit of RNA polymerase III, to SSA1 indicates that RPC82 as a bait pulled down SSA1, but not *vice versa*. Another unreciprocated edge goes from the phosphatase PHO3 to TEF2. An investigation of the dataset shows that PHO3, which localizes in the periplasmatic space, was not reported in any interaction as a prey, whereas RPC82C was. In the interpretation of the data, we would have most confidence that there is a real interaction between SSA1 and TEF2. We can differentiate between the two unreciprocated interactions; the one between RPC82C and SSA1 has been bi-directionally tested, but only found once, whereas the other one has only been uni-directionally tested and found.

As mentioned above, information on which protein pairs were tested for an interaction is rarely explicitly reported, and so we represent the current data by a directed graph with node attributes. Using viability as a proxy for testing, the nodes with non-zero out-degree are presumed to be the set of viable baits, and similarly the nodes with non-zero in-degree are presumed to be the viable prey. Isolated nodes become identified as the set of untested proteins (both as bait and prey). We make use of such a di-graph data structure in this report (Figure 1).

### Interactome coverage

**Figure 2:**
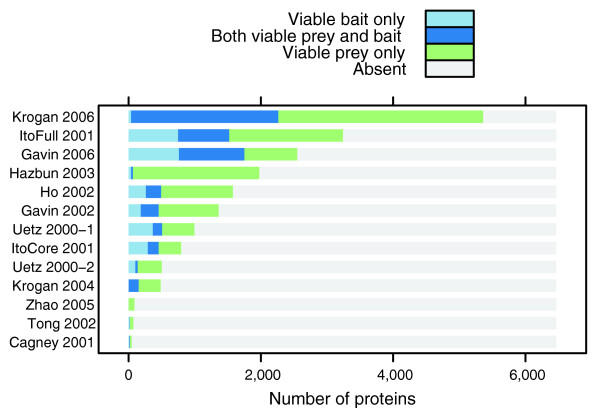
Proportions of proteins sampled across datasets. This bar chart shows the proportion of proteins sampled either as a viable bait (VB), a viable prey (VP), or as both (VBP). With the exception of the data report by Krogan and coworkers [11], the other 11 datasets show large portions of the yeast genome that did not participate in any positive observations. Without additional information, there is little we can do to elucidate whether these proteins were tested but inactive for all tests, or whether these proteins were not tested.

Given the experimental data, one can partition the proteins into four different sets: viable bait only (VB), viable prey only (VP), viable bait/prey (VBP), and the untested proteins. Figure 2 shows these proportions of the yeast genome as measured by each experiment. For most experiments, relatively large portions of the proteome were untested by the assay (gray area), thereby rendering an incomplete picture of the overall interactome [18,21,25,34].

We considered whether the sets of viable bait and viable prey exhibited a coverage bias in the experimental assays. Applying a conditional hypergeometric test [35] to the terms within the cellular component branch of Gene Ontology (GO), we found that proteins annotated to categories such as nucleus (primarily Y2H), cytoplasm, and protein complex were over-represented among the viable protein population relative to the yeast genome. This is not surprising because both Y2H and AP-MS assay two kinds of interactions in protein complexes. The Y2H technology is more successful in generating viable proteins within the nucleus because this is the cellular location where the test is performed, and so native proteins tend to work more successfully.

The conditional hypergeometric tests can also identify portions of the cellular component missed by either Y2H or AP-MS. For the Y2H technology, terms associated with mitochondrion, ribosome, and integral to membrane were under-represented by viable proteins. Like the Y2H systems, the viable proteins from AP-MS assays were also under-represented with respect to terms associated with mitochondrion and integral to membrane, but instead of ribosome AP-MS showed under-representation in vacuole. These under-represented categories are limited by the technologies because all datasets were derived before progress had been made to probe membrane-bound proteins.

Every dataset, whether Y2H or AP-MS, exhibited under-representation for the term cellular component unknown. One possible explanation for this phenomenon can be attributed to the correlation between different technologies. It seems that proteins that are problematic in the Y2H and AP-MS systems might also be problematic in systems to determine their cellular localization. Ultimately, further experiments are needed to determine why certain GO categories are under-represented. The hypergeometric analysis on each dataset can be found in the Additional data files.

These findings point to the fact that the subset of the interactome is either non-randomly sampled or non-randomly covered by the experiment. Either effect limits the type of inference that can be conducted on the resulting data. For instance, inference on statistics such as the degree distribution or the clustering coefficient of the overall graph is less meaningful as long as the direction and magnitude of the coverage or sampling biases are not well understood [20,36,37].

### Systematic bias: per protein and experiment wide

The interactions between VBP proteins were tested in both directions, and a surprising yet useful observation is that there is a large number of unreciprocated edges in the data [32]. These unreciprocated interactions can be used to understand better the experimental errors.

**Figure 3:**
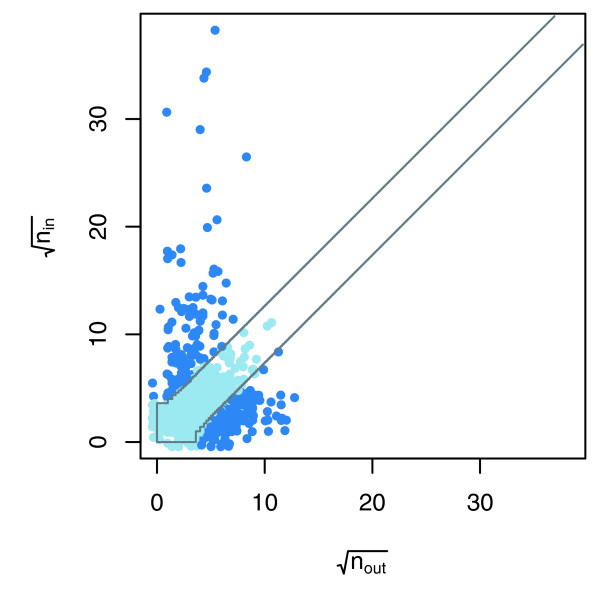
Two-sided binomial test on the data from Krogan and coworkers [11]. The scatter-plot shows (*o*_*p*_,*i*_*p*_) for each *p *∈ VBP from the report by Krogan and coworkers [11] (axes are scaled by the square root). The proteins that fall outside of the diagonal band exhibit high asymmetry in unreciprocated degree. This figure shows a graphical representation of a two-sided binomial test. The points above and below the diagonal band are proteins for which we reject the null hypothesis that the distribution of unreciprocated edges is governed by B(*n*_*p*_,$\frac{1}{2}$). For the purpose of visualization, small random offsets were added to the discrete coordinates of the data points by the R function jitter. VBP, viable bait/prey.

Each VBP protein *p *has *n*_*p *_unreciprocated edges, and under the assumption of randomness we expect the number of unreciprocated in-edges and out-edges to be similar. More precisely, under the assumption that the direction of the edge is random, the number of unreciprocated in-edges is distributed as the number of heads obtained by tossing a fair coin *n*_*p *_times. Based on this coin tossing model, we used a per protein binomial error model (see Materials and methods, below) to test the statistical significance for the number of unreciprocated in-edges (heads) against the number unreciprocated out-edges (tails). Figure 3 shows a partition of the VBP proteins from the data of Krogan and coworkers [11] based on the two-sided statistical test derived from the binomial model with a *P *value threshold of 0.01. Those proteins falling outside the diagonal band are considered to be affected by a systematic bias.

It is interesting to note that the proportion of VBP proteins identified by the binomial error model as potentially affected by bias is quite small for the Y2H experiments and the smaller scale AP-MS experiments (<3%), whereas the two larger scale AP-MS experiments showed relatively greater proportions (>14%). It is equally important to note that although these proportions still constitute a minority of VBP proteins, these proteins (within the large-scale AP-MS experiments) participate in a relatively large number of observed interactions, most of which are unreciprocated.

Having identified sets of proteins that are likely to have been affected by this systematic bias, we considered whether these proteins could be associated with biologic properties. To this end, we fit logistic regression models (Additional data files) to predict this effect, and in the AP-MS system we found evidence that the codon adaptation index (CAI) and protein abundance are associated with the highly unreciprocated in-degree of VBP proteins (proteins that were found by an exceptionally high number of baits relative to the number of prey they found themselves when tested as baits). The CAI is a per-gene score that is computed from the frequency of the usage of synonymous codons in a gene's sequence, and can serve as a proxy for protein abundance [38].

**Figure 4:**
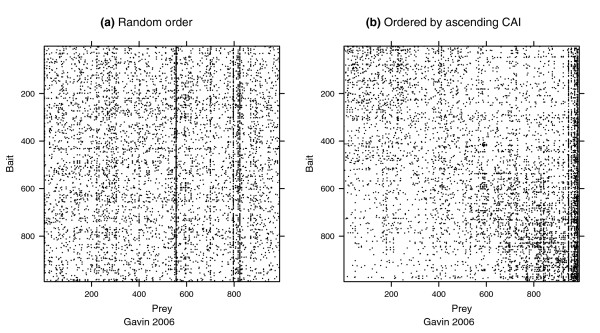
Adjacency matrices: random versus ascending CAI. These plots present a view of the adjacency matrix for the viable bait/prey (VBP) derived from the report from Gavin and coworkers [10]. An interaction between bait *b *and prey *p *is recorded by a dark pixel in (b,p)th position of the matrix. **(a)** Rows and columns are randomly ordered; **(b)** rows and columns are ordered by ascending values of each protein's codon adaptation index (CAI). Contrasting these two figures, we can ascertain that there is a relationship between bait/prey interactions and CAI. The relationship is based on proteins with large un-reciprocated in-degree because panel b shows a dark vertical band. Had unreciprocated out-degree also been associated with CAI, then there would be a similar horizontal band reflected across the main diagonal of the matrix.

To visualize the association between such proteins and CAI, we plotted diagrams of the adjacency matrix. If the value of CAI is associated with the tendency of a protein to have a large number of unreciprocated edges, then we should see a pattern in the adjacency matrix when the rows and columns are ordered by ascending CAI values. We do this for the data reported by Gavin and coworkers [10] in Figure 4. We see a dark vertical band in Figure 4b representing a relatively high volume of prey activity. There is no corresponding horizontal band in Figure 4a, which suggests that the relationship of CAI to the AP-MS system is primarily reflected in a protein's in-degree.

**Figure 5:**
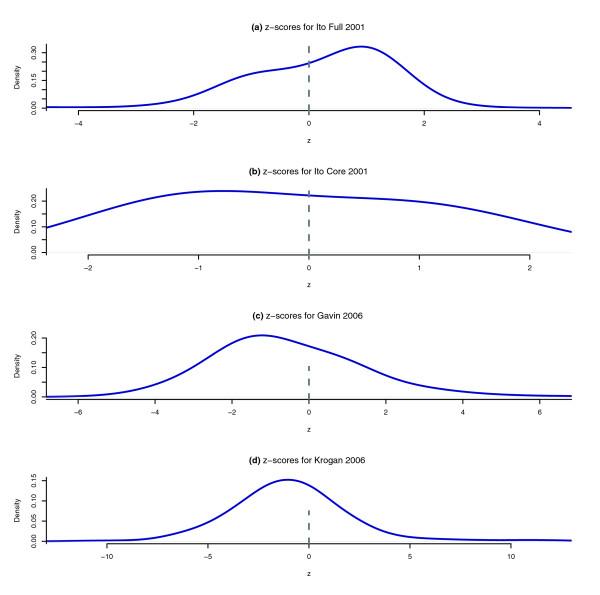
Density plots of the in-degree *z*-scores. The plots show the density estimates of the in-degree *z*-scores for [1,10,11]. The zero line is present to distinguish between positive and negative *z*-scores. The distribution reported by Ito and coworkers [1] shows a high concentration of data points that have positive *z*-scores, whereas the data reported by Gavin and coworkers [10] and Krogan and colleagues [11] have maximal density for negative *z*. Systematic artifacts such as auto-activators in the yeast two-hybrid (Y2H) system and protein abundance in affinity purification-mass spectrometry (AP-MS) might play a role in off-zero mean of these density plots. Restricting to the Ito-Core set appears to eliminate the effect from the Ito-Full set.

Next, we standardized the in-degree for each protein by calculating its *z*-score (see Materials and methods, below) and then plotted the distributions of these *z*-scores by their density estimates. Four experiments appeared to exhibit particularly distinct distributions (Ito-Full, Ito-Core, Gavin *et al*. 2006, and Krogan *et al*. 2006; Figure 5) [1,10,11]. The Ito-Full [1] dataset shows the largest mean (approximately two to four times the mean of the other Y2H distributions). This is consistent with reports that there were many auto-activating baits in the Ito-Full datasets [32]; if a relatively small number of baits auto-activate, resulting in the cell's expression of the reporter gene, then this artificially increases the number of in-edges for a large number of prey proteins. Auto-activation would cause a shift in the *z*-score distribution in the positive direction. This effect is not seen in the Ito-Core data.

Although Ito and coworkers [1] tried to eliminate systematic errors by generating the Ito-Core subset of interactions, it is noteworthy to recall that they only used reproducibility as a criterion for validation without considering reciprocity. Consequently, almost half of the reciprocated interactions were not recorded in the Ito-Core set. Although reproducibility is a necessary condition for validation, it is insufficient because systematic errors are often reproducible.

Among the AP-MS datasets, the data reported by both Gavin and coworkers [10] and Krogan and colleagues [11] display negative means. A possible interpretation of this effect can be attributed to the abundance of the prey under the conditions of the experimental assay. The AP-MS system is more sensitive in detecting the complex co-members of a particular bait than in the reverse. For instance, if a lowly expressed protein *p *is tagged and expressed as a bait and pulls-down proteins *p*_1_,...,*p*_*k *_as prey, then the reverse tagging of each protein of *p*_1_,...,*p*_*k *_will have a smaller probability of finding *p*. Even if the lowly abundant protein *p *is pulled down in the reverse tagging, the mass spectrometry may fail to detect *p *within the complex mixture [39,40]. Both of these observations could explain why we observed proteins having an overall slightly higher out-degree than in-degree, and therefore an overall slightly negative mean for the *z*-score distribution.

Finally, we wished to cross-compare the systematic errors between experiments. Only two experiments had sufficient size to give reasonable statistical power. Thus, to compare systematic errors of Gavin and coworkers [10] against those of Krogan and colleagues [11], we generated two-way tables (Tables 1 to 4; also, see Materials and methods, below). Although the concordance is not complete, there is evidence that overlapping sets of proteins are affected. This indicates that both experiment specific and more general factors could be at work, resulting in these unreciprocated edges.

**Table 1:** Across experiment comparison of protein subsets associated with systematic error

	Not in Krogan *et al*. [11]	In Krogan *et al*. [11]
Not in Gavin *et al*. [10]	624	63
In Gavin *et al*. [10]	31	12
		
	*P *= 6.5 × 10^-4^	Odds ratio = 3.82

This table compares the proteins affected by a reciprocity artifact from the datasets of Gavin and coworkers [10] and Krogan and colleagues [11]. Binomial tests were applied to identify the affected protein sets within each experiment, and their overlap was assessed in the 2 × 2 contingency table. In this table, the binomial tests were applied to the two experimental datasets independently, and only those proteins in which the in-degree is much larger than the out-degree are considered. Shown *P *value and odds ratio were calculated from the 2 × 2 table using the hypergeometric distribution.

**Table 2:** Across experiment comparison of protein subsets associated with systematic error

	Not in Krogan *et al*. [11]	In Krogan *et al*. [11]
Not in Gavin *et al*. [10]	480	181
In Gavin *et al*. [10]	40	29
		
	*P *= 1.6 × 10^-2^	Odds ratio = 1.92

Like Table 1, this table also compares the proteins affected by a reciprocity artifact from the datasets of Gavin and coworkers [10] and Krogan and colleagues [11]. The only exception is that the proteins compared were those identified by the binomial tests as having out-degree greater than in-degree. Compared with Table 1, the association between the two datasets is relatively weaker in terms of both the *P *value and odds-ratio.

**Table 3:** Across experiment comparison of protein subsets associated with systematic error

	Not in Krogan *et al*. [11]	In Krogan *et al*. [11]
Not in Gavin *et al*. [10]	651	45
In Gavin *et al*. [10]	26	8
		
	P = 1.8 × 10^-3^	Odds ratio = 4.44

This table represents the comparison of proteins affected by a reciprocity artifact from the datasets of Gavin and coworkers [10] and Krogan and colleagues [11] as well. Before conducting the binomial test, the data graphs were restricted to the nodes common to the viable bait/prey (VBP) sets of both experiments. Again, only those proteins identified by the binomial test in which in-degree is much larger than the out-degree is compared. Both the *P *value and odds ratio, obtained using the hypergeometric distribution, show a strong association between the two sets of proteins.

**Table 4:** Across experiment comparison of protein subsets associated with systematic error

	Not in Krogan *et al*. [11]	In Krogan *et al*. [11]
Not in Gavin *et al*. [10]	602	78
In Gavin *et al*. [10]	39	11
		
	*P *= 4.1 × 10^-2^	Odds ratio = 2.17

Like Table 3, this table also compares the proteins affected by a reciprocity artifact from the datasets of Gavin and coworkers [10] and Krogan and colleagues [11] restricted to the common viable bait/prey (VBP) proteins. We consider those proteins identified by the binomial test in which the out-degree is much larger than the in-degree. We again see that the association between the proteins sets in terms of *P *value and odds ratio is weaker when compared with the association obtained from Table 3.

### Stochastic error rate analysis

There has been confusion in the literature when analyzing error statistics, because different articles have used different definitions for the same statistic. Proteins pairs can either interact or not, and so the pairs themselves can be partitioned into two distinct sets; the set of interacting pairs, *I*, and the set of non-interacting pairs, *I*^*C*^. False negative (FN) interactions and true positive (TP) interactions can only occur within the set *I*, and therefore the false negative probability (*P*_FN_) and the true positive probability (*P*_TP_) are properties on *I*. Similarly, the false positive (*P*_FP_) and true negative (*P*_TN_) probabilities are properties on *I*^*C *^[41]. These standard definitions, along with the values *n *= |*I*| and *m *= |*I*^*C*^|, allow us to set up equations for the expectation values of three random variables: the number of reciprocated edges (*X*_1_), the number of protein pairs between which no edge exists (*X*_2_), and the number of unreciprocated edges (*X*_3_).

*E*[*X*_1_] = *n *(1 - *P*_FN_)^2 ^+ *mP*_FP_^2^

*E*[*X*_2_] = *nP*_FN_^2 ^+ *m*(1 - *P*_FP_)^2^

*E*[*X*_3_] = 2*nP*_FN_(1 - *P*_FN_) + 2*mP*_FP_(1 - *P*_FP_)

**Figure 6:**
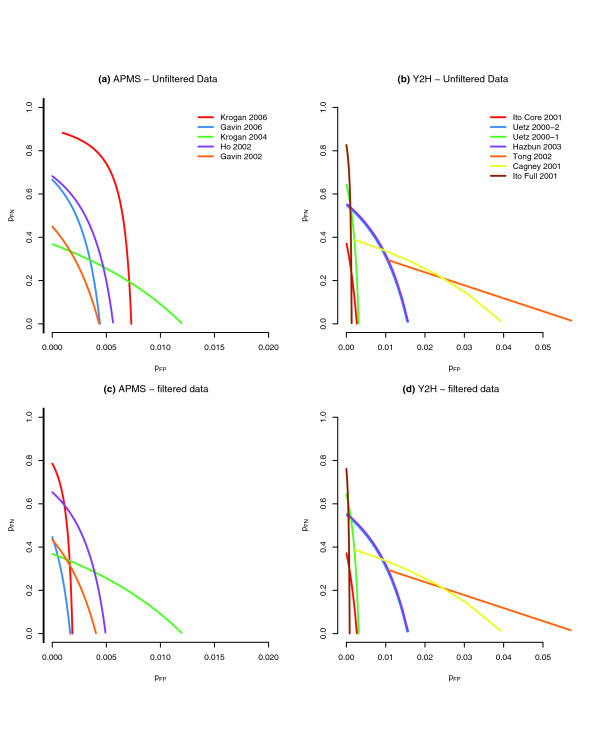
Geometric visualization of the solution curves from the algebraic equations 1 to 3. **(a)** Plot of (*P*_FP_,*P*_FN_) parameterized by *n *for the affinity purification-mass spectrometry (AP-MS) datasets. **(b)** Curves for the yeast two-hybrid (Y2H) datasets. **(c)** AP-MS data filtered for the proteins that were rejected by the binomial test for systematic bias. **(d)** curves for the Y2H data with the application of the analogous filters. These curves give upper bounds for the values of (*P*_FP_,*P*_FN_) in the multinomial error model for each experiment. Each point on any of the curves represents three distinct values based on the methods of moments restricted to the viable bait/prey (VBP) proteins: the true number of interactions between the VBP proteins, the *P*_FP _rate, and the *P*_FN _rate. If one of these three parameters can be estimated, then the other two will also be determined.

We recall that if *N *is the number of proteins, then *n *+ *m *= $\left(\begin{array}{c} N\\ 2 \end{array}\right)$, which is the number of all pairs of proteins. Any two of these three equations imply the third, and therefore there are three unknowns and two independent equations. By the method of moments[42], we replace the left hand side of Equations1 to 3 with the observed values for the number of reciprocated interactions (*x*_1_), for the number of reciprocally non-interacting protein pairs (*x*_2_), and for the number of unreciprocated interactions (*x*_3_); it follows that knowledge of any one of (*P*_FP_,*P*_FN_,*n*) yields the other two through an application of the quadratic formula (see Materials and methods, below). Otherwise, if none of these three parameters is known from other sources, then Equations1 to 3 define a family of solutions (a one-dimensional set of solutions in a space of three variables; Figure 6).

The variability, or stochastic error, that affects a bait to prey system can thus be characterized by a one-dimensional curve in a three-dimensional space, {(*P*_FP_,*P*_FN_,*n*)}, which depends on the experiment and can be estimated from the three experiment-specific numbers *x*_1_, *x*_2_, and *x*_3_. If we can identify portions of the data that appear to be affected by systematic bias, such as that described in the preceding section, then we can set these aside and focus the characterization of the experimental errors on the remaining filtered set of data, typically with lower estimates for *P*_FP _and *P*_FN_.

To gain insight into the prevalence of FP and FN stochastic errors, we calculated estimates of the expected number of FP and FN observations using Equations 1 to 3, and present the results in Tables 5 and 6. Table 5 considers the worst-case scenario for FP errors, setting *P*_FN _= 0, and hence assuming that all errors are false positives. We discuss the first row, corresponding to the data of Ito-Full [1], as an example. A total of 720 proteins were not rejected in the two-sided binomial test, and there are $\left(\begin{array}{c} 720\\ 2 \end{array}\right)$ = 258,840 protein pairs, excluding homomers. This gives us an upper limit for *m*. From the solution manifold shown in Figure 6d, we see that an estimate for *P*_FP _is approximately 0.0008. From this it follows that the expected number of unreciprocated FP interactions is 414 and of reciprocated FP interactions is 0.17. The actual data contain 435 unreciprocated interactions and 68 reciprocated ones. So, even in the estimated worst case, when all errors are FP observations, reciprocated observations are still most likely due to true interactions.

**Table 5:** Estimates for the FP errors of each filtered dataset

Dataset (ref.)	*N*	*m*	*P*_FP_	*E *[*Y*_1_]	*E *[*Y*_2_]	*U*_*obs*_	*R*_*obs*_
ItoFull [1]	720	258,840	0.0008	414	0.17	435	68
ItoCore [1]	128	8,128	0.0025	41	0.05	43	36
Uetz *et al*. [6]	108	5,778	0.003	35	0.05	36	10
Gavin *et al*. [10]	852	362,526	0.0017	1230	1.10	1201	743
Krogan *et al*. [11]	1,458	1,062,153	0.0019	4,029	3.80	3945	538

Shown are the expected number of false positive (FP) errors on the filtered datasets for [1,6,10,11]. *N *is the number of proteins within each filtered dataset. The values for P_FP _and *m *are estimated upper bounds obtained by setting *P*_FN _= 0 and using the solution curves of Figure 6c,d. Denote *Y*_1 _as the random variable for the number of unreciprocated FP observations, and *Y*_2 _for the number of reciprocated FP observations. The variables *U*_*obs *_and *R*_*obs *_show the observed number of unreciprocated and reciprocated interactions from the data, respectively. The table implies that even in the worst case scenario for maximal *P*_FP_, reciprocated edges mostly report true interactions.

**Table 6:** Estimates for the FN errors of each filtered dataset

Dataset (ref.)	*N*	*n*	*P*_FN_	*E *[*W*_1_]	*E *[*W*_2_]	*U*_*obs*_	*R*_*obs*_
ItoFull [1]	720	1,200	0.76	438	693	435	259,132
ItoCore [1]	128	100	0.38	47	14	43	8,156
Uetz *et al*. [6]	108	78	0.65	35	33	36	5,822
Gavin *et al*. [10]	852	2,429	0.44	1197	470	1,201	362,209
Krogan *et al*. [11]	1,458	11,744	0.80	3758	7,516	3,945	1,062,344

The expected number of false-negative (FN) errors on the filtered datasets for [1,6,10,11]. *N *is the number of proteins within each filtered dataset. The values for *P*_FN _and *n *are estimated upper bounds obtained by setting *P*_FP _= 0 and using the solution curves of Figure 6c,d. Denote *W*_1 _as the random variable for the number of unreciprocated FN observations, and *W*_2 _for the number of reciprocated FN observations. The variables *U*_*obs *_and *R*_*obs *_show the observed number of unreciprocated and reciprocated interactions from the data, respectively. The table implies that in the worst case scenario for *P*_FN_, the doubly tested, reciprocated noninteracting protein pairs do not give us a conclusive indication about the presence or absence of an interaction. For this, more data are needed.

It is important to contrast the nature of the stochastic error rates because there is confusion in the literature concerning these statistics. From Figure 6, the solution curve gives an estimate for the *P*_FP _rate at 0.0008 conditioned on the Ito-Full VBP data and conditioned on *P*_FN _= 0; a similar estimate for the Ito-Core dataset yields *P*_FP _at 0.0025. The reason for this is because the number of non-interacting protein pairs in the former is estimated to be approximately 250,000, whereas this number is 8,000 for the latter. Table 5 shows that the number of expected false positively identified unreciprocated interactions for Ito-Full is 414 and for the Ito-Core is 41. Thus, although the *P*_FP _rate of Ito-Full is three times smaller than that of Ito-Core, the expected number of falsely discovered interactions is an order of magnitude greater. Therefore, a generic interaction contained within Ito-Core is much more likely to be true than one from Ito-Full. Comparing the *P*_FP _rate from Ito-Full with the *P*_FP _rate from Ito-Core is unreasonable when the underlying sets of non-interacting proteins pairs are entirely different. The false discovery rate is more intuitive, and this statistic has often been confused in the literature with the FP rate.

We also considered the worst-case scenario for FN errors. By setting *P*_FP _= 0, we calculated the expected number of unreciprocated and reciprocated false negatives in the absence of FP errors. These numbers are presented in Table 6. Because of the size of *P*_FN_, we find that a large number of protein pairs between which no edge was reported in either direction may still, in truth, interact.

Ultimately, an observed unreciprocated interaction in the data indicates that either a FP or a FN observation was made. Computational models cannot definitively conclude which of these two occurred, but these models indicate the magnitude and nature of the problem and can be used to compare experiments, because those with relatively higher error rates should be discounted in any downstream analyses.

## Conclusion

We have shown that protein interaction datasets can be characterized by three traits: the coverage of the tested interactions, the presence of biases in the assay that systematically affect certain subsets of proteins, and stochastic variability in the measured interactions. In turn, these three characteristics can benefit the design of future protein interaction experiments.

The set of interactions tested is important because datasets usually report positive results, but tend to be ambiguous on the significance of the unreported interactions. Is it because the interaction was tested and not detected, or because it was not tested in the first place? Distinguishing the two cases is important for inference and for integration across datasets. For the currently available datasets from Y2H and AP-MS, a practical estimate of what is the set of tested interactions is all pairs of tested bait and tested prey. A comprehensive list of tested proteins is usually not reported. We can, however, obtain a useful approximation for the tested baits and prey using the notion of viability. However, this assumption does introduce some bias, especially for experiments with relatively few bait proteins, because proteins that were tested but did not interact with any bait protein will not be counted, falsely raising the proportion of interactions. On the other hand, when complete data are not reported the presumption that interactions were tested, when they were not, introduces bias in the other direction.

**Figure 7:**
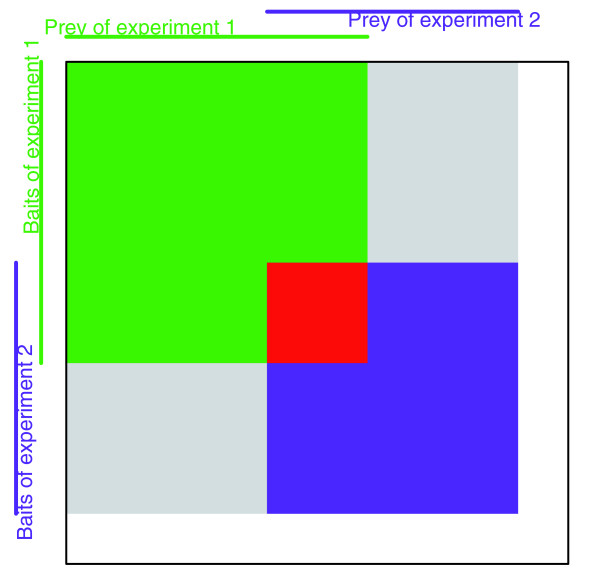
Matrix representation on two separate bait to prey datasets. A schematic representation of the interactome coverage of two protein interaction experiments. The adjacency matrix of the complete interactome is represented by the large square. Experiment 1 covers a certain set of proteins as baits (rows covered by the green vertical line) and as prey (columns covered by the green horizontal line). The tested interactions for experiment 1 are contained within the green rectangle. Similarly, experiment 2 covers another set of proteins and tests for a set of interactions contained in the purple rectangle. In the intersection of the rectangles, the red area, are the bait to prey interactions tested by both experiments, and in the union are the interactions tested by at least one of the experiments. Note that the interactions in the light gray area were tested by neither experiment, either because there are missing tested prey (upper right corner) or missing tested baits (lower left corner). The interactions in the white region are also tested by neither experiment because both the baits and the prey were not tested.

There has been substantial interest in cross-experiment analysis, or in integrating data from multiple sources [19,23,24,29,30]. The possible pitfalls of naïve comparisons between two experimental datasets are depicted in Figure 7. The interactions in the intersection of the rectangles (red) were tested by both; the interactions in the green and purple areas were tested by one experiment but not the other; and the interactions in the light gray areas were tested by neither experiment. Any data analysis that does not keep track of these different coverage characteristics risks being misled. Therefore, coverage must be taken into consideration when integrating and comparing multiple datasets. Additionally, systematic bias due to the experimental assay affects the detection of certain interactions between protein pairs, and these systematic errors should be isolated from the dataset before the estimating the stochastic errors. Ultimately, many more steps are still needed to integrate datasets, and we discuss a few necessary components.

If the assay system were perfect, then all bi-directionally tested protein pairs would either be reciprocally adjacent or not. In practice, unreciprocated edges are observed, and they can be used to understand better the sources of error. Measurement error can be divided into two categories: systematic and stochastic. We have shown that there are proteins with an inordinate imbalance between unreciprocated in-edges and out-edges, and they behave in a systematically different way when used as a bait than when found as a prey. This is an indication that the interaction data involving these proteins contain either a large number of false positives or of false negatives. Further data are needed to differentiate between these two alternatives. The mode in which they fail is distinct from the unspecific stochastic errors that we model via the FP and FN rates, and hence they should be excluded from these analyses.

It is useful to distinguish between the concepts of stochastic and systematic measurement error. Systematic errors are due to imperfections or biases in the experimental system, and they occur in a correlated or reproducible manner. Stochastic errors occur at random in an irreproducible manner; in principle, they can be averaged out by repeating the experiment often enough. There are many benefits to an analysis that identifies and separates these two types of measurement error. We have identified one type of systematic error in bait to prey systems that appears to be associated with artifacts of the technologies.

The occurrence of unreciprocated edges also points to some of the aspects of the technologies that could be improved. In AP-MS experiments, this artifact shows a strong association to CAI and protein abundance. Because mass spectrometry techniques are known to be, at times, less sensitive in identifying proteins with low abundance in a complex mixture, refinements of such methodology could potentially yield more accurate measurements.

The methods we have described are useful for future application of Y2H or AP-MS. Newer experiments can, and should, take into consideration relative protein abundance when assaying protein interactions. Besides this, the GO category analysis for under-representation shows certain proteins and protein complexes that do not work as intended under the conditions of the assay system. Knowing which categories are under-represented allows experimenters to adjust the technologies or create new technologies (such as the Y2H test for membrane bound proteins [43]).

These elementary questions of data pre-processing, quality assessment, and error modeling may appear far removed from the systems-level modeling of biologic systems. Such a modeling, however, requires the use and integration of multiple different datasets, to increase the breadth and depth of the data compared with those from a single experiment. This can only be done if the error statistics and possible patterns in the errors are sufficiently understood. We believe that the methods and tools developed in this work provide a step in this direction.

## Materials and methods

### Graph theory

We use a directed graph with node attributes to represent each measured dataset. The proteins correspond to the node set, and directed edges correspond with ordered protein pairs of the form (*b*,*p*) showing that a bait *b *detects a prey *p*. The node set with non-zero out-degree corresponds with the set of viable baits, and the node set with non-zero in-degree corresponds with the viable prey. We remove self-loops because we set aside homomer relationships. The subgraph generated by nodes that are both viable baits and viable prey will have tested all protein pairs bi-directionally.

### Protein interaction data

We investigated 12 publicly available datasets for *S. cerevisiae*, of which seven were assayed by Y2H and five were assayed by AP-MS. We obtained [1-6,10] from the IntAct repository [44] and [7-9,11] from their primary sources. All datasets have two key properties: information on the bait to prey directionality is retained; and the prey population is documented as genome wide. A table with an overview of the datasets can be found in the Additional data files.

### Statistical analysis

#### Binomial error model: detecting bias

The binomial error model assumes that in-degrees and out-degrees are equally likely among unreciprocated edges of a bi-directionally tested protein. Thus, we presume that the number of unreciprocated out-edges for any bi-directionally tested protein *p *is distributed as B(*n*_*p*_,$\frac{1}{2}$), where *n*_*p *_is the total number of unreciprocated edges of *p*. Under this hypothesis, we can compute the *P *value for the observed measured directed degree for each protein *p*. The null hypothesis is rejected at the 0.01 threshold. Proteins for which we reject the null hypothesis are deemed likely to be affected by a systematic bias in the assay.

#### Multinomial error model

Let *N *be the number of proteins in an interactome of interest, then the total number of distinct protein pairs, excluding homomers, is $\left(\begin{array}{c} N\\ 2 \end{array}\right)$. Denote the set of all unique interacting protein pairs among the *N *proteins by *I *and its complement by *I*^*C*^. Recall that $\left(\begin{array}{c} N\\ 2 \end{array}\right)$ = *n *+ *m*, where *n *= |*I*| and *m *= |*I*^*C*^|.

Only two of the three Equations 1 to 3 are independent, any two of them imply the third. We parameterize the one-dimensional solution manifold by *n *(0 ≤ *n *≤ $\left(\begin{array}{c} N\\ 2 \end{array}\right)$). Relevant solutions are those for which 0 ≤ *P*_FP_,*P*_FN _≤ 1. Consider *n *given, then we can solve for *P*_FN _in terms of *P*_FP_:

$$P_{FN}=\frac{1}{2n}(\Delta +2mP_{FP})$$

Where we have defined Δ = (*x*_2 _- *m*) - (*x*_1 _- *n*). Here, *x*_1 _is the observed number of reciprocated interactions and *x*_2 _is the number of reciprocated non-interacting protein pairs. Making a substitution for *P*_FN _in Equation 2, the problem reduces to a quadratic equation in one parameter, *P*_FP_:

$$\left(n+m\right)P_{FP}^{2}+\left(\Delta -2n\right)P_{FP}+n+\frac{\Delta^{2}}{4m}-\frac{n}{m}x_{2}=0$$

If we let

a = (*n *+ *m*), b = (Δ - 2*n*), and $\text{c}=n+\frac{\Delta^{2}}{4m}-\frac{n}{m}x_{2}$,

then an application of the quadratic formula gives two solutions for *p*_FP_:

$${\left(P_{FP}\right)}_{1,2}=\frac{-b\pm \sqrt{b^{2}-4ac}}{2a}$$

Then, substituting an estimate of P_FP _back into Equation 4 gives a solution for *P_FN_*. A similar argument carries through given any one of the three parameters {*n*,*P*_FP_,*P*_FN_}. Thus, an estimate of one of the parameters generates estimates of the other two.

#### Conditional hypergeometric, logistic regression tests, and two-way tables

We grouped the yeast genome into several defined subsets (VB, VP, VBP, and those proteins appearing to be affected by bias), and we wished to determine whether the subsets showed over-representation/under-representation among biologic categories such as GO, Kyoto Encyclopedia of Genes and Genomes, and Pfam. We used the conditional hypergeometric testing as described by Falcon and Gentleman [35] to probe for such over-representation/under-representation at a *P *value threshold of 0.01. A list of such GO categories and Pfam domains can be generated by the R scripts hgGO.R and hgPfam.R, contained with the Additional data files.

For those proteins that are affected by a systematic bias of each experiment, we fitted a logistic regression on these sets against 31 protein properties reported in the *Saccharomyces *Genome Database [45] and set a *P *value threshold at 0.01.

Let *S*_*i *_be the set of proteins identified to be affected by a systematic bias in dataset *i*, and suppose we wish to compare *S*_*i *_against *S*_*j*_; we define two methods of generating *S*_*i *_and *S*_*j *_for such a comparison. One method is the application of the binomial test on the *VBP *subgraph of each dataset *i *exclusively to determine each *S*_*i*_. The second method aims to streamline the experimental conditions of *i *with that of *j*. First, we compute *X *= *VBP*_*i *_∩ *VBP*_*j*_; then we apply the binomial test on the *X*_*i *_subgraph as well as the *X*_*j *_subgraph (because the edge-sets will be different). Obtaining such subsets allows us to generate a two-way table, *T*, to compare *S*_*i *_against *S*_*j*_. If the first method is used to generate the subsets *S*_*i *_and *S*_*j*_, then we must still restrict to *X *when computing *T*. T_(2,2) _counts |*S*_*i *_∩ *S*_*j*_|; *T*_(1,2) _and *T*_(2,1) _count |*S*_*i*_\*S*_*j*_| and |*S*_*j*_\*S*_*i*_|, respectively; and *T*_(1,1) _counts |$S_{i}^{c}\cap S_{j}^{c}$|. We can apply Fisher's exact test to ascertain the independence of these two sets at a designated *P *value threshold.

#### Per protein in-degree *z*-score and cross experimental comparisons

Let *o*_*p *_be the unreciprocated out-degree for a protein *p *and *i*_*p *_its unreciprocated in-degree. Then denote the number of unreciprocated edges by *n*_*p *_= *i*_*p *_+ *o*_*p*_. Assuming the distribution *i*_*p *_~ B(*n*_*p*_,$\frac{1}{2}$), we can compute the standardized in-degree (*z-score*) for *p*:

$$z_{p}=\frac{i_{p}-o_{p}}{\sqrt{i_{p}+o_{p}}}$$

#### Estimating the number of stochastic false positive/false negative observations

We used the filtered data after setting aside proteins rejected by the two-sided binomial tests to calculate the results presented in Tables 5 and 6. In the first case, we set *P*_FN _= 0, and *P*_FN _is the maximal value in the solution curve shown in Figure 6. *m *is estimated as $\left(\begin{array}{c} N\\ 2 \end{array}\right)$. The expected number of unreciprocated FP observations is 2*P*_FP_(1 - *P*_FP_)*m *and of reciprocated FP observations is $P_{FP}^{2}m$. In the second case, we set *P*_FP _= 0 and obtain *n *from the solution curve. The expected number of unreciprocated FN observations is 2*P*_FN_(1 - *P*_FN_)*n *and of reciprocated FN observations is $P_{FN}^{2}n$.

### Software implementation and availability

The R/Bioconductor packages used in the statistical analysis in this report are all available as freely distributed and open source software packages with an Artistic license. They are integrated into the R/Bioconductor environment for statistical computing and bioinformatics and run on operating systems Windows, Mac OS X, and Unix.

## Abbreviations

AP-MS, affinity purification-mass spectrometry; CAI, codon adaptation index; FN, false negative; FP, false positive; GO, Gene Ontology; TN, true negative; TP, true positive; VB, viable bait only; VBP, viable bait/prey; VP, viable prey only; Y2H, yeast two-hybrid.

## Authors' contributions

TC, RG and WH conceived and designed the investigations described in this report. TC, DS and DS performed the computational and statistical analyses. TC, RG and WH wrote paper. All authors read and approved the final version of the manuscript.

## Additional data files

The following additional data are available with the online version of this paper. Additional data file 1 demonstrates a full end-to-end analysis of the protein interaction datasets. Additional data file 2 is the Bioconductor package ppiStats (version 1.3.5 of 22 June 2007) in 'source' format. Additional data file 3 is the ppiStats package in the 'Windows Binary' format.

## Supplementary Material

Additional data file 1 Provided is a document demonstrating a full end-to-end analysis of the protein interaction datasets. Click here for file

Additional data file 2 Presented is the Bioconductor package ppiStats (version 1.3.5 of 22 June 2007) in 'source' format. ppiStats contains the novel methods developed in this paper. Click here for file

Additional data file 3 Presented is the Bioconductor package ppiStats in 'Windows binary' format. Click here for file
