# Supplementary material for: Coverage and error models of protein-protein interaction data by directed graph analysis
Source: Genome Biol. 2007 Sep 10;8(9):R186. doi: 10.1186/gb-2007-8-9-r186 (PMC2375024; doi:10.1186/gb-2007-8-9-r186)
Supplement: Additional data file 1 — Provided is a document demonstrating a full end-to-end analysis of the protein interaction datasets. [file gb-2007-8-9-r186-S1.gz › supp.pdf]

Supplementary Material for ”Coverage and Error Models of Protein-Protein Interaction Data by Directed Graph Analysis”

Tony Chiang Denise Scholtens Deepayan Sarkar
Robert Gentleman Wolfgang Huber

June 26, 2007

Contents

1 Introduction **2**

2 Obtaining the PPI Data **3**

3 Sampling and Coverage of the Interactome **5**
3.1 Analysis on the Bait/Prey Interactions 5
3.2 Hypergeometric Testing 8

4 Systematic Bias **9**
4.1 Probability model 9
4.1.1 Symmetry 10
4.1.2 Decomposition 10
4.1.3 Using in/out asymmetry to identify baits that are likely to be subject to systematic errors 11
4.2 Logistic Regressions 24
4.3 Results: log(abundance) as predictor 25
4.4 Results: log(YEPD-abundance) as predictor 26
4.5 Results: log(SD-abundance) as predictor 27
4.6 Results: log(CAI) as predictor 28
4.7 Fisher’s Exact Test Across Experiment 31
4.8 Unreciprocated Degree Statistics 37

5 Stochastic Error Analysis: Estimation of p_{fp} and p_{fn} by the method of moments 43
5.1 Derivation 43
5.2 Computation 44
5.2.1 Test on simulated data 44
5.3 Application to the PPI datasets 46

6 Stochastic Error Analysis: Estimation of Unreciprocated and Reciprocated FP/FN Errors within the Measured Data 47

7 Cross Data Integration and Analysis 51

Abstract

This compendium serves as a supplementary source of materials for the analysis presented in *Coverage and Error Models of Protein-Protein Interaction Data by Directed Graph Analysis.* We provide all necessary materials, methods, and code to reproduce the analysis, and present additional results.

1 Introduction

This technical report accompanies the paper *Coverage and Error Models of Protein-Protein Interaction Data by Directed Graph Analysis* by Chiang et al. It explains all the steps taken to perform the analysis of protein interaction data described in that paper. This report has been produced as a *reproducible document*: it contains all the computer instructions to reproduce the analysis and to create the figures, tables and numeric results of the paper. In addition, further analyses are produced that extend and support the main results described in the paper.

The production of the reproducible document employs the computational system and language R and the packages *ppiStats*, *ppiData*, and *yeastExpData*. You will need R version 2.4.1 or greater together with recent versions of the three packages and some other add-on packages that they depend upon and which can be obtain from CRAN or Bioconductor. To reproduce the computations shown here, you do not need to type them or copy-paste them from the PDF file; rather, you can take the file supp.Rnw in the doc directory of the *ppiStats* package, open it in a text editor, run it using the R command Sweave; and if you wish, modify the program it to your needs. Alternatively, if you would simply like the code without the surrounding text, you can call Rtangle on the supp.Rnw file to generate a script file called supp.R.

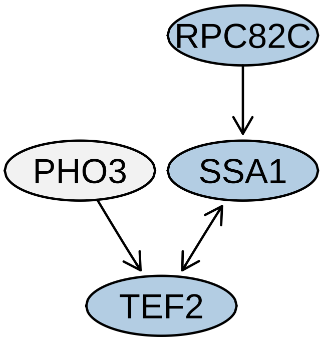

Figure 1: The graph shows the interaction data between four selected proteins from Krogan et al.'s experiment [10]. The bi-directional edge between the ATPase *SSA1* and the translational elongation factor *TEF2* indicates that either one as a bait pulled down the other one as a prey. The directed edge from *RPC82*, a subunit of RNA polymerase III, to *SSA1* indicates that *RPC82* as a bait pulled down *SSA1*, but not vice versa. Another unreciprocated edge goes from the phosphatase *PHO3* to *TEF2*. An investigation of the dataset shows that *PHO3*, which localizes in the periplasmatic space, was not reported in any interaction as a prey, while *RPC82C* was. In the interpretation of the data, we would have most confidence that there is a real interaction between *SSA1* and *TEF2*. We can differentiate between the two unreciprocated interactions: the one between *RPC82C* and *SSA1* has been bi-directionally tested, but only found once, while the other one has only been uni-directionally tested and found.

2 Obtaining the PPI Data

We begin by detailing the methods by which we obtained the 13 protein interaction datasets. We wanted data that had two properties: 1. information on the bait/prey data is preserved and 2. the prey population is documented as genome-wide. We downloaded the protein interaction data of [7, 14, 15, 1, 13, 5, 4] from the *IntAct* repository. We obtained [3, 6, 9, 10] from their primary sources. Having obtained the bait/prey protein interaction data, we created an R data-package *ppiData* where we stored this data. Each dataset is stored in *ppiData* as a directed graph object. As an example, we have selected 4 proteins from the dataset of [10] and rendered the vertex induced subgraph in Figure 1.

To make the dialogue clear, we first define some terms that will be used throughout this document:

Bait: A protein sampled for the purposes of ascertaining the proteins with which it interacts. The set of baits used in an experiment is the bait population.

Cloned Bait: A bait that was successfully cloned in a yeast cell with either a binding domain (Y2H) or a specified tag (AP-MS).

Viable Bait: A cloned bait that was observed to detect one or more proteins (prey).

Prey: A protein that is tested against the bait proteins. The set of prey used in an experiment is the prey population.

Cloned Prey: For Y2H, any prey that was successfully cloned in a yeast cell with an activation domain.

Viable Prey: A prey that was found to interact with a viable bait. Sometimes referred to as a *hit*.

Viable bait-prey: A protein that is both a viable bait and a viable prey.

The vector `bpExperimentNames` contains the names to each of the digraph objects. In addition, two list objects (called `viableBaits` and `viablePrey`) contain the viable baits and viable prey for each experimental dataset respectively. To represent all the data uniformly, the identifier for each protein is given by its corresponding Gene Locus Name. If the Gene Locus Name is unavailable, either the protein common name or another identifier (IntAct accession code, UniProt ID, etc) is used. We give some example code to show how to access this data.

```
> data("bpExperimentNames")
> bpExperimentNames
```

```
 [1] "ItoFull2001BPGraph" "Cagney2001BPGraph"
 [3] "Tong2002BPGraph"    "Hazbun2003BPGraph"
 [5] "Zhao2005BPGraph"    "Uetz2000BPGraph1"
 [7] "Uetz2000BPGraph2"   "Gavin2002BPGraph"
 [9] "Ho2002BPGraph"      "Krogan2004BPGraph"
[11] "Gavin2006BPGraph"   "Krogan2006BPGraph"
[13] "ItoCore2001BPGraph"
```

```
> gavin02 <- get(bpExperimentNames[8])
> gavin02

A graphNEL graph with directed edges
Number of Nodes = 1362
Number of Edges = 3418

> gavin02@nodes[1:4]

[1] "YBR236C" "YNR016C" "YLR359W" "YMR300C"
```

In addition to *ppiData*, another R-data package, *yeastExpData*, and the R package *ppiStats* were generated. *yeastExpData* contains R objects that contains published data on protein abundance. Yeast GFP fusion data, and a R dataframe consisting of 33 other yeast protein properties obtained from SGD (the dataframe is called `proteinProperties`). The *ppiStats* package contains all the statistical methods we have developed for the analysis of the directed protein interaction data.

3 Sampling and Coverage of the Interactome

3.1 Analysis on the Bait/Prey Interactions

We addressed the issue of coverage initially by the viable bait and viable prey population observed in the experimental datasets. From the directed graphs, we created the two lists `viableBaits` and `viablePrey` by asking if each protein as a vertex had non-zero out- and in-degree respectively modulo self-loops (i.e homomers). From these two lists, we were able to find the set theoretic intersections of the viable baits (VB) and viable prey (VP) per experiment to ascertain the viable bait/prey (VBP) populations.

```
> getVBP <- function() {
+     vbp <- list()
+     for (g in bpExperimentNames) {
+         m = as(get(g), "matrix")
+         diag(m) = 0
+         stopifnot(identical(rownames(m), colnames(m)))
+         vbpEach = rownames(m)[(rowSums(m) > 0) &
+             (colSums(m) > 0)]
+         vbp[[g]] <- vbpEach
+     }
```

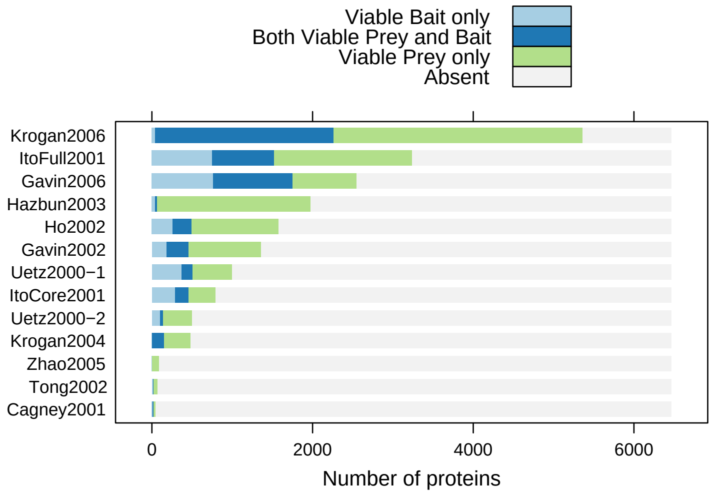

Figure 2: This bar chart shows the proportion of proteins sampled either as a viable bait (VB), a viable prey (VP), or as both (VBP). With the exception of the Krogan et al. [10]'s data, the other eleven show large portions of the yeast genome which did not participate in any positive interactions. Without additional information, there is little we can do to elucidate whether these proteins were tested but inactive for all tests, or whether these proteins were not tested.

```
+     return(vbp)
+ }
> vbp <- getVBP()
```

From SGD, we used 6466 as the number of known and characterized yeast ORFs. This allowed us to build bar charts (cf Figure 2) to gauge the proportion of the yeast interactome tested by each experimental dataset.

In addition to the bar chart, we were able to generate a number of coverage statistics on the Y2H and APMS datasets on a per experiment setting (cf Table 6), as well as between experiments of the same type, i.e. those which used the same system to test interactions (cf Table 7 and Table 8).

Before we conduct any other statistical tests on the protein interaction data, we list the definitions of some standard statistical terms in Table 1. Any of these terms used throughout this document (as well as the article *Coverage and Error Models in Protein Interaction Data by Directed Graph Analysis*) correspond to the given definitions.

Error Statistics		
True Positives	TP	Number of cases in which a true interaction is experimentally observed.
True Negatives	TN	Number of cases in which two proteins do not interact, their interaction is tested, but not observed.
False Positives	FP	Number of cases in which two proteins do not interact, but an interaction is reported by the experiment.
False Negatives	FN	Number of cases in which a true interaction is experimentally tested and not found.
True Tested Interactions	P	TP+FN
True Tested Non-interactions	N	TN+FP
False Positive Rate	p_{FP}	Probability that a truely absent interaction is detected. It can be estimated by FP / N.
False Negative Rate	p_{FN}	Probability that a true interaction is not detected. It can be estimated by FN / P.
Sensitivity		Probability that a true interaction is detected. It can be estimated by TP / P.
Specificity		Probability that a truely absent interaction is not detected, estimated by TN / N.
False Discovery Rate	FDR	Informally, the expected value of FP/(TP+FP) [12].
Positive Predictive Value	PPV	Probability that an observed interaction is indeed true. It can be estimated by TP / (TP+FP).
Negative Predictive Value	NPV	Probability that an observed non-interaction is truely absent. It can be estimated by TN / (TN+FN).

Table 1: Standard definitions of various error terms [8]. The probabilities are conditional on that the interaction is tested.

.

3.2 Hypergeometric Testing

We wanted to ascertain if the viably tested proteins showed signs of being affected by a coverage bias in the experimental assay. To investigate, we used the conditional hypergeometric tests described by [2] to test for over/under representation in GO categories. Using the R software packages *Category* and *GOstats*, we were able to asses these questions. For our purposes, we used a p-value cutoff at the 10^{-2} threshold. We were only interested in GO categories which contained at least 50 unique annotations as well. Both these parameters can be set by the user, and those familiar with the R programming language are free to manipulate these parameters within the R scripts.

The code written to conduct these hypergeometric tests has been supplied with the main article as an additional file. It can also be found in the Scripts directory of the *ppiStats* package. The file `hgGO.R` is a script file which computes the conditional hypergeometric testing on the GO directed acyclic graph (DAG). The file `hgPfam.R` computes the hypergeometric testing on Pfam domains. Running these scripts will generate several *.html* files which provide the results to the hypergeometric analysis.

We provide one example of the hypergeometric test in the following code chunck:

```
> geneSet1 <- viableBaits[[length(viableBaits)]]
> parameter1 <- ppiBuildParams4GO(geneSet = geneSet1,
+     universe = yeastGenome, direction = "over",
+     ontology = "CC", cond = TRUE, pThresh = 0.01)
> hg1 <- ppiHGTest4GO(parameter1, filename = names(viableBaits)[1],
+     label = names(viableBaits)[1], typeGeneSet = "Viable Baits",
+     cs = 50)

[1] "Processing ItoFull2001BPGraph for over representation in the  CC GO ontology."

> parameter1@testDirection <- "under"
> hg2 <- ppiHGTest4GO(parameter1, filename = names(viableBaits)[1],
+     label = names(viableBaits)[1], typeGeneSet = "Viable Baits",
+     cs = 50)

[1] "Processing ItoFull2001BPGraph for under representation in the  CC GO ontology."

> pvalues(hg1)[pvalues(hg1) < hg1@pvalueCutoff]
```

```
  GO:0044424   GO:0031965   GO:0005643   GO:0030660
1.891263e-06 3.729540e-06 1.398803e-05 5.512150e-05
  GO:0030658   GO:0000780   GO:0005634   GO:0000777
5.512150e-05 2.896615e-04 3.152791e-04 4.358567e-04
  GO:0005952   GO:0000793   GO:0044430   GO:0030117
4.460608e-04 5.239024e-04 1.164743e-03 1.212418e-03
  GO:0030127   GO:0015630   GO:0000775   GO:0000932
1.850904e-03 1.905391e-03 1.921109e-03 1.975141e-03
  GO:0012506   GO:0030662   GO:0005623   GO:0010008
2.786583e-03 2.786583e-03 2.816449e-03 2.987964e-03
  GO:0044439   GO:0005876   GO:0042729   GO:0042579
3.405520e-03 3.917458e-03 4.906094e-03 7.018767e-03
  GO:0030121
7.484728e-03
```

```
> pvalues(hg2)[pvalues(hg2) < hg2@pvalueCutoff]
```

```
  GO:0005739   GO:0031966   GO:0030312   GO:0009277
6.900628e-09 9.710471e-04 3.281781e-03 3.281781e-03
  GO:0005842
7.719654e-03
```

Even though the hypergeometric tests show that there are 25 number of GO terms which are over-represented by the viable baits of *Ito-Core* dataset, we have decided only to investigate those GO terms which at least 50 annotated genes.

4 Systematic Bias

4.1 Probability model

For a protein ρ from VBP, we want to construct a probability model for the joint distribution of N_R, the number of reciprocated edges, N_I, the number of unreciprocated in-edges, and N_O, the number of unreciprocated out-edges, given the true degree δ^* and the parameters p_{fp}, p_{fn} and N is the number of interesting proteins.

We will use the shortcut $N_U = N_I + N_O$ for the total number of unreciprocated edges, and $\Theta = (\delta^*, p_{\text{fp}}, p_{\text{fn}}, N)$ for the parameters.

We consider

$$
\begin{aligned}
& P\left(N_R = n_r, N_I = n_i, N_O = n_o\,;\,\Theta\right) \\
= \quad & P(N_I = n_i, N_O = n_u - n_i \mid N_U = n_u, N_R = n_r\,;\Theta) \\
& \times P(N_U = n_u, N_R = n_r\,;\,\Theta) \qquad (1)
\end{aligned}
$$

The decomposition of P in the right hand side will be useful.

For convenience, we suppress the index ρ in our notation, but please keep in mind that the parameter $\delta^* \equiv \delta^*_\rho$ depends on ρ, and that N_R, N_I, N_O and N_U are random variables that depend on ρ. N is an experiment-wide parameter, and we also consider p_{fp} and p_{fn} to be experiment-wide; although some of what follows might also apply to a model where p_{fp} and p_{fn} depend on ρ, if there were data to estimate them.

We will now make some modeling assumptions. If we find that the data for a particular protein does not concur well with these assumptions, we will consider it subject to systematic error.

4.1.1 Symmetry

The first assumption is that of symmetry, that is, equality of the distributions of N_I and N_O.

$$N_I =_d N_O \qquad (2)$$

and in particular

$$(N_I | N_U = n_u) \sim \mathrm{B}(n_u, \frac{1}{2}). \qquad (3)$$

This gives us the first term on the RHS of (1). The remarkable thing is that it depends on n_u, but not on any of the parameters! Now for the second term:

4.1.2 Decomposition

We can decompose N_R and N_U into those counts that originate from real interactions (i. e. that are true) and those that originate from false positive measurements.

$$
\begin{aligned}
N_R &= N_R^v + N_R^f \qquad (4) \\
N_U &= N_U^v + N_U^f \qquad (5)
\end{aligned}
$$

The false positives are easy:

$$
\begin{aligned}
N_R^f &\sim \mathrm{B}(N - \delta^* - 1,\, p_{\mathrm{fp}}^2) \\
N_U^f &\sim \mathrm{B}(N - \delta^* - 1,\, 2p_{\mathrm{fp}}(1 - p_{\mathrm{fp}})) \qquad (6)
\end{aligned}
$$

The ones that originate from a real interaction follow a multinomial distribution

$$
\begin{aligned}
& P(N_R^v = n_r^v,\, N_U^v = n_u^v \,|\, \Theta) \\
= \;& (1-p)^{2n_r^v} \cdot (2p(1-p))^{n_u^v} \cdot p^{2n_{\text{none}}^v} \cdot \frac{\delta^*!}{n_r^v! n_u^v! n_{\text{none}}^v!} \qquad (7)
\end{aligned}
$$

where for notational convenience we used the abbreviations $n_{\text{none}}^v = \delta^* - n_r^v - n_u^v$ and $p \equiv p_{\text{fn}}$.

The density function of the second term on the RHS of (1) can then be obtained by convolution of (6) and (7). For each value of the parameters $\Theta \equiv (N, \delta^*, p_{\text{fp}}, p_{\text{fn}})$, this is a 2D matrix with infinite numbers of rows and columns, corresponding to n_r and n_u. Most of the probability mass, however, is concentrated within a bounded range. Furthermore, we will restrict our attention to values of δ^* between 0 and δ^*_{max}, depending on the data set. This is implemented in the function `nullDistDoublyTestedEdges` in the package *ppiStats*.

4.1.3 Using in/out asymmetry to identify baits that are likely to be subject to systematic errors

We now use Equation (3) to assign a p-value to each protein. For a protein with unreciprocated degrees (n_i, n_o), the p-value is

$$
\begin{aligned}
p(n_i, n_o) &= P(\min\{N_I, N_O\} \leq \min\{n_i, n_o\}) \\
&= \max\{2P(N_I \leq \min\{n_i, n_o\}),\, 1\} \qquad (8)
\end{aligned}
$$

This is computed by the following function `assessSymmetry` which is also contained in the R package *ppiStats*. In addition, the function also calculates the contours of the function p in the (n_i, n_o)-plane. These will be used in the plots.

Now we are ready to apply the symmetry p-values, and we will create an environment, `bpRed` containing the reduced set of data with only proteins with p-values larger than or equal to p-value threshold of 10^{-2}.

For a more illuminating visual effect, we have perturbed the data on each point of the figures. This perturbation shows the relative concentration of data for each point in each of the figures.

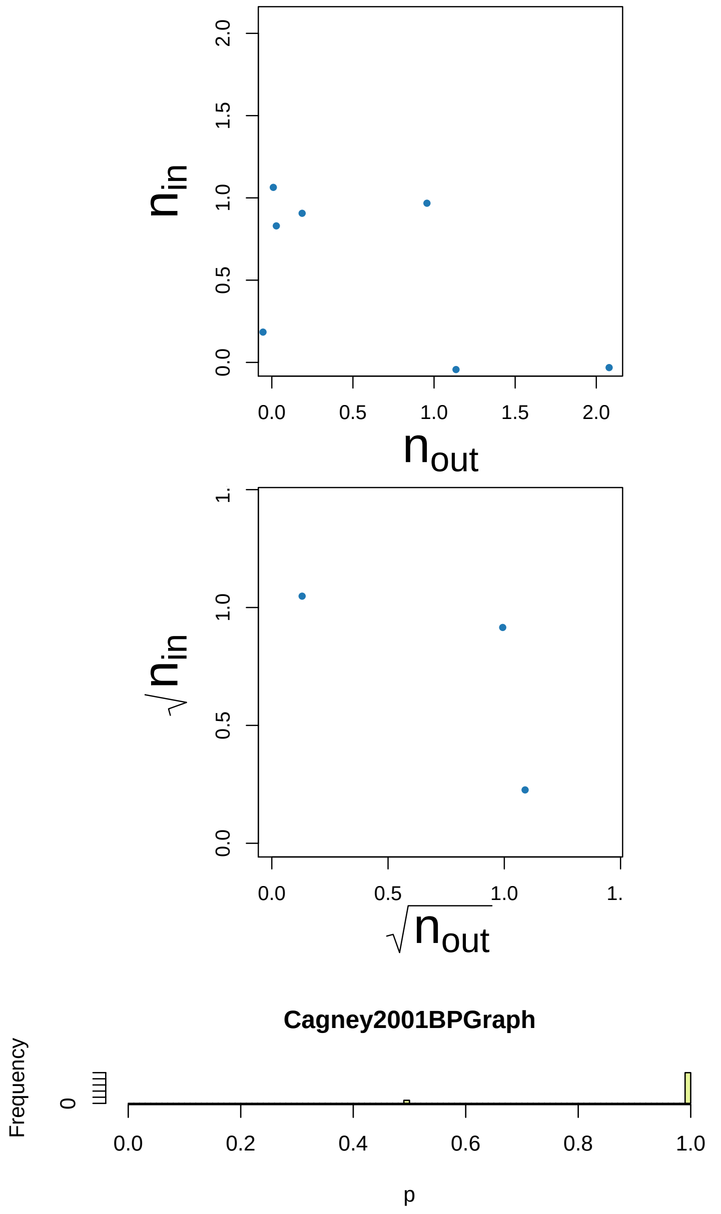

Figure 3: Scatterplots of in- and out-degree and symmetry p-values for Cagney2001BPGraph

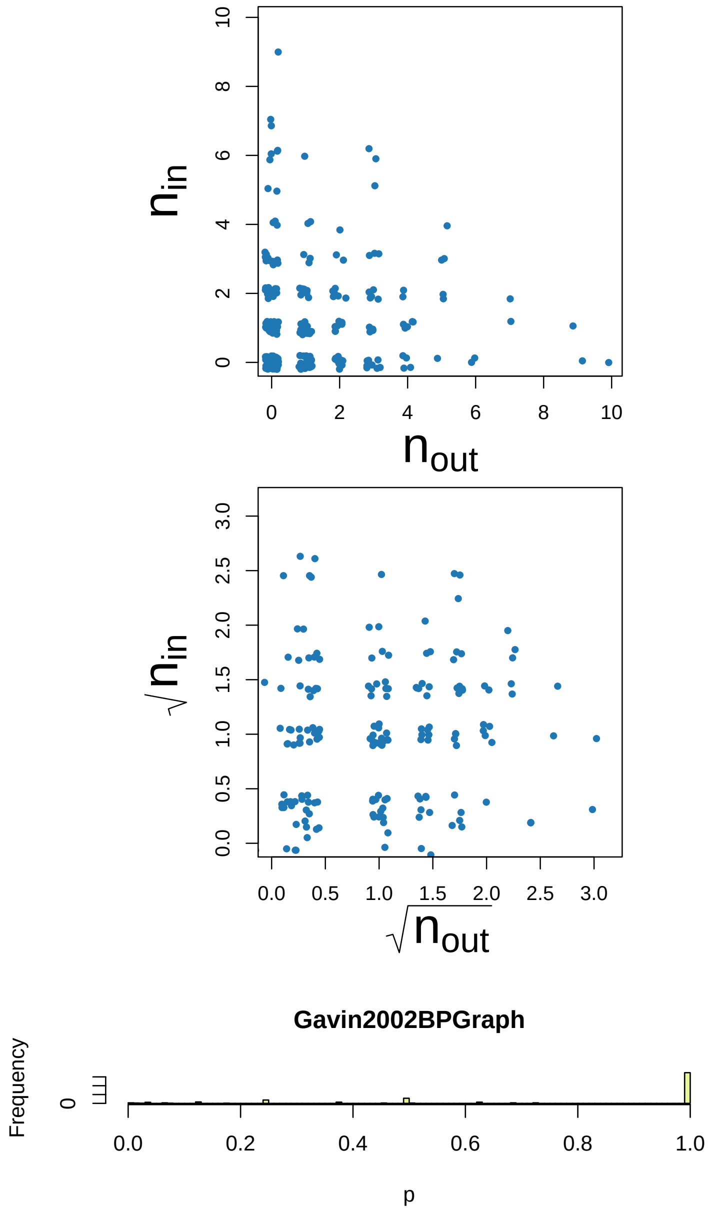

Figure 4: Scatterplots of in- and out-degree and symmetry p-values for Gavin2002BPGraph

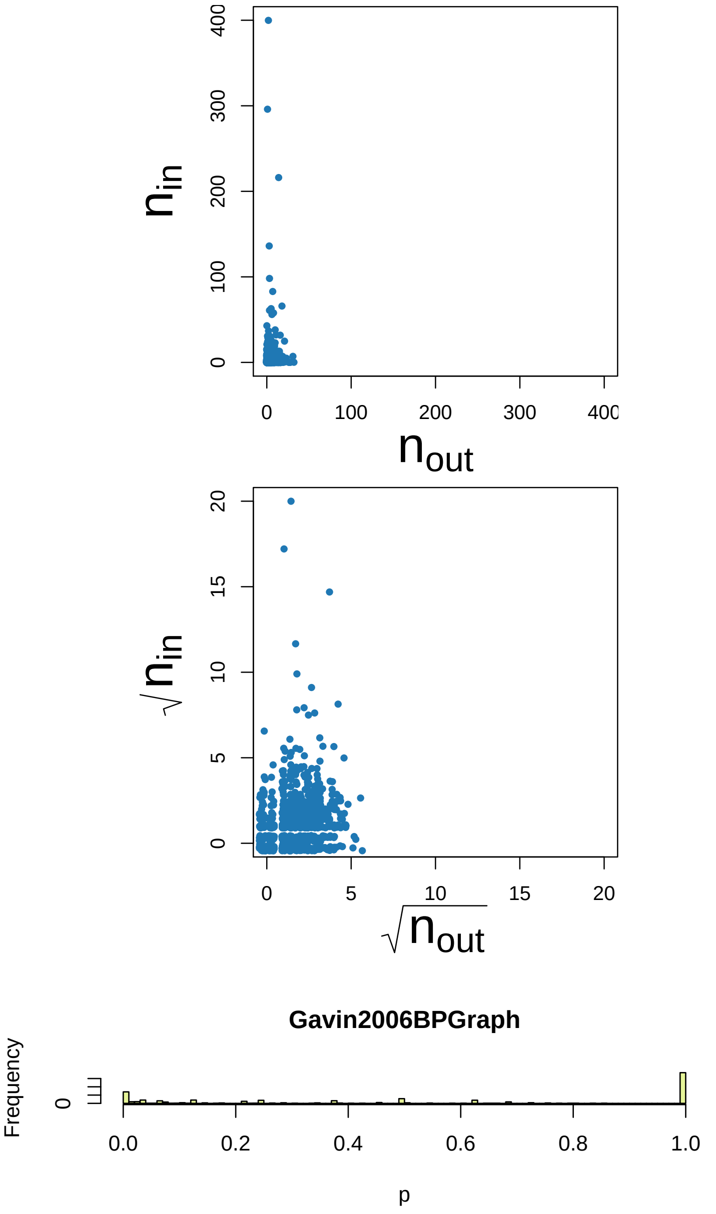

Figure 5: Scatterplots of in- and out-degree and symmetry p-values for Gavin2006BPGraph

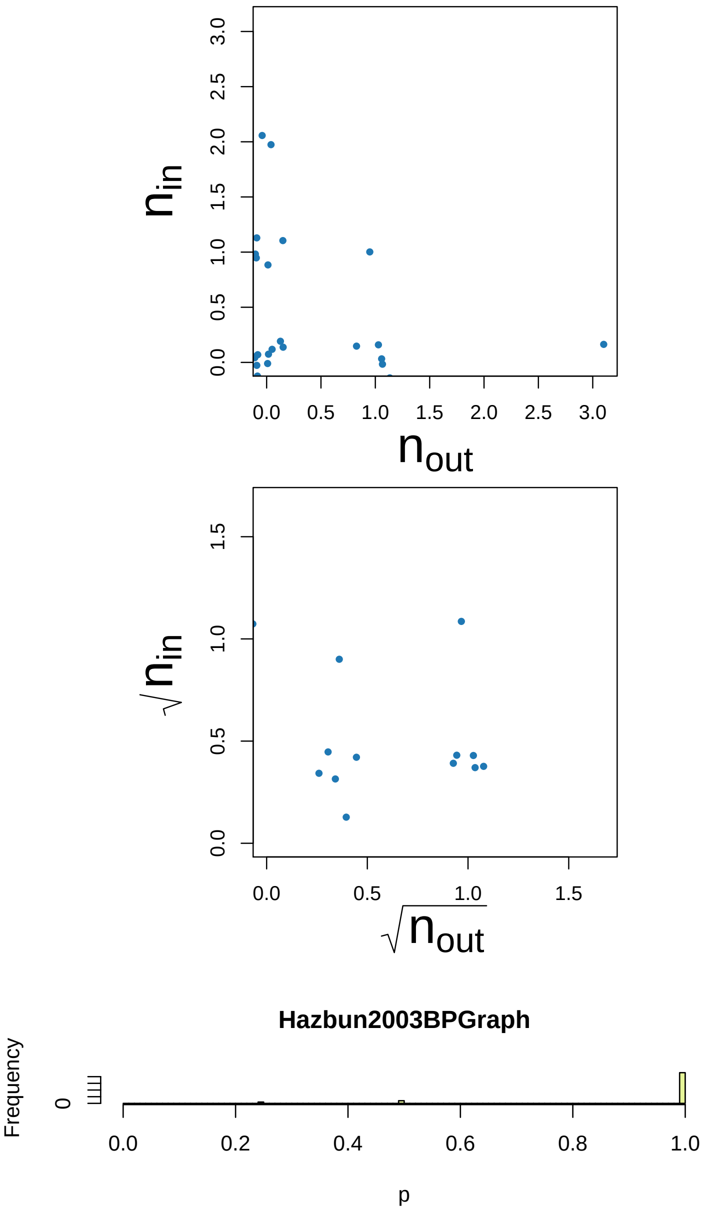

Figure 6: Scatterplots of in- and out-degree and symmetry p-values for Hazbun2003BPGraph

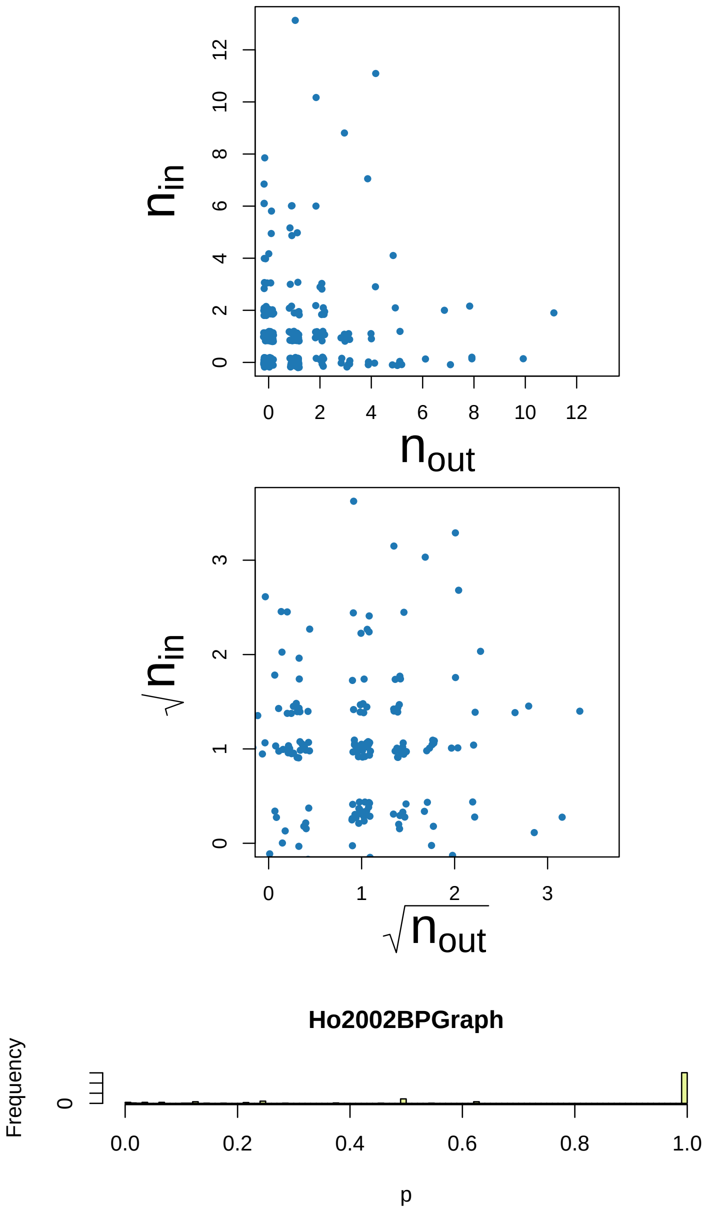

Figure 7: Scatterplots of in- and out-degree and symmetry p-values for Ho2002BPGraph

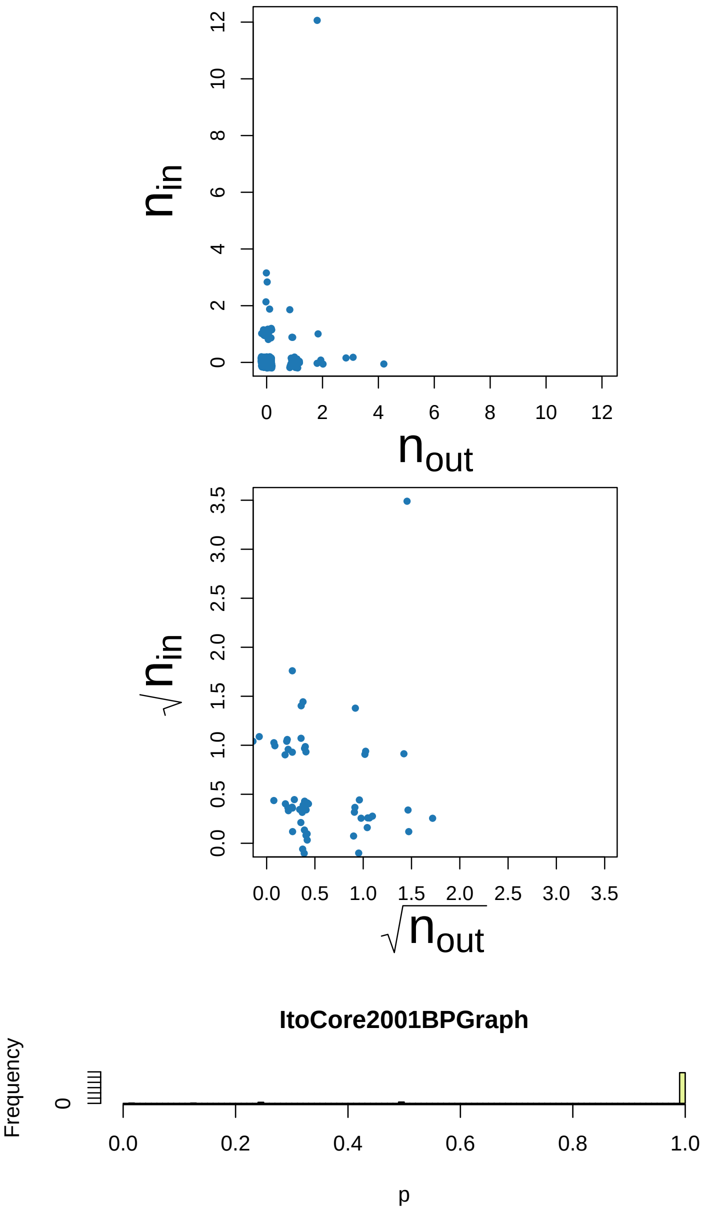

Figure 8: Scatterplots of in- and out-degree and symmetry p-values for ItoCore2001BPGraph

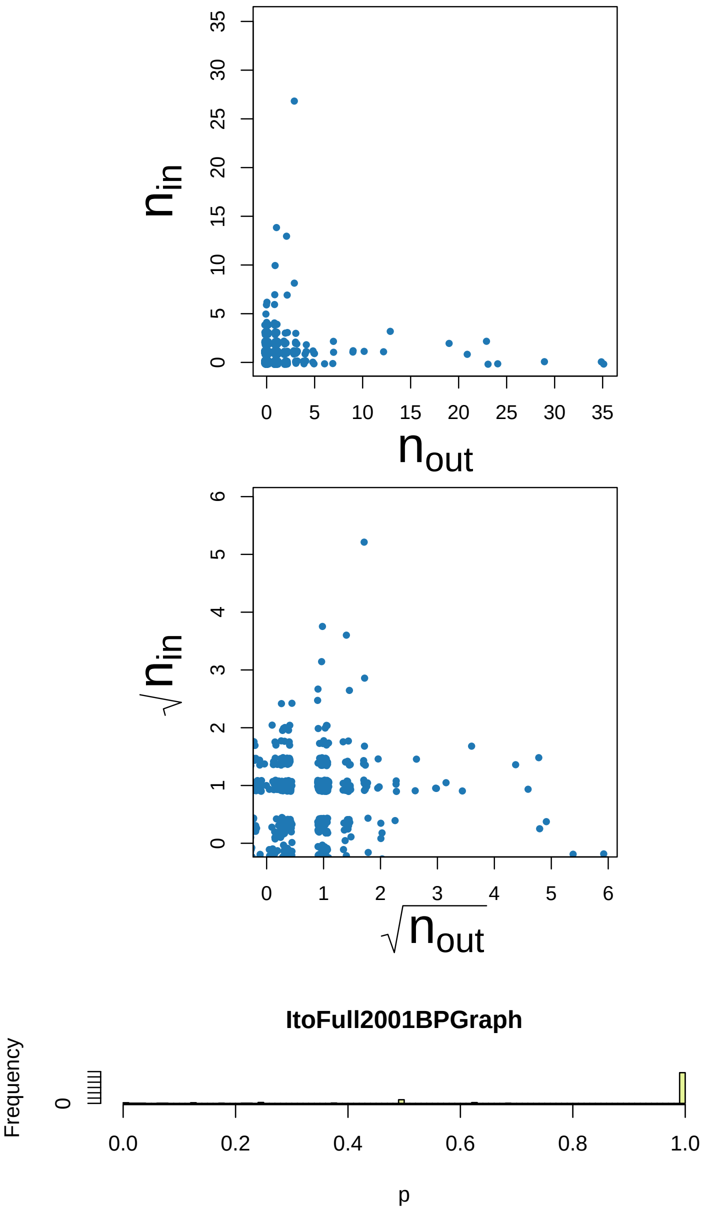

Figure 9: Scatterplots of in- and out-degree and symmetry p-values for ItoFull2001BPGraph

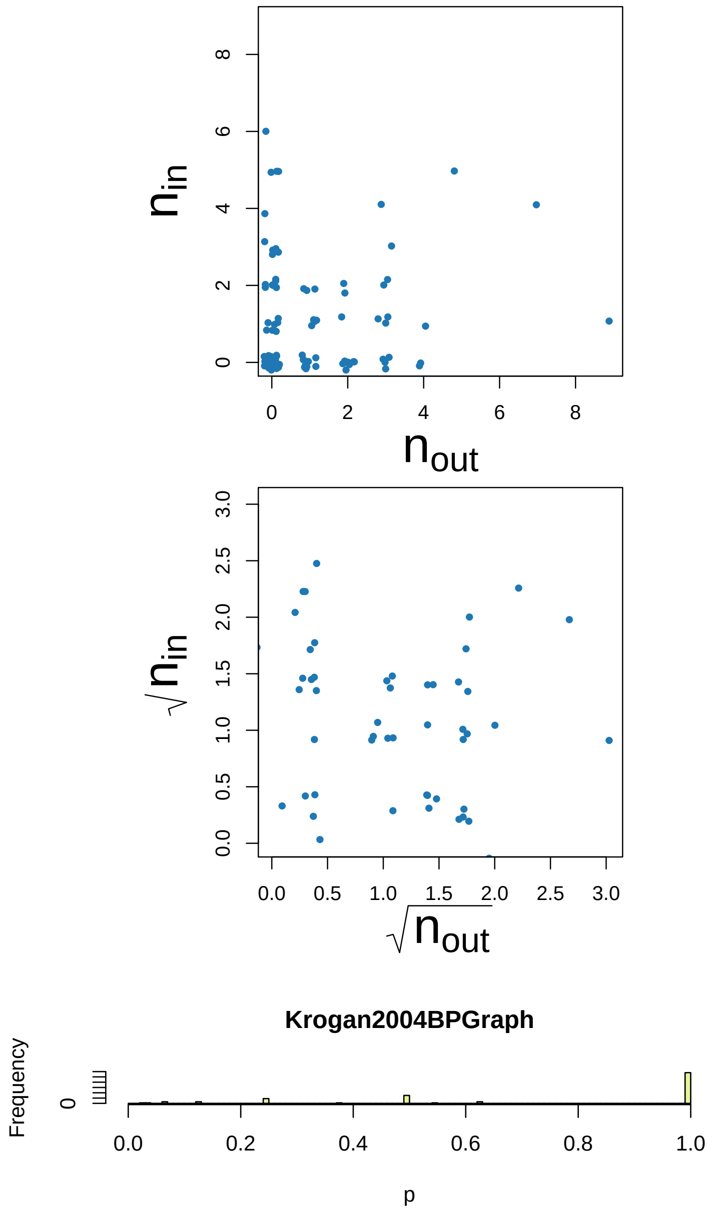

Figure 10: Scatterplots of in- and out-degree and symmetry p-values for Krogan2004BPGraph

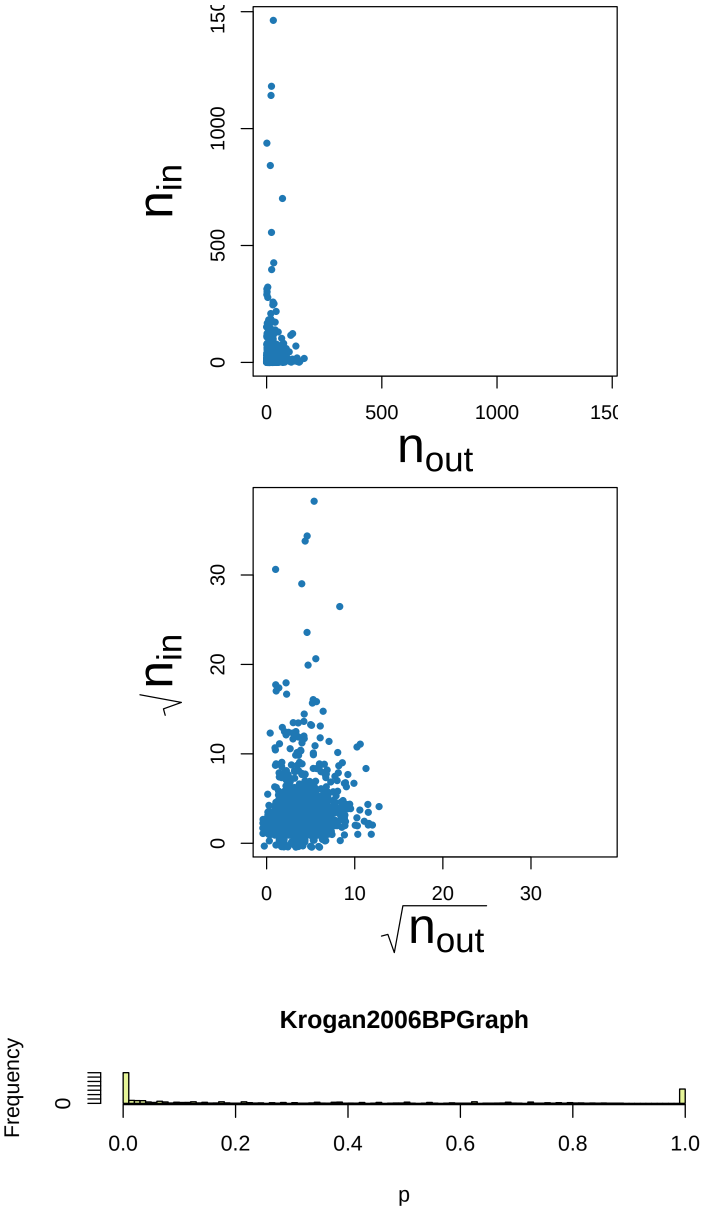

Figure 11: Scatterplots of in- and out-degree and symmetry p-values for Krogan2006BPGraph

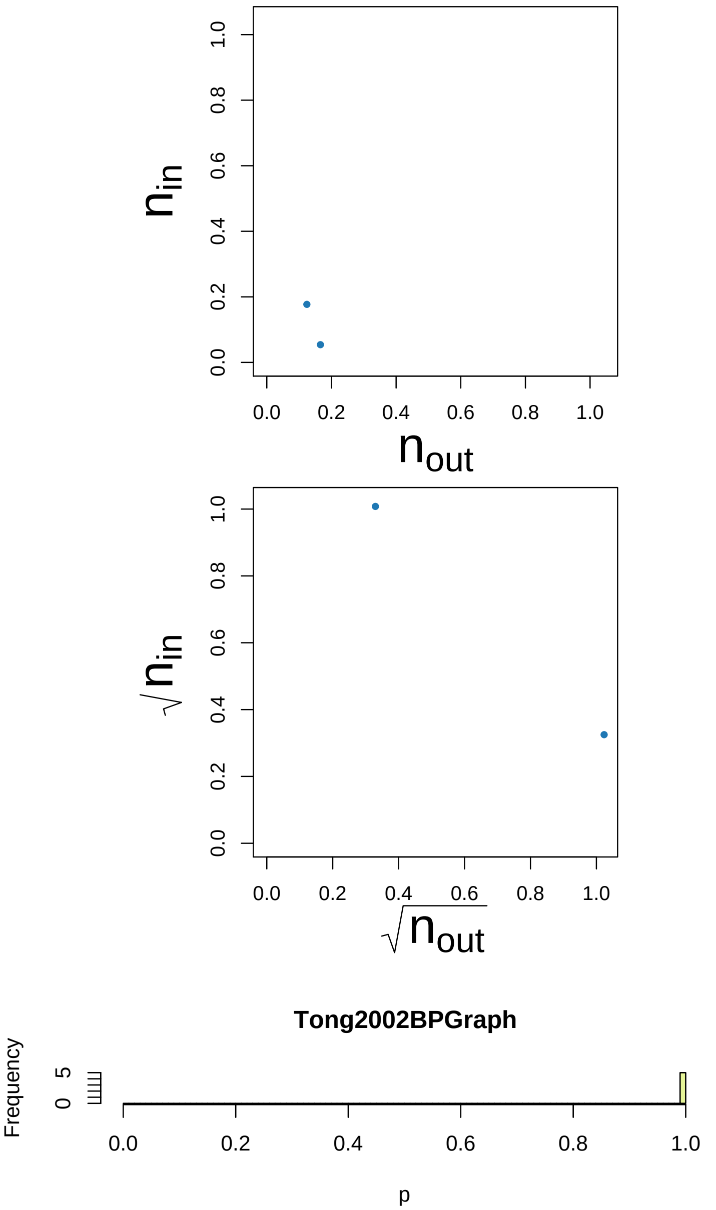

Figure 12: Scatterplots of in- and out-degree and symmetry p-values for Tong2002BPGraph

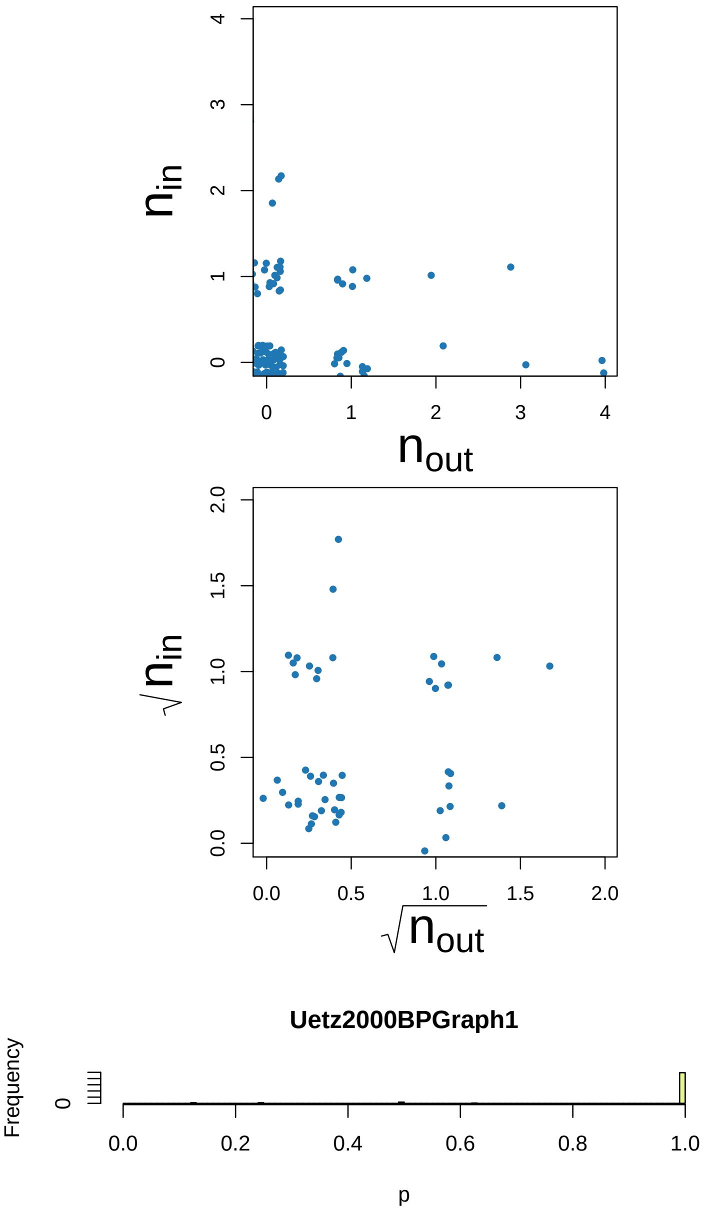

Figure 13: Scatterplots of in- and out-degree and symmetry p-values for Uetz2000BPGraph1

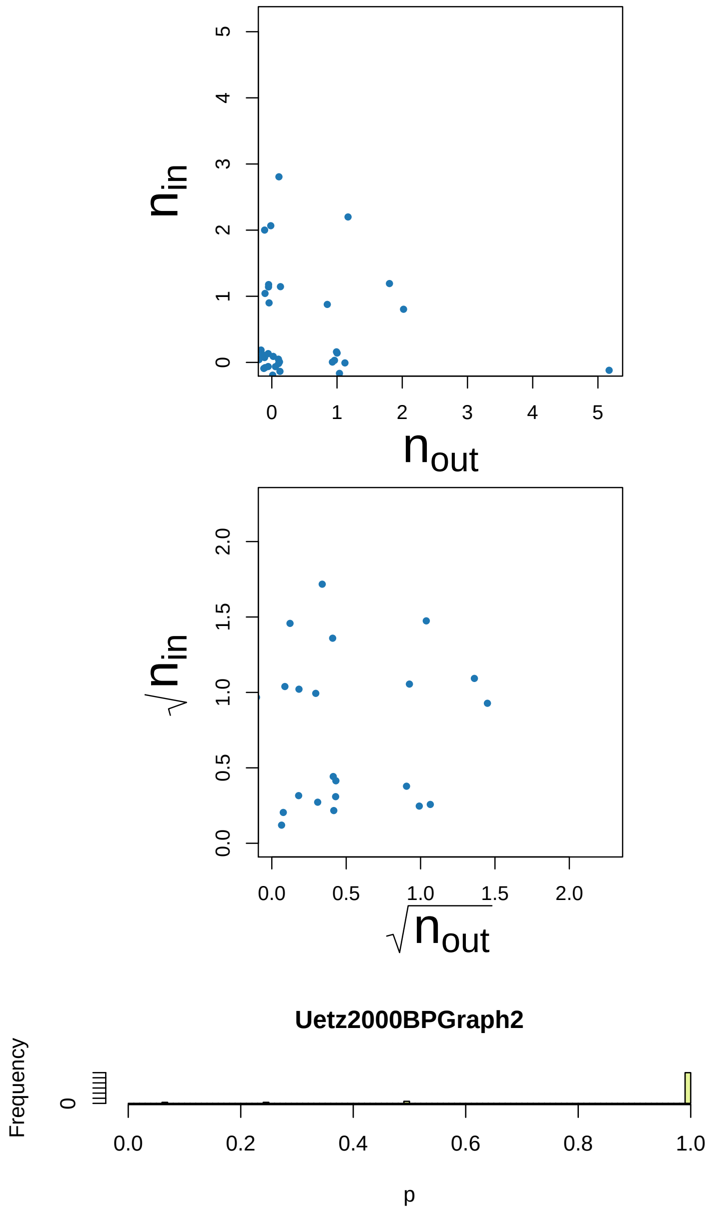

Figure 14: Scatterplots of in- and out-degree and symmetry p-values for Uetz2000BPGraph2

4.2 Logistic Regressions

For a protein with n_i unreciprocated in-edges and n_o unreciprocated out-edges, we expect

$$n_i \mid n_u \quad \sim \mathcal{B}\left(n_u, \frac{1}{2}\right)$$

if false positive and false negative errors are independent of a protein's properties. Let p be the true probability ($H_0 : p = \frac{1}{2}$) for any particular protein. We will

- Perform binomial tests for $H_1 : p < \frac{1}{2}$ and $H_1 : p > \frac{1}{2}$ for each protein (in each experiment)
- Use test outcomes as responses to fit logistic regressions with abundance and CAI as predictor [1].

Regression is restricted to the subgraph of proteins that are VBP.

[1]We actually use logarithm (base 2) of abundance and CAI as predictor since that has a much more symmetric distribution.

4.3 Results: log(abundance) as predictor

Systematic := unusually large in-degree

	Estimate	Std. Error	Pr(>|z|)
ItoFull2001BPGraph	-0.0965484	1.568464e-01	0.5381848
Cagney2001BPGraph	0.0000000	6.277441e+04	1.0000000
Tong2002BPGraph	0.0000000	3.100286e+04	1.0000000
Hazbun2003BPGraph	0.0000000	2.857672e+04	1.0000000
Uetz2000BPGraph1	0.0000000	2.693089e+04	1.0000000
Uetz2000BPGraph2	0.0000000	1.858329e+04	1.0000000
Gavin2002BPGraph	-0.0455738	2.071982e-01	0.8259080
Ho2002BPGraph	0.6177581	2.172607e-01	0.0044636
Krogan2004BPGraph	0.3048115	2.155429e-01	0.1573158
Gavin2006BPGraph	0.2470921	5.727970e-02	0.0000160
Krogan2006BPGraph	0.1747114	2.817510e-02	0.0000000
ItoCore2001BPGraph	-0.9033460	8.231313e-01	0.2724444

Systematic := unusually large out-degree

	Estimate	Std. Error	Pr(>|z|)
ItoFull2001BPGraph	-0.0673062	1.272444e-01	0.5968387
Cagney2001BPGraph	0.0000000	6.277441e+04	1.0000000
Tong2002BPGraph	0.0000000	3.100286e+04	1.0000000
Hazbun2003BPGraph	0.0000000	2.857672e+04	1.0000000
Uetz2000BPGraph1	0.0000000	2.693089e+04	1.0000000
Uetz2000BPGraph2	-0.1741740	4.273137e-01	0.6835658
Gavin2002BPGraph	0.0612910	2.069047e-01	0.7670560
Ho2002BPGraph	-0.0355630	1.760731e-01	0.8399333
Krogan2004BPGraph	0.4262939	3.195259e-01	0.1821563
Gavin2006BPGraph	0.0317204	4.039910e-02	0.4323495
Krogan2006BPGraph	0.0205081	2.025180e-02	0.3112261
ItoCore2001BPGraph	0.0000000	2.540830e+04	1.0000000

4.4 Results: log(YEPD-abundance) as predictor

Systematic := unusually large in-degree

	Estimate	Std. Error	Pr(>|z|)
ItoFull2001BPGraph	-0.2034193	2.539516e-01	0.4231223
Cagney2001BPGraph	0.0000000	4.015988e+04	1.0000000
Tong2002BPGraph	0.0000000	3.141114e+04	1.0000000
Hazbun2003BPGraph	0.0000000	5.718971e+04	1.0000000
Uetz2000BPGraph1	0.0000000	2.382507e+04	1.0000000
Uetz2000BPGraph2	0.0000000	4.779005e+04	1.0000000
Gavin2002BPGraph	0.0314538	2.138131e-01	0.8830462
Ho2002BPGraph	0.6995187	2.623436e-01	0.0076663
Krogan2004BPGraph	0.7332109	3.110509e-01	0.0184130
Gavin2006BPGraph	0.3310486	6.616190e-02	0.0000006
Krogan2006BPGraph	0.3342046	3.750350e-02	0.0000000
ItoCore2001BPGraph	0.0000000	2.795295e+04	1.0000000

Systematic := unusually large out-degree

	Estimate	Std. Error	Pr(>|z|)
ItoFull2001BPGraph	0.0639316	1.714852e-01	0.7092889
Cagney2001BPGraph	0.0000000	4.015988e+04	1.0000000
Tong2002BPGraph	0.0000000	3.141114e+04	1.0000000
Hazbun2003BPGraph	0.0000000	5.718971e+04	1.0000000
Uetz2000BPGraph1	0.0000000	2.382507e+04	1.0000000
Uetz2000BPGraph2	-172.3940519	3.625785e+05	0.9996206
Gavin2002BPGraph	-0.2873317	2.926935e-01	0.3262569
Ho2002BPGraph	-0.0839760	2.566496e-01	0.7435160
Krogan2004BPGraph	0.1809569	4.022975e-01	0.6528484
Gavin2006BPGraph	0.0040555	5.339530e-02	0.9394565
Krogan2006BPGraph	-0.0341308	2.937090e-02	0.2452114
ItoCore2001BPGraph	0.0000000	2.795295e+04	1.0000000

4.5 Results: log(SD-abundance) as predictor

Systematic := unusually large in-degree

	Estimate	Std. Error	Pr(>|z|)
ItoFull2001BPGraph	-0.0371755	2.534341e-01	0.8833791
Cagney2001BPGraph	0.0000000	4.652810e+04	1.0000000
Tong2002BPGraph	0.0000000	3.249386e+04	1.0000000
Hazbun2003BPGraph	0.0000000	5.032356e+04	1.0000000
Uetz2000BPGraph1	0.0000000	2.639200e+04	1.0000000
Uetz2000BPGraph2	0.0000000	6.453553e+04	1.0000000
Gavin2002BPGraph	-0.0065802	2.286774e-01	0.9770439
Ho2002BPGraph	0.7473637	2.624720e-01	0.0044077
Krogan2004BPGraph	0.7137744	3.118868e-01	0.0221044
Gavin2006BPGraph	0.3000127	6.650650e-02	0.0000065
Krogan2006BPGraph	0.3312490	3.870910e-02	0.0000000
ItoCore2001BPGraph	0.0000000	2.900438e+04	1.0000000

Systematic := unusually large out-degree

	Estimate	Std. Error	Pr(>|z|)
ItoFull2001BPGraph	0.2229019	1.574997e-01	0.1569944
Cagney2001BPGraph	0.0000000	4.652810e+04	1.0000000
Tong2002BPGraph	0.0000000	3.249386e+04	1.0000000
Hazbun2003BPGraph	0.0000000	5.032356e+04	1.0000000
Uetz2000BPGraph1	0.0000000	2.639200e+04	1.0000000
Uetz2000BPGraph2	0.0000000	6.453553e+04	1.0000000
Gavin2002BPGraph	-0.4646590	3.950941e-01	0.2395662
Ho2002BPGraph	-0.0817470	2.732001e-01	0.7647722
Krogan2004BPGraph	-0.2072876	4.624194e-01	0.6539602
Gavin2006BPGraph	-0.0367753	5.514980e-02	0.5048840
Krogan2006BPGraph	-0.0662342	3.091590e-02	0.0321613
ItoCore2001BPGraph	0.0000000	2.900438e+04	1.0000000

4.6 Results: log(CAI) as predictor

```
Systematic := unusually large in-degree

                      Estimate   Std. Error  Pr(>|z|)
ItoFull2001BPGraph  0.1324551 4.233209e-01 0.7543603
Cagney2001BPGraph   0.0000000 2.212620e+05 1.0000000
Tong2002BPGraph     0.0000000 2.902217e+05 1.0000000
Hazbun2003BPGraph   0.0000000 1.279461e+05 1.0000000
Uetz2000BPGraph1    0.0000000 8.274419e+04 1.0000000
Uetz2000BPGraph2    0.0000000 6.164974e+04 1.0000000
Gavin2002BPGraph   -0.6442715 7.577149e-01 0.3951682
Ho2002BPGraph       1.4918111 4.683387e-01 0.0014459
Krogan2004BPGraph   2.0058012 7.165575e-01 0.0051226
Gavin2006BPGraph    1.0523019 1.623679e-01 0.0000000
Krogan2006BPGraph   0.9233587 8.326850e-02 0.0000000
ItoCore2001BPGraph  0.1964774 1.869238e+00 0.9162878
```

```
Systematic := unusually large out-degree

                      Estimate   Std. Error  Pr(>|z|)
ItoFull2001BPGraph -0.1525036 4.013274e-01 0.7039470
Cagney2001BPGraph   0.0000000 2.212620e+05 1.0000000
Tong2002BPGraph     0.0000000 2.902217e+05 1.0000000
Hazbun2003BPGraph   0.0000000 1.279461e+05 1.0000000
Uetz2000BPGraph1    0.0000000 8.274419e+04 1.0000000
Uetz2000BPGraph2   -0.2867229 2.038068e+00 0.8881199
Gavin2002BPGraph   -1.5156465 9.808245e-01 0.1222791
Ho2002BPGraph      -1.3921662 8.686472e-01 0.1090046
Krogan2004BPGraph   0.6392686 1.114364e+00 0.5661964
Gavin2006BPGraph   -0.0393550 1.425905e-01 0.7825482
Krogan2006BPGraph  -0.1703917 7.103590e-02 0.0164547
ItoCore2001BPGraph -0.8348575 2.378194e+00 0.7255532
```

In addition to the Logistic Regressions, we plotted the adjacency matrix diagram of the bait/prey interactions in two different ways: 1. the rows and columns randomly ordered and 2. the rows and columns ordered by ascending CAI (cf Figure 15 and Figure 16). This readily gives a visual method of identifying the association between CAI and proteins rejecting the 2-sided Binomial test.

There has been a number of research articles that point to the relationship between CAI and protein abundance. To verify this fact, we computed

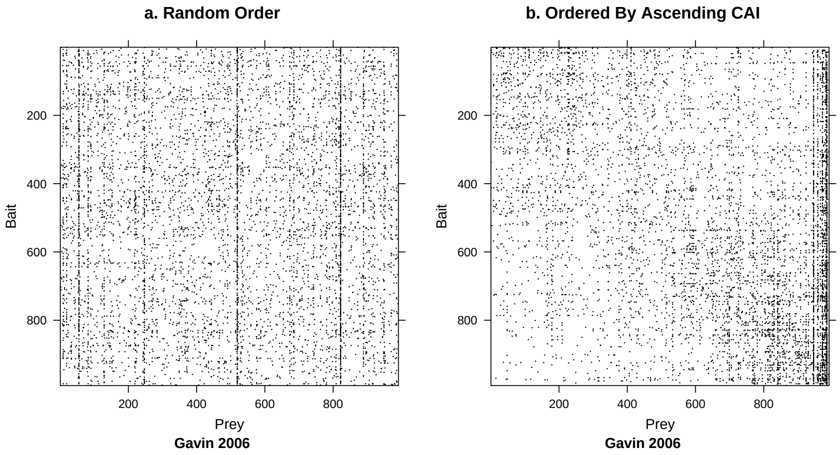

Figure 15: These plots present a view of the adjacency matrix for the VBP derived from the Gavin et al's. [4] experimental data set. An interaction between bait b and prey p is recorded by a dark pixel in $(b,p)^{th}$ position of the matrix. The left panel has the rows and columns randomly ordered while the right panel has the rows and columns ordered by ascending values of each protein's condon adaptation index (CAI). Contrasting these two figures, we can ascertain that there is a relationship between bait/prey interactions and CAI. The relationship is based on proteins with large un-reciprocated in-degree since the right panel shows a dark vertical band. Had unreciprocated out-degree also been associated with CAI then there would be a similar horizontal band reflected across the main diagonal of the matrix.

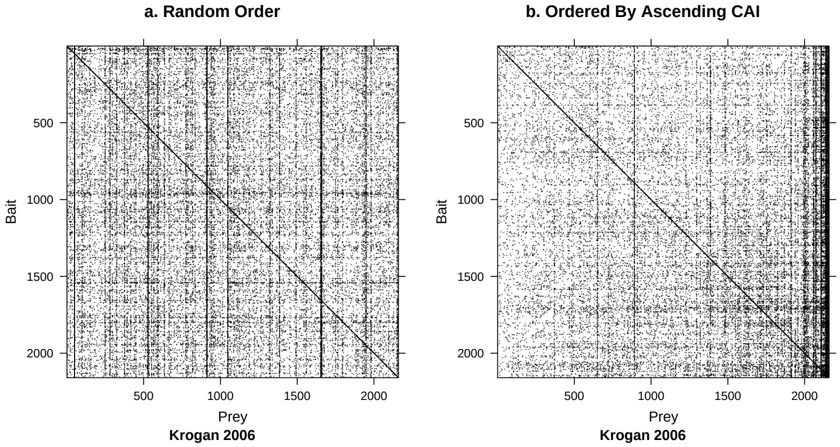

Figure 16: Same adjacency matrix plots for the Krogan et al's [10] data.

both the Pearson and Spearman correlation coefficients between CAI and three sets of abundance data: 1. a general measure of abundance in the yeast cell, 2. the mean measure of abundance of a yeast cell in YEPD medium, and 3. the mean measure of abundance of a yeast cell in a SD medium (Table 2). The Spearman correlation seems to be the more accurate measure, not simply because it is larger, but because the relationship between CAI and abundance is not linear [11] (cf Figures 17 and 18).

	General Abundance	YEPD	SD
Pearson	0.48	0.53	0.55
Spearman	0.54	0.65	0.66

Table 2: This table gives both the Pearson and Spearman correlation between CAI against three distinct protein abundance datasets: 1. General Abundance, 2. Abundance in the YEPD medium, 3. Abundance in the SD medium. An interesting observation is that the highest correlation found is between CAI and protein abundance in the SD medium.

We plotted the values of each protein's CAI value against (log) the three sets of measured abundance data to visualize this association (cf Figures **??**). Those proteins which are likely affected by a systematic bias in the Gavin et al's [4] data are colored red in Figure 17; proteins affected in Krogan et al's [10] are colored red in Figure 17. The most interesting fact is that the measured protein abundances in SD medium have the highest correlation with CAI. This seems to suggest that the reference set of genes used to compute all CAI might be highly expressed under SD medium. In addition, the relationship between the systematic bias with CAI and protein abundance becomes much more apparent (more so with [10]).

4.7 Fisher's Exact Test Across Experiment

Next we wanted to ascertain if the protein subset (S_1) that was affected by a systematic bias in one experiment is related to the protein subset (S_2) affected by a systematic bias of another experiment. There are two ways to generate the subsets S_1 and S_2. The first methods generates these sets in an independent manner; the Binomial model is applied to each experimental dataset generating a subset S_i per experiment i. Then these subsets can be compared by restricting to the set of common VBP of the two experiments. The second method generates S_1 and S_2 by first restricting to the common VBP (denoted by X) of experiment 1 and experiment 2. Then the subset S_1 is generated by applying the Binomial model to the dataset of Experiment 1 restricted only to X, or to use graph theoretic terms, using the node induced subgraph generated by X. S_2 is generated in the same manner with the dataset of experiment 2. We compare the protein subsets S_1 and S_2 using both methods.

To investigate this relationship, we created three 2×2 tables. Only two datasets [4, 10] contained sufficient data points for this analysis. The 2×2 tables were created where the overall universe is restricted to $X = \mathrm{VBP}_{[4]} \cap \mathrm{VBP}_{[10]}$. [4] index the rows; [10], the columns. In the $(2,2)$-entry of each table, we count the number of common proteins affected by a bias in both experiments ($|S_1 \cap S_2|$); in $(1,2)$-entry, we count the number affected in [4] only ($|S_1 \setminus S_2|$); in $(2,1)$, the number in [10] only ($|S_2 \setminus S_1|$); and in $(1,1)$, the number not affected in both ($|S_1^c \cap S_2^c|$). We can create three separate 2×2 tables based on which Binomial test we use:

- Number of proteins identified by the two-sided Binomial test.
- Number of proteins identified by the one-sided Binomial test where in-degree is much larger than out-degree.

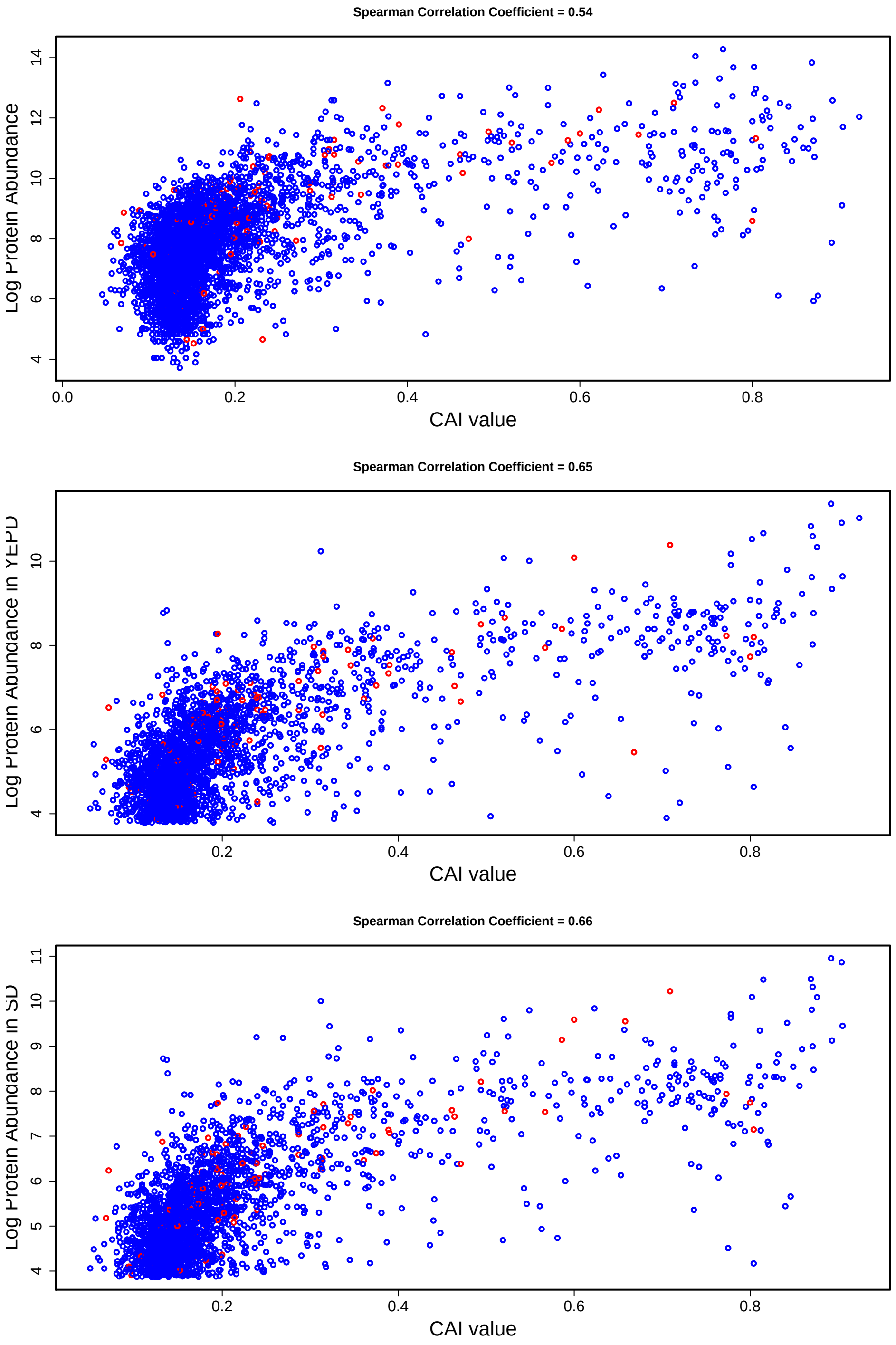

Figure 17: Plots of CAI against log of the three measured abundance datasets. We colored those proteins found to be affected by a systematic bias in the Gavin et al.'s [4] data red and all other proteins blue.

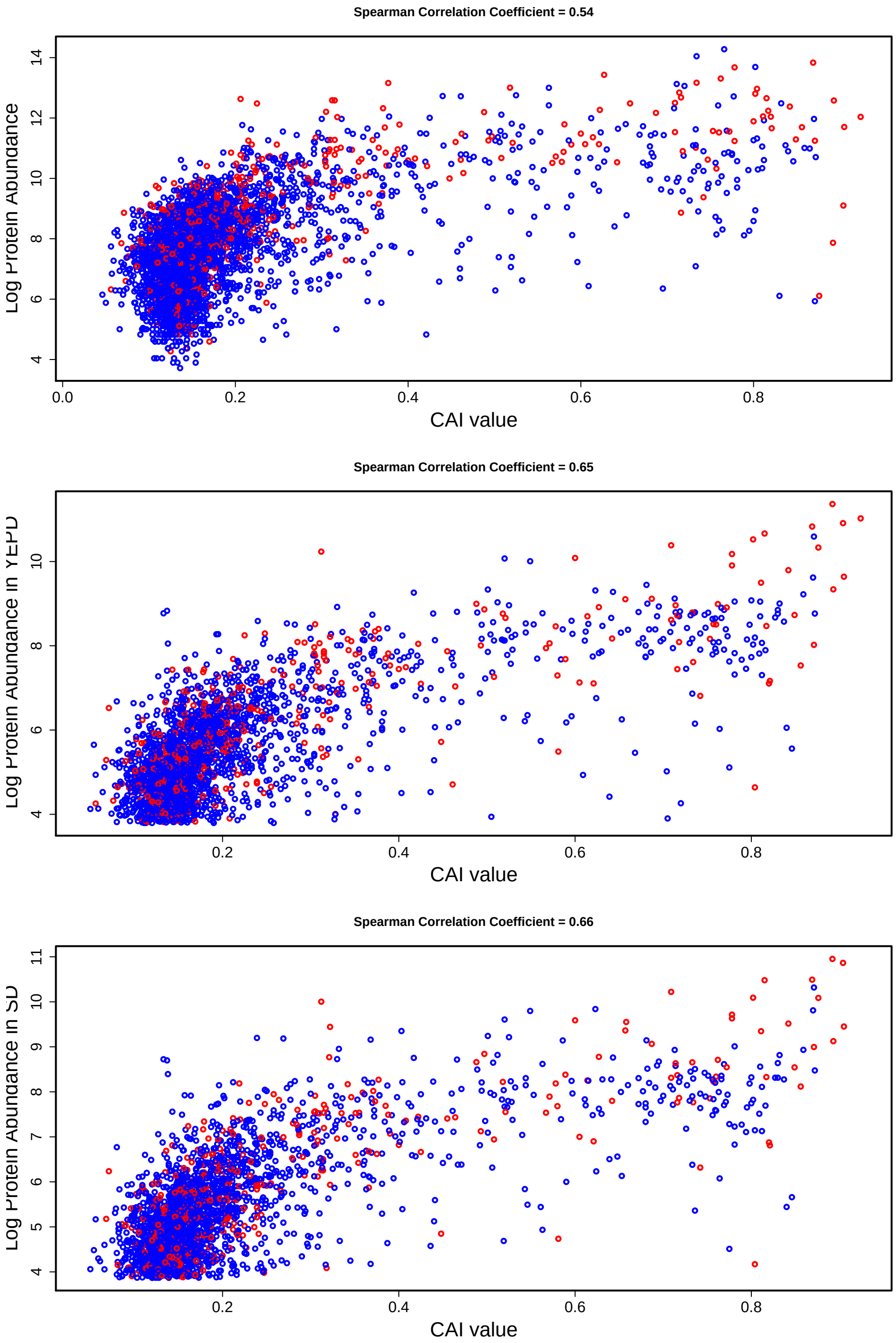

Figure 18: Plots of CAI against log of the three measured abundance datasets. We colored those proteins found to be affected by a systematic bias in the Krogan et al.'s [10] data red and all other proteins blue.

- Number of proteins identified by the one-sided Binomial test where out-degree is much larger than in-degree.

```
> tab2Way

      0   1
  0 391 228
  1  57  57

> tab1WayIN

      0   1
  0 625  63
  1  33  12

> tab1WayOUT

      0   1
  0 483 181
  1  40  29
```

We can use these three tables as the parameters for Fisher's Exact test (again a hypergeometric test), and see the results:

```
> fisher.test(tab2Way)

        Fisher's Exact Test for Count Data

data:  tab2Way
p-value = 0.009
alternative hypothesis: true odds ratio is not equal to 1
95 percent confidence interval:
 1.123257 2.614963
sample estimates:
odds ratio
  1.713527

> fisher.test(tab1WayIN)

        Fisher's Exact Test for Count Data

data:  tab1WayIN
```

```
p-value = 0.0009893
alternative hypothesis: true odds ratio is not equal to 1
95 percent confidence interval:
 1.608501 7.593418
sample estimates:
odds ratio
  3.597664

> fisher.test(tab1WayOUT)

        Fisher's Exact Test for Count Data

data:  tab1WayOUT
p-value = 0.01189
alternative hypothesis: true odds ratio is not equal to 1
95 percent confidence interval:
 1.119609 3.304052
sample estimates:
odds ratio
  1.932813
```

The previous table used each individual dataset's VBP population to generate the sets S_1 and S_2. We then restricted to X to calculate two way tables based on these protein subsets. In the following tables, we first restrict to the node induced subgraph of X for each experiment, and then generate the sets S_1 and S_2. We then create two way tables based on these protein subsets to determine the level of independence.

```
> ta2Way

      0   1
  0 519 115
  1  70  29

> ta1WayIN

      0   1
  0 652  46
  1  27   8

> ta1WayOUT
```

```
     0   1
  0 590  79
  1  53  11

> fisher.test(ta2Way)

        Fisher's Exact Test for Count Data

data:  ta2Way
p-value = 0.01379
alternative hypothesis: true odds ratio is not equal to 1
95 percent confidence interval:
 1.114654 3.073070
sample estimates:
odds ratio
  1.867942

> fisher.test(ta1WayIN)

        Fisher's Exact Test for Count Data

data:  ta1WayIN
p-value = 0.002547
alternative hypothesis: true odds ratio is not equal to 1
95 percent confidence interval:
  1.554209 10.167306
sample estimates:
odds ratio
  4.185891

> fisher.test(ta1WayOUT)

        Fisher's Exact Test for Count Data

data:  ta1WayOUT
p-value = 0.2297
alternative hypothesis: true odds ratio is not equal to 1
95 percent confidence interval:
 0.699581 3.161824
sample estimates:
odds ratio
  1.548972
```

Because we are looking for reproducibility across experiments, the two-way tables `tab2Way` and (ta2Way) as well as the results from their corresponding Fisher's exact test are not particularly relevant. Indeed, we should only focus on the one-sided Binomial tests and see if we such artifacts are reproducible across experiments. For those proteins which in-degree dominates out-degree, i.e. proteins tested in `tab1WayIN` and `ta1WayIN`, we see an exceptionally small p-value for the former and a reasonably significant p-value for the latter, and so we should probably reject the null hypothesis that these S_1 is independent of S_2. For those proteins which out-degree dominates in-degree, `tab1WayOUT` and `ta1WayOUT`, we see a less significant p-value in the former but an incredibly small p-value in the latter, and so again we should reject independent null hypothesis. For the sake of completeness we present all the two way tables and note that all the tests show relatively small p-values as well as substantial odds ratios.

4.8 Unreciprocated Degree Statistics

Our Binomial model allows us to determine proteins that might be subject to a systematic bias of the experiment where each statistical test is conducted on a per protein level. In addition to these series of statistical test, we can describe experiment-wide artifacts by using the same binomial model for each protein.

For each protein ρ with directed degree (i_ρ, o_ρ) and $n_\rho = i_\rho + o_\rho$, we can standardize the in-degree and compute the corresponding *z-score* for each protein:

$$z_\rho = \frac{\frac{1}{2}\, n_\rho - o_\rho}{\sqrt{\left(\frac{1}{2}\right)^2\, n_\rho}} \tag{9}$$

$$= \frac{i_\rho - o_\rho}{\sqrt{i_\rho + o_\rho}} \tag{10}$$

After calculating the z-score for each protein within an experiment, we were able to estimate the distributions within each dataset. Using the R `hist` and `density` functions, we were able to render the histograms for eleven of the datasets and the smooth density distribution for the three largest datasets.

Histograms are plotted to determine the standard out-degree distributions (Figure 19). We can see that for datasets such as [1, 13, 5], there are relatively few data points which yield little statistical power. Overall, Only

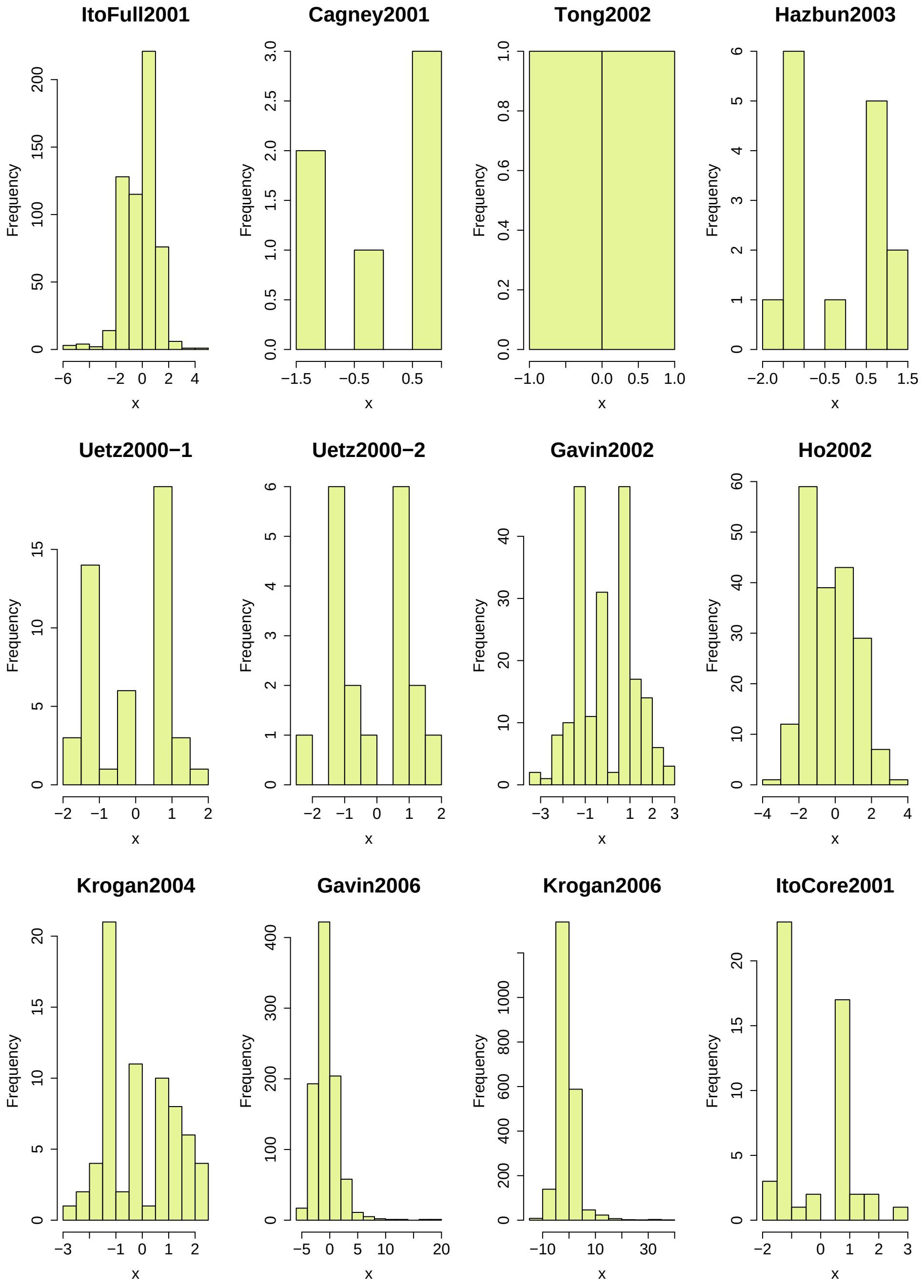

Figure 19: Histograms of the out-degree z-scores.

the [7, 4, 10] plots showed distinct and approximately unimodal distributions.

Thesethree plots have either a strong positive or negative concentration of data points in the distributions based on their densities (Figure 20). We have discussed the possible reasons for these concentrations in the main article.

In addition to the density plots, we wanted to compare z-scores across experimental datasets. Currently, there are only two datasets [4, 10] for which this comparison is insightful. Again we can calculate the z-score for a protein in two ways: either within the original VBP population of each individual dataset or by first restricting to the common VBP population X in each dataset. We have computed the z-score in both manners. Once the z-scores have been computed, we can plot the scores of [4] against [10] restricted to protein set X (See Figure **??**). From the plots, we were also able to generate correlation coefficients between the z-scores [4] and the z-scores from [10] restricted to X.

	Pearson	Kendall	Spearman
Calculations from the Original Data	0.18	0.042	0.062
Calculations Restricted to X	0.17	0.046	0.071

Table 3: This table gives both the Pearson, Kendall, and Spearman correlation between the z-scores from the data of Gavin et al. and the z-scores from the data of Krogan et al. (both 2006). The correlation is computed with the restriction to those VBP in both datasets.

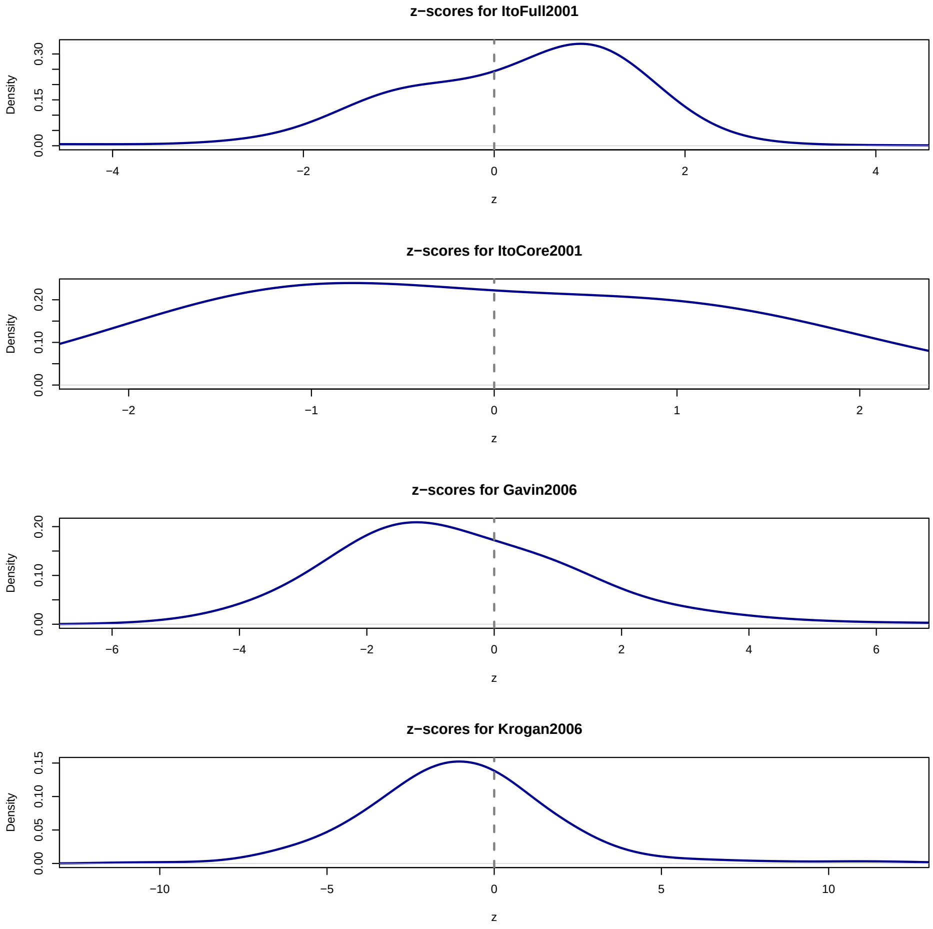

Figure 20: Density plots for the three largest bait/prey datasets with the zero-line.

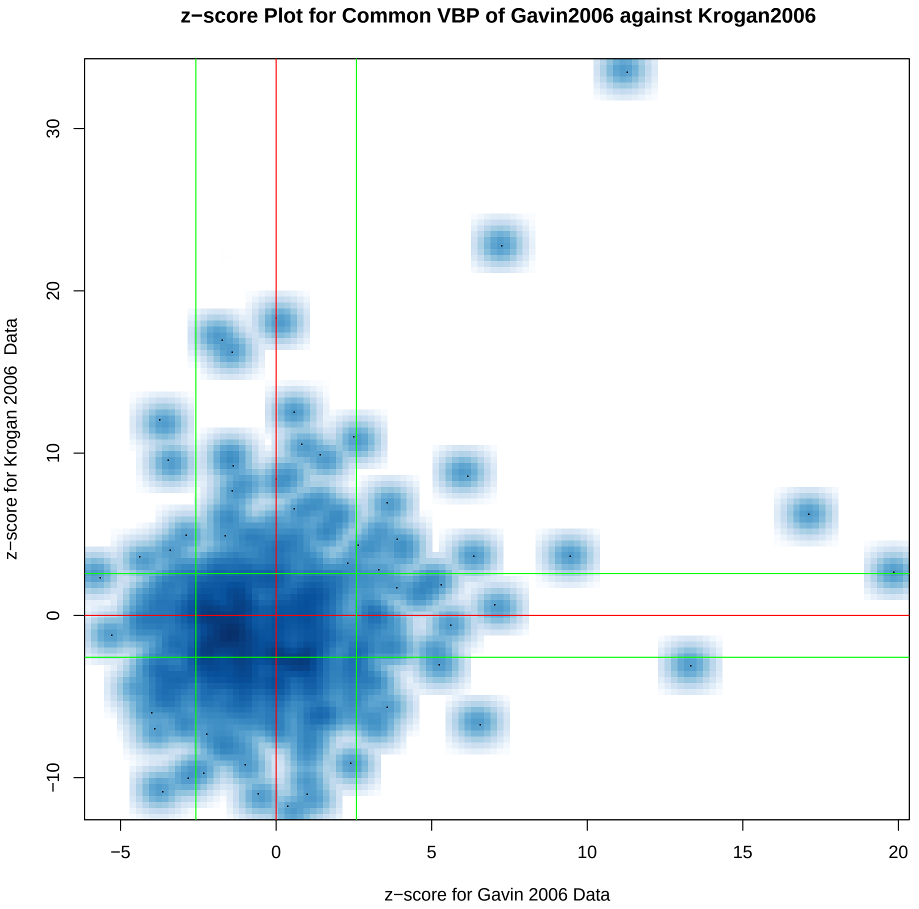

Figure 21: This figure shows the z-score for the common VBP of Gavin et al.'s [4] data against Krogan et al.'s [10] data. We can see from the figure that the highest concentration of z-scores is off the origin. There is also evidence that proteins which have relatively large z-scores in absolute value in one experiment will also have relatively large z-scores in the other.

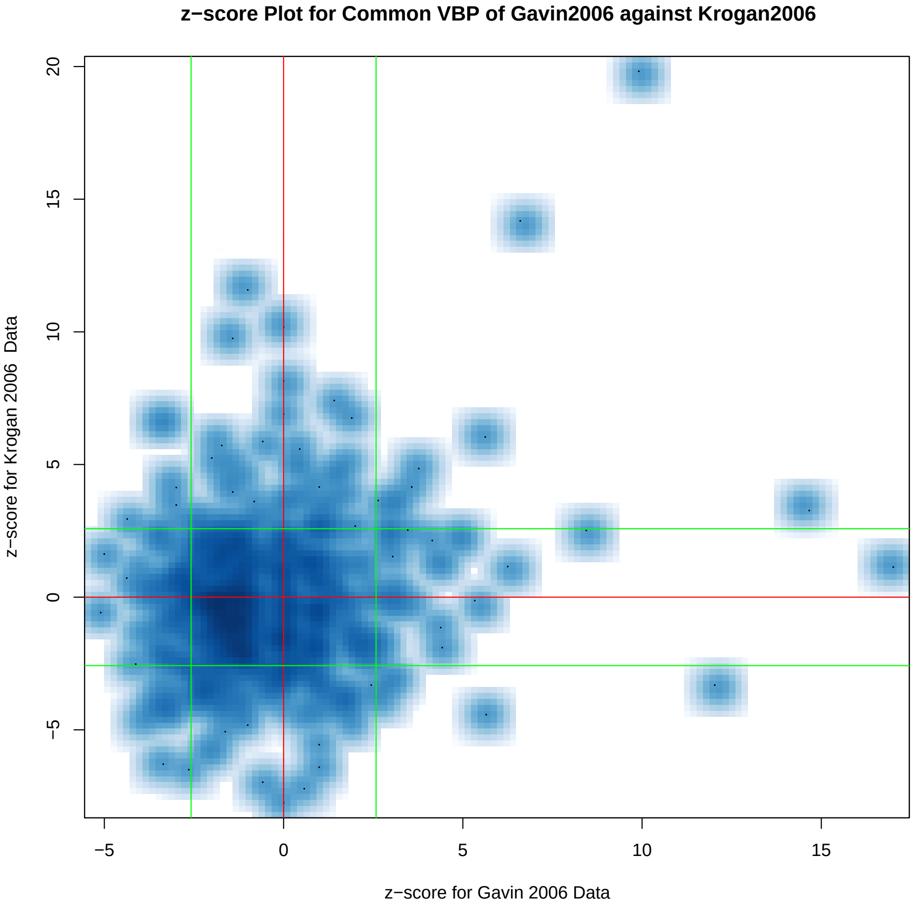

Figure 22: This figure shows the z-score for the common VBP of Gavin et al.'s [4] data against Krogan et al.'s [10] data when we restrict first and then find the systematic proteins.

5 Stochastic Error Analysis: Estimation of p_{fp} and p_{fn} by the method of moments

5.1 Derivation

$\binom{N}{2}$		The total number of possible interactions (excluding homomers)
n		the true number of interactions
m	$= \binom{N}{2} - n$	the true number of non-interactions
X_1		observed number of reciprocated edges
X_2		observed number of non-edges
X_3	$= n + m - X_1 - X_2$	observed number of unreciprocated eges

we have

$$
\begin{aligned}
E[X_1] &= n\,(1-p_{\mathrm{fn}})^2 + m\,p_{\mathrm{fp}}^2 && (11)\\
E[X_2] &= n\,p_{\mathrm{fn}}^2 + m\,(1-p_{\mathrm{fp}})^2 && (12)\\
E[X_3] &= 2n\,p_{\mathrm{fn}}(1-p_{\mathrm{fn}}) + 2m\,p_{\mathrm{fp}}(1-p_{\mathrm{fp}}) && (13)
\end{aligned}
$$

Only two of the three equations (11)–(13) are independent, any two of them imply the third. Our goal is to estimate p_{fp}, p_{fn}. We can replace the expectation values on the left side of Equations (11)–(13) by the observed sample values x_1, x_2, x_3. Since we do not know n, the above system of two independent equations for three variables defines a one-dimensional solution manifold.

We will parameterize that manifold by n ($0 \le n \le \binom{N}{2}$) in $(p_{\mathrm{fp}}, p_{\mathrm{fn}})$-space. Relevant solutions are those for which $0 \le p_{\mathrm{fp}}, p_{\mathrm{fn}} \le 1$.

Consider that n is given. Let us solve Equations (11)–(13) for p_{fp} and p_{fn}. First, subtracting (14) = (11) − (12), we have

$$
\begin{aligned}
x_1 - x_2 &= n\,(1-2p_{\mathrm{fn}}) - m\,(1-2p_{\mathrm{fp}}) && (14)\\
\Leftrightarrow \quad p_{\mathrm{fn}} &= \frac{1}{2n}\left((x_2-m)-(x_1-n)+2m\,p_{\mathrm{fp}}\right) &&\\
p_{\mathrm{fn}} &= \frac{1}{2n}\left(\Delta + 2m\,p_{\mathrm{fp}}\right), && (15)
\end{aligned}
$$

where we have defined $\Delta := (x_2 - m) - (x_1 - n)$ for convenience. We can plug this expression for p_{fn} into Equation (12) and obtain

$$
\underbrace{(n+m)}_{=:a} p_{\mathrm{fp}}^2 + \underbrace{(\Delta - 2n)}_{=:b} p_{\mathrm{fp}} + \underbrace{n + \frac{\Delta^2}{4m} - \frac{n}{m} x_2}_{=:c} = 0. \qquad (16)
$$

The equation $a(p_{\text{fp}})^2 + b(p_{\text{fp}}) + c = 0$ is solved by

$$(p_{\text{fp}})_{1,2} = \frac{-b \pm \sqrt{b^2 - 4ac}}{2a}. \quad (17)$$

Hence, the problem is solved: for data N, x_1, x_2 (from these, x_3 is implied) and for all possible (unknown) $n = 0, 1, \ldots, \binom{N}{2}$ we can calculate p_{fp} via Equation (17), then p_{fn} via Equation (15). Only some of the theoretically possible values of n will lead to admissible solutions for p_{fp} and p_{fn}. This is exemplified in the following section.

5.2 Computation

The function `estErrProbMethodOfMoments` in the *ppiStats* package implements the computations described in the previous section.

5.2.1 Test on simulated data

First, we want to gain confidence in the algorithm and its software implementation by looking at simulated data. The function `sim` calculates $E[X_1]$, $E[X_2]$ and $E[X_3]$ according to Equations (11)–(13). Its arguments `pfp` (p_{fp}), `pfn` (p_{fn}), `ntot` (N) need to be scalars, `n1` (n) can be a vector.

```
> sim = function(n1, pfp, pfn, ntot) {
+     nEdges = ntot * (ntot - 1)/2
+     stopifnot(length(pfp) == 1, length(pfn) == 1,
+         length(ntot) == 1, all(n1 <= nEdges))
+     n2 = nEdges - n1
+     cbind(x1 = n1 * (1 - pfn)^2 + n2 * pfp^2, x2 = n1 *
+         pfn^2 + n2 * (1 - pfp)^2, x3 = 2 * n1 *
+         pfn * (1 - pfn) + 2 * n2 * pfp * (1 - pfp))
+ }
```

We consider the following example.

```
> ntot = 2000
> n1 = 12000
> pfp = 0.001
> pfn = 0.1
> s = sim(n1 = n1, pfp = pfp, pfn = pfn, ntot = ntot)
> s
```

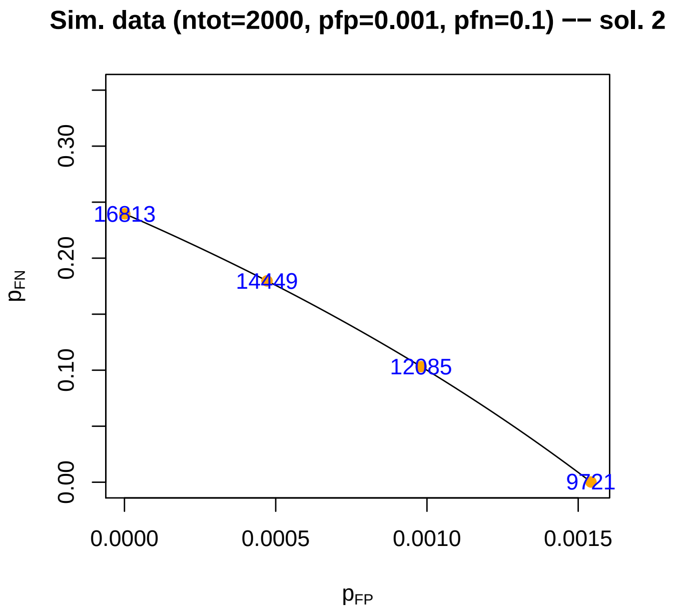

Figure 23: The solution manifold. The numbers in blue are the corresponding values of n_1, the corresponding (unknown) true number of interactions.

```
           x1      x2       x3
[1,] 9721.987 1983148 6130.026
```

Now pretend we found data with x_1 =9722, x_2 =1983148 and x_3 =6130, and we try out many possible values of `n1`. The plot is shown in Figure 23.

```
> n1try = seq(1, 3 * n1, by = 12)
> r = estErrProbMethodOfMoments(n1try, nrec = round(s[1,
+     "x1"]), nunr = round(s[1, "x3"]), ntot = ntot)
> plotpfppfn(r, main = sprintf("Sim. data (ntot=%d, pfp=%g, pfn=%g)",
+     ntot, pfp, pfn), qmax = 0.35)
```

We can also verify that if we provide the correct value `n1`=12000, we recover the original probabilities:

```
> res = estErrProbMethodOfMoments(n1, nrec = s[1,
+     "x1"], nunr = s[1, "x3"], ntot = ntot)
```

```
      nint       pfp1     pfn1  pfp2 pfn2
[1,] 12000 0.01179340 1.887207 0.001  0.1
```

5.3 Application to the PPI datasets

We now take the experimental datasets obtained from [7, 14, 1, 5, 13, 3, 6, 9, 4, 10] to obtain the 1-dimensional manifolds.

We plot each dataset individually to ascertain the range for p_{fp} and for p_{fn}. The result of this is plotted in Figure 24 for the unfiltered data and in Figure 25 for the filtered data.

Next we wanted to superimpose all experiments of the same type (i.e. the same system was used to determine the interactions) so that we can compare the solutions curves across experiments. We do this first on the set of VBP for each dataset, and these are rendered in the top two plots. After, we filtered out the proteins likely to be affected by a systematic bias and recalibrated the solution curves. These are rendered in the bottom two plots. The result of this is plotted in Figure 26.

6 Stochastic Error Analysis: Estimation of Unreciprocated and Reciprocated FP/FN Errors within the Measured Data

Using the Multinomial error model, we can estimate the expected number unreciprocated and reciprocated number of false positive as well as false negative interactions. For these estimates, we use only the filtered set of data on the protein interactions (let N be the number of proteins in the filtered set). We begin by estimating the FP errors. To calculate the expected number of FP observations, we need estimates for p_{fp} as well as m (since FP is a property on I^c). To obtain these estimates, we assume that $p_{\text{fn}} = 0$ so that all errors will be strictly FP. This makes the estimate for p_{fp} maximal, and the number of expected FP observations that we calculate will all be for the worst case scenairo. Using the Multinomial error model, we generate p_{fp}'s for all the datasets. We can also generate the value for m by assuming that $p_{\text{fn}} = 0$, but for the sake of convenience, we will approximate m by $\binom{N}{2}$. Unreciprocated FP errors can be estimated by $2p_{\text{fp}}(1-p_{\text{fp}})m$; reciprocated, by $p_{\text{fp}}^2 m$. Lastly we provide the number of observed unreciprcated and reciprocated interactions to serve as a reference. These estimates can be found in Table 4

Similarly, we also use the Multinomial error model to estimate the expected number of unreciprocated and reciprocated FN observations. We estimate p_{fn} and n by assuming that $p_{\text{fp}} = 0$, and so, again we presume that all errors are strictly FN. This makes p_{fn} maximal, and this also makes these estimates for FN errors in the worst case scenario. Similar to the FP estimates, unreciprocated FN errors are estimated by $2p_{\text{fn}}(1-p_{\text{fn}})n$; reciprocated by $p_{\text{fn}}^2 n$. We also provide the number of observed unreciprcated and reciprocated non-interacting protein pairs found within the datasets. These estimates can be found in Table 5

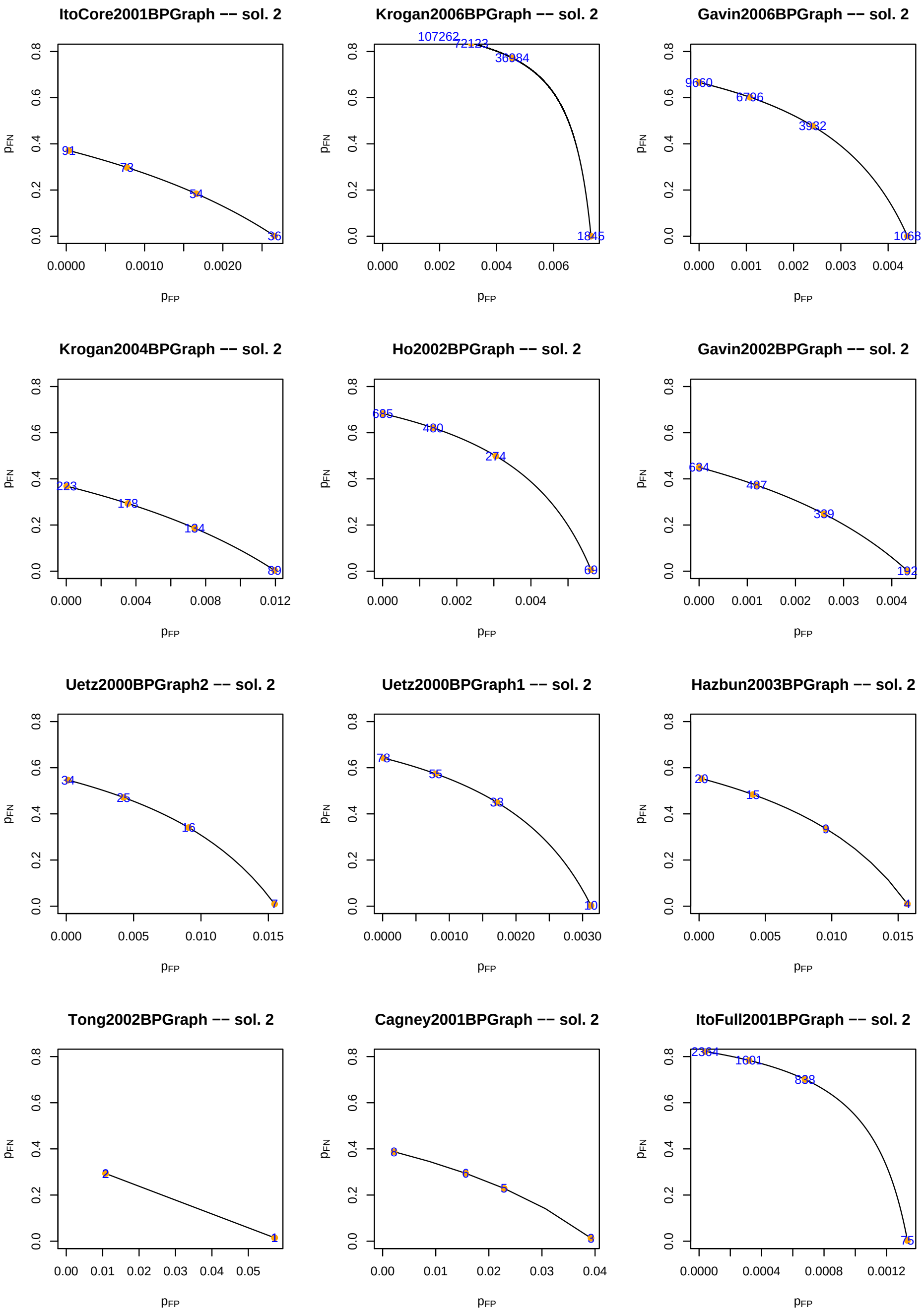

Figure 24: The solution manifolds (one plot per dataset, unfiltered data).

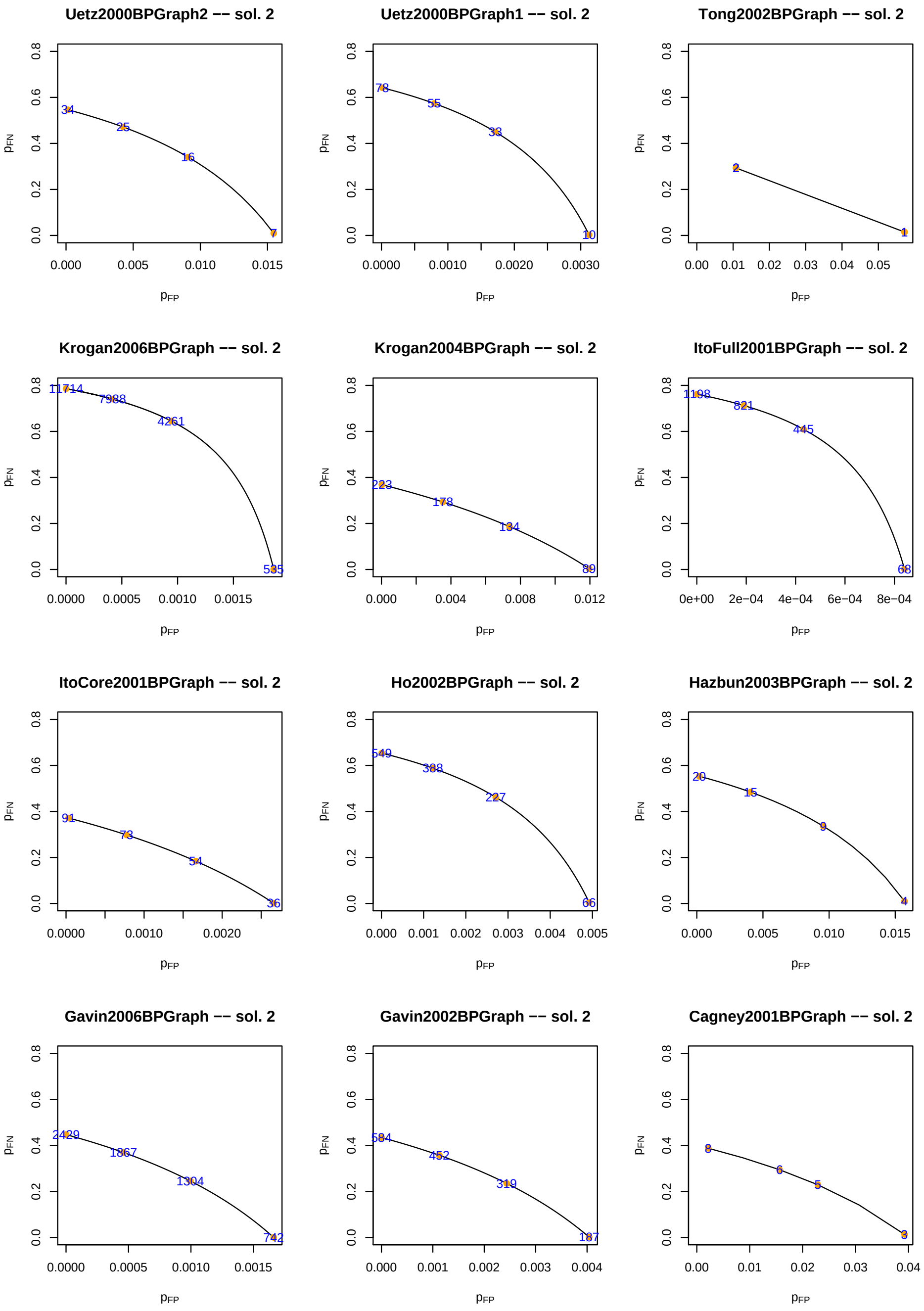

Figure 25: The solution manifolds (one plot per dataset, filtered data)

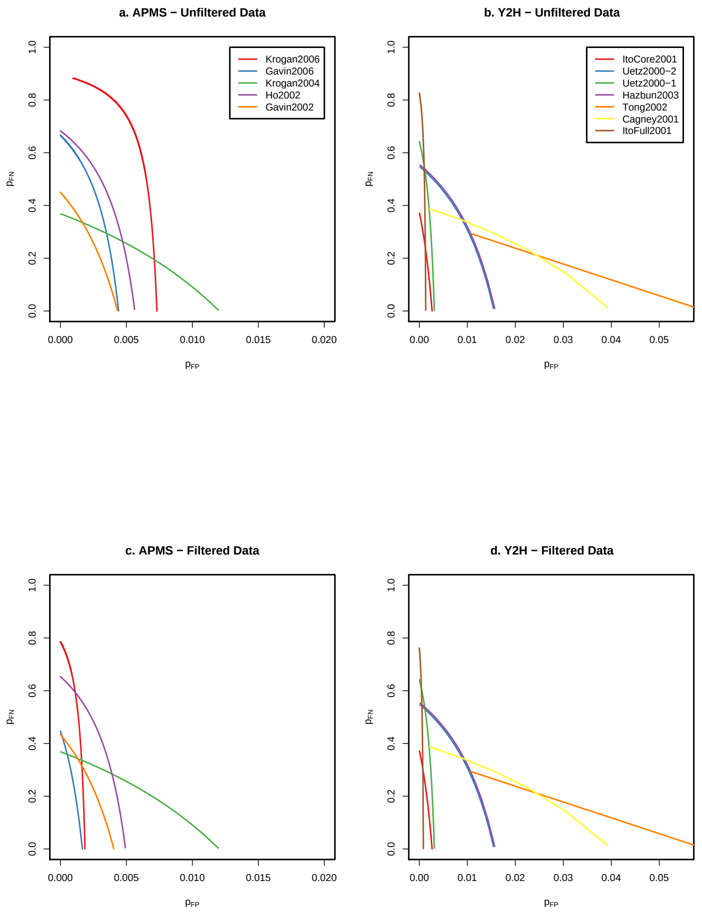

Figure 26: These figures detail the error statistics for each of the datasets. Plot *a* generates the 1-dimensional solution curves for $(p_{\text{fp}}, p_{\text{fn}})$ parametrized by n for the AP-MS datasets. Plot *b* generates similar 1-dimensional curves but for the Y2H datasets. Plots *c* and *d* recalculates these solution curves for the AP-MS and Y2H respectively. These recalculations are done by setting aside those interactions which appear to be affected by a systeamtic bias of the experimental assay. Having set aside those interactions, the range for p_{fp} is substantially constrained for the solution curves characterizing [4, 10] implying that systematic errors may potentially have large effects on p_{fp}.

	N	m	pfp	E[Z]	E[Z2]	z	z2
ItoFull2001	720	258840	0.0008	414	0.17	435	68
Cagney2001	11	55	0.04	4	0.09	4	3
Tong2002	5	10	0.07	1	0.05	1	1
Hazbun2003	26	325	0.016	10	0.08	10	4
Uetz2000-1	108	5778	0.003	35	0.05	36	10
Uetz2000-2	34	561	0.015	17	0.13	17	7
Gavin2002	268	35778	0.004	285	0.57	287	187
Ho2002	226	25425	0.005	253	0.64	249	66
Krogan2004	95	4465	0.012	106	0.64	104	89
Gavin2006	852	362526	0.0017	1230	1.1	1201	743
Krogan2006	1458	1062153	0.0019	4029	3.8	3945	538
ItoCore2001	128	8128	0.0025	41	0.05	43	36

Table 4: Estimates of FP unreciprocated and reciprocated errors via the Multinomial error Model. Z is the random variable associated with unreciprocated FP errors; Z2 corresponds with reciprocated FP errors. z and z2 denote the observed number of unreciprocated and reciprocated interactions found in the data.

7 Cross Data Integration and Analysis

We have shown that protein interaction analysis can be measured by three quality metrics: 1. coverage, 2. proteins that might be affected by systematic bias due to the experiment, and 3. general stochastic variation. It is necessary to consider each of these three metrics if one would like to begin cross experimental analysis.

As an example we show how coverage (and sampling) is fundamental for inter-experimental analysis.

The possible pitfalls of naive comparisons between two experimental datasets are depicted in Figure 27. The interactions in the intersection of the rectangles (red) were tested by both; the interactions in the green and purple areas were tested by one experiment but not the other; and the interactions in the light grey areas were tested by neither experiment. A data analysis that does not keep track of these different coverage characteristics risks being misleading. Therefore, coverage must be taken into consideration when integrating and comparing multiple datasets. Additionally, discrepancies will arise due to the set conditions of each experiment, and these discrepancies should be isolated from the variability across the

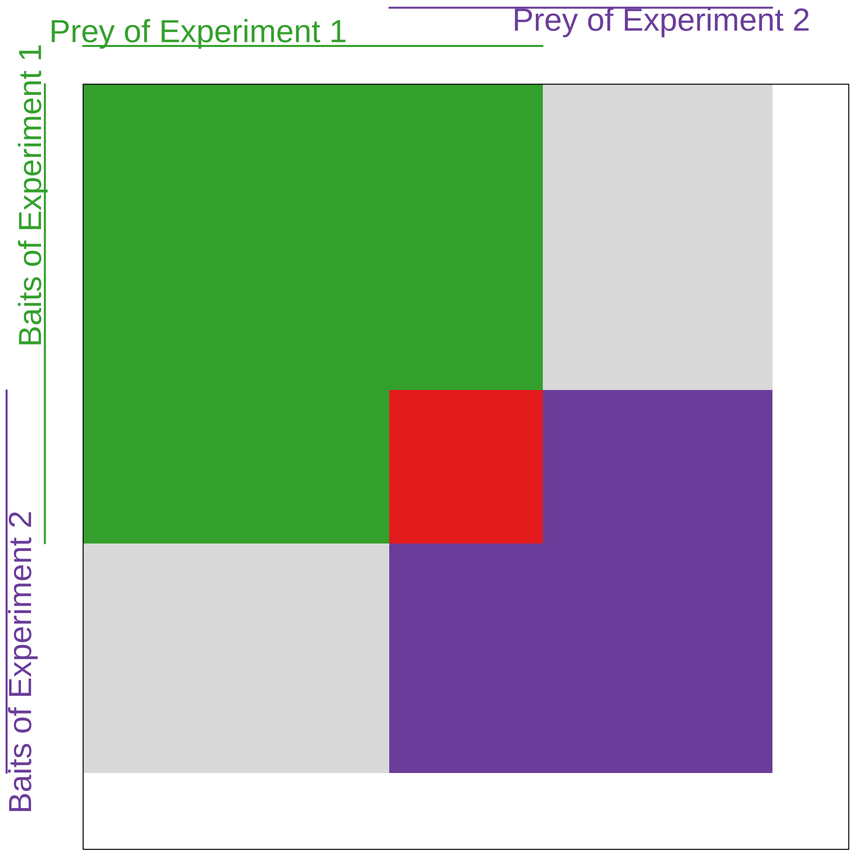

Figure 27: A schematic representation of the interactome coverage of two protein interaction experiments. The adjacency matrix of the complete interactome is represented by the large square. Experiment 1 covers a certain set of proteins as baits (rows covered by the green vertical line) and as prey (columns covered by the green horizontal line). The tested interactions for Experiment 1 are contained within the green rectangle. Similarly, Experiment 2 covers another set of proteins and tests for a set of interactions contained in the purple rectangle. The intersection of the rectangles, the red area, are the bait to prey interactions tested by both experiments, and the union are the interactions tested by at least one of the experiments. Note that the interactions in the light gray area were tested by *neither* experiment either because there are missing tested prey (upper right corner) or missing tested baits (lower left corner). The interactions in the white region are also tested by *neither* experiment because both the baits and the prey were not tested.

	N	n	pfn	E[W]	E[W2]	w	w2
ItoFull2001	720	1200	0.76	438	693	435	259132
Cagney2001	11	8	0.39	4	1	4	57
Tong2002	5	2	0.30	1	0	1	11
Hazbun2003	26	20	0.55	10	6	10	334
Uetz2000-1	108	78	0.65	35	33	36	5822
Uetz2000-2	34	34	0.55	17	10	17	571
Gavin2002	268	584	0.44	288	113	287	35725
Ho2002	226	649	0.68	282	300	249	25472
Krogan2004	95	223	0.37	104	31	104	4423
Gavin2006	852	2429	0.44	1197	470	1201	362209
Krogan2006	1458	11744	0.80	3758	7516	3945	1062344
ItoCore2001	128	100	0.38	47	14	43	8156

Table 5: Estimates of FN unreciprocated and reciprocated errors via the Multinomial error Model. W is the random variable associated with unreciprocated FP errors; W2 corresponds with reciprocated FP errors. w and w2 denote the observed number of unreciprocated and reciprocated interactions found in the data.

experiments so the error rates have a more meaningful interpretation. Ultimately, there are still many more steps needed to integrate datasets, and we discuss a few necessary components.

References

[1] G Cagney et al. Two-hybrid analysis of the saccharomyces cerevisiae 26s proteasome. *Physiological Genomics*, 7:27–34, 2001.

[2] S. Falcon and R. Gentleman. Using gostats to test gene lists for go term association. *Bioinformatics*, 2:257–258, 2006.

[3] Anne-Claude Gavin et al. Functional organization of the yeast proteome by systematic analysis of protein complexes. *Nature*, 415:141–147, 2002.

[4] Anne-Claude Gavin et al. Proteome survey reveals modularity of the yeast cell machinery. *Nature*, 440:631–636, 2006.

[5] T Hazbun et al. Assigning function to yeast proteins by integration of technologies. *Molecular Cell*, 6:1353–1365, 2003.

	VB	CB	TB	VB/TB	VP	VBP	VBP/BP	VP/VB	TI	TI/VB
ItoFull2001	1522		6604	0.23	2493	773	0.51	1.64	4524	2.97
Cagney2001	19		31	0.61	40	11	0.58	2.11	54	2.84
Tong2002	20		22	0.91	59	5	0.25	2.95	115	5.75
Hazbun2003	66		100	0.66	1940	28	0.42	29.39	2524	38.24
Zhao2005	1		1	1.00	90	0	0.00	90.00	90	90.00
Uetz2000-1	508		6604	0.08	630	142	0.28	1.24	952	1.87
Uetz2000-2	139		192	0.72	400	36	0.26	2.88	524	3.77
Gavin2002	455	600	725	0.63	1178	271	0.60	2.59	3418	7.51
Ho2002	493	589	1739	0.28	1316	231	0.47	2.67	3687	7.48
Krogan2004	153	165	165	0.93	483	151	0.99	3.16	1132	7.40
Gavin2006	1752	1993	6466	0.27	1790	991	0.57	1.02	19105	10.90
Krogan2006	2264	2357	4562	0.50	5323	2226	0.98	2.35	63360	27.99
ItoCore2001	455		6604	0.07	504	164	0.36	1.11	839	1.84

Table 6: A general overview of seven Y2H and five AP-MS experiments: VB - the number of viable baits; CB - the number of cloned (hybridized) baits if available; TB - the total number of baits; VB/TB; VP - the number of viable prey; VBP - the number of proteins observed as both bait and prey; VBP/VB; VP/VB, TI - the total number of interactions observed; TI/VB.

[6] Y. Ho et al. Systematic identification of protein complexes in *Saccharomyces cerevisiae* by mass spectrometry. *Nature*, 415:180–183, 2002.

[7] T. Ito et al. A comprehensive two-hybrid analysis to explore the yeast protein interactome. *Proc. Natl. Acad. Sci. of the U.S.A.*, 98:4569–4574, 2001.

[8] J Kelsey et al. Methods in observational epidemiology. In *Monographs in Epidemiology and Biostatistics*. Oxford University Press, New York, 1996.

[9] N. J. Krogan et al. High-definition macromolecular composition of yeast rna-processing complexes. *Molecular Cell*, 13:225–239, 2004.

[10] N. J. Krogan et al. Global landscape of protein complexes in the yeast saccharomyces cerevisiae. *Nature*, 440:637–643, 2006.

[11] P M Sharp and W H Li. The codon Adaptation Index–a measure of directional synonymous codon usage bias, and its potential applications. *Nucleic Acid Res*, 15:1281–1295, 1987.

	ItoFull2001	Cagney2001	Tong2002	Hazbun2003	Zhao2005	Uetz2000-1	Uetz2[illegible]
ItoFull2001		9	7	24	1	224	
Cagney2001	28		0	0	0	7	
Tong2002	34	0		0	0	4	
Hazbun2003	855	14	25		0	15	
Zhao2005	43	1	2	38		0	
Uetz2000-1	389	14	22	272	15		
Uetz2000-2	199	9	26	204	13	108	
ItoCore2001	498	10	12	211	17	205	

Table 7: This table shows two distinct statistics on the pair-wise comparison of the data sets. The values above the diagonal give the number of common viable baits, the values below the diagonal give the number of common viable prey.

	Gavin2002	Ho2002	Krogan2004	Gavin2006	Krogan2006
Gavin2002		82	51	446	336
Ho2002	517		25	223	286
Krogan2004	300	246		122	151
Gavin2006	1148	721	373		1131
Krogan2006	1150	1277	478	1756	

Table 8: This table shows two distinct statistics on the pair-wise comparison of the data sets. The values above the diagonal give the number of common viable baits, the values below the diagonal give the number of common viable prey.

[12] John Storey. A direct approach to false discovery rates. *Journal of the Royal Statistical Society, Series B*, 64:479–498, 2002.

[13] Amy Hin Yan Tong et al. A combined experimental and computational strategy to define protein interaction networks for peptide recognition modules. *Science*, pages 321–324, 2002.

[14] Peter Uetz et al. A comprehensive analysis of protein-protein interactions in *Saccharomyces cerevisiae*. *Nature*, 403:623–627, 2000.

[15] R. Zhao et al. Navigating the chaperone network: An integrative map of physical and genetic interactions mediated by the Hsp90 chaperone.

Cell, 120:715–727, 2005.
