# Supplementary material for: Coverage and error models of protein-protein interaction data by directed graph analysis
Source: Genome Biol. 2007 Sep 10;8(9):R186. doi: 10.1186/gb-2007-8-9-r186 (PMC2375024; doi:10.1186/gb-2007-8-9-r186)
Supplement: Additional data file 2 — Presented is the Bioconductor package ppiStats (version 1.3.5 of 22 June 2007) in 'source' format. ppiStats contains the novel methods developed in this paper. [file gb-2007-8-9-r186-S2.gz › ppiStats/inst/doc/fig-pfppfnFiltSep.pdf]

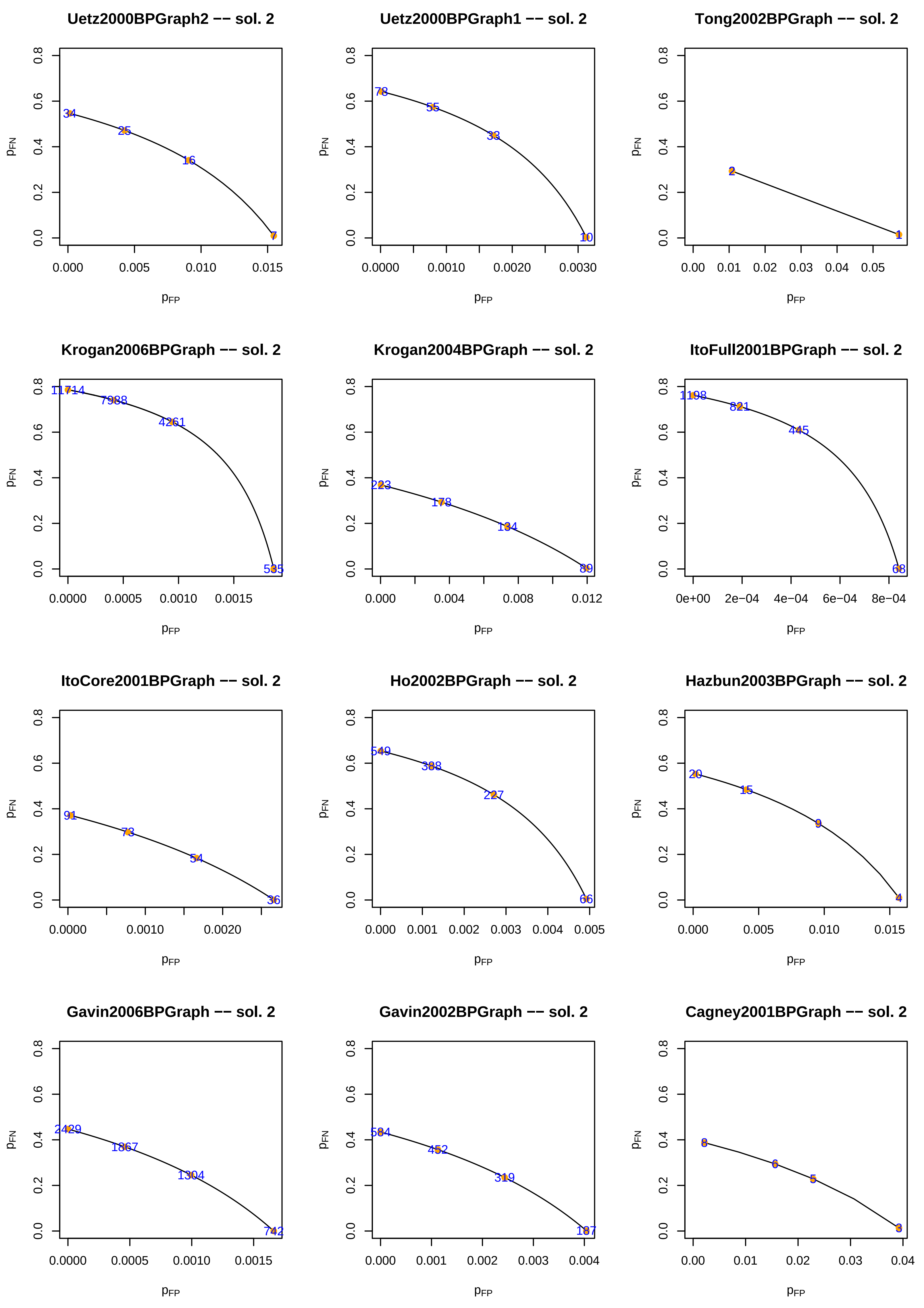

Uetz2000BPGraph2 -- sol. 2
34
25
16
7
Uetz2000BPGraph1 -- sol. 2
78
55
33
10
Tong2002BPGraph -- sol. 2
2
1
Krogan2006BPGraph -- sol. 2
11714
7988
4261
535
Krogan2004BPGraph -- sol. 2
223
178
134
89
ItoFull2001BPGraph -- sol. 2
1198
821
445
68
ItoCore2001BPGraph -- sol. 2
91
73
54
36
Ho2002BPGraph -- sol. 2
549
388
227
66
Hazbun2003BPGraph -- sol. 2
20
15
9
4
Gavin2006BPGraph -- sol. 2
2429
1867
1304
742
Gavin2002BPGraph -- sol. 2
584
452
319
187
Cagney2001BPGraph -- sol. 2
8
6
5
3
p_{FN}
p_{FP}
