# Supplementary material for: Coverage and error models of protein-protein interaction data by directed graph analysis
Source: Genome Biol. 2007 Sep 10;8(9):R186. doi: 10.1186/gb-2007-8-9-r186 (PMC2375024; doi:10.1186/gb-2007-8-9-r186)
Supplement: Additional data file 2 — Presented is the Bioconductor package ppiStats (version 1.3.5 of 22 June 2007) in 'source' format. ppiStats contains the novel methods developed in this paper. [file gb-2007-8-9-r186-S2.gz › ppiStats/inst/doc/fig-pfppfnUnfiltSep.pdf]

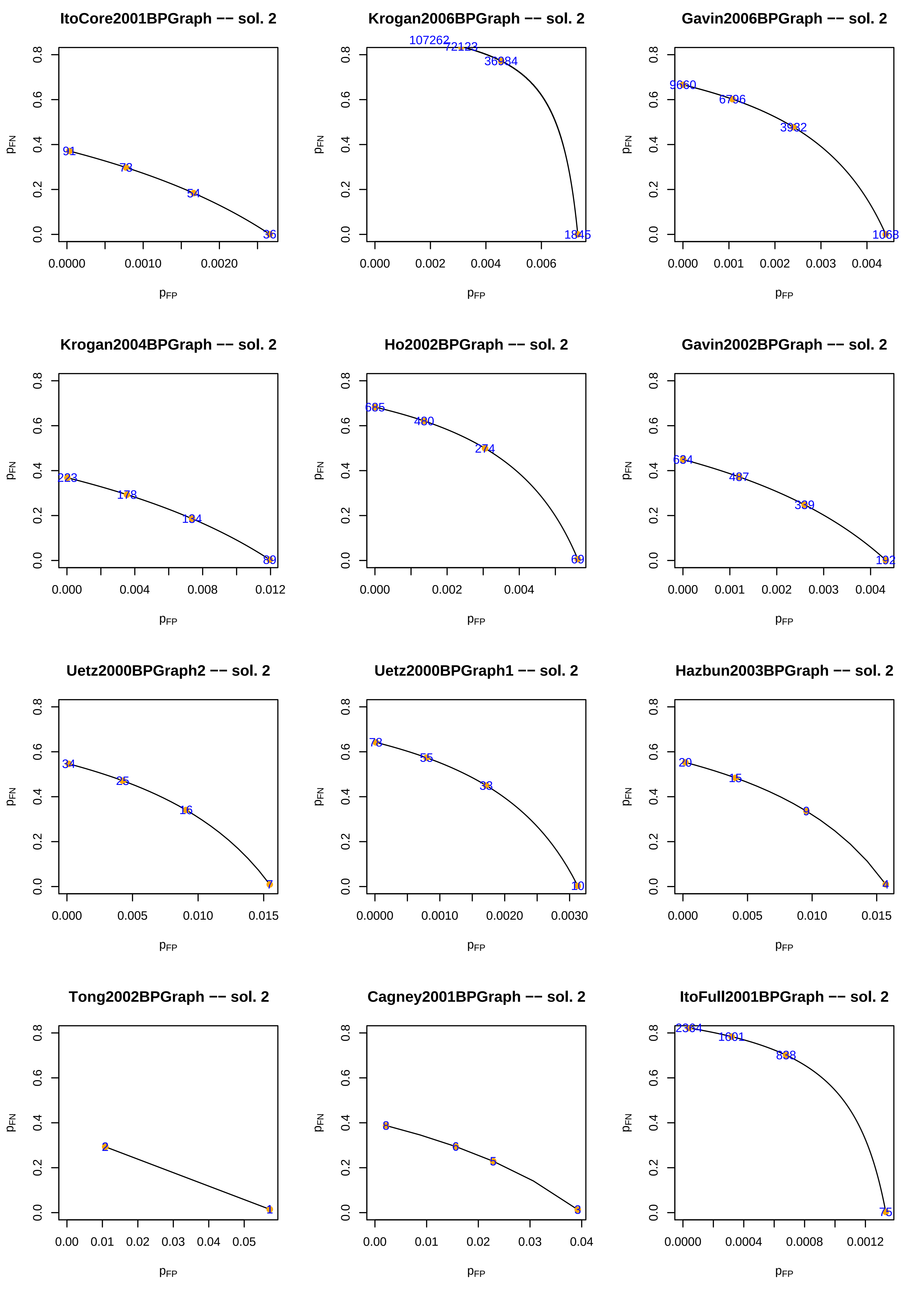
ItoCore2001BPGraph –– sol. 2
91
73
54
36
Krogan2006BPGraph –– sol. 2
107262
72123
36984
1845
Gavin2006BPGraph –– sol. 2
9660
6796
3932
1068
Krogan2004BPGraph –– sol. 2
223
178
134
89
Ho2002BPGraph –– sol. 2
685
480
274
69
Gavin2002BPGraph –– sol. 2
634
487
339
192
Uetz2000BPGraph2 –– sol. 2
34
25
16
7
Uetz2000BPGraph1 –– sol. 2
78
55
33
10
Hazbun2003BPGraph –– sol. 2
20
15
9
4
Tong2002BPGraph –– sol. 2
2
1
Cagney2001BPGraph –– sol. 2
8
6
5
3
ItoFull2001BPGraph –– sol. 2
2364
1601
838
75
p_{FN}
p_{FP}
