# Supplementary material for: Coverage and error models of protein-protein interaction data by directed graph analysis
Source: Genome Biol. 2007 Sep 10;8(9):R186. doi: 10.1186/gb-2007-8-9-r186 (PMC2375024; doi:10.1186/gb-2007-8-9-r186)
Supplement: Additional data file 2 — Presented is the Bioconductor package ppiStats (version 1.3.5 of 22 June 2007) in 'source' format. ppiStats contains the novel methods developed in this paper. [file gb-2007-8-9-r186-S2.gz › ppiStats/inst/doc/ItoFull2001BPGraph.html]

ItoFull2001BPGraph: Viable Baits Gene to GO CC Conditional test for over-representation

| GOCCID | Pvalue | OddsRatio | ExpCount | Count | Size | Term |
| GO:0044424 | 0.00 | 1.87 | 346 | 384 | 4527 | intracellular part |
| GO:0031965 | 0.00 | 4.50 | 5 | 17 | 64 | nuclear membrane |
| GO:0005643 | 0.00 | 4.81 | 4 | 14 | 50 | nuclear pore |
| GO:0000780 | 0.00 | 3.89 | 4 | 12 | 50 | condensed nuclear chromosome, pericentric region |
| GO:0005634 | 0.00 | 1.43 | 139 | 172 | 1814 | nucleus |
| GO:0000793 | 0.00 | 2.91 | 6 | 16 | 84 | condensed chromosome |
| GO:0044430 | 0.00 | 2.06 | 15 | 27 | 190 | cytoskeletal part |
| GO:0015630 | 0.00 | 2.44 | 8 | 17 | 103 | microtubule cytoskeleton |
| GO:0000775 | 0.00 | 3.01 | 5 | 12 | 61 | chromosome, pericentric region |
| GO:0005623 | 0.00 | 1.55 | 379 | 399 | 4954 | cell |
| GO:0042579 | 0.01 | 2.78 | 4 | 10 | 54 | microbody |


ItoFull2001BPGraph: Viable Baits Gene to GO CC Conditional test for under-representation

| GOCCID | Pvalue | OddsRatio | ExpCount | Count | Size | Term |
| GO:0005739 | 0.00 | 0.41 | 79 | 38 | 1035 | mitochondrion |
| GO:0031966 | 0.00 | 0.34 | 19 | 7 | 247 | mitochondrial membrane |
| GO:0030312 | 0.00 | 0.12 | 8 | 1 | 99 | external encapsulating structure |
| GO:0009277 | 0.00 | 0.12 | 8 | 1 | 99 | cell wall (sensu Fungi) |
| GO:0005842 | 0.01 | 0.14 | 7 | 1 | 87 | cytosolic large ribosomal subunit (sensu Eukaryota) |


ItoFull2001BPGraph: Viable Baits Gene to GO CC Conditional test for over-representation

| GOCCID | Pvalue | OddsRatio | ExpCount | Count | Size | Term |
| GO:0044424 | 0.00 | 1.87 | 346 | 384 | 4524 | intracellular part |
| GO:0031965 | 0.00 | 4.50 | 5 | 17 | 64 | nuclear membrane |
| GO:0005643 | 0.00 | 4.81 | 4 | 14 | 50 | nuclear pore |
| GO:0000780 | 0.00 | 3.89 | 4 | 12 | 50 | condensed nuclear chromosome, pericentric region |
| GO:0005634 | 0.00 | 1.43 | 139 | 172 | 1814 | nucleus |
| GO:0000793 | 0.00 | 2.91 | 6 | 16 | 84 | condensed chromosome |
| GO:0044430 | 0.00 | 2.06 | 15 | 27 | 190 | cytoskeletal part |
| GO:0015630 | 0.00 | 2.44 | 8 | 17 | 103 | microtubule cytoskeleton |
| GO:0000775 | 0.00 | 3.01 | 5 | 12 | 61 | chromosome, pericentric region |
| GO:0005623 | 0.00 | 1.55 | 379 | 399 | 4954 | cell |
| GO:0042579 | 0.01 | 2.78 | 4 | 10 | 54 | microbody |


ItoFull2001BPGraph: Viable Baits Gene to GO CC Conditional test for under-representation

| GOCCID | Pvalue | OddsRatio | ExpCount | Count | Size | Term |
| GO:0005739 | 0.00 | 0.41 | 79 | 38 | 1035 | mitochondrion |
| GO:0031966 | 0.00 | 0.34 | 19 | 7 | 247 | mitochondrial membrane |
| GO:0030312 | 0.00 | 0.12 | 8 | 1 | 99 | external encapsulating structure |
| GO:0009277 | 0.00 | 0.12 | 8 | 1 | 99 | cell wall (sensu Fungi) |
| GO:0005842 | 0.01 | 0.14 | 7 | 1 | 87 | cytosolic large ribosomal subunit (sensu Eukaryota) |


ItoFull2001BPGraph: Viable Baits Gene to GO CC Conditional test for over-representation

| GOCCID | Pvalue | OddsRatio | ExpCount | Count | Size | Term |
| GO:0044424 | 0.00 | 1.87 | 346 | 384 | 4524 | intracellular part |
| GO:0031965 | 0.00 | 4.50 | 5 | 17 | 64 | nuclear membrane |
| GO:0005643 | 0.00 | 4.81 | 4 | 14 | 50 | nuclear pore |
| GO:0000780 | 0.00 | 3.89 | 4 | 12 | 50 | condensed nuclear chromosome, pericentric region |
| GO:0005634 | 0.00 | 1.43 | 139 | 172 | 1814 | nucleus |
| GO:0000793 | 0.00 | 2.91 | 6 | 16 | 84 | condensed chromosome |
| GO:0044430 | 0.00 | 2.06 | 15 | 27 | 190 | cytoskeletal part |
| GO:0015630 | 0.00 | 2.44 | 8 | 17 | 103 | microtubule cytoskeleton |
| GO:0000775 | 0.00 | 3.01 | 5 | 12 | 61 | chromosome, pericentric region |
| GO:0005623 | 0.00 | 1.55 | 379 | 399 | 4954 | cell |
| GO:0042579 | 0.01 | 2.78 | 4 | 10 | 54 | microbody |


ItoFull2001BPGraph: Viable Baits Gene to GO CC Conditional test for under-representation

| GOCCID | Pvalue | OddsRatio | ExpCount | Count | Size | Term |
| GO:0005739 | 0.00 | 0.41 | 79 | 38 | 1035 | mitochondrion |
| GO:0031966 | 0.00 | 0.34 | 19 | 7 | 247 | mitochondrial membrane |
| GO:0030312 | 0.00 | 0.12 | 8 | 1 | 99 | external encapsulating structure |
| GO:0009277 | 0.00 | 0.12 | 8 | 1 | 99 | cell wall (sensu Fungi) |
| GO:0005842 | 0.01 | 0.14 | 7 | 1 | 87 | cytosolic large ribosomal subunit (sensu Eukaryota) |


ItoFull2001BPGraph: Viable Baits Gene to GO CC Conditional test for over-representation

| GOCCID | Pvalue | OddsRatio | ExpCount | Count | Size | Term |
| GO:0044424 | 0.00 | 1.87 | 346 | 384 | 4524 | intracellular part |
| GO:0031965 | 0.00 | 4.50 | 5 | 17 | 64 | nuclear membrane |
| GO:0005643 | 0.00 | 4.81 | 4 | 14 | 50 | nuclear pore |
| GO:0000780 | 0.00 | 3.89 | 4 | 12 | 50 | condensed nuclear chromosome, pericentric region |
| GO:0005634 | 0.00 | 1.43 | 139 | 172 | 1814 | nucleus |
| GO:0000793 | 0.00 | 2.91 | 6 | 16 | 84 | condensed chromosome |
| GO:0044430 | 0.00 | 2.06 | 15 | 27 | 190 | cytoskeletal part |
| GO:0015630 | 0.00 | 2.44 | 8 | 17 | 103 | microtubule cytoskeleton |
| GO:0000775 | 0.00 | 3.01 | 5 | 12 | 61 | chromosome, pericentric region |
| GO:0005623 | 0.00 | 1.55 | 379 | 399 | 4954 | cell |
| GO:0042579 | 0.01 | 2.78 | 4 | 10 | 54 | microbody |


ItoFull2001BPGraph: Viable Baits Gene to GO CC Conditional test for under-representation

| GOCCID | Pvalue | OddsRatio | ExpCount | Count | Size | Term |
| GO:0005739 | 0.00 | 0.41 | 79 | 38 | 1035 | mitochondrion |
| GO:0031966 | 0.00 | 0.34 | 19 | 7 | 247 | mitochondrial membrane |
| GO:0030312 | 0.00 | 0.12 | 8 | 1 | 99 | external encapsulating structure |
| GO:0009277 | 0.00 | 0.12 | 8 | 1 | 99 | cell wall (sensu Fungi) |
| GO:0005842 | 0.01 | 0.14 | 7 | 1 | 87 | cytosolic large ribosomal subunit (sensu Eukaryota) |


ItoFull2001BPGraph: Viable Baits Gene to GO CC Conditional test for over-representation

| GOCCID | Pvalue | OddsRatio | ExpCount | Count | Size | Term |
| GO:0044424 | 0.00 | 1.87 | 346 | 384 | 4524 | intracellular part |
| GO:0031965 | 0.00 | 4.50 | 5 | 17 | 64 | nuclear membrane |
| GO:0005643 | 0.00 | 4.81 | 4 | 14 | 50 | nuclear pore |
| GO:0000780 | 0.00 | 3.89 | 4 | 12 | 50 | condensed nuclear chromosome, pericentric region |
| GO:0005634 | 0.00 | 1.43 | 139 | 172 | 1814 | nucleus |
| GO:0000793 | 0.00 | 2.91 | 6 | 16 | 84 | condensed chromosome |
| GO:0044430 | 0.00 | 2.06 | 15 | 27 | 190 | cytoskeletal part |
| GO:0015630 | 0.00 | 2.44 | 8 | 17 | 103 | microtubule cytoskeleton |
| GO:0000775 | 0.00 | 3.01 | 5 | 12 | 61 | chromosome, pericentric region |
| GO:0005623 | 0.00 | 1.55 | 379 | 399 | 4954 | cell |
| GO:0042579 | 0.01 | 2.78 | 4 | 10 | 54 | microbody |


ItoFull2001BPGraph: Viable Baits Gene to GO CC Conditional test for under-representation

| GOCCID | Pvalue | OddsRatio | ExpCount | Count | Size | Term |
| GO:0005739 | 0.00 | 0.41 | 79 | 38 | 1035 | mitochondrion |
| GO:0031966 | 0.00 | 0.34 | 19 | 7 | 247 | mitochondrial membrane |
| GO:0030312 | 0.00 | 0.12 | 8 | 1 | 99 | external encapsulating structure |
| GO:0009277 | 0.00 | 0.12 | 8 | 1 | 99 | cell wall (sensu Fungi) |
| GO:0005842 | 0.01 | 0.14 | 7 | 1 | 87 | cytosolic large ribosomal subunit (sensu Eukaryota) |


ItoFull2001BPGraph: Viable Baits Gene to GO CC Conditional test for over-representation

| GOCCID | Pvalue | OddsRatio | ExpCount | Count | Size | Term |
| GO:0044424 | 0.00 | 1.87 | 346 | 384 | 4524 | intracellular part |
| GO:0031965 | 0.00 | 4.50 | 5 | 17 | 64 | nuclear membrane |
| GO:0005643 | 0.00 | 4.81 | 4 | 14 | 50 | nuclear pore |
| GO:0000780 | 0.00 | 3.89 | 4 | 12 | 50 | condensed nuclear chromosome, pericentric region |
| GO:0005634 | 0.00 | 1.43 | 139 | 172 | 1814 | nucleus |
| GO:0000793 | 0.00 | 2.91 | 6 | 16 | 84 | condensed chromosome |
| GO:0044430 | 0.00 | 2.06 | 15 | 27 | 190 | cytoskeletal part |
| GO:0015630 | 0.00 | 2.44 | 8 | 17 | 103 | microtubule cytoskeleton |
| GO:0000775 | 0.00 | 3.01 | 5 | 12 | 61 | chromosome, pericentric region |
| GO:0005623 | 0.00 | 1.55 | 379 | 399 | 4954 | cell |
| GO:0042579 | 0.01 | 2.78 | 4 | 10 | 54 | microbody |


ItoFull2001BPGraph: Viable Baits Gene to GO CC Conditional test for under-representation

| GOCCID | Pvalue | OddsRatio | ExpCount | Count | Size | Term |
| GO:0005739 | 0.00 | 0.41 | 79 | 38 | 1035 | mitochondrion |
| GO:0031966 | 0.00 | 0.34 | 19 | 7 | 247 | mitochondrial membrane |
| GO:0030312 | 0.00 | 0.12 | 8 | 1 | 99 | external encapsulating structure |
| GO:0009277 | 0.00 | 0.12 | 8 | 1 | 99 | cell wall (sensu Fungi) |
| GO:0005842 | 0.01 | 0.14 | 7 | 1 | 87 | cytosolic large ribosomal subunit (sensu Eukaryota) |


ItoFull2001BPGraph: Viable Baits Gene to GO CC Conditional test for over-representation

| GOCCID | Pvalue | OddsRatio | ExpCount | Count | Size | Term |
| GO:0044424 | 0.00 | 1.87 | 346 | 384 | 4524 | intracellular part |
| GO:0031965 | 0.00 | 4.50 | 5 | 17 | 64 | nuclear membrane |
| GO:0005643 | 0.00 | 4.81 | 4 | 14 | 50 | nuclear pore |
| GO:0000780 | 0.00 | 3.89 | 4 | 12 | 50 | condensed nuclear chromosome, pericentric region |
| GO:0005634 | 0.00 | 1.43 | 139 | 172 | 1814 | nucleus |
| GO:0000793 | 0.00 | 2.91 | 6 | 16 | 84 | condensed chromosome |
| GO:0044430 | 0.00 | 2.06 | 15 | 27 | 190 | cytoskeletal part |
| GO:0015630 | 0.00 | 2.44 | 8 | 17 | 103 | microtubule cytoskeleton |
| GO:0000775 | 0.00 | 3.01 | 5 | 12 | 61 | chromosome, pericentric region |
| GO:0005623 | 0.00 | 1.55 | 379 | 399 | 4954 | cell |
| GO:0042579 | 0.01 | 2.78 | 4 | 10 | 54 | microbody |


ItoFull2001BPGraph: Viable Baits Gene to GO CC Conditional test for under-representation

| GOCCID | Pvalue | OddsRatio | ExpCount | Count | Size | Term |
| GO:0005739 | 0.00 | 0.41 | 79 | 38 | 1035 | mitochondrion |
| GO:0031966 | 0.00 | 0.34 | 19 | 7 | 247 | mitochondrial membrane |
| GO:0030312 | 0.00 | 0.12 | 8 | 1 | 99 | external encapsulating structure |
| GO:0009277 | 0.00 | 0.12 | 8 | 1 | 99 | cell wall (sensu Fungi) |
| GO:0005842 | 0.01 | 0.14 | 7 | 1 | 87 | cytosolic large ribosomal subunit (sensu Eukaryota) |


ItoFull2001BPGraph: Viable Baits Gene to GO CC Conditional test for over-representation

| GOCCID | Pvalue | OddsRatio | ExpCount | Count | Size | Term |
| GO:0044424 | 0.00 | 1.87 | 346 | 384 | 4524 | intracellular part |
| GO:0031965 | 0.00 | 4.50 | 5 | 17 | 64 | nuclear membrane |
| GO:0005643 | 0.00 | 4.81 | 4 | 14 | 50 | nuclear pore |
| GO:0000780 | 0.00 | 3.89 | 4 | 12 | 50 | condensed nuclear chromosome, pericentric region |
| GO:0005634 | 0.00 | 1.43 | 139 | 172 | 1814 | nucleus |
| GO:0000793 | 0.00 | 2.91 | 6 | 16 | 84 | condensed chromosome |
| GO:0044430 | 0.00 | 2.06 | 15 | 27 | 190 | cytoskeletal part |
| GO:0015630 | 0.00 | 2.44 | 8 | 17 | 103 | microtubule cytoskeleton |
| GO:0000775 | 0.00 | 3.01 | 5 | 12 | 61 | chromosome, pericentric region |
| GO:0005623 | 0.00 | 1.55 | 379 | 399 | 4954 | cell |
| GO:0042579 | 0.01 | 2.78 | 4 | 10 | 54 | microbody |


ItoFull2001BPGraph: Viable Baits Gene to GO CC Conditional test for under-representation

| GOCCID | Pvalue | OddsRatio | ExpCount | Count | Size | Term |
| GO:0005739 | 0.00 | 0.41 | 79 | 38 | 1035 | mitochondrion |
| GO:0031966 | 0.00 | 0.34 | 19 | 7 | 247 | mitochondrial membrane |
| GO:0030312 | 0.00 | 0.12 | 8 | 1 | 99 | external encapsulating structure |
| GO:0009277 | 0.00 | 0.12 | 8 | 1 | 99 | cell wall (sensu Fungi) |
| GO:0005842 | 0.01 | 0.14 | 7 | 1 | 87 | cytosolic large ribosomal subunit (sensu Eukaryota) |


ItoFull2001BPGraph: Viable Baits Gene to GO CC Conditional test for over-representation

| GOCCID | Pvalue | OddsRatio | ExpCount | Count | Size | Term |
| GO:0044424 | 0.00 | 1.87 | 346 | 384 | 4524 | intracellular part |
| GO:0031965 | 0.00 | 4.50 | 5 | 17 | 64 | nuclear membrane |
| GO:0005643 | 0.00 | 4.81 | 4 | 14 | 50 | nuclear pore |
| GO:0000780 | 0.00 | 3.89 | 4 | 12 | 50 | condensed nuclear chromosome, pericentric region |
| GO:0005634 | 0.00 | 1.43 | 139 | 172 | 1814 | nucleus |
| GO:0000793 | 0.00 | 2.91 | 6 | 16 | 84 | condensed chromosome |
| GO:0044430 | 0.00 | 2.06 | 15 | 27 | 190 | cytoskeletal part |
| GO:0015630 | 0.00 | 2.44 | 8 | 17 | 103 | microtubule cytoskeleton |
| GO:0000775 | 0.00 | 3.01 | 5 | 12 | 61 | chromosome, pericentric region |
| GO:0005623 | 0.00 | 1.55 | 379 | 399 | 4954 | cell |
| GO:0042579 | 0.01 | 2.78 | 4 | 10 | 54 | microbody |


ItoFull2001BPGraph: Viable Baits Gene to GO CC Conditional test for under-representation

| GOCCID | Pvalue | OddsRatio | ExpCount | Count | Size | Term |
| GO:0005739 | 0.00 | 0.41 | 79 | 38 | 1035 | mitochondrion |
| GO:0031966 | 0.00 | 0.34 | 19 | 7 | 247 | mitochondrial membrane |
| GO:0030312 | 0.00 | 0.12 | 8 | 1 | 99 | external encapsulating structure |
| GO:0009277 | 0.00 | 0.12 | 8 | 1 | 99 | cell wall (sensu Fungi) |
| GO:0005842 | 0.01 | 0.14 | 7 | 1 | 87 | cytosolic large ribosomal subunit (sensu Eukaryota) |


ItoFull2001BPGraph: Viable Baits Gene to GO CC Conditional test for over-representation

| GOCCID | Pvalue | OddsRatio | ExpCount | Count | Size | Term |
| GO:0044424 | 0.00 | 1.87 | 346 | 384 | 4527 | intracellular part |
| GO:0031965 | 0.00 | 4.50 | 5 | 17 | 64 | nuclear membrane |
| GO:0005643 | 0.00 | 4.81 | 4 | 14 | 50 | nuclear pore |
| GO:0000780 | 0.00 | 3.89 | 4 | 12 | 50 | condensed nuclear chromosome, pericentric region |
| GO:0005634 | 0.00 | 1.43 | 139 | 172 | 1814 | nucleus |
| GO:0000793 | 0.00 | 2.91 | 6 | 16 | 84 | condensed chromosome |
| GO:0044430 | 0.00 | 2.06 | 15 | 27 | 190 | cytoskeletal part |
| GO:0015630 | 0.00 | 2.44 | 8 | 17 | 103 | microtubule cytoskeleton |
| GO:0000775 | 0.00 | 3.01 | 5 | 12 | 61 | chromosome, pericentric region |
| GO:0005623 | 0.00 | 1.55 | 379 | 399 | 4954 | cell |
| GO:0042579 | 0.01 | 2.78 | 4 | 10 | 54 | microbody |


ItoFull2001BPGraph: Viable Baits Gene to GO CC Conditional test for under-representation

| GOCCID | Pvalue | OddsRatio | ExpCount | Count | Size | Term |
| GO:0005739 | 0.00 | 0.41 | 79 | 38 | 1035 | mitochondrion |
| GO:0031966 | 0.00 | 0.34 | 19 | 7 | 247 | mitochondrial membrane |
| GO:0030312 | 0.00 | 0.12 | 8 | 1 | 99 | external encapsulating structure |
| GO:0009277 | 0.00 | 0.12 | 8 | 1 | 99 | cell wall (sensu Fungi) |
| GO:0005842 | 0.01 | 0.14 | 7 | 1 | 87 | cytosolic large ribosomal subunit (sensu Eukaryota) |


ItoFull2001BPGraph: Viable Baits Gene to GO CC Conditional test for over-representation

| GOCCID | Pvalue | OddsRatio | ExpCount | Count | Size | Term |
| GO:0044424 | 0.00 | 1.87 | 346 | 384 | 4524 | intracellular part |
| GO:0031965 | 0.00 | 4.50 | 5 | 17 | 64 | nuclear membrane |
| GO:0005643 | 0.00 | 4.81 | 4 | 14 | 50 | nuclear pore |
| GO:0000780 | 0.00 | 3.89 | 4 | 12 | 50 | condensed nuclear chromosome, pericentric region |
| GO:0005634 | 0.00 | 1.43 | 139 | 172 | 1814 | nucleus |
| GO:0000793 | 0.00 | 2.91 | 6 | 16 | 84 | condensed chromosome |
| GO:0044430 | 0.00 | 2.06 | 15 | 27 | 190 | cytoskeletal part |
| GO:0015630 | 0.00 | 2.44 | 8 | 17 | 103 | microtubule cytoskeleton |
| GO:0000775 | 0.00 | 3.01 | 5 | 12 | 61 | chromosome, pericentric region |
| GO:0005623 | 0.00 | 1.55 | 379 | 399 | 4954 | cell |
| GO:0042579 | 0.01 | 2.78 | 4 | 10 | 54 | microbody |


ItoFull2001BPGraph: Viable Baits Gene to GO CC Conditional test for under-representation

| GOCCID | Pvalue | OddsRatio | ExpCount | Count | Size | Term |
| GO:0005739 | 0.00 | 0.41 | 79 | 38 | 1035 | mitochondrion |
| GO:0031966 | 0.00 | 0.34 | 19 | 7 | 247 | mitochondrial membrane |
| GO:0030312 | 0.00 | 0.12 | 8 | 1 | 99 | external encapsulating structure |
| GO:0009277 | 0.00 | 0.12 | 8 | 1 | 99 | cell wall (sensu Fungi) |
| GO:0005842 | 0.01 | 0.14 | 7 | 1 | 87 | cytosolic large ribosomal subunit (sensu Eukaryota) |


ItoFull2001BPGraph: Viable Baits Gene to GO CC Conditional test for over-representation

| GOCCID | Pvalue | OddsRatio | ExpCount | Count | Size | Term |
| GO:0044424 | 0.00 | 1.87 | 346 | 384 | 4524 | intracellular part |
| GO:0031965 | 0.00 | 4.50 | 5 | 17 | 64 | nuclear membrane |
| GO:0005643 | 0.00 | 4.81 | 4 | 14 | 50 | nuclear pore |
| GO:0000780 | 0.00 | 3.89 | 4 | 12 | 50 | condensed nuclear chromosome, pericentric region |
| GO:0005634 | 0.00 | 1.43 | 139 | 172 | 1814 | nucleus |
| GO:0000793 | 0.00 | 2.91 | 6 | 16 | 84 | condensed chromosome |
| GO:0044430 | 0.00 | 2.06 | 15 | 27 | 190 | cytoskeletal part |
| GO:0015630 | 0.00 | 2.44 | 8 | 17 | 103 | microtubule cytoskeleton |
| GO:0000775 | 0.00 | 3.01 | 5 | 12 | 61 | chromosome, pericentric region |
| GO:0005623 | 0.00 | 1.55 | 379 | 399 | 4954 | cell |
| GO:0042579 | 0.01 | 2.78 | 4 | 10 | 54 | microbody |


ItoFull2001BPGraph: Viable Baits Gene to GO CC Conditional test for under-representation

| GOCCID | Pvalue | OddsRatio | ExpCount | Count | Size | Term |
| GO:0005739 | 0.00 | 0.41 | 79 | 38 | 1035 | mitochondrion |
| GO:0031966 | 0.00 | 0.34 | 19 | 7 | 247 | mitochondrial membrane |
| GO:0030312 | 0.00 | 0.12 | 8 | 1 | 99 | external encapsulating structure |
| GO:0009277 | 0.00 | 0.12 | 8 | 1 | 99 | cell wall (sensu Fungi) |
| GO:0005842 | 0.01 | 0.14 | 7 | 1 | 87 | cytosolic large ribosomal subunit (sensu Eukaryota) |


ItoFull2001BPGraph: Viable Baits Gene to GO CC Conditional test for over-representation

| GOCCID | Pvalue | OddsRatio | ExpCount | Count | Size | Term |
| GO:0044424 | 0.00 | 1.87 | 346 | 384 | 4524 | intracellular part |
| GO:0031965 | 0.00 | 4.50 | 5 | 17 | 64 | nuclear membrane |
| GO:0005643 | 0.00 | 4.81 | 4 | 14 | 50 | nuclear pore |
| GO:0000780 | 0.00 | 3.89 | 4 | 12 | 50 | condensed nuclear chromosome, pericentric region |
| GO:0005634 | 0.00 | 1.43 | 139 | 172 | 1814 | nucleus |
| GO:0000793 | 0.00 | 2.91 | 6 | 16 | 84 | condensed chromosome |
| GO:0044430 | 0.00 | 2.06 | 15 | 27 | 190 | cytoskeletal part |
| GO:0015630 | 0.00 | 2.44 | 8 | 17 | 103 | microtubule cytoskeleton |
| GO:0000775 | 0.00 | 3.01 | 5 | 12 | 61 | chromosome, pericentric region |
| GO:0005623 | 0.00 | 1.55 | 379 | 399 | 4954 | cell |
| GO:0042579 | 0.01 | 2.78 | 4 | 10 | 54 | microbody |


ItoFull2001BPGraph: Viable Baits Gene to GO CC Conditional test for under-representation

| GOCCID | Pvalue | OddsRatio | ExpCount | Count | Size | Term |
| GO:0005739 | 0.00 | 0.41 | 79 | 38 | 1035 | mitochondrion |
| GO:0031966 | 0.00 | 0.34 | 19 | 7 | 247 | mitochondrial membrane |
| GO:0030312 | 0.00 | 0.12 | 8 | 1 | 99 | external encapsulating structure |
| GO:0009277 | 0.00 | 0.12 | 8 | 1 | 99 | cell wall (sensu Fungi) |
| GO:0005842 | 0.01 | 0.14 | 7 | 1 | 87 | cytosolic large ribosomal subunit (sensu Eukaryota) |


ItoFull2001BPGraph: Viable Baits Gene to GO CC Conditional test for over-representation

| GOCCID | Pvalue | OddsRatio | ExpCount | Count | Size | Term |
| GO:0044424 | 0.00 | 1.87 | 346 | 384 | 4524 | intracellular part |
| GO:0031965 | 0.00 | 4.50 | 5 | 17 | 64 | nuclear membrane |
| GO:0005643 | 0.00 | 4.81 | 4 | 14 | 50 | nuclear pore |
| GO:0000780 | 0.00 | 3.89 | 4 | 12 | 50 | condensed nuclear chromosome, pericentric region |
| GO:0005634 | 0.00 | 1.43 | 139 | 172 | 1814 | nucleus |
| GO:0000793 | 0.00 | 2.91 | 6 | 16 | 84 | condensed chromosome |
| GO:0044430 | 0.00 | 2.06 | 15 | 27 | 190 | cytoskeletal part |
| GO:0015630 | 0.00 | 2.44 | 8 | 17 | 103 | microtubule cytoskeleton |
| GO:0000775 | 0.00 | 3.01 | 5 | 12 | 61 | chromosome, pericentric region |
| GO:0005623 | 0.00 | 1.55 | 379 | 399 | 4954 | cell |
| GO:0042579 | 0.01 | 2.78 | 4 | 10 | 54 | microbody |


ItoFull2001BPGraph: Viable Baits Gene to GO CC Conditional test for under-representation

| GOCCID | Pvalue | OddsRatio | ExpCount | Count | Size | Term |
| GO:0005739 | 0.00 | 0.41 | 79 | 38 | 1035 | mitochondrion |
| GO:0031966 | 0.00 | 0.34 | 19 | 7 | 247 | mitochondrial membrane |
| GO:0030312 | 0.00 | 0.12 | 8 | 1 | 99 | external encapsulating structure |
| GO:0009277 | 0.00 | 0.12 | 8 | 1 | 99 | cell wall (sensu Fungi) |
| GO:0005842 | 0.01 | 0.14 | 7 | 1 | 87 | cytosolic large ribosomal subunit (sensu Eukaryota) |


ItoFull2001BPGraph: Viable Baits Gene to GO CC Conditional test for over-representation

| GOCCID | Pvalue | OddsRatio | ExpCount | Count | Size | Term |
| GO:0044424 | 0.00 | 1.87 | 346 | 384 | 4524 | intracellular part |
| GO:0031965 | 0.00 | 4.50 | 5 | 17 | 64 | nuclear membrane |
| GO:0005643 | 0.00 | 4.81 | 4 | 14 | 50 | nuclear pore |
| GO:0000780 | 0.00 | 3.89 | 4 | 12 | 50 | condensed nuclear chromosome, pericentric region |
| GO:0005634 | 0.00 | 1.43 | 139 | 172 | 1814 | nucleus |
| GO:0000793 | 0.00 | 2.91 | 6 | 16 | 84 | condensed chromosome |
| GO:0044430 | 0.00 | 2.06 | 15 | 27 | 190 | cytoskeletal part |
| GO:0015630 | 0.00 | 2.44 | 8 | 17 | 103 | microtubule cytoskeleton |
| GO:0000775 | 0.00 | 3.01 | 5 | 12 | 61 | chromosome, pericentric region |
| GO:0005623 | 0.00 | 1.55 | 379 | 399 | 4954 | cell |
| GO:0042579 | 0.01 | 2.78 | 4 | 10 | 54 | microbody |


ItoFull2001BPGraph: Viable Baits Gene to GO CC Conditional test for under-representation

| GOCCID | Pvalue | OddsRatio | ExpCount | Count | Size | Term |
| GO:0005739 | 0.00 | 0.41 | 79 | 38 | 1035 | mitochondrion |
| GO:0031966 | 0.00 | 0.34 | 19 | 7 | 247 | mitochondrial membrane |
| GO:0030312 | 0.00 | 0.12 | 8 | 1 | 99 | external encapsulating structure |
| GO:0009277 | 0.00 | 0.12 | 8 | 1 | 99 | cell wall (sensu Fungi) |
| GO:0005842 | 0.01 | 0.14 | 7 | 1 | 87 | cytosolic large ribosomal subunit (sensu Eukaryota) |


ItoFull2001BPGraph: Viable Baits Gene to GO CC Conditional test for over-representation

| GOCCID | Pvalue | OddsRatio | ExpCount | Count | Size | Term |
| GO:0044424 | 0.00 | 1.87 | 346 | 384 | 4524 | intracellular part |
| GO:0031965 | 0.00 | 4.50 | 5 | 17 | 64 | nuclear membrane |
| GO:0005643 | 0.00 | 4.81 | 4 | 14 | 50 | nuclear pore |
| GO:0000780 | 0.00 | 3.89 | 4 | 12 | 50 | condensed nuclear chromosome, pericentric region |
| GO:0005634 | 0.00 | 1.43 | 139 | 172 | 1814 | nucleus |
| GO:0000793 | 0.00 | 2.91 | 6 | 16 | 84 | condensed chromosome |
| GO:0044430 | 0.00 | 2.06 | 15 | 27 | 190 | cytoskeletal part |
| GO:0015630 | 0.00 | 2.44 | 8 | 17 | 103 | microtubule cytoskeleton |
| GO:0000775 | 0.00 | 3.01 | 5 | 12 | 61 | chromosome, pericentric region |
| GO:0005623 | 0.00 | 1.55 | 379 | 399 | 4954 | cell |
| GO:0042579 | 0.01 | 2.78 | 4 | 10 | 54 | microbody |


ItoFull2001BPGraph: Viable Baits Gene to GO CC Conditional test for under-representation

| GOCCID | Pvalue | OddsRatio | ExpCount | Count | Size | Term |
| GO:0005739 | 0.00 | 0.41 | 79 | 38 | 1035 | mitochondrion |
| GO:0031966 | 0.00 | 0.34 | 19 | 7 | 247 | mitochondrial membrane |
| GO:0030312 | 0.00 | 0.12 | 8 | 1 | 99 | external encapsulating structure |
| GO:0009277 | 0.00 | 0.12 | 8 | 1 | 99 | cell wall (sensu Fungi) |
| GO:0005842 | 0.01 | 0.14 | 7 | 1 | 87 | cytosolic large ribosomal subunit (sensu Eukaryota) |
