# Supplementary material for: Coverage and error models of protein-protein interaction data by directed graph analysis
Source: Genome Biol. 2007 Sep 10;8(9):R186. doi: 10.1186/gb-2007-8-9-r186 (PMC2375024; doi:10.1186/gb-2007-8-9-r186)
Supplement: Additional data file 2 — Presented is the Bioconductor package ppiStats (version 1.3.5 of 22 June 2007) in 'source' format. ppiStats contains the novel methods developed in this paper. [file gb-2007-8-9-r186-S2.gz › ppiStats/inst/Scripts/Cagney2001.html]

Cagney2001: Viable Baits Gene to GO BP Conditional test for over-representation

| GOBPID | Pvalue | OddsRatio | ExpCount | Count | Size | Term |
| GO:0006511 | 0.00 | Inf | 0 | 19 | 146 | ubiquitin-dependent protein catabolic process |
| GO:0051603 | 0.00 | Inf | 0 | 19 | 148 | proteolysis involved in cellular protein catabolic process |
| GO:0043632 | 0.00 | Inf | 1 | 19 | 153 | modification-dependent macromolecule catabolic process |
| GO:0030163 | 0.00 | Inf | 1 | 19 | 171 | protein catabolic process |
| GO:0009057 | 0.00 | Inf | 1 | 19 | 314 | macromolecule catabolic process |
| GO:0044248 | 0.00 | Inf | 1 | 19 | 390 | cellular catabolic process |
| GO:0044267 | 0.00 | Inf | 4 | 19 | 1143 | cellular protein metabolic process |
| GO:0043283 | 0.00 | Inf | 6 | 19 | 1800 | biopolymer metabolic process |
| GO:0044238 | 0.00 | Inf | 9 | 19 | 2763 | primary metabolic process |
| GO:0009987 | 0.00 | Inf | 14 | 19 | 4342 | cellular process |


Cagney2001: Viable Prey Gene to GO BP Conditional test for over-representation

| GOBPID | Pvalue | OddsRatio | ExpCount | Count | Size | Term |
| GO:0030163 | 0.00 | 39.20 | 1 | 20 | 171 | protein catabolic process |
| GO:0006511 | 0.00 | 42.24 | 1 | 19 | 146 | ubiquitin-dependent protein catabolic process |
| GO:0051603 | 0.00 | 41.57 | 1 | 19 | 148 | proteolysis involved in cellular protein catabolic process |
| GO:0043632 | 0.00 | 39.99 | 1 | 19 | 153 | modification-dependent macromolecule catabolic process |
| GO:0009057 | 0.00 | 19.62 | 2 | 20 | 314 | macromolecule catabolic process |
| GO:0044248 | 0.00 | 13.84 | 3 | 19 | 390 | cellular catabolic process |
| GO:0044267 | 0.00 | 9.39 | 8 | 27 | 1143 | cellular protein metabolic process |
| GO:0043283 | 0.00 | 6.55 | 12 | 29 | 1800 | biopolymer metabolic process |
| GO:0044238 | 0.00 | 7.59 | 19 | 34 | 2763 | primary metabolic process |
| GO:0006406 | 0.00 | 10.88 | 0 | 4 | 64 | mRNA export from nucleus |
| GO:0009987 | 0.00 | 6.31 | 29 | 37 | 4342 | cellular process |
| GO:0050658 | 0.00 | 8.93 | 1 | 4 | 77 | RNA transport |
| GO:0015931 | 0.00 | 7.93 | 1 | 4 | 86 | nucleobase, nucleoside, nucleotide and nucleic acid transport |
| GO:0006403 | 0.00 | 7.84 | 1 | 4 | 87 | RNA localization |
| GO:0051168 | 0.00 | 6.98 | 1 | 4 | 97 | nuclear export |


Cagney2001: Viable Baits Gene to GO MF Conditional test for over-representation

| GOMFID | Pvalue | OddsRatio | ExpCount | Count | Size | Term |
| GO:0004175 | 0.00 | 1220.17 | 0 | 17 | 57 | endopeptidase activity |
| GO:0016787 | 0.00 | 60.05 | 2 | 17 | 734 | hydrolase activity |


Cagney2001: Viable Prey Gene to GO MF Conditional test for over-representation

| GOMFID | Pvalue | OddsRatio | ExpCount | Count | Size | Term |
| GO:0004175 | 0.00 | 85.12 | 0 | 15 | 57 | endopeptidase activity |
| GO:0016787 | 0.00 | 5.30 | 3 | 12 | 734 | hydrolase activity |
| GO:0016887 | 0.00 | 5.30 | 1 | 6 | 197 | ATPase activity |
| GO:0016817 | 0.01 | 3.70 | 2 | 6 | 276 | hydrolase activity, acting on acid anhydrides |
| GO:0016462 | 0.01 | 3.70 | 2 | 6 | 276 | pyrophosphatase activity |


Cagney2001: Viable Baits Gene to GO CC Conditional test for under-representation

| GOCCID | Pvalue | OddsRatio | ExpCount | Count | Size | Term |
| GO:0044446 | 0.00 | 0.00 | 7 | 0 | 2078 | intracellular organelle part |
| GO:0043226 | 0.00 | 0.19 | 12 | 5 | 3756 | organelle |
| GO:0043231 | 0.00 | 0.25 | 11 | 5 | 3423 | intracellular membrane-bound organelle |
| GO:0044444 | 0.00 | 0.17 | 8 | 2 | 2357 | cytoplasmic part |


Cagney2001: Viable Prey Gene to GO CC Conditional test for under-representation

| GOCCID | Pvalue | OddsRatio | ExpCount | Count | Size | Term |
| GO:0044444 | 0.00 | 0.32 | 16 | 7 | 2357 | cytoplasmic part |


Cagney2001: Viable Baits Gene to GO BP Conditional test for under-representation

| GOBPID | Pvalue | OddsRatio | ExpCount | Count | Size | Term |
| GO:0006139 | 0.01 | 0.00 | 5 | 0 | 1402 | nucleobase, nucleoside, nucleotide and nucleic acid metabolic process |
| GO:0006996 | 0.01 | 0.00 | 4 | 0 | 1272 | organelle organization and biogenesis |
