# Supplementary material for: Coverage and error models of protein-protein interaction data by directed graph analysis
Source: Genome Biol. 2007 Sep 10;8(9):R186. doi: 10.1186/gb-2007-8-9-r186 (PMC2375024; doi:10.1186/gb-2007-8-9-r186)
Supplement: Additional data file 2 — Presented is the Bioconductor package ppiStats (version 1.3.5 of 22 June 2007) in 'source' format. ppiStats contains the novel methods developed in this paper. [file gb-2007-8-9-r186-S2.gz › ppiStats/inst/Scripts/Gavin2002.html]

Gavin2002: Viable Baits Gene to GO CC Conditional test for over-representation

| GOCCID | Pvalue | OddsRatio | ExpCount | Count | Size | Term |
| GO:0005634 | 0.00 | 2.99 | 141 | 250 | 1814 | nucleus |
| GO:0005622 | 0.00 | 7.21 | 356 | 435 | 4563 | intracellular |
| GO:0044446 | 0.00 | 2.90 | 159 | 267 | 2078 | intracellular organelle part |
| GO:0005654 | 0.00 | 4.81 | 25 | 84 | 326 | nucleoplasm |
| GO:0043233 | 0.00 | 3.28 | 57 | 133 | 736 | organelle lumen |
| GO:0005623 | 0.00 | 9.29 | 386 | 444 | 4954 | cell |
| GO:0043227 | 0.00 | 2.19 | 267 | 339 | 3423 | membrane-bound organelle |
| GO:0043234 | 0.00 | 2.13 | 55 | 97 | 1519 | protein complex |
| GO:0005681 | 0.00 | 4.14 | 5 | 17 | 78 | spliceosome |
| GO:0043232 | 0.00 | 1.77 | 55 | 85 | 931 | intracellular non-membrane-bound organelle |
| GO:0000228 | 0.00 | 2.41 | 14 | 30 | 183 | nuclear chromosome |
| GO:0016591 | 0.00 | 4.14 | 4 | 12 | 72 | DNA-directed RNA polymerase II, holoenzyme |
| GO:0005667 | 0.00 | 3.23 | 6 | 16 | 130 | transcription factor complex |
| GO:0044452 | 0.00 | 2.60 | 8 | 18 | 102 | nucleolar part |
| GO:0005730 | 0.00 | 2.33 | 10 | 20 | 226 | nucleolus |
| GO:0030529 | 0.01 | 1.55 | 34 | 49 | 516 | ribonucleoprotein complex |
| GO:0031982 | 0.01 | 2.17 | 8 | 16 | 105 | vesicle |


Gavin2002: Viable Prey Gene to GO CC Conditional test for over-representation

| GOCCID | Pvalue | OddsRatio | ExpCount | Count | Size | Term |
| GO:0043233 | 0.00 | 7.00 | 148 | 407 | 736 | organelle lumen |
| GO:0005622 | 0.00 | 9.68 | 918 | 1128 | 4563 | intracellular |
| GO:0005654 | 0.00 | 9.48 | 66 | 217 | 326 | nucleoplasm |
| GO:0044446 | 0.00 | 3.53 | 391 | 669 | 2078 | intracellular organelle part |
| GO:0005623 | 0.00 | 14.99 | 996 | 1152 | 4954 | cell |
| GO:0005634 | 0.00 | 3.15 | 365 | 612 | 1814 | nucleus |
| GO:0043227 | 0.00 | 2.56 | 688 | 883 | 3423 | membrane-bound organelle |
| GO:0005730 | 0.00 | 5.38 | 45 | 125 | 226 | nucleolus |
| GO:0000313 | 0.00 | 9.87 | 16 | 57 | 81 | organellar ribosome |
| GO:0005732 | 0.00 | 8.87 | 11 | 39 | 57 | small nucleolar ribonucleoprotein complex |
| GO:0005681 | 0.00 | 7.35 | 13 | 43 | 78 | spliceosome |
| GO:0005759 | 0.00 | 3.11 | 31 | 67 | 163 | mitochondrial matrix |
| GO:0043232 | 0.00 | 1.94 | 101 | 158 | 931 | intracellular non-membrane-bound organelle |
| GO:0043234 | 0.00 | 1.88 | 111 | 169 | 1519 | protein complex |
| GO:0030529 | 0.00 | 1.91 | 72 | 112 | 516 | ribonucleoprotein complex |
| GO:0005667 | 0.00 | 3.22 | 9 | 20 | 130 | transcription factor complex |
| GO:0000228 | 0.00 | 1.84 | 37 | 57 | 183 | nuclear chromosome |
| GO:0005856 | 0.00 | 1.69 | 41 | 60 | 204 | cytoskeleton |
| GO:0005935 | 0.00 | 1.99 | 23 | 37 | 112 | bud neck |
| GO:0031982 | 0.00 | 2.02 | 21 | 35 | 105 | vesicle |
| GO:0005794 | 0.00 | 1.72 | 36 | 53 | 178 | Golgi apparatus |
| GO:0030532 | 0.00 | Inf | 1 | 4 | 59 | small nuclear ribonucleoprotein complex |
| GO:0016585 | 0.00 | 2.73 | 8 | 17 | 78 | chromatin remodeling complex |
| GO:0005934 | 0.00 | 2.46 | 10 | 19 | 50 | bud tip |
| GO:0015935 | 0.00 | 1.95 | 19 | 31 | 95 | small ribosomal subunit |
| GO:0044448 | 0.01 | 2.33 | 10 | 18 | 89 | cell cortex part |


Gavin2002: Viable Baits Gene to GO BP Conditional test for over-representation

| GOBPID | Pvalue | OddsRatio | ExpCount | Count | Size | Term |
| GO:0043170 | 0.00 | 3.80 | 184 | 317 | 2355 | macromolecule metabolic process |
| GO:0044238 | 0.00 | 3.43 | 215 | 335 | 2763 | primary metabolic process |
| GO:0044237 | 0.00 | 2.07 | 197 | 272 | 2988 | cellular metabolic process |
| GO:0000375 | 0.00 | 5.14 | 8 | 30 | 103 | RNA splicing, via transesterification reactions |
| GO:0050789 | 0.00 | 2.15 | 55 | 98 | 708 | regulation of biological process |
| GO:0006402 | 0.00 | 6.14 | 5 | 20 | 60 | mRNA catabolic process |
| GO:0042254 | 0.00 | 2.97 | 18 | 45 | 321 | ribosome biogenesis and assembly |
| GO:0031323 | 0.00 | 2.34 | 36 | 70 | 459 | regulation of cellular metabolic process |
| GO:0006397 | 0.00 | 9.87 | 2 | 13 | 139 | mRNA processing |
| GO:0044267 | 0.00 | 1.84 | 76 | 117 | 1143 | cellular protein metabolic process |
| GO:0000398 | 0.00 | 4.69 | 6 | 21 | 95 | nuclear mRNA splicing, via spliceosome |
| GO:0006333 | 0.00 | 3.73 | 9 | 26 | 112 | chromatin assembly or disassembly |
| GO:0006323 | 0.00 | 2.69 | 19 | 42 | 238 | DNA packaging |
| GO:0016070 | 0.00 | 2.09 | 40 | 71 | 918 | RNA metabolic process |
| GO:0045449 | 0.00 | 2.32 | 27 | 54 | 350 | regulation of transcription |
| GO:0045892 | 0.00 | 3.20 | 11 | 29 | 141 | negative regulation of transcription, DNA-dependent |
| GO:0007001 | 0.00 | 2.02 | 41 | 71 | 551 | chromosome organization and biogenesis (sensu Eukaryota) |
| GO:0043285 | 0.00 | 2.47 | 21 | 44 | 268 | biopolymer catabolic process |
| GO:0016072 | 0.00 | 2.87 | 14 | 33 | 176 | rRNA metabolic process |
| GO:0006996 | 0.00 | 1.84 | 57 | 91 | 1272 | organelle organization and biogenesis |
| GO:0016458 | 0.00 | 3.78 | 7 | 21 | 89 | gene silencing |
| GO:0045814 | 0.00 | 3.78 | 7 | 21 | 89 | negative regulation of gene expression, epigenetic |
| GO:0031507 | 0.00 | 3.78 | 7 | 21 | 89 | heterochromatin formation |
| GO:0006260 | 0.00 | 3.35 | 9 | 24 | 112 | DNA replication |
| GO:0016568 | 0.00 | 2.59 | 16 | 36 | 209 | chromatin modification |
| GO:0006365 | 0.00 | 3.93 | 6 | 19 | 78 | 35S primary transcript processing |
| GO:0065004 | 0.00 | 3.99 | 6 | 18 | 73 | protein-DNA complex assembly |
| GO:0009892 | 0.00 | 2.59 | 15 | 33 | 191 | negative regulation of metabolic process |
| GO:0045934 | 0.00 | 2.69 | 13 | 29 | 162 | negative regulation of nucleobase, nucleoside, nucleotide and nucleic acid metabolic process |
| GO:0044265 | 0.00 | 2.90 | 10 | 25 | 284 | cellular macromolecule catabolic process |
| GO:0009056 | 0.00 | 1.98 | 31 | 55 | 404 | catabolic process |
| GO:0043412 | 0.00 | 1.81 | 44 | 71 | 569 | biopolymer modification |
| GO:0006396 | 0.00 | 2.40 | 16 | 33 | 350 | RNA processing |
| GO:0048523 | 0.00 | 2.28 | 17 | 34 | 218 | negative regulation of cellular process |
| GO:0022618 | 0.00 | 4.37 | 4 | 13 | 130 | protein-RNA complex assembly |
| GO:0006348 | 0.00 | 4.14 | 4 | 13 | 51 | chromatin silencing at telomere |
| GO:0006974 | 0.00 | 2.18 | 18 | 34 | 226 | response to DNA damage stimulus |
| GO:0000074 | 0.00 | 2.34 | 13 | 26 | 162 | regulation of progression through cell cycle |
| GO:0006366 | 0.00 | 3.49 | 5 | 13 | 308 | transcription from RNA polymerase II promoter |
| GO:0006351 | 0.00 | 2.43 | 9 | 19 | 471 | transcription, DNA-dependent |
| GO:0043543 | 0.00 | 3.21 | 4 | 12 | 57 | protein amino acid acylation |
| GO:0051641 | 0.00 | 4.57 | 2 | 8 | 559 | cellular localization |
| GO:0045045 | 0.00 | 1.90 | 19 | 32 | 238 | secretory pathway |
| GO:0006913 | 0.00 | 2.23 | 10 | 19 | 122 | nucleocytoplasmic transport |
| GO:0022402 | 0.00 | 2.70 | 6 | 13 | 399 | cell cycle process |
| GO:0042273 | 0.00 | 2.83 | 5 | 12 | 63 | ribosomal large subunit biogenesis and assembly |
| GO:0051169 | 0.00 | 2.25 | 9 | 18 | 115 | nuclear transport |
| GO:0043632 | 0.00 | 2.04 | 12 | 22 | 153 | modification-dependent macromolecule catabolic process |
| GO:0022403 | 0.00 | 1.71 | 24 | 37 | 328 | cell cycle phase |
| GO:0006357 | 0.00 | 1.91 | 15 | 26 | 206 | regulation of transcription from RNA polymerase II promoter |
| GO:0000087 | 0.00 | 2.15 | 10 | 19 | 126 | M phase of mitotic cell cycle |
| GO:0007010 | 0.01 | 1.77 | 17 | 28 | 220 | cytoskeleton organization and biogenesis |
| GO:0048193 | 0.01 | 1.92 | 13 | 22 | 161 | Golgi vesicle transport |
| GO:0007015 | 0.01 | 2.64 | 5 | 11 | 61 | actin filament organization |
| GO:0030036 | 0.01 | 3.00 | 4 | 9 | 106 | actin cytoskeleton organization and biogenesis |
| GO:0042255 | 0.01 | 2.59 | 5 | 11 | 62 | ribosome assembly |
| GO:0016570 | 0.01 | 2.92 | 4 | 9 | 81 | histone modification |
| GO:0006508 | 0.01 | 1.85 | 14 | 23 | 174 | proteolysis |
| GO:0022414 | 0.01 | 1.66 | 21 | 32 | 267 | reproductive process |
| GO:0006511 | 0.01 | 1.92 | 11 | 20 | 146 | ubiquitin-dependent protein catabolic process |
| GO:0000278 | 0.01 | 2.03 | 9 | 17 | 244 | mitotic cell cycle |
| GO:0051649 | 0.01 | 1.45 | 41 | 56 | 530 | establishment of cellular localization |


Gavin2002: Viable Prey Gene to GO BP Conditional test for over-representation

| GOBPID | Pvalue | OddsRatio | ExpCount | Count | Size | Term |
| GO:0043283 | 0.00 | 3.58 | 127 | 271 | 1800 | biopolymer metabolic process |
| GO:0032774 | 0.00 | 3.36 | 95 | 201 | 476 | RNA biosynthetic process |
| GO:0006350 | 0.00 | 3.18 | 104 | 213 | 517 | transcription |
| GO:0006139 | 0.00 | 3.06 | 107 | 215 | 1402 | nucleobase, nucleoside, nucleotide and nucleic acid metabolic process |
| GO:0006365 | 0.00 | 13.90 | 16 | 60 | 78 | 35S primary transcript processing |
| GO:0006325 | 0.00 | 4.31 | 48 | 119 | 238 | establishment and/or maintenance of chromatin architecture |
| GO:0016071 | 0.00 | 6.44 | 26 | 78 | 191 | mRNA metabolic process |
| GO:0000375 | 0.00 | 6.29 | 21 | 62 | 103 | RNA splicing, via transesterification reactions |
| GO:0000398 | 0.00 | 8.43 | 15 | 51 | 95 | nuclear mRNA splicing, via spliceosome |
| GO:0016569 | 0.00 | 7.83 | 16 | 53 | 81 | covalent chromatin modification |
| GO:0006996 | 0.00 | 2.05 | 179 | 277 | 1272 | organelle organization and biogenesis |
| GO:0006396 | 0.00 | 4.01 | 26 | 63 | 350 | RNA processing |
| GO:0006338 | 0.00 | 3.82 | 26 | 61 | 149 | chromatin remodeling |
| GO:0006259 | 0.00 | 2.64 | 50 | 97 | 503 | DNA metabolic process |
| GO:0006366 | 0.00 | 2.70 | 46 | 90 | 308 | transcription from RNA polymerase II promoter |
| GO:0050794 | 0.00 | 1.86 | 136 | 204 | 678 | regulation of cellular process |
| GO:0044267 | 0.00 | 1.80 | 155 | 226 | 1143 | cellular protein metabolic process |
| GO:0006402 | 0.00 | 6.85 | 10 | 32 | 60 | mRNA catabolic process |
| GO:0043285 | 0.00 | 3.85 | 20 | 47 | 268 | biopolymer catabolic process |
| GO:0044265 | 0.00 | 3.17 | 26 | 57 | 284 | cellular macromolecule catabolic process |
| GO:0065007 | 0.00 | 1.72 | 157 | 223 | 783 | biological regulation |
| GO:0009056 | 0.00 | 2.02 | 81 | 131 | 404 | catabolic process |
| GO:0019222 | 0.00 | 1.97 | 85 | 135 | 488 | regulation of metabolic process |
| GO:0006355 | 0.00 | 2.10 | 64 | 107 | 327 | regulation of transcription, DNA-dependent |
| GO:0019219 | 0.00 | 2.02 | 73 | 118 | 396 | regulation of nucleobase, nucleoside, nucleotide and nucleic acid metabolic process |
| GO:0031497 | 0.00 | 3.39 | 20 | 44 | 97 | chromatin assembly |
| GO:0040029 | 0.00 | 3.44 | 19 | 43 | 94 | regulation of gene expression, epigenetic |
| GO:0016481 | 0.00 | 2.62 | 29 | 57 | 146 | negative regulation of transcription |
| GO:0006260 | 0.00 | 2.95 | 23 | 47 | 112 | DNA replication |
| GO:0051649 | 0.00 | 1.74 | 107 | 155 | 530 | establishment of cellular localization |
| GO:0043170 | 0.00 | 1.71 | 112 | 160 | 2355 | macromolecule metabolic process |
| GO:0007049 | 0.00 | 1.81 | 84 | 126 | 417 | cell cycle |
| GO:0065004 | 0.00 | 3.54 | 15 | 34 | 73 | protein-DNA complex assembly |
| GO:0042273 | 0.00 | 9.19 | 5 | 16 | 63 | ribosomal large subunit biogenesis and assembly |
| GO:0009719 | 0.00 | 2.04 | 47 | 78 | 235 | response to endogenous stimulus |
| GO:0019954 | 0.00 | 3.15 | 16 | 35 | 80 | asexual reproduction |
| GO:0006913 | 0.00 | 2.55 | 25 | 47 | 122 | nucleocytoplasmic transport |
| GO:0051169 | 0.00 | 2.62 | 23 | 45 | 115 | nuclear transport |
| GO:0042255 | 0.00 | 3.55 | 12 | 29 | 62 | ribosome assembly |
| GO:0006364 | 0.00 | 4.03 | 9 | 23 | 166 | rRNA processing |
| GO:0048519 | 0.00 | 1.94 | 46 | 74 | 230 | negative regulation of biological process |
| GO:0030029 | 0.00 | 2.51 | 22 | 42 | 110 | actin filament-based process |
| GO:0031324 | 0.00 | 2.08 | 36 | 61 | 181 | negative regulation of cellular metabolic process |
| GO:0043543 | 0.00 | 3.38 | 11 | 26 | 57 | protein amino acid acylation |
| GO:0030468 | 0.00 | 2.51 | 21 | 39 | 102 | establishment of cell polarity (sensu Fungi) |
| GO:0006888 | 0.00 | 2.81 | 16 | 32 | 78 | ER to Golgi vesicle-mediated transport |
| GO:0043632 | 0.00 | 2.09 | 31 | 52 | 153 | modification-dependent macromolecule catabolic process |
| GO:0000723 | 0.00 | 1.76 | 54 | 81 | 269 | telomere maintenance |
| GO:0051329 | 0.00 | 2.48 | 19 | 35 | 92 | interphase of mitotic cell cycle |
| GO:0007163 | 0.00 | 2.28 | 23 | 41 | 114 | establishment and/or maintenance of cell polarity |
| GO:0030163 | 0.00 | 1.93 | 34 | 55 | 171 | protein catabolic process |
| GO:0016043 | 0.00 | 1.41 | 144 | 181 | 2008 | cell organization and biogenesis |
| GO:0022403 | 0.00 | 1.63 | 61 | 86 | 328 | cell cycle phase |
| GO:0007033 | 0.00 | 2.65 | 13 | 25 | 63 | vacuole organization and biogenesis |
| GO:0043412 | 0.00 | 1.46 | 106 | 138 | 569 | biopolymer modification |
| GO:0006511 | 0.00 | 1.92 | 29 | 47 | 146 | ubiquitin-dependent protein catabolic process |
| GO:0006406 | 0.00 | 2.58 | 13 | 25 | 64 | mRNA export from nucleus |
| GO:0006302 | 0.00 | 2.81 | 10 | 21 | 51 | double-strand break repair |
| GO:0048193 | 0.00 | 2.28 | 17 | 30 | 161 | Golgi vesicle transport |
| GO:0051603 | 0.00 | 1.88 | 30 | 47 | 148 | proteolysis involved in cellular protein catabolic process |
| GO:0045045 | 0.00 | 1.84 | 32 | 50 | 238 | secretory pathway |
| GO:0007001 | 0.00 | 1.57 | 63 | 87 | 551 | chromosome organization and biogenesis (sensu Eukaryota) |
| GO:0042254 | 0.00 | 2.19 | 18 | 31 | 321 | ribosome biogenesis and assembly |
| GO:0007059 | 0.00 | 1.99 | 23 | 38 | 115 | chromosome segregation |
| GO:0006412 | 0.00 | 1.52 | 67 | 90 | 372 | translation |
| GO:0006348 | 0.00 | 2.59 | 10 | 20 | 51 | chromatin silencing at telomere |
| GO:0006950 | 0.00 | 1.41 | 93 | 119 | 464 | response to stress |
| GO:0006944 | 0.00 | 2.41 | 11 | 21 | 56 | membrane fusion |
| GO:0006461 | 0.00 | 1.95 | 20 | 32 | 98 | protein complex assembly |
| GO:0032506 | 0.00 | 2.05 | 17 | 28 | 83 | cytokinetic process |
| GO:0006342 | 0.00 | 5.33 | 3 | 8 | 89 | chromatin silencing |
| GO:0006281 | 0.00 | 1.92 | 20 | 32 | 181 | DNA repair |
| GO:0007015 | 0.00 | 2.26 | 12 | 22 | 61 | actin filament organization |
| GO:0051246 | 0.00 | 2.20 | 13 | 23 | 65 | regulation of protein metabolic process |
| GO:0022618 | 0.00 | 7.98 | 2 | 6 | 130 | protein-RNA complex assembly |
| GO:0006403 | 0.00 | 2.05 | 15 | 26 | 87 | RNA localization |
| GO:0050658 | 0.00 | 2.05 | 15 | 26 | 77 | RNA transport |
| GO:0000074 | 0.00 | 1.65 | 33 | 47 | 162 | regulation of progression through cell cycle |
| GO:0009653 | 0.00 | 1.51 | 50 | 67 | 247 | anatomical structure morphogenesis |
| GO:0040007 | 0.01 | 1.66 | 28 | 40 | 137 | growth |
| GO:0000087 | 0.01 | 1.67 | 25 | 37 | 126 | M phase of mitotic cell cycle |
| GO:0000122 | 0.01 | 2.19 | 10 | 18 | 51 | negative regulation of transcription from RNA polymerase II promoter |
| GO:0008104 | 0.01 | 1.41 | 60 | 77 | 298 | protein localization |


Gavin2002: Viable Baits Gene to GO MF Conditional test for over-representation

| GOMFID | Pvalue | OddsRatio | ExpCount | Count | Size | Term |
| GO:0003723 | 0.00 | 3.42 | 12 | 33 | 236 | RNA binding |
| GO:0016887 | 0.00 | 2.99 | 15 | 38 | 197 | ATPase activity |
| GO:0016251 | 0.00 | 5.40 | 5 | 19 | 62 | general RNA polymerase II transcription factor activity |
| GO:0016817 | 0.00 | 2.44 | 22 | 45 | 276 | hydrolase activity, acting on acid anhydrides |
| GO:0016462 | 0.00 | 2.44 | 22 | 45 | 276 | pyrophosphatase activity |
| GO:0031202 | 0.00 | 4.58 | 4 | 14 | 51 | RNA splicing factor activity, transesterification mechanism |
| GO:0005515 | 0.00 | 1.94 | 33 | 57 | 443 | protein binding |
| GO:0045182 | 0.00 | 4.03 | 4 | 14 | 56 | translation regulator activity |
| GO:0003676 | 0.00 | 1.83 | 21 | 35 | 505 | nucleic acid binding |
| GO:0004518 | 0.00 | 2.47 | 8 | 17 | 100 | nuclease activity |
| GO:0016787 | 0.00 | 1.58 | 36 | 52 | 734 | hydrolase activity |
| GO:0005488 | 0.00 | 2.03 | 11 | 21 | 1056 | binding |
| GO:0030234 | 0.01 | 1.86 | 15 | 25 | 188 | enzyme regulator activity |


Gavin2002: Viable Prey Gene to GO MF Conditional test for over-representation

| GOMFID | Pvalue | OddsRatio | ExpCount | Count | Size | Term |
| GO:0003723 | 0.00 | 4.00 | 26 | 63 | 236 | RNA binding |
| GO:0016817 | 0.00 | 2.62 | 56 | 106 | 276 | hydrolase activity, acting on acid anhydrides |
| GO:0016462 | 0.00 | 2.62 | 56 | 106 | 276 | pyrophosphatase activity |
| GO:0016251 | 0.00 | 10.14 | 8 | 30 | 62 | general RNA polymerase II transcription factor activity |
| GO:0031202 | 0.00 | 6.83 | 10 | 32 | 51 | RNA splicing factor activity, transesterification mechanism |
| GO:0045182 | 0.00 | 5.02 | 11 | 31 | 56 | translation regulator activity |
| GO:0030528 | 0.00 | 2.03 | 62 | 102 | 320 | transcription regulator activity |
| GO:0016887 | 0.00 | 4.32 | 12 | 31 | 197 | ATPase activity |
| GO:0003676 | 0.00 | 2.00 | 54 | 88 | 505 | nucleic acid binding |
| GO:0004175 | 0.00 | 3.63 | 11 | 27 | 57 | endopeptidase activity |
| GO:0005515 | 0.00 | 1.71 | 77 | 112 | 443 | protein binding |
| GO:0003824 | 0.00 | 1.35 | 384 | 448 | 1907 | catalytic activity |
| GO:0008094 | 0.00 | 3.67 | 9 | 21 | 51 | DNA-dependent ATPase activity |
| GO:0005198 | 0.00 | 2.04 | 25 | 41 | 338 | structural molecule activity |
| GO:0042623 | 0.00 | 2.27 | 17 | 31 | 137 | ATPase activity, coupled |
| GO:0008092 | 0.00 | 2.71 | 10 | 21 | 52 | cytoskeletal protein binding |
| GO:0030234 | 0.00 | 1.72 | 38 | 56 | 188 | enzyme regulator activity |
| GO:0004518 | 0.00 | 1.98 | 20 | 33 | 100 | nuclease activity |
| GO:0016772 | 0.00 | 1.48 | 59 | 79 | 295 | transferase activity, transferring phosphorus-containing groups |
| GO:0016810 | 0.00 | 2.04 | 15 | 25 | 74 | hydrolase activity, acting on carbon-nitrogen (but not peptide) bonds |
| GO:0003735 | 0.01 | 1.48 | 44 | 58 | 216 | structural constituent of ribosome |


Gavin2002: Viable Baits Gene to GO CC Conditional test for under-representation

| GOCCID | Pvalue | OddsRatio | ExpCount | Count | Size | Term |
| GO:0005886 | 0.00 | 0.28 | 20 | 6 | 255 | plasma membrane |
| GO:0005743 | 0.00 | 0.15 | 13 | 2 | 161 | mitochondrial inner membrane |
| GO:0005740 | 0.00 | 0.33 | 22 | 8 | 288 | mitochondrial envelope |
| GO:0031301 | 0.01 | 0.00 | 5 | 0 | 64 | integral to organelle membrane |


Gavin2002: Viable Prey Gene to GO CC Conditional test for under-representation

| GOCCID | Pvalue | OddsRatio | ExpCount | Count | Size | Term |
| GO:0005886 | 0.00 | 0.29 | 51 | 18 | 255 | plasma membrane |
| GO:0005740 | 0.00 | 0.33 | 58 | 23 | 288 | mitochondrial envelope |
| GO:0005783 | 0.00 | 0.38 | 69 | 31 | 343 | endoplasmic reticulum |
| GO:0030312 | 0.00 | 0.21 | 20 | 5 | 99 | external encapsulating structure |
| GO:0009277 | 0.00 | 0.21 | 20 | 5 | 99 | cell wall (sensu Fungi) |
| GO:0005743 | 0.00 | 0.34 | 32 | 13 | 161 | mitochondrial inner membrane |
| GO:0031975 | 0.00 | 0.55 | 79 | 49 | 391 | envelope |
| GO:0005842 | 0.00 | 0.24 | 17 | 5 | 87 | cytosolic large ribosomal subunit (sensu Eukaryota) |
| GO:0016020 | 0.00 | 0.71 | 164 | 128 | 1071 | membrane |
| GO:0042579 | 0.00 | 0.15 | 11 | 2 | 54 | microbody |
| GO:0044455 | 0.00 | 0.40 | 20 | 9 | 98 | mitochondrial membrane part |
| GO:0005737 | 0.00 | 0.84 | 673 | 632 | 3346 | cytoplasm |
| GO:0031301 | 0.01 | 0.33 | 13 | 5 | 64 | integral to organelle membrane |
| GO:0005843 | 0.01 | 0.35 | 12 | 5 | 62 | cytosolic small ribosomal subunit (sensu Eukaryota) |
| GO:0005741 | 0.01 | 0.43 | 16 | 8 | 82 | mitochondrial outer membrane |
| GO:0019867 | 0.01 | 0.43 | 16 | 8 | 82 | outer membrane |


Gavin2002: Viable Baits Gene to GO BP Conditional test for under-representation

| GOBPID | Pvalue | OddsRatio | ExpCount | Count | Size | Term |
| GO:0006812 | 0.00 | 0.13 | 7 | 1 | 92 | cation transport |


Gavin2002: Viable Prey Gene to GO BP Conditional test for under-representation

| GOBPID | Pvalue | OddsRatio | ExpCount | Count | Size | Term |
| GO:0006811 | 0.00 | 0.23 | 22 | 6 | 110 | ion transport |
| GO:0006629 | 0.00 | 0.47 | 47 | 25 | 232 | lipid metabolic process |
| GO:0015849 | 0.00 | 0.08 | 10 | 1 | 51 | organic acid transport |
| GO:0006766 | 0.00 | 0.24 | 17 | 5 | 87 | vitamin metabolic process |
| GO:0030005 | 0.00 | 0.15 | 11 | 2 | 55 | di-, tri-valent inorganic cation homeostasis |
| GO:0009117 | 0.00 | 0.35 | 22 | 9 | 109 | nucleotide metabolic process |
| GO:0051186 | 0.00 | 0.47 | 34 | 18 | 167 | cofactor metabolic process |
| GO:0030001 | 0.00 | 0.22 | 11 | 3 | 57 | metal ion transport |
| GO:0015674 | 0.00 | 0.25 | 10 | 3 | 51 | di-, tri-valent inorganic cation transport |
| GO:0042221 | 0.00 | 0.67 | 70 | 51 | 347 | response to chemical stimulus |
| GO:0009108 | 0.01 | 0.33 | 13 | 5 | 65 | coenzyme biosynthetic process |
| GO:0006733 | 0.01 | 0.30 | 11 | 4 | 56 | oxidoreduction coenzyme metabolic process |
| GO:0006839 | 0.01 | 0.34 | 13 | 5 | 63 | mitochondrial transport |
| GO:0008610 | 0.01 | 0.51 | 24 | 14 | 121 | lipid biosynthetic process |
| GO:0006800 | 0.01 | 0.38 | 14 | 6 | 68 | oxygen and reactive oxygen species metabolic process |


Gavin2002: Viable Baits Gene to GO MF Conditional test for under-representation

| GOMFID | Pvalue | OddsRatio | ExpCount | Count | Size | Term |
| GO:0005215 | 0.00 | 0.40 | 32 | 14 | 408 | transporter activity |
| GO:0003735 | 0.01 | 0.44 | 17 | 8 | 216 | structural constituent of ribosome |


Gavin2002: Viable Prey Gene to GO MF Conditional test for under-representation

| GOMFID | Pvalue | OddsRatio | ExpCount | Count | Size | Term |
| GO:0005215 | 0.00 | 0.54 | 72 | 44 | 408 | transporter activity |
| GO:0005342 | 0.00 | 0.08 | 11 | 1 | 53 | organic acid transporter activity |
| GO:0016614 | 0.01 | 0.40 | 15 | 7 | 76 | oxidoreductase activity, acting on CH-OH group of donors |
| GO:0016491 | 0.01 | 0.60 | 38 | 25 | 264 | oxidoreductase activity |
