# Supplementary material for: Coverage and error models of protein-protein interaction data by directed graph analysis
Source: Genome Biol. 2007 Sep 10;8(9):R186. doi: 10.1186/gb-2007-8-9-r186 (PMC2375024; doi:10.1186/gb-2007-8-9-r186)
Supplement: Additional data file 2 — Presented is the Bioconductor package ppiStats (version 1.3.5 of 22 June 2007) in 'source' format. ppiStats contains the novel methods developed in this paper. [file gb-2007-8-9-r186-S2.gz › ppiStats/inst/Scripts/Gavin2006.html]

Gavin2006: Viable Baits Gene to GO CC Conditional test for over-representation

| GOCCID | Pvalue | OddsRatio | ExpCount | Count | Size | Term |
| GO:0005622 | 0.00 | 5.63 | 1375 | 1638 | 4563 | intracellular |
| GO:0005623 | 0.00 | 8.27 | 1493 | 1700 | 4954 | cell |
| GO:0005634 | 0.00 | 2.26 | 547 | 769 | 1814 | nucleus |
| GO:0043233 | 0.00 | 2.62 | 222 | 365 | 736 | organelle lumen |
| GO:0043227 | 0.00 | 2.00 | 1031 | 1229 | 3423 | membrane-bound organelle |
| GO:0044446 | 0.00 | 1.89 | 583 | 761 | 2078 | intracellular organelle part |
| GO:0043234 | 0.00 | 1.67 | 339 | 442 | 1519 | protein complex |
| GO:0005737 | 0.00 | 1.48 | 1008 | 1123 | 3346 | cytoplasm |
| GO:0044451 | 0.00 | 3.77 | 27 | 54 | 307 | nucleoplasm part |
| GO:0005730 | 0.00 | 2.24 | 68 | 109 | 226 | nucleolus |
| GO:0005681 | 0.00 | 3.08 | 20 | 38 | 78 | spliceosome |
| GO:0005643 | 0.00 | 3.52 | 15 | 30 | 50 | nuclear pore |
| GO:0016591 | 0.00 | 3.23 | 17 | 33 | 72 | DNA-directed RNA polymerase II, holoenzyme |
| GO:0044431 | 0.00 | 2.17 | 36 | 57 | 133 | Golgi apparatus part |
| GO:0043228 | 0.00 | 1.36 | 280 | 333 | 931 | non-membrane-bound organelle |
| GO:0005667 | 0.00 | 2.47 | 26 | 44 | 130 | transcription factor complex |
| GO:0031965 | 0.00 | 2.66 | 19 | 34 | 64 | nuclear membrane |
| GO:0005654 | 0.00 | 6.54 | 6 | 14 | 326 | nucleoplasm |
| GO:0005938 | 0.00 | 2.17 | 30 | 48 | 100 | cell cortex |
| GO:0012505 | 0.00 | 1.63 | 79 | 107 | 296 | endomembrane system |
| GO:0016023 | 0.00 | 2.09 | 31 | 48 | 102 | cytoplasmic membrane-bound vesicle |
| GO:0031982 | 0.00 | 2.06 | 32 | 49 | 105 | vesicle |
| GO:0005732 | 0.00 | 2.60 | 17 | 30 | 57 | small nucleolar ribonucleoprotein complex |
| GO:0000228 | 0.00 | 1.64 | 55 | 75 | 183 | nuclear chromosome |
| GO:0005934 | 0.00 | 2.34 | 15 | 25 | 50 | bud tip |
| GO:0030863 | 0.00 | 2.25 | 15 | 25 | 51 | cortical cytoskeleton |
| GO:0005935 | 0.01 | 1.63 | 34 | 46 | 112 | bud neck |


Gavin2006: Viable Prey Gene to GO CC Conditional test for over-representation

| GOCCID | Pvalue | OddsRatio | ExpCount | Count | Size | Term |
| GO:0005622 | 0.00 | 8.36 | 1401 | 1702 | 4563 | intracellular |
| GO:0043233 | 0.00 | 6.39 | 226 | 503 | 736 | organelle lumen |
| GO:0005623 | 0.00 | 10.02 | 1521 | 1741 | 4954 | cell |
| GO:0044446 | 0.00 | 2.70 | 498 | 758 | 2078 | intracellular organelle part |
| GO:0005634 | 0.00 | 2.57 | 557 | 817 | 1814 | nucleus |
| GO:0043232 | 0.00 | 2.79 | 217 | 365 | 931 | intracellular non-membrane-bound organelle |
| GO:0030529 | 0.00 | 3.67 | 110 | 214 | 516 | ribonucleoprotein complex |
| GO:0043227 | 0.00 | 2.00 | 1051 | 1251 | 3423 | membrane-bound organelle |
| GO:0005730 | 0.00 | 4.77 | 69 | 150 | 226 | nucleolus |
| GO:0000313 | 0.00 | 12.23 | 25 | 68 | 81 | organellar ribosome |
| GO:0005732 | 0.00 | 24.14 | 18 | 52 | 57 | small nucleolar ribonucleoprotein complex |
| GO:0043234 | 0.00 | 2.26 | 162 | 253 | 1519 | protein complex |
| GO:0043226 | 0.00 | 2.38 | 102 | 166 | 3756 | organelle |
| GO:0005759 | 0.00 | 3.75 | 42 | 85 | 163 | mitochondrial matrix |
| GO:0005681 | 0.00 | 6.80 | 21 | 50 | 78 | spliceosome |
| GO:0015934 | 0.00 | 12.16 | 14 | 37 | 131 | large ribosomal subunit |
| GO:0044451 | 0.00 | 5.68 | 23 | 54 | 307 | nucleoplasm part |
| GO:0015935 | 0.00 | 22.93 | 10 | 30 | 95 | small ribosomal subunit |
| GO:0005842 | 0.00 | 3.78 | 27 | 54 | 87 | cytosolic large ribosomal subunit (sensu Eukaryota) |
| GO:0005843 | 0.00 | 4.48 | 19 | 41 | 62 | cytosolic small ribosomal subunit (sensu Eukaryota) |
| GO:0005794 | 0.00 | 2.02 | 55 | 83 | 178 | Golgi apparatus |
| GO:0000228 | 0.00 | 1.92 | 56 | 83 | 183 | nuclear chromosome |
| GO:0044445 | 0.00 | 5.85 | 8 | 18 | 185 | cytosolic part |
| GO:0005643 | 0.00 | 3.15 | 15 | 29 | 50 | nuclear pore |
| GO:0031965 | 0.00 | 2.59 | 20 | 34 | 64 | nuclear membrane |
| GO:0031982 | 0.00 | 2.00 | 32 | 49 | 105 | vesicle |
| GO:0005938 | 0.00 | 2.03 | 31 | 47 | 100 | cell cortex |
| GO:0005737 | 0.00 | 1.21 | 1028 | 1084 | 3346 | cytoplasm |
| GO:0016591 | 0.00 | 4.92 | 6 | 13 | 72 | DNA-directed RNA polymerase II, holoenzyme |
| GO:0005667 | 0.00 | 2.59 | 14 | 25 | 130 | transcription factor complex |
| GO:0005934 | 0.00 | 2.47 | 15 | 26 | 50 | bud tip |
| GO:0005654 | 0.00 | 3.89 | 6 | 12 | 326 | nucleoplasm |
| GO:0016023 | 0.00 | 1.78 | 30 | 43 | 102 | cytoplasmic membrane-bound vesicle |
| GO:0030863 | 0.00 | 2.19 | 16 | 25 | 51 | cortical cytoskeleton |
| GO:0044427 | 0.01 | 1.58 | 45 | 60 | 192 | chromosomal part |
| GO:0005935 | 0.01 | 1.65 | 34 | 47 | 112 | bud neck |
| GO:0030532 | 0.01 | Inf | 1 | 4 | 59 | small nuclear ribonucleoprotein complex |


Gavin2006: Viable Baits Gene to GO BP Conditional test for over-representation

| GOBPID | Pvalue | OddsRatio | ExpCount | Count | Size | Term |
| GO:0044238 | 0.00 | 2.15 | 832 | 1063 | 2763 | primary metabolic process |
| GO:0043170 | 0.00 | 2.05 | 709 | 923 | 2355 | macromolecule metabolic process |
| GO:0016043 | 0.00 | 2.02 | 553 | 745 | 2008 | cell organization and biogenesis |
| GO:0042254 | 0.00 | 3.02 | 71 | 131 | 321 | ribosome biogenesis and assembly |
| GO:0044237 | 0.00 | 1.58 | 679 | 814 | 2988 | cellular metabolic process |
| GO:0051649 | 0.00 | 2.03 | 160 | 238 | 530 | establishment of cellular localization |
| GO:0016072 | 0.00 | 3.18 | 53 | 100 | 176 | rRNA metabolic process |
| GO:0006402 | 0.00 | 8.61 | 15 | 40 | 60 | mRNA catabolic process |
| GO:0051276 | 0.00 | 1.91 | 167 | 242 | 556 | chromosome organization and biogenesis |
| GO:0006365 | 0.00 | 4.75 | 23 | 52 | 78 | 35S primary transcript processing |
| GO:0051169 | 0.00 | 3.45 | 35 | 68 | 115 | nuclear transport |
| GO:0006913 | 0.00 | 3.32 | 37 | 71 | 122 | nucleocytoplasmic transport |
| GO:0006351 | 0.00 | 1.95 | 129 | 190 | 471 | transcription, DNA-dependent |
| GO:0016070 | 0.00 | 1.93 | 130 | 191 | 918 | RNA metabolic process |
| GO:0006396 | 0.00 | 2.48 | 61 | 103 | 350 | RNA processing |
| GO:0050789 | 0.00 | 1.68 | 213 | 286 | 708 | regulation of biological process |
| GO:0006325 | 0.00 | 2.88 | 38 | 69 | 238 | establishment and/or maintenance of chromatin architecture |
| GO:0000398 | 0.00 | 3.26 | 29 | 55 | 95 | nuclear mRNA splicing, via spliceosome |
| GO:0016569 | 0.00 | 3.44 | 24 | 48 | 81 | covalent chromatin modification |
| GO:0006333 | 0.00 | 2.84 | 34 | 61 | 112 | chromatin assembly or disassembly |
| GO:0006259 | 0.00 | 1.73 | 136 | 187 | 503 | DNA metabolic process |
| GO:0045184 | 0.00 | 1.97 | 83 | 124 | 275 | establishment of protein localization |
| GO:0006611 | 0.00 | 4.71 | 15 | 34 | 51 | protein export from nucleus |
| GO:0006886 | 0.00 | 1.97 | 77 | 116 | 257 | intracellular protein transport |
| GO:0000375 | 0.00 | 2.82 | 31 | 56 | 103 | RNA splicing, via transesterification reactions |
| GO:0031323 | 0.00 | 1.72 | 120 | 165 | 459 | regulation of cellular metabolic process |
| GO:0045892 | 0.00 | 2.34 | 42 | 70 | 141 | negative regulation of transcription, DNA-dependent |
| GO:0016458 | 0.00 | 2.89 | 27 | 49 | 89 | gene silencing |
| GO:0045814 | 0.00 | 2.89 | 27 | 49 | 89 | negative regulation of gene expression, epigenetic |
| GO:0031507 | 0.00 | 2.89 | 27 | 49 | 89 | heterochromatin formation |
| GO:0044265 | 0.00 | 1.83 | 86 | 123 | 284 | cellular macromolecule catabolic process |
| GO:0045449 | 0.00 | 1.72 | 105 | 146 | 350 | regulation of transcription |
| GO:0006338 | 0.00 | 3.53 | 18 | 36 | 149 | chromatin remodeling |
| GO:0006520 | 0.00 | 2.04 | 55 | 85 | 184 | amino acid metabolic process |
| GO:0007163 | 0.00 | 2.36 | 34 | 57 | 114 | establishment and/or maintenance of cell polarity |
| GO:0043285 | 0.00 | 1.77 | 81 | 114 | 268 | biopolymer catabolic process |
| GO:0030468 | 0.00 | 2.46 | 31 | 52 | 102 | establishment of cell polarity (sensu Fungi) |
| GO:0043412 | 0.00 | 1.52 | 159 | 204 | 569 | biopolymer modification |
| GO:0016044 | 0.00 | 1.98 | 51 | 77 | 173 | membrane organization and biogenesis |
| GO:0006397 | 0.00 | 5.20 | 9 | 20 | 139 | mRNA processing |
| GO:0006807 | 0.00 | 1.76 | 73 | 103 | 242 | nitrogen compound metabolic process |
| GO:0045045 | 0.00 | 2.21 | 36 | 57 | 238 | secretory pathway |
| GO:0022618 | 0.00 | 2.81 | 20 | 37 | 130 | protein-RNA complex assembly |
| GO:0048523 | 0.00 | 1.77 | 66 | 93 | 218 | negative regulation of cellular process |
| GO:0006403 | 0.00 | 2.54 | 23 | 40 | 87 | RNA localization |
| GO:0050658 | 0.00 | 2.54 | 23 | 40 | 77 | RNA transport |
| GO:0006897 | 0.00 | 2.47 | 25 | 42 | 82 | endocytosis |
| GO:0009892 | 0.00 | 1.82 | 58 | 83 | 191 | negative regulation of metabolic process |
| GO:0006348 | 0.00 | 3.09 | 15 | 29 | 51 | chromatin silencing at telomere |
| GO:0009056 | 0.00 | 1.52 | 122 | 157 | 404 | catabolic process |
| GO:0045934 | 0.00 | 1.89 | 49 | 72 | 162 | negative regulation of nucleobase, nucleoside, nucleotide and nucleic acid metabolic process |
| GO:0000910 | 0.00 | 2.22 | 30 | 49 | 101 | cytokinesis |
| GO:0044267 | 0.00 | 1.31 | 335 | 388 | 1143 | cellular protein metabolic process |
| GO:0048193 | 0.00 | 2.48 | 22 | 38 | 161 | Golgi vesicle transport |
| GO:0009309 | 0.00 | 2.08 | 33 | 52 | 111 | amine biosynthetic process |
| GO:0006888 | 0.00 | 2.35 | 23 | 39 | 78 | ER to Golgi vesicle-mediated transport |
| GO:0015931 | 0.00 | 2.24 | 26 | 42 | 86 | nucleobase, nucleoside, nucleotide and nucleic acid transport |
| GO:0065004 | 0.00 | 2.59 | 18 | 32 | 73 | protein-DNA complex assembly |
| GO:0006082 | 0.00 | 1.55 | 92 | 121 | 307 | organic acid metabolic process |
| GO:0006406 | 0.00 | 2.50 | 19 | 33 | 64 | mRNA export from nucleus |
| GO:0019954 | 0.00 | 2.23 | 24 | 39 | 80 | asexual reproduction |
| GO:0007105 | 0.00 | 2.42 | 20 | 33 | 65 | cytokinesis, site selection |
| GO:0006261 | 0.00 | 2.51 | 18 | 31 | 91 | DNA-dependent DNA replication |
| GO:0051179 | 0.00 | 1.30 | 275 | 319 | 1001 | localization |
| GO:0042273 | 0.00 | 4.38 | 7 | 15 | 63 | ribosomal large subunit biogenesis and assembly |
| GO:0030036 | 0.00 | 1.87 | 32 | 47 | 106 | actin cytoskeleton organization and biogenesis |
| GO:0006974 | 0.00 | 1.53 | 68 | 89 | 226 | response to DNA damage stimulus |
| GO:0007049 | 0.00 | 1.38 | 126 | 153 | 417 | cell cycle |
| GO:0006399 | 0.00 | 1.80 | 34 | 49 | 113 | tRNA metabolic process |
| GO:0051246 | 0.00 | 2.14 | 20 | 31 | 65 | regulation of protein metabolic process |
| GO:0000087 | 0.00 | 1.71 | 38 | 53 | 126 | M phase of mitotic cell cycle |
| GO:0000074 | 0.00 | 1.58 | 49 | 65 | 162 | regulation of progression through cell cycle |
| GO:0043543 | 0.00 | 2.10 | 17 | 27 | 57 | protein amino acid acylation |
| GO:0006468 | 0.01 | 1.77 | 28 | 40 | 93 | protein amino acid phosphorylation |
| GO:0009653 | 0.01 | 1.42 | 74 | 93 | 247 | anatomical structure morphogenesis |
| GO:0031326 | 0.01 | 1.93 | 19 | 28 | 62 | regulation of cellular biosynthetic process |
| GO:0042255 | 0.01 | 1.93 | 19 | 28 | 62 | ribosome assembly |
| GO:0000723 | 0.01 | 1.37 | 81 | 99 | 269 | telomere maintenance |


Gavin2006: Viable Prey Gene to GO BP Conditional test for over-representation

| GOBPID | Pvalue | OddsRatio | ExpCount | Count | Size | Term |
| GO:0043283 | 0.00 | 2.97 | 194 | 338 | 1800 | biopolymer metabolic process |
| GO:0009987 | 0.00 | 2.10 | 1069 | 1279 | 4342 | cellular process |
| GO:0006412 | 0.00 | 4.46 | 87 | 182 | 372 | translation |
| GO:0043170 | 0.00 | 2.87 | 170 | 295 | 2355 | macromolecule metabolic process |
| GO:0006139 | 0.00 | 2.76 | 164 | 280 | 1402 | nucleobase, nucleoside, nucleotide and nucleic acid metabolic process |
| GO:0006325 | 0.00 | 4.03 | 73 | 149 | 238 | establishment and/or maintenance of chromatin architecture |
| GO:0032774 | 0.00 | 2.68 | 145 | 245 | 476 | RNA biosynthetic process |
| GO:0006350 | 0.00 | 2.55 | 159 | 262 | 517 | transcription |
| GO:0016071 | 0.00 | 6.00 | 40 | 94 | 191 | mRNA metabolic process |
| GO:0006365 | 0.00 | 9.79 | 24 | 63 | 78 | 35S primary transcript processing |
| GO:0006396 | 0.00 | 4.22 | 49 | 102 | 350 | RNA processing |
| GO:0051641 | 0.00 | 2.20 | 172 | 264 | 559 | cellular localization |
| GO:0000375 | 0.00 | 5.68 | 32 | 73 | 103 | RNA splicing, via transesterification reactions |
| GO:0006996 | 0.00 | 1.98 | 218 | 316 | 1272 | organelle organization and biogenesis |
| GO:0000398 | 0.00 | 7.48 | 23 | 58 | 95 | nuclear mRNA splicing, via spliceosome |
| GO:0044238 | 0.00 | 1.69 | 418 | 540 | 2763 | primary metabolic process |
| GO:0006366 | 0.00 | 9.88 | 18 | 47 | 308 | transcription from RNA polymerase II promoter |
| GO:0016569 | 0.00 | 6.22 | 25 | 59 | 81 | covalent chromatin modification |
| GO:0044249 | 0.00 | 1.86 | 232 | 326 | 841 | cellular biosynthetic process |
| GO:0042255 | 0.00 | 7.92 | 19 | 48 | 62 | ribosome assembly |
| GO:0050794 | 0.00 | 1.87 | 208 | 294 | 678 | regulation of cellular process |
| GO:0065007 | 0.00 | 1.78 | 240 | 329 | 783 | biological regulation |
| GO:0006259 | 0.00 | 2.07 | 118 | 179 | 503 | DNA metabolic process |
| GO:0006338 | 0.00 | 3.35 | 39 | 75 | 149 | chromatin remodeling |
| GO:0006402 | 0.00 | 5.82 | 18 | 43 | 60 | mRNA catabolic process |
| GO:0006406 | 0.00 | 5.46 | 20 | 45 | 64 | mRNA export from nucleus |
| GO:0044267 | 0.00 | 1.71 | 213 | 287 | 1143 | cellular protein metabolic process |
| GO:0006403 | 0.00 | 4.27 | 24 | 50 | 87 | RNA localization |
| GO:0050658 | 0.00 | 4.27 | 24 | 50 | 77 | RNA transport |
| GO:0048193 | 0.00 | 3.67 | 25 | 51 | 161 | Golgi vesicle transport |
| GO:0009056 | 0.00 | 1.84 | 124 | 177 | 404 | catabolic process |
| GO:0043285 | 0.00 | 3.29 | 30 | 57 | 268 | biopolymer catabolic process |
| GO:0000723 | 0.00 | 2.03 | 83 | 125 | 269 | telomere maintenance |
| GO:0044265 | 0.00 | 2.65 | 40 | 70 | 284 | cellular macromolecule catabolic process |
| GO:0019222 | 0.00 | 1.75 | 130 | 180 | 488 | regulation of metabolic process |
| GO:0031497 | 0.00 | 3.02 | 30 | 55 | 97 | chromatin assembly |
| GO:0015931 | 0.00 | 3.20 | 26 | 50 | 86 | nucleobase, nucleoside, nucleotide and nucleic acid transport |
| GO:0007001 | 0.00 | 1.87 | 96 | 139 | 551 | chromosome organization and biogenesis (sensu Eukaryota) |
| GO:0007059 | 0.00 | 2.70 | 35 | 62 | 115 | chromosome segregation |
| GO:0019219 | 0.00 | 1.75 | 111 | 155 | 396 | regulation of nucleobase, nucleoside, nucleotide and nucleic acid metabolic process |
| GO:0065004 | 0.00 | 3.29 | 22 | 43 | 73 | protein-DNA complex assembly |
| GO:0006260 | 0.00 | 2.56 | 34 | 59 | 112 | DNA replication |
| GO:0045045 | 0.00 | 2.42 | 39 | 65 | 238 | secretory pathway |
| GO:0051168 | 0.00 | 4.29 | 14 | 30 | 97 | nuclear export |
| GO:0040029 | 0.00 | 2.73 | 29 | 51 | 94 | regulation of gene expression, epigenetic |
| GO:0043632 | 0.00 | 2.16 | 47 | 74 | 153 | modification-dependent macromolecule catabolic process |
| GO:0006974 | 0.00 | 1.87 | 69 | 101 | 226 | response to DNA damage stimulus |
| GO:0006364 | 0.00 | 3.90 | 14 | 29 | 166 | rRNA processing |
| GO:0048519 | 0.00 | 1.85 | 71 | 102 | 230 | negative regulation of biological process |
| GO:0031324 | 0.00 | 1.96 | 56 | 83 | 181 | negative regulation of cellular metabolic process |
| GO:0007010 | 0.00 | 1.82 | 68 | 97 | 220 | cytoskeleton organization and biogenesis |
| GO:0006888 | 0.00 | 2.67 | 24 | 42 | 78 | ER to Golgi vesicle-mediated transport |
| GO:0016481 | 0.00 | 2.06 | 45 | 69 | 146 | negative regulation of transcription |
| GO:0006461 | 0.00 | 2.39 | 30 | 50 | 98 | protein complex assembly |
| GO:0030163 | 0.00 | 1.93 | 53 | 78 | 171 | protein catabolic process |
| GO:0042273 | 0.00 | 6.45 | 7 | 17 | 63 | ribosomal large subunit biogenesis and assembly |
| GO:0051246 | 0.00 | 2.84 | 20 | 36 | 65 | regulation of protein metabolic process |
| GO:0030468 | 0.00 | 2.29 | 31 | 51 | 102 | establishment of cell polarity (sensu Fungi) |
| GO:0006511 | 0.00 | 2.01 | 45 | 68 | 146 | ubiquitin-dependent protein catabolic process |
| GO:0006342 | 0.00 | 3.91 | 12 | 24 | 89 | chromatin silencing |
| GO:0000278 | 0.00 | 1.96 | 47 | 70 | 244 | mitotic cell cycle |
| GO:0045184 | 0.00 | 1.66 | 84 | 115 | 275 | establishment of protein localization |
| GO:0000910 | 0.00 | 2.25 | 31 | 50 | 101 | cytokinesis |
| GO:0051603 | 0.00 | 1.95 | 45 | 68 | 148 | proteolysis involved in cellular protein catabolic process |
| GO:0006886 | 0.00 | 1.68 | 79 | 108 | 257 | intracellular protein transport |
| GO:0042254 | 0.00 | 2.29 | 27 | 44 | 321 | ribosome biogenesis and assembly |
| GO:0043412 | 0.00 | 1.42 | 162 | 200 | 569 | biopolymer modification |
| GO:0043543 | 0.00 | 2.72 | 18 | 31 | 57 | protein amino acid acylation |
| GO:0031326 | 0.00 | 2.60 | 19 | 33 | 62 | regulation of cellular biosynthetic process |
| GO:0007105 | 0.00 | 2.50 | 20 | 34 | 65 | cytokinesis, site selection |
| GO:0007163 | 0.00 | 1.99 | 35 | 53 | 114 | establishment and/or maintenance of cell polarity |
| GO:0000074 | 0.00 | 1.80 | 46 | 66 | 162 | regulation of progression through cell cycle |
| GO:0051329 | 0.00 | 2.10 | 28 | 44 | 92 | interphase of mitotic cell cycle |
| GO:0046907 | 0.00 | 1.95 | 35 | 52 | 520 | intracellular transport |
| GO:0000279 | 0.00 | 1.57 | 76 | 101 | 249 | M phase |
| GO:0006355 | 0.00 | 1.90 | 36 | 53 | 327 | regulation of transcription, DNA-dependent |
| GO:0009653 | 0.00 | 1.54 | 76 | 99 | 247 | anatomical structure morphogenesis |
| GO:0030036 | 0.00 | 1.89 | 33 | 48 | 106 | actin cytoskeleton organization and biogenesis |
| GO:0000070 | 0.00 | 2.36 | 18 | 29 | 57 | mitotic sister chromatid segregation |
| GO:0006302 | 0.00 | 2.37 | 16 | 26 | 51 | double-strand break repair |
| GO:0006611 | 0.00 | 3.30 | 8 | 16 | 51 | protein export from nucleus |
| GO:0006730 | 0.00 | 2.28 | 16 | 26 | 52 | one-carbon compound metabolic process |
| GO:0006457 | 0.00 | 1.94 | 23 | 34 | 83 | protein folding |
| GO:0040007 | 0.00 | 1.63 | 42 | 57 | 137 | growth |
| GO:0016072 | 0.00 | 13.58 | 2 | 6 | 176 | rRNA metabolic process |
| GO:0006348 | 0.00 | 2.19 | 16 | 25 | 51 | chromatin silencing at telomere |
| GO:0006357 | 0.00 | 1.50 | 59 | 76 | 206 | regulation of transcription from RNA polymerase II promoter |
| GO:0022618 | 0.00 | 7.93 | 3 | 7 | 130 | protein-RNA complex assembly |
| GO:0007067 | 0.01 | 1.96 | 21 | 31 | 125 | mitosis |
| GO:0016044 | 0.01 | 1.52 | 53 | 69 | 173 | membrane organization and biogenesis |
| GO:0006399 | 0.01 | 1.80 | 24 | 34 | 113 | tRNA metabolic process |
| GO:0007114 | 0.01 | 2.09 | 15 | 23 | 80 | cell budding |


Gavin2006: Viable Baits Gene to GO MF Conditional test for over-representation

| GOMFID | Pvalue | OddsRatio | ExpCount | Count | Size | Term |
| GO:0005488 | 0.00 | 2.17 | 185 | 283 | 1056 | binding |
| GO:0016817 | 0.00 | 2.43 | 83 | 138 | 276 | hydrolase activity, acting on acid anhydrides |
| GO:0016462 | 0.00 | 2.43 | 83 | 138 | 276 | pyrophosphatase activity |
| GO:0016887 | 0.00 | 2.52 | 59 | 101 | 197 | ATPase activity |
| GO:0016251 | 0.00 | 6.62 | 13 | 31 | 62 | general RNA polymerase II transcription factor activity |
| GO:0003723 | 0.00 | 2.73 | 38 | 68 | 236 | RNA binding |
| GO:0008094 | 0.00 | 3.95 | 15 | 32 | 51 | DNA-dependent ATPase activity |
| GO:0005515 | 0.00 | 1.67 | 115 | 157 | 443 | protein binding |
| GO:0003824 | 0.00 | 1.33 | 316 | 369 | 1907 | catalytic activity |
| GO:0030234 | 0.00 | 1.79 | 57 | 81 | 188 | enzyme regulator activity |
| GO:0031202 | 0.00 | 2.85 | 15 | 28 | 51 | RNA splicing factor activity, transesterification mechanism |
| GO:0030528 | 0.00 | 1.44 | 97 | 121 | 320 | transcription regulator activity |
| GO:0045182 | 0.00 | 2.17 | 17 | 27 | 56 | translation regulator activity |
| GO:0003924 | 0.00 | 2.17 | 16 | 26 | 54 | GTPase activity |
| GO:0016874 | 0.00 | 1.65 | 39 | 53 | 128 | ligase activity |
| GO:0016772 | 0.00 | 1.40 | 89 | 110 | 295 | transferase activity, transferring phosphorus-containing groups |
| GO:0004674 | 0.01 | 1.85 | 21 | 31 | 70 | protein serine/threonine kinase activity |
| GO:0016787 | 0.01 | 1.28 | 138 | 161 | 734 | hydrolase activity |


Gavin2006: Viable Prey Gene to GO MF Conditional test for over-representation

| GOMFID | Pvalue | OddsRatio | ExpCount | Count | Size | Term |
| GO:0003735 | 0.00 | 6.02 | 66 | 154 | 216 | structural constituent of ribosome |
| GO:0003723 | 0.00 | 6.18 | 39 | 92 | 236 | RNA binding |
| GO:0016887 | 0.00 | 3.30 | 61 | 115 | 197 | ATPase activity |
| GO:0016817 | 0.00 | 2.47 | 85 | 141 | 276 | hydrolase activity, acting on acid anhydrides |
| GO:0016462 | 0.00 | 2.47 | 85 | 141 | 276 | pyrophosphatase activity |
| GO:0016251 | 0.00 | 11.45 | 13 | 35 | 62 | general RNA polymerase II transcription factor activity |
| GO:0031202 | 0.00 | 6.05 | 16 | 37 | 51 | RNA splicing factor activity, transesterification mechanism |
| GO:0005515 | 0.00 | 1.84 | 87 | 125 | 443 | protein binding |
| GO:0008094 | 0.00 | 4.41 | 14 | 29 | 51 | DNA-dependent ATPase activity |
| GO:0003824 | 0.00 | 1.32 | 587 | 663 | 1907 | catalytic activity |
| GO:0045182 | 0.00 | 3.53 | 17 | 34 | 56 | translation regulator activity |
| GO:0030528 | 0.00 | 1.72 | 98 | 136 | 320 | transcription regulator activity |
| GO:0005198 | 0.00 | 2.22 | 38 | 60 | 338 | structural molecule activity |
| GO:0004175 | 0.00 | 3.13 | 18 | 33 | 57 | endopeptidase activity |
| GO:0003676 | 0.00 | 1.67 | 83 | 113 | 505 | nucleic acid binding |
| GO:0005488 | 0.00 | 1.78 | 45 | 64 | 1056 | binding |
| GO:0030234 | 0.00 | 1.55 | 58 | 76 | 188 | enzyme regulator activity |
| GO:0004518 | 0.00 | 1.79 | 31 | 44 | 100 | nuclease activity |
| GO:0051082 | 0.00 | 2.12 | 18 | 28 | 58 | unfolded protein binding |
| GO:0008092 | 0.01 | 2.08 | 15 | 23 | 52 | cytoskeletal protein binding |


Gavin2006: Viable Baits Gene to GO CC Conditional test for under-representation

| GOCCID | Pvalue | OddsRatio | ExpCount | Count | Size | Term |
| GO:0005842 | 0.00 | 0.30 | 26 | 10 | 87 | cytosolic large ribosomal subunit (sensu Eukaryota) |
| GO:0030312 | 0.00 | 0.35 | 30 | 13 | 99 | external encapsulating structure |
| GO:0009277 | 0.00 | 0.35 | 30 | 13 | 99 | cell wall (sensu Fungi) |
| GO:0005886 | 0.00 | 0.55 | 77 | 50 | 255 | plasma membrane |
| GO:0005773 | 0.00 | 0.54 | 58 | 37 | 194 | vacuole |
| GO:0005840 | 0.00 | 0.63 | 102 | 74 | 339 | ribosome |
| GO:0005843 | 0.00 | 0.29 | 19 | 7 | 62 | cytosolic small ribosomal subunit (sensu Eukaryota) |
| GO:0000324 | 0.00 | 0.54 | 46 | 29 | 152 | vacuole (sensu Fungi) |
| GO:0005777 | 0.01 | 0.40 | 16 | 8 | 54 | peroxisome |


Gavin2006: Viable Prey Gene to GO CC Conditional test for under-representation

| GOCCID | Pvalue | OddsRatio | ExpCount | Count | Size | Term |
| GO:0005886 | 0.00 | 0.31 | 78 | 32 | 255 | plasma membrane |
| GO:0005740 | 0.00 | 0.40 | 88 | 45 | 288 | mitochondrial envelope |
| GO:0030312 | 0.00 | 0.25 | 30 | 10 | 99 | external encapsulating structure |
| GO:0009277 | 0.00 | 0.25 | 30 | 10 | 99 | cell wall (sensu Fungi) |
| GO:0005783 | 0.00 | 0.53 | 105 | 67 | 343 | endoplasmic reticulum |
| GO:0005777 | 0.00 | 0.13 | 17 | 3 | 54 | peroxisome |
| GO:0005743 | 0.00 | 0.43 | 49 | 26 | 161 | mitochondrial inner membrane |
| GO:0031975 | 0.00 | 0.66 | 120 | 90 | 391 | envelope |
| GO:0005741 | 0.00 | 0.38 | 25 | 12 | 82 | mitochondrial outer membrane |
| GO:0019867 | 0.00 | 0.38 | 25 | 12 | 82 | outer membrane |
| GO:0016020 | 0.00 | 0.77 | 251 | 213 | 1071 | membrane |
| GO:0000324 | 0.00 | 0.55 | 47 | 30 | 152 | vacuole (sensu Fungi) |
| GO:0005773 | 0.00 | 0.60 | 60 | 41 | 194 | vacuole |
| GO:0031301 | 0.00 | 0.37 | 20 | 9 | 64 | integral to organelle membrane |
| GO:0044455 | 0.00 | 0.47 | 30 | 17 | 98 | mitochondrial membrane part |


Gavin2006: Viable Baits Gene to GO BP Conditional test for under-representation

| GOBPID | Pvalue | OddsRatio | ExpCount | Count | Size | Term |
| GO:0015849 | 0.00 | 0.31 | 15 | 6 | 51 | organic acid transport |
| GO:0007047 | 0.00 | 0.63 | 58 | 42 | 194 | cell wall organization and biogenesis |
| GO:0030435 | 0.00 | 0.55 | 36 | 23 | 119 | sporulation |


Gavin2006: Viable Prey Gene to GO BP Conditional test for under-representation

| GOBPID | Pvalue | OddsRatio | ExpCount | Count | Size | Term |
| GO:0015849 | 0.00 | 0.14 | 16 | 3 | 51 | organic acid transport |
| GO:0044255 | 0.00 | 0.56 | 67 | 44 | 219 | cellular lipid metabolic process |
| GO:0006733 | 0.00 | 0.32 | 17 | 7 | 56 | oxidoreduction coenzyme metabolic process |
| GO:0030005 | 0.00 | 0.33 | 17 | 7 | 55 | di-, tri-valent inorganic cation homeostasis |
| GO:0009108 | 0.00 | 0.41 | 20 | 10 | 65 | coenzyme biosynthetic process |
| GO:0030001 | 0.01 | 0.00 | 4 | 0 | 57 | metal ion transport |
| GO:0006812 | 0.01 | 0.29 | 11 | 4 | 92 | cation transport |
| GO:0006767 | 0.01 | 0.36 | 13 | 6 | 87 | water-soluble vitamin metabolic process |


Gavin2006: Viable Baits Gene to GO MF Conditional test for under-representation

| GOMFID | Pvalue | OddsRatio | ExpCount | Count | Size | Term |
| GO:0003735 | 0.00 | 0.33 | 65 | 28 | 216 | structural constituent of ribosome |
| GO:0005215 | 0.00 | 0.55 | 123 | 81 | 408 | transporter activity |


Gavin2006: Viable Prey Gene to GO MF Conditional test for under-representation

| GOMFID | Pvalue | OddsRatio | ExpCount | Count | Size | Term |
| GO:0005215 | 0.00 | 0.54 | 97 | 63 | 408 | transporter activity |
| GO:0005342 | 0.00 | 0.13 | 16 | 3 | 53 | organic acid transporter activity |
| GO:0003700 | 0.01 | 0.45 | 18 | 10 | 60 | transcription factor activity |
