# Supplementary material for: Coverage and error models of protein-protein interaction data by directed graph analysis
Source: Genome Biol. 2007 Sep 10;8(9):R186. doi: 10.1186/gb-2007-8-9-r186 (PMC2375024; doi:10.1186/gb-2007-8-9-r186)
Supplement: Additional data file 2 — Presented is the Bioconductor package ppiStats (version 1.3.5 of 22 June 2007) in 'source' format. ppiStats contains the novel methods developed in this paper. [file gb-2007-8-9-r186-S2.gz › ppiStats/inst/Scripts/Hazbun2003.html]

Hazbun2003: Viable Baits Gene to GO CC Conditional test for over-representation

| GOCCID | Pvalue | OddsRatio | ExpCount | Count | Size | Term |
| GO:0005622 | 0.00 | 5.63 | 50 | 61 | 4563 | intracellular |
| GO:0043231 | 0.00 | 2.76 | 38 | 51 | 3423 | intracellular membrane-bound organelle |
| GO:0005623 | 0.00 | 11.04 | 55 | 63 | 4954 | cell |
| GO:0044422 | 0.00 | 2.33 | 23 | 36 | 2078 | organelle part |
| GO:0044428 | 0.00 | 2.48 | 10 | 20 | 931 | nuclear part |


Hazbun2003: Viable Prey Gene to GO CC Conditional test for over-representation

| GOCCID | Pvalue | OddsRatio | ExpCount | Count | Size | Term |
| GO:0005654 | 0.00 | 1.95 | 100 | 148 | 326 | nucleoplasm |
| GO:0043233 | 0.00 | 1.57 | 226 | 291 | 736 | organelle lumen |
| GO:0005634 | 0.00 | 1.32 | 557 | 632 | 1814 | nucleus |
| GO:0005667 | 0.00 | 2.03 | 40 | 61 | 130 | transcription factor complex |
| GO:0043227 | 0.00 | 1.24 | 1052 | 1115 | 3423 | membrane-bound organelle |
| GO:0005730 | 0.00 | 1.64 | 69 | 94 | 226 | nucleolus |
| GO:0005623 | 0.00 | 1.33 | 1522 | 1565 | 4954 | cell |
| GO:0044446 | 0.00 | 1.22 | 639 | 696 | 2078 | intracellular organelle part |
| GO:0016591 | 0.00 | 1.92 | 22 | 33 | 72 | DNA-directed RNA polymerase II, holoenzyme |
| GO:0005933 | 0.01 | 1.57 | 45 | 60 | 147 | bud |
| GO:0043228 | 0.01 | 1.20 | 286 | 317 | 931 | non-membrane-bound organelle |


Hazbun2003: Viable Baits Gene to GO BP Conditional test for over-representation

| GOBPID | Pvalue | OddsRatio | ExpCount | Count | Size | Term |
| GO:0006396 | 0.00 | 4.95 | 4 | 15 | 350 | RNA processing |
| GO:0043283 | 0.00 | 2.90 | 20 | 36 | 1800 | biopolymer metabolic process |
| GO:0006139 | 0.00 | 2.81 | 15 | 30 | 1402 | nucleobase, nucleoside, nucleotide and nucleic acid metabolic process |
| GO:0009987 | 0.00 | 5.14 | 48 | 60 | 4342 | cellular process |
| GO:0006839 | 0.00 | 8.32 | 1 | 5 | 63 | mitochondrial transport |
| GO:0016072 | 0.00 | 4.75 | 2 | 8 | 176 | rRNA metabolic process |
| GO:0006365 | 0.00 | 6.59 | 1 | 5 | 78 | 35S primary transcript processing |
| GO:0017038 | 0.00 | 5.45 | 1 | 5 | 93 | protein import |
| GO:0000398 | 0.00 | 5.33 | 1 | 5 | 95 | nuclear mRNA splicing, via spliceosome |
| GO:0016071 | 0.00 | 3.71 | 2 | 7 | 191 | mRNA metabolic process |
| GO:0007165 | 0.00 | 3.67 | 2 | 7 | 193 | signal transduction |
| GO:0000375 | 0.01 | 4.89 | 1 | 5 | 103 | RNA splicing, via transesterification reactions |
| GO:0042254 | 0.01 | 2.85 | 4 | 9 | 321 | ribosome biogenesis and assembly |


Hazbun2003: Viable Prey Gene to GO BP Conditional test for over-representation

| GOBPID | Pvalue | OddsRatio | ExpCount | Count | Size | Term |
| GO:0006350 | 0.00 | 1.57 | 159 | 207 | 517 | transcription |
| GO:0006366 | 0.00 | 1.72 | 95 | 131 | 308 | transcription from RNA polymerase II promoter |
| GO:0032774 | 0.00 | 1.54 | 146 | 189 | 476 | RNA biosynthetic process |
| GO:0050794 | 0.00 | 1.44 | 208 | 257 | 678 | regulation of cellular process |
| GO:0065007 | 0.00 | 1.40 | 241 | 291 | 783 | biological regulation |
| GO:0043283 | 0.00 | 1.28 | 553 | 620 | 1800 | biopolymer metabolic process |
| GO:0006355 | 0.00 | 1.59 | 101 | 133 | 327 | regulation of transcription, DNA-dependent |
| GO:0019219 | 0.00 | 1.51 | 122 | 156 | 396 | regulation of nucleobase, nucleoside, nucleotide and nucleic acid metabolic process |
| GO:0051276 | 0.00 | 1.40 | 171 | 209 | 556 | chromosome organization and biogenesis |
| GO:0016043 | 0.00 | 1.23 | 617 | 675 | 2008 | cell organization and biogenesis |
| GO:0019222 | 0.00 | 1.39 | 150 | 183 | 488 | regulation of metabolic process |
| GO:0022402 | 0.00 | 1.42 | 123 | 152 | 399 | cell cycle process |
| GO:0006325 | 0.00 | 1.52 | 73 | 95 | 238 | establishment and/or maintenance of chromatin architecture |
| GO:0000278 | 0.00 | 1.51 | 75 | 97 | 244 | mitotic cell cycle |
| GO:0016569 | 0.00 | 2.01 | 25 | 38 | 81 | covalent chromatin modification |
| GO:0050896 | 0.00 | 1.29 | 219 | 254 | 713 | response to stimulus |
| GO:0045944 | 0.00 | 2.04 | 22 | 34 | 72 | positive regulation of transcription from RNA polymerase II promoter |
| GO:0006396 | 0.00 | 1.39 | 108 | 132 | 350 | RNA processing |
| GO:0000074 | 0.00 | 1.57 | 50 | 66 | 162 | regulation of progression through cell cycle |
| GO:0000003 | 0.01 | 1.38 | 94 | 115 | 306 | reproduction |
| GO:0048518 | 0.01 | 1.65 | 37 | 50 | 119 | positive regulation of biological process |
| GO:0007067 | 0.01 | 1.62 | 38 | 52 | 125 | mitosis |
| GO:0000723 | 0.01 | 1.40 | 83 | 102 | 269 | telomere maintenance |
| GO:0006974 | 0.01 | 1.43 | 69 | 87 | 226 | response to DNA damage stimulus |
| GO:0031325 | 0.01 | 1.67 | 32 | 44 | 104 | positive regulation of cellular metabolic process |
| GO:0045941 | 0.01 | 1.72 | 29 | 40 | 93 | positive regulation of transcription |
| GO:0007017 | 0.01 | 1.68 | 30 | 42 | 99 | microtubule-based process |
| GO:0000279 | 0.01 | 1.39 | 77 | 94 | 249 | M phase |


Hazbun2003: Viable Baits Gene to GO MF Conditional test for over-representation

| GOMFID | Pvalue | OddsRatio | ExpCount | Count | Size | Term |
| GO:0005488 | 0.01 | 2.11 | 11 | 20 | 1056 | binding |


Hazbun2003: Viable Prey Gene to GO MF Conditional test for over-representation

| GOMFID | Pvalue | OddsRatio | ExpCount | Count | Size | Term |
| GO:0016563 | 0.00 | 2.45 | 17 | 28 | 54 | transcriptional activator activity |
| GO:0003702 | 0.00 | 1.73 | 38 | 53 | 123 | RNA polymerase II transcription factor activity |


Hazbun2003: Viable Baits Gene to GO MF Conditional test for under-representation

| GOMFID | Pvalue | OddsRatio | ExpCount | Count | Size | Term |
| GO:0016787 | 0.01 | 0.22 | 8 | 2 | 734 | hydrolase activity |


Hazbun2003: Viable Prey Gene to GO MF Conditional test for under-representation

| GOMFID | Pvalue | OddsRatio | ExpCount | Count | Size | Term |
| GO:0004175 | 0.01 | 0.42 | 18 | 9 | 57 | endopeptidase activity |
