# Supplementary material for: Coverage and error models of protein-protein interaction data by directed graph analysis
Source: Genome Biol. 2007 Sep 10;8(9):R186. doi: 10.1186/gb-2007-8-9-r186 (PMC2375024; doi:10.1186/gb-2007-8-9-r186)
Supplement: Additional data file 2 — Presented is the Bioconductor package ppiStats (version 1.3.5 of 22 June 2007) in 'source' format. ppiStats contains the novel methods developed in this paper. [file gb-2007-8-9-r186-S2.gz › ppiStats/inst/Scripts/Ho2002.html]

Ho2002: Viable Baits Gene to GO CC Conditional test for over-representation

| GOCCID | Pvalue | OddsRatio | ExpCount | Count | Size | Term |
| GO:0005622 | 0.00 | 3.14 | 385 | 449 | 4563 | intracellular |
| GO:0005623 | 0.00 | 3.31 | 419 | 465 | 4954 | cell |
| GO:0005634 | 0.00 | 2.13 | 75 | 128 | 1814 | nucleus |
| GO:0043228 | 0.00 | 1.94 | 79 | 126 | 931 | non-membrane-bound organelle |
| GO:0043229 | 0.00 | 1.71 | 317 | 368 | 3755 | intracellular organelle |
| GO:0005730 | 0.00 | 2.45 | 19 | 40 | 226 | nucleolus |
| GO:0005694 | 0.00 | 2.25 | 19 | 37 | 223 | chromosome |
| GO:0005732 | 0.00 | 3.96 | 5 | 15 | 57 | small nucleolar ribonucleoprotein complex |
| GO:0005935 | 0.00 | 2.73 | 9 | 22 | 112 | bud neck |
| GO:0044422 | 0.00 | 1.42 | 176 | 213 | 2078 | organelle part |
| GO:0005938 | 0.00 | 2.78 | 8 | 20 | 100 | cell cortex |
| GO:0044430 | 0.00 | 2.34 | 13 | 26 | 190 | cytoskeletal part |
| GO:0044428 | 0.00 | 1.56 | 54 | 76 | 931 | nuclear part |
| GO:0043227 | 0.00 | 1.36 | 289 | 322 | 3423 | membrane-bound organelle |
| GO:0015629 | 0.00 | 2.45 | 7 | 14 | 77 | actin cytoskeleton |
| GO:0030863 | 0.01 | 2.68 | 4 | 10 | 51 | cortical cytoskeleton |
| GO:0030532 | 0.01 | 2.52 | 5 | 11 | 59 | small nuclear ribonucleoprotein complex |


Ho2002: Viable Prey Gene to GO CC Conditional test for over-representation

| GOCCID | Pvalue | OddsRatio | ExpCount | Count | Size | Term |
| GO:0005622 | 0.00 | 3.63 | 1016 | 1184 | 4563 | intracellular |
| GO:0005623 | 0.00 | 3.95 | 1103 | 1228 | 4954 | cell |
| GO:0005730 | 0.00 | 4.34 | 50 | 121 | 226 | nucleolus |
| GO:0005634 | 0.00 | 1.94 | 404 | 555 | 1814 | nucleus |
| GO:0043233 | 0.00 | 2.29 | 164 | 270 | 736 | organelle lumen |
| GO:0043228 | 0.00 | 1.84 | 207 | 298 | 931 | non-membrane-bound organelle |
| GO:0043227 | 0.00 | 1.43 | 762 | 847 | 3423 | membrane-bound organelle |
| GO:0044446 | 0.00 | 1.42 | 454 | 537 | 2078 | intracellular organelle part |
| GO:0005732 | 0.00 | 4.24 | 13 | 31 | 57 | small nucleolar ribonucleoprotein complex |
| GO:0005654 | 0.00 | 9.87 | 4 | 14 | 326 | nucleoplasm |
| GO:0043234 | 0.00 | 1.37 | 309 | 370 | 1519 | protein complex |
| GO:0005938 | 0.00 | 2.58 | 22 | 42 | 100 | cell cortex |
| GO:0030863 | 0.00 | 3.14 | 11 | 24 | 51 | cortical cytoskeleton |
| GO:0005935 | 0.00 | 2.13 | 25 | 42 | 112 | bud neck |
| GO:0005737 | 0.00 | 1.26 | 745 | 801 | 3346 | cytoplasm |
| GO:0015629 | 0.00 | 2.48 | 14 | 26 | 77 | actin cytoskeleton |
| GO:0044451 | 0.00 | 1.50 | 62 | 82 | 307 | nucleoplasm part |
| GO:0005934 | 0.01 | 2.16 | 11 | 19 | 50 | bud tip |


Ho2002: Viable Baits Gene to GO BP Conditional test for over-representation

| GOBPID | Pvalue | OddsRatio | ExpCount | Count | Size | Term |
| GO:0006468 | 0.00 | 16.74 | 8 | 54 | 93 | protein amino acid phosphorylation |
| GO:0006796 | 0.00 | 7.56 | 17 | 75 | 199 | phosphate metabolic process |
| GO:0051726 | 0.00 | 7.55 | 14 | 62 | 162 | regulation of cell cycle |
| GO:0065007 | 0.00 | 3.18 | 66 | 148 | 783 | biological regulation |
| GO:0043412 | 0.00 | 3.42 | 48 | 118 | 569 | biopolymer modification |
| GO:0043283 | 0.00 | 2.19 | 104 | 173 | 1800 | biopolymer metabolic process |
| GO:0000075 | 0.00 | 9.38 | 4 | 24 | 53 | cell cycle checkpoint |
| GO:0044267 | 0.00 | 2.09 | 97 | 158 | 1143 | cellular protein metabolic process |
| GO:0051321 | 0.00 | 4.42 | 12 | 39 | 141 | meiotic cell cycle |
| GO:0006139 | 0.00 | 2.19 | 71 | 125 | 1402 | nucleobase, nucleoside, nucleotide and nucleic acid metabolic process |
| GO:0009653 | 0.00 | 3.20 | 21 | 53 | 247 | anatomical structure morphogenesis |
| GO:0007049 | 0.00 | 7.02 | 5 | 23 | 417 | cell cycle |
| GO:0000279 | 0.00 | 4.37 | 10 | 34 | 249 | M phase |
| GO:0000087 | 0.00 | 4.23 | 11 | 34 | 126 | M phase of mitotic cell cycle |
| GO:0008361 | 0.00 | 3.92 | 11 | 33 | 129 | regulation of cell size |
| GO:0048519 | 0.00 | 2.97 | 19 | 47 | 230 | negative regulation of biological process |
| GO:0043632 | 0.00 | 3.52 | 13 | 36 | 153 | modification-dependent macromolecule catabolic process |
| GO:0022414 | 0.00 | 2.74 | 23 | 51 | 267 | reproductive process |
| GO:0040007 | 0.00 | 3.62 | 12 | 33 | 137 | growth |
| GO:0009057 | 0.00 | 2.47 | 27 | 55 | 314 | macromolecule catabolic process |
| GO:0051603 | 0.00 | 3.26 | 13 | 33 | 148 | proteolysis involved in cellular protein catabolic process |
| GO:0030163 | 0.00 | 3.04 | 14 | 36 | 171 | protein catabolic process |
| GO:0007165 | 0.00 | 4.77 | 6 | 20 | 193 | signal transduction |
| GO:0006974 | 0.00 | 6.15 | 4 | 16 | 226 | response to DNA damage stimulus |
| GO:0051325 | 0.00 | 3.97 | 8 | 24 | 92 | interphase |
| GO:0006260 | 0.00 | 3.59 | 9 | 27 | 112 | DNA replication |
| GO:0006996 | 0.00 | 1.77 | 80 | 121 | 1272 | organelle organization and biogenesis |
| GO:0007126 | 0.00 | 3.40 | 10 | 28 | 141 | meiosis |
| GO:0006281 | 0.00 | 3.55 | 8 | 24 | 181 | DNA repair |
| GO:0006396 | 0.00 | 2.15 | 30 | 55 | 350 | RNA processing |
| GO:0007163 | 0.00 | 3.15 | 10 | 25 | 114 | establishment and/or maintenance of cell polarity |
| GO:0006511 | 0.00 | 2.78 | 11 | 27 | 146 | ubiquitin-dependent protein catabolic process |
| GO:0007015 | 0.00 | 3.95 | 5 | 16 | 61 | actin filament organization |
| GO:0006897 | 0.00 | 3.36 | 7 | 19 | 82 | endocytosis |
| GO:0007124 | 0.00 | 3.78 | 5 | 16 | 63 | pseudohyphal growth |
| GO:0051301 | 0.00 | 2.24 | 19 | 37 | 325 | cell division |
| GO:0019222 | 0.00 | 1.80 | 41 | 66 | 488 | regulation of metabolic process |
| GO:0007088 | 0.00 | 6.13 | 2 | 10 | 51 | regulation of mitosis |
| GO:0016072 | 0.00 | 2.39 | 14 | 30 | 176 | rRNA metabolic process |
| GO:0031324 | 0.00 | 2.32 | 15 | 31 | 181 | negative regulation of cellular metabolic process |
| GO:0030029 | 0.00 | 2.79 | 9 | 22 | 110 | actin filament-based process |
| GO:0016481 | 0.00 | 2.42 | 12 | 26 | 146 | negative regulation of transcription |
| GO:0000278 | 0.00 | 2.64 | 10 | 22 | 244 | mitotic cell cycle |
| GO:0006342 | 0.00 | 2.81 | 8 | 18 | 89 | chromatin silencing |
| GO:0031497 | 0.00 | 2.71 | 8 | 19 | 97 | chromatin assembly |
| GO:0042254 | 0.00 | 1.84 | 27 | 45 | 321 | ribosome biogenesis and assembly |
| GO:0000910 | 0.00 | 6.98 | 2 | 7 | 101 | cytokinesis |
| GO:0050794 | 0.00 | 1.65 | 44 | 65 | 678 | regulation of cellular process |
| GO:0032200 | 0.00 | 1.91 | 23 | 39 | 269 | telomere organization and biogenesis |
| GO:0022413 | 0.00 | 2.04 | 18 | 32 | 239 | reproductive process in single-celled organism |
| GO:0022613 | 0.00 | 3.16 | 5 | 14 | 384 | ribonucleoprotein complex biogenesis and assembly |
| GO:0030468 | 0.00 | 4.08 | 3 | 10 | 102 | establishment of cell polarity (sensu Fungi) |
| GO:0040029 | 0.00 | 2.63 | 8 | 18 | 94 | regulation of gene expression, epigenetic |
| GO:0006323 | 0.00 | 1.94 | 20 | 35 | 238 | DNA packaging |
| GO:0044248 | 0.00 | 1.72 | 31 | 49 | 390 | cellular catabolic process |
| GO:0006350 | 0.00 | 1.58 | 44 | 63 | 517 | transcription |
| GO:0007001 | 0.00 | 1.77 | 22 | 35 | 551 | chromosome organization and biogenesis (sensu Eukaryota) |
| GO:0032502 | 0.00 | 2.09 | 12 | 22 | 420 | developmental process |
| GO:0006950 | 0.00 | 1.80 | 20 | 33 | 464 | response to stress |
| GO:0032506 | 0.00 | 2.43 | 7 | 15 | 83 | cytokinetic process |
| GO:0019219 | 0.00 | 1.59 | 33 | 49 | 396 | regulation of nucleobase, nucleoside, nucleotide and nucleic acid metabolic process |
| GO:0032774 | 0.00 | 1.52 | 40 | 56 | 476 | RNA biosynthetic process |
| GO:0007242 | 0.00 | 2.21 | 9 | 17 | 123 | intracellular signaling cascade |
| GO:0031326 | 0.00 | 2.64 | 5 | 12 | 62 | regulation of cellular biosynthetic process |
| GO:0006365 | 0.01 | 2.41 | 7 | 14 | 78 | 35S primary transcript processing |
| GO:0006355 | 0.01 | 1.60 | 28 | 41 | 327 | regulation of transcription, DNA-dependent |
| GO:0000746 | 0.01 | 2.09 | 10 | 18 | 113 | conjugation |
| GO:0019953 | 0.01 | 2.09 | 10 | 18 | 113 | sexual reproduction |
| GO:0006333 | 0.01 | 5.47 | 1 | 5 | 112 | chromatin assembly or disassembly |
| GO:0006406 | 0.01 | 2.54 | 5 | 12 | 64 | mRNA export from nucleus |
| GO:0044238 | 0.01 | 1.31 | 115 | 138 | 2763 | primary metabolic process |
| GO:0051168 | 0.01 | 2.18 | 8 | 16 | 97 | nuclear export |
| GO:0007114 | 0.01 | 2.82 | 4 | 10 | 80 | cell budding |
| GO:0000070 | 0.01 | 2.63 | 5 | 11 | 57 | mitotic sister chromatid segregation |
| GO:0016567 | 0.01 | 3.65 | 2 | 7 | 59 | protein ubiquitination |
| GO:0000282 | 0.01 | 2.49 | 5 | 12 | 65 | bud site selection |
| GO:0030154 | 0.01 | 2.01 | 10 | 18 | 160 | cell differentiation |
| GO:0016043 | 0.01 | 1.39 | 57 | 74 | 2008 | cell organization and biogenesis |
| GO:0042221 | 0.01 | 1.54 | 29 | 42 | 347 | response to chemical stimulus |


Ho2002: Viable Prey Gene to GO BP Conditional test for over-representation

| GOBPID | Pvalue | OddsRatio | ExpCount | Count | Size | Term |
| GO:0006139 | 0.00 | 2.14 | 173 | 274 | 1402 | nucleobase, nucleoside, nucleotide and nucleic acid metabolic process |
| GO:0006996 | 0.00 | 1.97 | 229 | 338 | 1272 | organelle organization and biogenesis |
| GO:0022613 | 0.00 | 2.87 | 71 | 139 | 384 | ribonucleoprotein complex biogenesis and assembly |
| GO:0065007 | 0.00 | 1.92 | 174 | 260 | 783 | biological regulation |
| GO:0043283 | 0.00 | 1.81 | 224 | 317 | 1800 | biopolymer metabolic process |
| GO:0008152 | 0.00 | 1.59 | 548 | 663 | 3064 | metabolic process |
| GO:0006365 | 0.00 | 5.46 | 17 | 47 | 78 | 35S primary transcript processing |
| GO:0042273 | 0.00 | 23.63 | 5 | 20 | 63 | ribosomal large subunit biogenesis and assembly |
| GO:0009056 | 0.00 | 2.05 | 90 | 144 | 404 | catabolic process |
| GO:0050794 | 0.00 | 2.61 | 41 | 77 | 678 | regulation of cellular process |
| GO:0043632 | 0.00 | 2.66 | 34 | 65 | 153 | modification-dependent macromolecule catabolic process |
| GO:0009987 | 0.00 | 1.41 | 729 | 813 | 4342 | cellular process |
| GO:0006403 | 0.00 | 3.34 | 19 | 42 | 87 | RNA localization |
| GO:0006468 | 0.00 | 3.21 | 21 | 44 | 93 | protein amino acid phosphorylation |
| GO:0030163 | 0.00 | 2.38 | 38 | 68 | 171 | protein catabolic process |
| GO:0007049 | 0.00 | 1.79 | 93 | 137 | 417 | cell cycle |
| GO:0009653 | 0.00 | 2.08 | 55 | 90 | 247 | anatomical structure morphogenesis |
| GO:0065003 | 0.00 | 1.95 | 66 | 103 | 295 | macromolecule complex assembly |
| GO:0006796 | 0.00 | 2.18 | 44 | 75 | 199 | phosphate metabolic process |
| GO:0006511 | 0.00 | 2.43 | 33 | 59 | 146 | ubiquitin-dependent protein catabolic process |
| GO:0051603 | 0.00 | 2.38 | 33 | 59 | 148 | proteolysis involved in cellular protein catabolic process |
| GO:0043285 | 0.00 | 2.84 | 22 | 43 | 268 | biopolymer catabolic process |
| GO:0050658 | 0.00 | 3.13 | 17 | 36 | 77 | RNA transport |
| GO:0000087 | 0.00 | 2.43 | 28 | 51 | 126 | M phase of mitotic cell cycle |
| GO:0006406 | 0.00 | 3.34 | 14 | 31 | 64 | mRNA export from nucleus |
| GO:0044267 | 0.00 | 1.41 | 254 | 312 | 1143 | cellular protein metabolic process |
| GO:0006970 | 0.00 | 3.09 | 16 | 33 | 71 | response to osmotic stress |
| GO:0015980 | 0.00 | 2.02 | 44 | 71 | 197 | energy derivation by oxidation of organic compounds |
| GO:0007010 | 0.00 | 1.94 | 49 | 77 | 220 | cytoskeleton organization and biogenesis |
| GO:0000074 | 0.00 | 2.78 | 18 | 36 | 162 | regulation of progression through cell cycle |
| GO:0051168 | 0.00 | 2.93 | 16 | 33 | 97 | nuclear export |
| GO:0006974 | 0.00 | 1.89 | 50 | 78 | 226 | response to DNA damage stimulus |
| GO:0006323 | 0.00 | 1.82 | 53 | 80 | 238 | DNA packaging |
| GO:0006519 | 0.00 | 1.90 | 44 | 69 | 199 | amino acid and derivative metabolic process |
| GO:0015931 | 0.00 | 2.56 | 19 | 36 | 86 | nucleobase, nucleoside, nucleotide and nucleic acid transport |
| GO:0022403 | 0.00 | 1.77 | 53 | 78 | 328 | cell cycle phase |
| GO:0030468 | 0.00 | 2.29 | 23 | 40 | 102 | establishment of cell polarity (sensu Fungi) |
| GO:0007001 | 0.00 | 1.47 | 121 | 157 | 551 | chromosome organization and biogenesis (sensu Eukaryota) |
| GO:0044262 | 0.00 | 1.86 | 42 | 64 | 187 | cellular carbohydrate metabolic process |
| GO:0007163 | 0.00 | 2.15 | 25 | 43 | 114 | establishment and/or maintenance of cell polarity |
| GO:0000910 | 0.00 | 2.23 | 22 | 39 | 101 | cytokinesis |
| GO:0008361 | 0.00 | 2.04 | 29 | 47 | 129 | regulation of cell size |
| GO:0006333 | 0.00 | 2.13 | 25 | 42 | 112 | chromatin assembly or disassembly |
| GO:0006260 | 0.00 | 2.13 | 25 | 42 | 112 | DNA replication |
| GO:0040007 | 0.00 | 1.98 | 30 | 49 | 137 | growth |
| GO:0031323 | 0.00 | 1.47 | 102 | 133 | 459 | regulation of cellular metabolic process |
| GO:0007088 | 0.00 | 4.07 | 6 | 15 | 51 | regulation of mitosis |
| GO:0006364 | 0.00 | 2.97 | 10 | 21 | 166 | rRNA processing |
| GO:0048523 | 0.00 | 1.69 | 49 | 70 | 218 | negative regulation of cellular process |
| GO:0044265 | 0.00 | 1.92 | 29 | 46 | 284 | cellular macromolecule catabolic process |
| GO:0006402 | 0.00 | 2.68 | 11 | 22 | 60 | mRNA catabolic process |
| GO:0016072 | 0.00 | 21.05 | 2 | 6 | 176 | rRNA metabolic process |
| GO:0007114 | 0.00 | 2.74 | 11 | 21 | 80 | cell budding |
| GO:0007154 | 0.00 | 1.64 | 49 | 70 | 222 | cell communication |
| GO:0006350 | 0.00 | 1.41 | 115 | 145 | 517 | transcription |
| GO:0030036 | 0.00 | 2.82 | 10 | 20 | 106 | actin cytoskeleton organization and biogenesis |
| GO:0019752 | 0.00 | 1.54 | 67 | 90 | 307 | carboxylic acid metabolic process |
| GO:0007015 | 0.00 | 2.45 | 14 | 25 | 61 | actin filament organization |
| GO:0009117 | 0.00 | 1.98 | 24 | 39 | 109 | nucleotide metabolic process |
| GO:0043412 | 0.00 | 1.38 | 127 | 157 | 569 | biopolymer modification |
| GO:0006897 | 0.00 | 2.15 | 18 | 31 | 82 | endocytosis |
| GO:0032774 | 0.00 | 1.41 | 106 | 134 | 476 | RNA biosynthetic process |
| GO:0009892 | 0.00 | 1.67 | 43 | 61 | 191 | negative regulation of metabolic process |
| GO:0006807 | 0.00 | 1.57 | 54 | 74 | 242 | nitrogen compound metabolic process |
| GO:0006066 | 0.00 | 1.66 | 36 | 51 | 160 | alcohol metabolic process |
| GO:0016569 | 0.00 | 1.97 | 18 | 29 | 81 | covalent chromatin modification |
| GO:0006913 | 0.00 | 3.24 | 6 | 12 | 122 | nucleocytoplasmic transport |
| GO:0009889 | 0.00 | 2.12 | 14 | 24 | 64 | regulation of biosynthetic process |
| GO:0006950 | 0.00 | 1.61 | 38 | 53 | 464 | response to stress |
| GO:0051649 | 0.00 | 1.33 | 118 | 143 | 530 | establishment of cellular localization |
| GO:0009309 | 0.00 | 1.77 | 25 | 37 | 111 | amine biosynthetic process |
| GO:0007105 | 0.00 | 2.06 | 14 | 24 | 65 | cytokinesis, site selection |
| GO:0019318 | 0.01 | 1.90 | 18 | 29 | 83 | hexose metabolic process |
| GO:0042255 | 0.01 | 2.08 | 14 | 23 | 62 | ribosome assembly |
| GO:0006512 | 0.01 | 1.86 | 18 | 28 | 81 | ubiquitin cycle |
| GO:0051325 | 0.01 | 1.79 | 20 | 31 | 92 | interphase |
| GO:0000075 | 0.01 | 2.13 | 12 | 20 | 53 | cell cycle checkpoint |
| GO:0000003 | 0.01 | 1.39 | 68 | 86 | 306 | reproduction |
| GO:0051169 | 0.01 | 3.51 | 4 | 9 | 115 | nuclear transport |


Ho2002: Viable Baits Gene to GO MF Conditional test for over-representation

| GOMFID | Pvalue | OddsRatio | ExpCount | Count | Size | Term |
| GO:0016772 | 0.00 | 4.81 | 25 | 81 | 295 | transferase activity, transferring phosphorus-containing groups |
| GO:0004674 | 0.00 | 14.71 | 4 | 29 | 70 | protein serine/threonine kinase activity |
| GO:0004672 | 0.00 | 10.70 | 4 | 25 | 128 | protein kinase activity |
| GO:0042578 | 0.00 | 5.93 | 8 | 34 | 101 | phosphoric ester hydrolase activity |
| GO:0003924 | 0.00 | 5.68 | 4 | 18 | 54 | GTPase activity |
| GO:0016817 | 0.00 | 2.07 | 23 | 42 | 276 | hydrolase activity, acting on acid anhydrides |
| GO:0016462 | 0.00 | 2.07 | 23 | 42 | 276 | pyrophosphatase activity |
| GO:0003824 | 0.00 | 1.51 | 98 | 130 | 1907 | catalytic activity |
| GO:0008047 | 0.00 | 3.02 | 5 | 14 | 66 | enzyme activator activity |
| GO:0005515 | 0.00 | 1.63 | 37 | 55 | 443 | protein binding |
| GO:0045182 | 0.00 | 3.05 | 5 | 12 | 56 | translation regulator activity |
| GO:0003676 | 0.00 | 1.74 | 23 | 37 | 505 | nucleic acid binding |
| GO:0003677 | 0.00 | 2.01 | 13 | 24 | 226 | DNA binding |
| GO:0016881 | 0.00 | 3.00 | 4 | 11 | 52 | acid-amino acid ligase activity |
| GO:0005488 | 0.00 | 2.00 | 12 | 22 | 1056 | binding |
| GO:0016787 | 0.00 | 1.53 | 38 | 54 | 734 | hydrolase activity |
| GO:0008094 | 0.01 | 2.87 | 4 | 9 | 51 | DNA-dependent ATPase activity |


Ho2002: Viable Prey Gene to GO MF Conditional test for over-representation

| GOMFID | Pvalue | OddsRatio | ExpCount | Count | Size | Term |
| GO:0003676 | 0.00 | 2.07 | 112 | 179 | 505 | nucleic acid binding |
| GO:0016817 | 0.00 | 2.52 | 61 | 112 | 276 | hydrolase activity, acting on acid anhydrides |
| GO:0003824 | 0.00 | 1.61 | 319 | 413 | 1907 | catalytic activity |
| GO:0016462 | 0.00 | 2.42 | 61 | 108 | 276 | pyrophosphatase activity |
| GO:0005488 | 0.00 | 1.68 | 118 | 166 | 1056 | binding |
| GO:0003924 | 0.00 | 4.12 | 12 | 29 | 54 | GTPase activity |
| GO:0004175 | 0.00 | 3.95 | 13 | 30 | 57 | endopeptidase activity |
| GO:0004386 | 0.00 | 2.78 | 18 | 36 | 82 | helicase activity |
| GO:0016301 | 0.00 | 1.96 | 44 | 70 | 198 | kinase activity |
| GO:0016773 | 0.00 | 1.91 | 38 | 60 | 172 | phosphotransferase activity, alcohol group as acceptor |
| GO:0016791 | 0.00 | 2.34 | 20 | 35 | 88 | phosphoric monoester hydrolase activity |
| GO:0045182 | 0.00 | 2.85 | 12 | 25 | 56 | translation regulator activity |
| GO:0042623 | 0.00 | 1.98 | 30 | 49 | 137 | ATPase activity, coupled |
| GO:0008092 | 0.00 | 2.99 | 11 | 22 | 52 | cytoskeletal protein binding |
| GO:0016616 | 0.00 | 2.48 | 15 | 28 | 68 | oxidoreductase activity, acting on the CH-OH group of donors, NAD or NADP as acceptor |
| GO:0016887 | 0.00 | 2.52 | 13 | 25 | 197 | ATPase activity |
| GO:0031202 | 0.00 | 2.68 | 11 | 22 | 51 | RNA splicing factor activity, transesterification mechanism |
| GO:0016772 | 0.00 | 2.07 | 21 | 35 | 295 | transferase activity, transferring phosphorus-containing groups |
| GO:0016491 | 0.00 | 1.55 | 59 | 80 | 264 | oxidoreductase activity |
| GO:0016874 | 0.00 | 5.02 | 4 | 10 | 128 | ligase activity |
| GO:0008047 | 0.00 | 2.30 | 15 | 26 | 66 | enzyme activator activity |
| GO:0030234 | 0.00 | 1.86 | 27 | 42 | 188 | enzyme regulator activity |
| GO:0016879 | 0.00 | 2.11 | 17 | 28 | 75 | ligase activity, forming carbon-nitrogen bonds |
| GO:0004674 | 0.00 | 2.19 | 13 | 23 | 70 | protein serine/threonine kinase activity |
| GO:0016788 | 0.01 | 1.45 | 53 | 69 | 238 | hydrolase activity, acting on ester bonds |


Ho2002: Viable Baits Gene to GO CC Conditional test for under-representation

| GOCCID | Pvalue | OddsRatio | ExpCount | Count | Size | Term |
| GO:0005739 | 0.00 | 0.46 | 87 | 47 | 1035 | mitochondrion |
| GO:0005783 | 0.00 | 0.38 | 29 | 12 | 343 | endoplasmic reticulum |
| GO:0005740 | 0.00 | 0.34 | 24 | 9 | 288 | mitochondrial envelope |
| GO:0016021 | 0.00 | 0.32 | 23 | 8 | 269 | integral to membrane |
| GO:0005737 | 0.00 | 0.72 | 283 | 246 | 3346 | cytoplasm |
| GO:0005743 | 0.00 | 0.20 | 14 | 3 | 161 | mitochondrial inner membrane |
| GO:0005761 | 0.00 | 0.00 | 7 | 0 | 81 | mitochondrial ribosome |
| GO:0031090 | 0.00 | 0.58 | 52 | 33 | 620 | organelle membrane |
| GO:0031975 | 0.00 | 0.54 | 33 | 19 | 391 | envelope |
| GO:0005840 | 0.00 | 0.52 | 29 | 16 | 339 | ribosome |
| GO:0005759 | 0.01 | 0.13 | 7 | 1 | 163 | mitochondrial matrix |
| GO:0030312 | 0.01 | 0.22 | 8 | 2 | 99 | external encapsulating structure |
| GO:0009277 | 0.01 | 0.22 | 8 | 2 | 99 | cell wall (sensu Fungi) |
| GO:0044455 | 0.01 | 0.22 | 8 | 2 | 98 | mitochondrial membrane part |


Ho2002: Viable Prey Gene to GO CC Conditional test for under-representation

| GOCCID | Pvalue | OddsRatio | ExpCount | Count | Size | Term |
| GO:0005783 | 0.00 | 0.42 | 76 | 38 | 343 | endoplasmic reticulum |
| GO:0016020 | 0.00 | 0.67 | 238 | 182 | 1071 | membrane |
| GO:0005842 | 0.00 | 0.21 | 19 | 5 | 87 | cytosolic large ribosomal subunit (sensu Eukaryota) |
| GO:0005789 | 0.00 | 0.36 | 28 | 12 | 128 | endoplasmic reticulum membrane |
| GO:0030312 | 0.00 | 0.30 | 22 | 8 | 99 | external encapsulating structure |
| GO:0009277 | 0.00 | 0.30 | 22 | 8 | 99 | cell wall (sensu Fungi) |
| GO:0005843 | 0.00 | 0.18 | 14 | 3 | 62 | cytosolic small ribosomal subunit (sensu Eukaryota) |
| GO:0015934 | 0.00 | 0.08 | 10 | 1 | 131 | large ribosomal subunit |
| GO:0000313 | 0.00 | 0.28 | 18 | 6 | 81 | organellar ribosome |
| GO:0031224 | 0.00 | 0.58 | 61 | 40 | 275 | intrinsic to membrane |
| GO:0031301 | 0.00 | 0.29 | 14 | 5 | 64 | integral to organelle membrane |
| GO:0005773 | 0.01 | 0.60 | 43 | 29 | 194 | vacuole |


Ho2002: Viable Baits Gene to GO BP Conditional test for under-representation

| GOBPID | Pvalue | OddsRatio | ExpCount | Count | Size | Term |
| GO:0006811 | 0.00 | 0.00 | 9 | 0 | 110 | ion transport |
| GO:0044249 | 0.00 | 0.52 | 55 | 32 | 841 | cellular biosynthetic process |
| GO:0009308 | 0.00 | 0.29 | 19 | 6 | 221 | amine metabolic process |
| GO:0006732 | 0.00 | 0.16 | 11 | 2 | 135 | coenzyme metabolic process |
| GO:0009060 | 0.00 | 0.00 | 7 | 0 | 80 | aerobic respiration |
| GO:0006519 | 0.00 | 0.33 | 17 | 6 | 199 | amino acid and derivative metabolic process |
| GO:0032787 | 0.00 | 0.18 | 10 | 2 | 121 | monocarboxylic acid metabolic process |
| GO:0019752 | 0.00 | 0.35 | 16 | 6 | 307 | carboxylic acid metabolic process |
| GO:0044271 | 0.00 | 0.20 | 9 | 2 | 111 | nitrogen compound biosynthetic process |
| GO:0006767 | 0.00 | 0.12 | 7 | 1 | 87 | water-soluble vitamin metabolic process |
| GO:0008652 | 0.01 | 0.21 | 9 | 2 | 103 | amino acid biosynthetic process |
| GO:0030001 | 0.01 | 0.00 | 5 | 0 | 57 | metal ion transport |
| GO:0051188 | 0.01 | 0.14 | 7 | 1 | 78 | cofactor biosynthetic process |
| GO:0044255 | 0.01 | 0.45 | 18 | 9 | 219 | cellular lipid metabolic process |
| GO:0009101 | 0.01 | 0.14 | 7 | 1 | 77 | glycoprotein biosynthetic process |


Ho2002: Viable Prey Gene to GO BP Conditional test for under-representation

| GOBPID | Pvalue | OddsRatio | ExpCount | Count | Size | Term |
| GO:0009100 | 0.00 | 0.29 | 17 | 6 | 78 | glycoprotein metabolic process |
| GO:0006486 | 0.00 | 0.31 | 16 | 6 | 72 | protein amino acid glycosylation |
| GO:0044255 | 0.00 | 0.59 | 49 | 32 | 219 | cellular lipid metabolic process |
| GO:0006644 | 0.01 | 0.48 | 20 | 11 | 91 | phospholipid metabolic process |


Ho2002: Viable Baits Gene to GO MF Conditional test for under-representation

| GOMFID | Pvalue | OddsRatio | ExpCount | Count | Size | Term |
| GO:0005215 | 0.00 | 0.09 | 30 | 3 | 408 | transporter activity |
| GO:0016491 | 0.00 | 0.16 | 22 | 4 | 264 | oxidoreductase activity |
| GO:0003735 | 0.00 | 0.15 | 18 | 3 | 216 | structural constituent of ribosome |
| GO:0008324 | 0.00 | 0.00 | 10 | 0 | 125 | cation transporter activity |
| GO:0016757 | 0.00 | 0.11 | 8 | 1 | 97 | transferase activity, transferring glycosyl groups |
| GO:0015078 | 0.01 | 0.00 | 4 | 0 | 54 | hydrogen ion transporter activity |
| GO:0005342 | 0.01 | 0.00 | 4 | 0 | 53 | organic acid transporter activity |


Ho2002: Viable Prey Gene to GO MF Conditional test for under-representation

| GOMFID | Pvalue | OddsRatio | ExpCount | Count | Size | Term |
| GO:0003735 | 0.00 | 0.10 | 48 | 6 | 216 | structural constituent of ribosome |
| GO:0005215 | 0.00 | 0.52 | 79 | 47 | 408 | transporter activity |
| GO:0005342 | 0.00 | 0.21 | 12 | 3 | 53 | organic acid transporter activity |
