# Supplementary material for: Coverage and error models of protein-protein interaction data by directed graph analysis
Source: Genome Biol. 2007 Sep 10;8(9):R186. doi: 10.1186/gb-2007-8-9-r186 (PMC2375024; doi:10.1186/gb-2007-8-9-r186)
Supplement: Additional data file 2 — Presented is the Bioconductor package ppiStats (version 1.3.5 of 22 June 2007) in 'source' format. ppiStats contains the novel methods developed in this paper. [file gb-2007-8-9-r186-S2.gz › ppiStats/inst/Scripts/ItoCore2001.html]

ItoCore2001: Viable Baits Gene to GO CC Conditional test for over-representation

| GOCCID | Pvalue | OddsRatio | ExpCount | Count | Size | Term |
| GO:0044424 | 0.00 | 1.87 | 346 | 384 | 4527 | intracellular part |
| GO:0031965 | 0.00 | 4.50 | 5 | 17 | 64 | nuclear membrane |
| GO:0005643 | 0.00 | 4.81 | 4 | 14 | 50 | nuclear pore |
| GO:0000780 | 0.00 | 3.89 | 4 | 12 | 50 | condensed nuclear chromosome, pericentric region |
| GO:0005634 | 0.00 | 1.43 | 139 | 172 | 1814 | nucleus |
| GO:0000793 | 0.00 | 2.91 | 6 | 16 | 84 | condensed chromosome |
| GO:0044430 | 0.00 | 2.06 | 15 | 27 | 190 | cytoskeletal part |
| GO:0015630 | 0.00 | 2.44 | 8 | 17 | 103 | microtubule cytoskeleton |
| GO:0000775 | 0.00 | 3.01 | 5 | 12 | 61 | chromosome, pericentric region |
| GO:0005623 | 0.00 | 1.55 | 379 | 399 | 4954 | cell |
| GO:0042579 | 0.01 | 2.78 | 4 | 10 | 54 | microbody |


ItoCore2001: Viable Prey Gene to GO CC Conditional test for over-representation

| GOCCID | Pvalue | OddsRatio | ExpCount | Count | Size | Term |
| GO:0005622 | 0.00 | 2.26 | 381 | 430 | 4563 | intracellular |
| GO:0000775 | 0.00 | 4.74 | 5 | 18 | 61 | chromosome, pericentric region |
| GO:0000780 | 0.00 | 5.31 | 4 | 16 | 50 | condensed nuclear chromosome, pericentric region |
| GO:0015630 | 0.00 | 3.46 | 9 | 24 | 103 | microtubule cytoskeleton |
| GO:0005623 | 0.00 | 1.86 | 413 | 442 | 4954 | cell |
| GO:0000793 | 0.00 | 3.30 | 7 | 19 | 84 | condensed chromosome |
| GO:0005768 | 0.00 | 3.03 | 7 | 18 | 85 | endosome |
| GO:0044430 | 0.00 | 2.43 | 10 | 22 | 190 | cytoskeletal part |
| GO:0031965 | 0.00 | 3.14 | 5 | 14 | 64 | nuclear membrane |
| GO:0005634 | 0.00 | 1.37 | 151 | 183 | 1814 | nucleus |
| GO:0005643 | 0.00 | 3.15 | 4 | 11 | 50 | nuclear pore |
| GO:0005737 | 0.01 | 1.27 | 279 | 305 | 3346 | cytoplasm |
| GO:0000922 | 0.01 | 2.48 | 6 | 12 | 66 | spindle pole |


ItoCore2001: Viable Baits Gene to GO BP Conditional test for over-representation

| GOBPID | Pvalue | OddsRatio | ExpCount | Count | Size | Term |
| GO:0016192 | 0.00 | 2.02 | 24 | 44 | 320 | vesicle-mediated transport |
| GO:0051649 | 0.00 | 1.77 | 41 | 64 | 530 | establishment of cellular localization |
| GO:0006468 | 0.00 | 2.77 | 7 | 17 | 93 | protein amino acid phosphorylation |
| GO:0043285 | 0.00 | 1.88 | 21 | 35 | 268 | biopolymer catabolic process |
| GO:0050789 | 0.00 | 4.45 | 2 | 8 | 708 | regulation of biological process |
| GO:0044265 | 0.00 | 1.82 | 22 | 36 | 284 | cellular macromolecule catabolic process |
| GO:0006397 | 0.00 | 2.20 | 11 | 21 | 139 | mRNA processing |
| GO:0007034 | 0.00 | 2.33 | 8 | 17 | 107 | vacuolar transport |
| GO:0006406 | 0.00 | 2.83 | 5 | 12 | 64 | mRNA export from nucleus |
| GO:0016043 | 0.00 | 1.32 | 154 | 180 | 2008 | cell organization and biogenesis |
| GO:0016049 | 0.00 | 2.29 | 8 | 16 | 102 | cell growth |
| GO:0006611 | 0.00 | 2.99 | 4 | 10 | 51 | protein export from nucleus |
| GO:0044238 | 0.01 | 1.29 | 212 | 238 | 2763 | primary metabolic process |
| GO:0050658 | 0.01 | 2.49 | 6 | 13 | 77 | RNA transport |
| GO:0007049 | 0.01 | 1.55 | 32 | 46 | 417 | cell cycle |
| GO:0006508 | 0.01 | 1.88 | 13 | 23 | 174 | proteolysis |
| GO:0050794 | 0.01 | 1.41 | 52 | 68 | 678 | regulation of cellular process |


ItoCore2001: Viable Prey Gene to GO BP Conditional test for over-representation

| GOBPID | Pvalue | OddsRatio | ExpCount | Count | Size | Term |
| GO:0007010 | 0.00 | 2.16 | 18 | 35 | 220 | cytoskeleton organization and biogenesis |
| GO:0065007 | 0.00 | 1.57 | 65 | 92 | 783 | biological regulation |
| GO:0051649 | 0.00 | 1.68 | 44 | 67 | 530 | establishment of cellular localization |
| GO:0016192 | 0.00 | 1.88 | 27 | 45 | 320 | vesicle-mediated transport |
| GO:0006406 | 0.00 | 3.14 | 5 | 14 | 64 | mRNA export from nucleus |
| GO:0040007 | 0.00 | 2.28 | 11 | 23 | 137 | growth |
| GO:0009165 | 0.00 | 3.16 | 5 | 13 | 59 | nucleotide biosynthetic process |
| GO:0050658 | 0.00 | 2.71 | 6 | 15 | 77 | RNA transport |
| GO:0007049 | 0.00 | 1.63 | 35 | 52 | 417 | cell cycle |
| GO:0006403 | 0.00 | 2.53 | 7 | 16 | 87 | RNA localization |
| GO:0006623 | 0.00 | 2.85 | 5 | 13 | 64 | protein targeting to vacuole |
| GO:0008361 | 0.00 | 2.19 | 11 | 21 | 129 | regulation of cell size |
| GO:0006611 | 0.00 | 3.07 | 4 | 11 | 51 | protein export from nucleus |
| GO:0017038 | 0.00 | 2.33 | 8 | 16 | 93 | protein import |
| GO:0045184 | 0.00 | 1.71 | 23 | 36 | 275 | establishment of protein localization |
| GO:0015931 | 0.00 | 2.36 | 7 | 15 | 86 | nucleobase, nucleoside, nucleotide and nucleic acid transport |
| GO:0016043 | 0.01 | 1.29 | 168 | 194 | 2008 | cell organization and biogenesis |
| GO:0000226 | 0.01 | 2.66 | 5 | 11 | 80 | microtubule cytoskeleton organization and biogenesis |
| GO:0043543 | 0.01 | 2.66 | 5 | 11 | 57 | protein amino acid acylation |
| GO:0050794 | 0.01 | 1.41 | 57 | 74 | 678 | regulation of cellular process |
| GO:0006886 | 0.01 | 1.66 | 21 | 33 | 257 | intracellular protein transport |
| GO:0016071 | 0.01 | 1.77 | 16 | 26 | 191 | mRNA metabolic process |


ItoCore2001: Viable Baits Gene to GO MF Conditional test for over-representation

| GOMFID | Pvalue | OddsRatio | ExpCount | Count | Size | Term |
| GO:0016301 | 0.00 | 2.33 | 15 | 31 | 198 | kinase activity |
| GO:0005515 | 0.00 | 1.85 | 34 | 56 | 443 | protein binding |
| GO:0016773 | 0.00 | 2.01 | 13 | 24 | 172 | phosphotransferase activity, alcohol group as acceptor |
| GO:0004674 | 0.00 | 2.83 | 5 | 12 | 70 | protein serine/threonine kinase activity |


ItoCore2001: Viable Prey Gene to GO MF Conditional test for over-representation

| GOMFID | Pvalue | OddsRatio | ExpCount | Count | Size | Term |
| GO:0005200 | 0.00 | 4.24 | 4 | 14 | 51 | structural constituent of cytoskeleton |
| GO:0005515 | 0.00 | 1.84 | 36 | 59 | 443 | protein binding |


ItoCore2001: Viable Baits Gene to GO CC Conditional test for under-representation

| GOCCID | Pvalue | OddsRatio | ExpCount | Count | Size | Term |
| GO:0005739 | 0.00 | 0.41 | 79 | 38 | 1035 | mitochondrion |
| GO:0031966 | 0.00 | 0.34 | 19 | 7 | 247 | mitochondrial membrane |
| GO:0030312 | 0.00 | 0.12 | 8 | 1 | 99 | external encapsulating structure |
| GO:0009277 | 0.00 | 0.12 | 8 | 1 | 99 | cell wall (sensu Fungi) |
| GO:0005842 | 0.01 | 0.14 | 7 | 1 | 87 | cytosolic large ribosomal subunit (sensu Eukaryota) |


ItoCore2001: Viable Prey Gene to GO CC Conditional test for under-representation

| GOCCID | Pvalue | OddsRatio | ExpCount | Count | Size | Term |
| GO:0005739 | 0.00 | 0.56 | 86 | 54 | 1035 | mitochondrion |
| GO:0015934 | 0.00 | 0.25 | 11 | 3 | 131 | large ribosomal subunit |
| GO:0005830 | 0.00 | 0.34 | 14 | 5 | 164 | cytosolic ribosome (sensu Eukaryota) |
| GO:0030529 | 0.01 | 0.61 | 43 | 28 | 516 | ribonucleoprotein complex |
| GO:0005743 | 0.01 | 0.35 | 13 | 5 | 161 | mitochondrial inner membrane |


ItoCore2001: Viable Baits Gene to GO BP Conditional test for under-representation

| GOBPID | Pvalue | OddsRatio | ExpCount | Count | Size | Term |
| GO:0006486 | 0.00 | 0.00 | 6 | 0 | 72 | protein amino acid glycosylation |
| GO:0006412 | 0.01 | 0.53 | 28 | 16 | 372 | translation |
| GO:0006839 | 0.01 | 0.00 | 5 | 0 | 63 | mitochondrial transport |
| GO:0006811 | 0.01 | 0.22 | 8 | 2 | 110 | ion transport |


ItoCore2001: Viable Prey Gene to GO BP Conditional test for under-representation

| GOBPID | Pvalue | OddsRatio | ExpCount | Count | Size | Term |
| GO:0006412 | 0.00 | 0.51 | 31 | 17 | 372 | translation |
| GO:0006812 | 0.00 | 0.12 | 8 | 1 | 92 | cation transport |
| GO:0042254 | 0.00 | 0.49 | 27 | 14 | 321 | ribosome biogenesis and assembly |
| GO:0016072 | 0.01 | 0.38 | 15 | 6 | 176 | rRNA metabolic process |


ItoCore2001: Viable Baits Gene to GO MF Conditional test for under-representation

| GOMFID | Pvalue | OddsRatio | ExpCount | Count | Size | Term |
| GO:0005215 | 0.00 | 0.41 | 31 | 14 | 408 | transporter activity |
| GO:0003735 | 0.00 | 0.39 | 17 | 7 | 216 | structural constituent of ribosome |


ItoCore2001: Viable Prey Gene to GO MF Conditional test for under-representation

| GOMFID | Pvalue | OddsRatio | ExpCount | Count | Size | Term |
| GO:0005215 | 0.00 | 0.43 | 30 | 14 | 408 | transporter activity |
| GO:0003735 | 0.00 | 0.36 | 18 | 7 | 216 | structural constituent of ribosome |
| GO:0016853 | 0.01 | 0.00 | 5 | 0 | 56 | isomerase activity |
| GO:0005342 | 0.01 | 0.00 | 4 | 0 | 53 | organic acid transporter activity |
