# Supplementary material for: Coverage and error models of protein-protein interaction data by directed graph analysis
Source: Genome Biol. 2007 Sep 10;8(9):R186. doi: 10.1186/gb-2007-8-9-r186 (PMC2375024; doi:10.1186/gb-2007-8-9-r186)
Supplement: Additional data file 2 — Presented is the Bioconductor package ppiStats (version 1.3.5 of 22 June 2007) in 'source' format. ppiStats contains the novel methods developed in this paper. [file gb-2007-8-9-r186-S2.gz › ppiStats/inst/Scripts/ItoFull2001.html]

ItoFull2001: Viable Baits Gene to GO CC Conditional test for over-representation

| GOCCID | Pvalue | OddsRatio | ExpCount | Count | Size | Term |
| GO:0005622 | 0.00 | 1.46 | 1128 | 1193 | 4563 | intracellular |
| GO:0005623 | 0.00 | 1.56 | 1225 | 1280 | 4954 | cell |
| GO:0031965 | 0.00 | 2.90 | 16 | 31 | 64 | nuclear membrane |
| GO:0005643 | 0.00 | 2.84 | 12 | 24 | 50 | nuclear pore |
| GO:0012505 | 0.00 | 1.54 | 73 | 98 | 296 | endomembrane system |
| GO:0005816 | 0.00 | 2.28 | 15 | 26 | 61 | spindle pole body |
| GO:0015630 | 0.00 | 1.88 | 25 | 39 | 103 | microtubule cytoskeleton |
| GO:0043227 | 0.00 | 1.20 | 846 | 893 | 3423 | membrane-bound organelle |
| GO:0005783 | 0.00 | 1.39 | 85 | 106 | 343 | endoplasmic reticulum |
| GO:0030427 | 0.01 | 1.61 | 37 | 51 | 149 | site of polarized growth |
| GO:0005634 | 0.01 | 1.18 | 449 | 488 | 1814 | nucleus |
| GO:0005737 | 0.01 | 1.17 | 827 | 869 | 3346 | cytoplasm |
| GO:0044431 | 0.01 | 1.63 | 33 | 46 | 133 | Golgi apparatus part |
| GO:0030135 | 0.01 | 1.82 | 19 | 29 | 78 | coated vesicle |


ItoFull2001: Viable Prey Gene to GO CC Conditional test for over-representation

| GOCCID | Pvalue | OddsRatio | ExpCount | Count | Size | Term |
| GO:0005622 | 0.00 | 1.53 | 1781 | 1878 | 4563 | intracellular |
| GO:0005623 | 0.00 | 1.65 | 1934 | 2017 | 4954 | cell |
| GO:0043227 | 0.00 | 1.25 | 1336 | 1410 | 3423 | membrane-bound organelle |
| GO:0043229 | 0.00 | 1.23 | 1466 | 1532 | 3755 | intracellular organelle |
| GO:0005737 | 0.00 | 1.22 | 1306 | 1374 | 3346 | cytoplasm |
| GO:0000776 | 0.00 | 2.68 | 21 | 34 | 54 | kinetochore |
| GO:0005634 | 0.00 | 1.22 | 708 | 767 | 1814 | nucleus |
| GO:0000922 | 0.00 | 2.14 | 26 | 38 | 66 | spindle pole |
| GO:0015630 | 0.00 | 1.81 | 40 | 55 | 103 | microtubule cytoskeleton |
| GO:0000779 | 0.00 | 2.36 | 20 | 30 | 50 | condensed chromosome, pericentric region |


ItoFull2001: Viable Baits Gene to GO BP Conditional test for over-representation

| GOBPID | Pvalue | OddsRatio | ExpCount | Count | Size | Term |
| GO:0051641 | 0.00 | 1.52 | 138 | 181 | 559 | cellular localization |
| GO:0016192 | 0.00 | 1.64 | 79 | 110 | 320 | vesicle-mediated transport |
| GO:0009987 | 0.00 | 1.32 | 1064 | 1120 | 4342 | cellular process |
| GO:0006888 | 0.00 | 2.39 | 19 | 34 | 78 | ER to Golgi vesicle-mediated transport |
| GO:0046903 | 0.00 | 1.66 | 61 | 85 | 245 | secretion |
| GO:0046907 | 0.00 | 1.51 | 54 | 72 | 520 | intracellular transport |
| GO:0007034 | 0.00 | 1.77 | 26 | 39 | 107 | vacuolar transport |
| GO:0006402 | 0.01 | 2.05 | 15 | 24 | 60 | mRNA catabolic process |
| GO:0006611 | 0.01 | 2.15 | 13 | 21 | 51 | protein export from nucleus |
| GO:0006886 | 0.01 | 1.43 | 64 | 81 | 257 | intracellular protein transport |
| GO:0006403 | 0.01 | 1.79 | 22 | 32 | 87 | RNA localization |


ItoFull2001: Viable Prey Gene to GO BP Conditional test for over-representation

| GOBPID | Pvalue | OddsRatio | ExpCount | Count | Size | Term |
| GO:0009987 | 0.00 | 1.25 | 1695 | 1752 | 4342 | cellular process |
| GO:0015980 | 0.00 | 1.51 | 77 | 96 | 197 | energy derivation by oxidation of organic compounds |
| GO:0007088 | 0.01 | 2.07 | 20 | 29 | 51 | regulation of mitosis |


ItoFull2001: Viable Baits Gene to GO MF Conditional test for over-representation

| GOMFID | Pvalue | OddsRatio | ExpCount | Count | Size | Term |
| GO:0005515 | 0.00 | 1.40 | 110 | 137 | 443 | protein binding |
| GO:0016301 | 0.01 | 1.47 | 49 | 64 | 198 | kinase activity |


ItoFull2001: Viable Prey Gene to GO MF Conditional test for over-representation

| GOMFID | Pvalue | OddsRatio | ExpCount | Count | Size | Term |
| GO:0030234 | 0.01 | 1.48 | 73 | 91 | 188 | enzyme regulator activity |


ItoFull2001: Viable Baits Gene to GO CC Conditional test for under-representation

| GOCCID | Pvalue | OddsRatio | ExpCount | Count | Size | Term |
| GO:0005842 | 0.00 | 0.30 | 22 | 8 | 87 | cytosolic large ribosomal subunit (sensu Eukaryota) |
| GO:0005739 | 0.00 | 0.79 | 256 | 220 | 1035 | mitochondrion |
| GO:0005740 | 0.01 | 0.69 | 71 | 54 | 288 | mitochondrial envelope |


ItoFull2001: Viable Prey Gene to GO CC Conditional test for under-representation

| GOCCID | Pvalue | OddsRatio | ExpCount | Count | Size | Term |
| GO:0005840 | 0.00 | 0.71 | 132 | 107 | 339 | ribosome |
| GO:0005843 | 0.00 | 0.45 | 24 | 14 | 62 | cytosolic small ribosomal subunit (sensu Eukaryota) |


ItoFull2001: Viable Baits Gene to GO BP Conditional test for under-representation

| GOBPID | Pvalue | OddsRatio | ExpCount | Count | Size | Term |
| GO:0045333 | 0.01 | 0.45 | 21 | 11 | 85 | cellular respiration |


ItoFull2001: Viable Baits Gene to GO MF Conditional test for under-representation

| GOMFID | Pvalue | OddsRatio | ExpCount | Count | Size | Term |
| GO:0003735 | 0.00 | 0.60 | 53 | 36 | 216 | structural constituent of ribosome |
| GO:0030528 | 0.01 | 0.69 | 79 | 60 | 320 | transcription regulator activity |


ItoFull2001: Viable Prey Gene to GO MF Conditional test for under-representation

| GOMFID | Pvalue | OddsRatio | ExpCount | Count | Size | Term |
| GO:0016887 | 0.00 | 0.61 | 77 | 56 | 197 | ATPase activity |
| GO:0003735 | 0.00 | 0.65 | 84 | 64 | 216 | structural constituent of ribosome |
| GO:0003677 | 0.00 | 0.68 | 88 | 69 | 226 | DNA binding |
| GO:0008094 | 0.01 | 0.43 | 20 | 11 | 51 | DNA-dependent ATPase activity |
| GO:0004386 | 0.01 | 0.53 | 32 | 21 | 82 | helicase activity |
