# Supplementary material for: Coverage and error models of protein-protein interaction data by directed graph analysis
Source: Genome Biol. 2007 Sep 10;8(9):R186. doi: 10.1186/gb-2007-8-9-r186 (PMC2375024; doi:10.1186/gb-2007-8-9-r186)
Supplement: Additional data file 2 — Presented is the Bioconductor package ppiStats (version 1.3.5 of 22 June 2007) in 'source' format. ppiStats contains the novel methods developed in this paper. [file gb-2007-8-9-r186-S2.gz › ppiStats/inst/Scripts/Krogan2004.html]

Krogan2004: Viable Baits Gene to GO CC Conditional test for over-representation

| GOCCID | Pvalue | OddsRatio | ExpCount | Count | Size | Term |
| GO:0005634 | 0.00 | 6.57 | 48 | 113 | 1814 | nucleus |
| GO:0043233 | 0.00 | 7.07 | 19 | 74 | 736 | organelle lumen |
| GO:0005730 | 0.00 | 10.42 | 6 | 40 | 226 | nucleolus |
| GO:0005622 | 0.00 | Inf | 120 | 153 | 4563 | intracellular |
| GO:0044446 | 0.00 | 3.12 | 55 | 96 | 2078 | intracellular organelle part |
| GO:0005623 | 0.00 | Inf | 130 | 153 | 4954 | cell |
| GO:0005732 | 0.00 | 13.15 | 2 | 14 | 57 | small nucleolar ribonucleoprotein complex |
| GO:0043234 | 0.00 | 2.88 | 38 | 73 | 1519 | protein complex |
| GO:0044451 | 0.00 | 4.30 | 7 | 26 | 307 | nucleoplasm part |
| GO:0043228 | 0.00 | 2.72 | 25 | 51 | 931 | non-membrane-bound organelle |
| GO:0043227 | 0.00 | 2.49 | 90 | 119 | 3423 | membrane-bound organelle |
| GO:0005654 | 0.00 | 13.62 | 1 | 5 | 326 | nucleoplasm |
| GO:0030532 | 0.01 | 5.60 | 1 | 4 | 59 | small nuclear ribonucleoprotein complex |


Krogan2004: Viable Prey Gene to GO CC Conditional test for over-representation

| GOCCID | Pvalue | OddsRatio | ExpCount | Count | Size | Term |
| GO:0043233 | 0.00 | 4.55 | 61 | 168 | 736 | organelle lumen |
| GO:0005634 | 0.00 | 3.60 | 149 | 283 | 1814 | nucleus |
| GO:0005730 | 0.00 | 7.63 | 19 | 83 | 226 | nucleolus |
| GO:0005622 | 0.00 | 7.24 | 375 | 459 | 4563 | intracellular |
| GO:0044446 | 0.00 | 2.89 | 171 | 284 | 2078 | intracellular organelle part |
| GO:0043228 | 0.00 | 2.88 | 77 | 157 | 931 | non-membrane-bound organelle |
| GO:0005623 | 0.00 | 7.32 | 407 | 466 | 4954 | cell |
| GO:0044451 | 0.00 | 3.24 | 21 | 55 | 307 | nucleoplasm part |
| GO:0005732 | 0.00 | 8.25 | 4 | 22 | 57 | small nucleolar ribonucleoprotein complex |
| GO:0005654 | 0.00 | 12.64 | 2 | 10 | 326 | nucleoplasm |
| GO:0005840 | 0.00 | 2.20 | 28 | 53 | 339 | ribosome |
| GO:0043227 | 0.00 | 1.57 | 282 | 327 | 3423 | membrane-bound organelle |
| GO:0030529 | 0.00 | 4.36 | 5 | 16 | 516 | ribonucleoprotein complex |
| GO:0005681 | 0.00 | 3.90 | 6 | 17 | 78 | spliceosome |
| GO:0005843 | 0.00 | 3.32 | 5 | 14 | 62 | cytosolic small ribosomal subunit (sensu Eukaryota) |
| GO:0005842 | 0.00 | 2.77 | 7 | 17 | 87 | cytosolic large ribosomal subunit (sensu Eukaryota) |
| GO:0005829 | 0.00 | 1.74 | 27 | 43 | 330 | cytosol |
| GO:0043234 | 0.00 | 1.45 | 72 | 95 | 1519 | protein complex |


Krogan2004: Viable Baits Gene to GO BP Conditional test for over-representation

| GOBPID | Pvalue | OddsRatio | ExpCount | Count | Size | Term |
| GO:0044238 | 0.00 | 5.88 | 73 | 128 | 2763 | primary metabolic process |
| GO:0043170 | 0.00 | 4.02 | 54 | 104 | 2355 | macromolecule metabolic process |
| GO:0044237 | 0.00 | 3.82 | 77 | 121 | 2988 | cellular metabolic process |
| GO:0016070 | 0.00 | 4.14 | 14 | 43 | 918 | RNA metabolic process |
| GO:0006365 | 0.00 | 11.47 | 2 | 17 | 78 | 35S primary transcript processing |
| GO:0042254 | 0.00 | 7.57 | 3 | 16 | 321 | ribosome biogenesis and assembly |
| GO:0006402 | 0.00 | 15.15 | 1 | 10 | 60 | mRNA catabolic process |
| GO:0043285 | 0.00 | 4.35 | 7 | 25 | 268 | biopolymer catabolic process |
| GO:0065003 | 0.00 | 3.50 | 8 | 23 | 295 | macromolecule complex assembly |
| GO:0006396 | 0.00 | 4.41 | 4 | 16 | 350 | RNA processing |
| GO:0000398 | 0.00 | 5.72 | 3 | 12 | 95 | nuclear mRNA splicing, via spliceosome |
| GO:0051168 | 0.00 | 5.58 | 3 | 12 | 97 | nuclear export |
| GO:0006351 | 0.00 | 2.90 | 11 | 27 | 471 | transcription, DNA-dependent |
| GO:0000375 | 0.00 | 5.21 | 3 | 12 | 103 | RNA splicing, via transesterification reactions |
| GO:0009056 | 0.00 | 2.86 | 11 | 26 | 404 | catabolic process |
| GO:0016568 | 0.00 | 3.56 | 6 | 17 | 209 | chromatin modification |
| GO:0022613 | 0.00 | 6.49 | 2 | 9 | 384 | ribonucleoprotein complex biogenesis and assembly |
| GO:0042273 | 0.00 | 6.49 | 2 | 9 | 63 | ribosomal large subunit biogenesis and assembly |
| GO:0044265 | 0.00 | 4.36 | 3 | 13 | 284 | cellular macromolecule catabolic process |
| GO:0006406 | 0.00 | 6.37 | 2 | 9 | 64 | mRNA export from nucleus |
| GO:0000723 | 0.00 | 3.07 | 7 | 19 | 269 | telomere maintenance |
| GO:0006364 | 0.00 | 6.19 | 2 | 8 | 166 | rRNA processing |
| GO:0006323 | 0.00 | 3.08 | 6 | 17 | 238 | DNA packaging |
| GO:0016071 | 0.00 | 3.96 | 3 | 12 | 191 | mRNA metabolic process |
| GO:0050658 | 0.00 | 5.14 | 2 | 9 | 77 | RNA transport |
| GO:0007001 | 0.00 | 2.30 | 15 | 29 | 551 | chromosome organization and biogenesis (sensu Eukaryota) |
| GO:0006730 | 0.00 | 5.98 | 1 | 7 | 52 | one-carbon compound metabolic process |
| GO:0015931 | 0.00 | 4.53 | 2 | 9 | 86 | nucleobase, nucleoside, nucleotide and nucleic acid transport |
| GO:0006403 | 0.00 | 4.47 | 2 | 9 | 87 | RNA localization |
| GO:0043632 | 0.00 | 3.01 | 4 | 11 | 153 | modification-dependent macromolecule catabolic process |
| GO:0051325 | 0.00 | 3.66 | 2 | 8 | 92 | interphase |
| GO:0042255 | 0.01 | 4.08 | 2 | 6 | 62 | ribosome assembly |
| GO:0006310 | 0.01 | 3.01 | 3 | 8 | 110 | DNA recombination |


Krogan2004: Viable Prey Gene to GO BP Conditional test for over-representation

| GOBPID | Pvalue | OddsRatio | ExpCount | Count | Size | Term |
| GO:0006139 | 0.00 | 4.62 | 44 | 131 | 1402 | nucleobase, nucleoside, nucleotide and nucleic acid metabolic process |
| GO:0006365 | 0.00 | 16.70 | 6 | 45 | 78 | 35S primary transcript processing |
| GO:0043283 | 0.00 | 2.88 | 88 | 175 | 1800 | biopolymer metabolic process |
| GO:0065003 | 0.00 | 4.69 | 24 | 79 | 295 | macromolecule complex assembly |
| GO:0008152 | 0.00 | 2.44 | 219 | 314 | 3064 | metabolic process |
| GO:0032774 | 0.00 | 3.23 | 39 | 95 | 476 | RNA biosynthetic process |
| GO:0022613 | 0.00 | 10.76 | 5 | 30 | 384 | ribonucleoprotein complex biogenesis and assembly |
| GO:0006350 | 0.00 | 2.98 | 43 | 97 | 517 | transcription |
| GO:0042255 | 0.00 | 8.47 | 5 | 26 | 62 | ribosome assembly |
| GO:0006396 | 0.00 | 4.14 | 15 | 47 | 350 | RNA processing |
| GO:0009987 | 0.00 | 2.15 | 301 | 371 | 4342 | cellular process |
| GO:0009056 | 0.00 | 2.83 | 33 | 75 | 404 | catabolic process |
| GO:0016071 | 0.00 | 4.68 | 11 | 37 | 191 | mRNA metabolic process |
| GO:0043285 | 0.00 | 5.54 | 8 | 31 | 268 | biopolymer catabolic process |
| GO:0043632 | 0.00 | 4.22 | 13 | 40 | 153 | modification-dependent macromolecule catabolic process |
| GO:0006402 | 0.00 | 13.26 | 2 | 15 | 60 | mRNA catabolic process |
| GO:0044260 | 0.00 | 1.90 | 98 | 151 | 1192 | cellular macromolecule metabolic process |
| GO:0030163 | 0.00 | 3.50 | 14 | 39 | 171 | protein catabolic process |
| GO:0006511 | 0.00 | 3.72 | 12 | 35 | 146 | ubiquitin-dependent protein catabolic process |
| GO:0051603 | 0.00 | 3.65 | 12 | 35 | 148 | proteolysis involved in cellular protein catabolic process |
| GO:0042254 | 0.00 | 4.42 | 8 | 28 | 321 | ribosome biogenesis and assembly |
| GO:0000375 | 0.00 | 4.14 | 8 | 27 | 103 | RNA splicing, via transesterification reactions |
| GO:0051168 | 0.00 | 4.26 | 8 | 26 | 97 | nuclear export |
| GO:0006364 | 0.00 | 5.18 | 5 | 18 | 166 | rRNA processing |
| GO:0006406 | 0.00 | 4.87 | 5 | 19 | 64 | mRNA export from nucleus |
| GO:0044265 | 0.00 | 3.16 | 11 | 28 | 284 | cellular macromolecule catabolic process |
| GO:0050658 | 0.00 | 4.04 | 6 | 20 | 77 | RNA transport |
| GO:0006325 | 0.00 | 3.17 | 10 | 27 | 238 | establishment and/or maintenance of chromatin architecture |
| GO:0051276 | 0.00 | 1.91 | 46 | 76 | 556 | chromosome organization and biogenesis |
| GO:0006403 | 0.00 | 3.67 | 7 | 21 | 87 | RNA localization |
| GO:0000398 | 0.00 | 3.83 | 6 | 19 | 95 | nuclear mRNA splicing, via spliceosome |
| GO:0015931 | 0.00 | 3.49 | 7 | 20 | 86 | nucleobase, nucleoside, nucleotide and nucleic acid transport |
| GO:0000278 | 0.00 | 2.22 | 20 | 39 | 244 | mitotic cell cycle |
| GO:0042273 | 0.00 | 7.29 | 2 | 9 | 63 | ribosomal large subunit biogenesis and assembly |
| GO:0031326 | 0.00 | 3.65 | 5 | 15 | 62 | regulation of cellular biosynthetic process |
| GO:0006259 | 0.00 | 1.76 | 41 | 65 | 503 | DNA metabolic process |
| GO:0051246 | 0.00 | 3.42 | 5 | 15 | 65 | regulation of protein metabolic process |
| GO:0006412 | 0.00 | 1.78 | 27 | 44 | 372 | translation |
| GO:0051325 | 0.00 | 2.79 | 5 | 13 | 92 | interphase |
| GO:0006366 | 0.00 | 1.76 | 22 | 36 | 308 | transcription from RNA polymerase II promoter |
| GO:0019219 | 0.00 | 1.60 | 33 | 48 | 396 | regulation of nucleobase, nucleoside, nucleotide and nucleic acid metabolic process |
| GO:0050794 | 0.00 | 1.46 | 56 | 75 | 678 | regulation of cellular process |
| GO:0019538 | 0.01 | 1.37 | 79 | 100 | 1194 | protein metabolic process |
| GO:0016569 | 0.01 | 2.37 | 7 | 14 | 81 | covalent chromatin modification |
| GO:0000723 | 0.01 | 1.66 | 22 | 34 | 269 | telomere maintenance |
| GO:0006399 | 0.01 | 2.10 | 9 | 17 | 113 | tRNA metabolic process |
| GO:0006338 | 0.01 | 2.00 | 10 | 19 | 149 | chromatin remodeling |
| GO:0006730 | 0.01 | 2.69 | 4 | 10 | 52 | one-carbon compound metabolic process |
| GO:0006333 | 0.01 | 2.03 | 9 | 17 | 112 | chromatin assembly or disassembly |


Krogan2004: Viable Baits Gene to GO MF Conditional test for over-representation

| GOMFID | Pvalue | OddsRatio | ExpCount | Count | Size | Term |
| GO:0005488 | 0.00 | 2.53 | 28 | 54 | 1056 | binding |
| GO:0003723 | 0.00 | 4.21 | 4 | 15 | 236 | RNA binding |
| GO:0016887 | 0.00 | 3.26 | 5 | 15 | 197 | ATPase activity |
| GO:0004518 | 0.00 | 4.32 | 3 | 10 | 100 | nuclease activity |
| GO:0031202 | 0.00 | 6.11 | 1 | 7 | 51 | RNA splicing factor activity, transesterification mechanism |
| GO:0045182 | 0.00 | 5.48 | 1 | 7 | 56 | translation regulator activity |
| GO:0008094 | 0.00 | 5.08 | 1 | 6 | 51 | DNA-dependent ATPase activity |
| GO:0016817 | 0.00 | 2.42 | 7 | 16 | 276 | hydrolase activity, acting on acid anhydrides |
| GO:0016462 | 0.00 | 2.42 | 7 | 16 | 276 | pyrophosphatase activity |
| GO:0004386 | 0.01 | 3.56 | 2 | 7 | 82 | helicase activity |


Krogan2004: Viable Prey Gene to GO MF Conditional test for over-representation

| GOMFID | Pvalue | OddsRatio | ExpCount | Count | Size | Term |
| GO:0045182 | 0.00 | 10.15 | 5 | 26 | 56 | translation regulator activity |
| GO:0004175 | 0.00 | 8.47 | 5 | 24 | 57 | endopeptidase activity |
| GO:0003723 | 0.00 | 4.41 | 12 | 39 | 236 | RNA binding |
| GO:0016817 | 0.00 | 2.71 | 23 | 51 | 276 | hydrolase activity, acting on acid anhydrides |
| GO:0016462 | 0.00 | 2.71 | 23 | 51 | 276 | pyrophosphatase activity |
| GO:0004518 | 0.00 | 3.65 | 8 | 24 | 100 | nuclease activity |
| GO:0008094 | 0.00 | 5.23 | 4 | 16 | 51 | DNA-dependent ATPase activity |
| GO:0016772 | 0.00 | 2.17 | 24 | 46 | 295 | transferase activity, transferring phosphorus-containing groups |
| GO:0031202 | 0.00 | 4.31 | 4 | 14 | 51 | RNA splicing factor activity, transesterification mechanism |
| GO:0003676 | 0.00 | 2.03 | 22 | 40 | 505 | nucleic acid binding |
| GO:0016887 | 0.00 | 3.14 | 5 | 13 | 197 | ATPase activity |
| GO:0042623 | 0.00 | 2.60 | 7 | 16 | 137 | ATPase activity, coupled |
| GO:0016787 | 0.01 | 1.51 | 38 | 53 | 734 | hydrolase activity |


Krogan2004: Viable Baits Gene to GO CC Conditional test for under-representation

| GOCCID | Pvalue | OddsRatio | ExpCount | Count | Size | Term |
| GO:0044425 | 0.00 | 0.11 | 16 | 2 | 611 | membrane part |
| GO:0005737 | 0.00 | 0.54 | 88 | 65 | 3346 | cytoplasm |
| GO:0005783 | 0.00 | 0.10 | 9 | 1 | 343 | endoplasmic reticulum |
| GO:0005886 | 0.00 | 0.00 | 7 | 0 | 255 | plasma membrane |
| GO:0031966 | 0.00 | 0.00 | 7 | 0 | 247 | mitochondrial membrane |
| GO:0031967 | 0.00 | 0.18 | 10 | 2 | 391 | organelle envelope |
| GO:0031090 | 0.00 | 0.19 | 10 | 2 | 620 | organelle membrane |
| GO:0005773 | 0.01 | 0.00 | 5 | 0 | 194 | vacuole |
| GO:0016021 | 0.01 | 0.13 | 7 | 1 | 269 | integral to membrane |
| GO:0044429 | 0.01 | 0.00 | 5 | 0 | 438 | mitochondrial part |
| GO:0044430 | 0.01 | 0.00 | 5 | 0 | 190 | cytoskeletal part |


Krogan2004: Viable Prey Gene to GO CC Conditional test for under-representation

| GOCCID | Pvalue | OddsRatio | ExpCount | Count | Size | Term |
| GO:0005783 | 0.00 | 0.16 | 28 | 5 | 343 | endoplasmic reticulum |
| GO:0005886 | 0.00 | 0.21 | 21 | 5 | 255 | plasma membrane |
| GO:0044425 | 0.00 | 0.41 | 40 | 18 | 611 | membrane part |
| GO:0005740 | 0.00 | 0.31 | 24 | 8 | 288 | mitochondrial envelope |
| GO:0005737 | 0.00 | 0.70 | 275 | 236 | 3346 | cytoplasm |
| GO:0031410 | 0.00 | 0.00 | 8 | 0 | 102 | cytoplasmic vesicle |
| GO:0031988 | 0.00 | 0.00 | 8 | 0 | 102 | membrane-bound vesicle |
| GO:0031090 | 0.00 | 0.47 | 40 | 21 | 620 | organelle membrane |
| GO:0016021 | 0.00 | 0.33 | 22 | 8 | 269 | integral to membrane |
| GO:0031975 | 0.00 | 0.46 | 32 | 16 | 391 | envelope |
| GO:0005739 | 0.00 | 0.64 | 85 | 60 | 1035 | mitochondrion |
| GO:0030135 | 0.00 | 0.00 | 6 | 0 | 78 | coated vesicle |
| GO:0031980 | 0.00 | 0.27 | 13 | 4 | 163 | mitochondrial lumen |
| GO:0030312 | 0.00 | 0.11 | 8 | 1 | 99 | external encapsulating structure |
| GO:0009277 | 0.00 | 0.11 | 8 | 1 | 99 | cell wall (sensu Fungi) |
| GO:0005794 | 0.00 | 0.32 | 15 | 5 | 178 | Golgi apparatus |
| GO:0005798 | 0.00 | 0.00 | 5 | 0 | 65 | Golgi-associated vesicle |
| GO:0000324 | 0.00 | 0.30 | 13 | 4 | 152 | vacuole (sensu Fungi) |
| GO:0005789 | 0.01 | 0.26 | 11 | 3 | 128 | endoplasmic reticulum membrane |
| GO:0005743 | 0.01 | 0.35 | 13 | 5 | 161 | mitochondrial inner membrane |
| GO:0005773 | 0.01 | 0.41 | 16 | 7 | 194 | vacuole |
| GO:0005761 | 0.01 | 0.14 | 7 | 1 | 81 | mitochondrial ribosome |
| GO:0005777 | 0.01 | 0.00 | 4 | 0 | 54 | peroxisome |
| GO:0000776 | 0.01 | 0.00 | 4 | 0 | 54 | kinetochore |


Krogan2004: Viable Baits Gene to GO BP Conditional test for under-representation

| GOBPID | Pvalue | OddsRatio | ExpCount | Count | Size | Term |
| GO:0016192 | 0.00 | 0.00 | 8 | 0 | 320 | vesicle-mediated transport |
| GO:0045045 | 0.00 | 0.00 | 6 | 0 | 238 | secretory pathway |
| GO:0015980 | 0.00 | 0.00 | 5 | 0 | 197 | energy derivation by oxidation of organic compounds |
| GO:0044262 | 0.01 | 0.00 | 5 | 0 | 187 | cellular carbohydrate metabolic process |
| GO:0000279 | 0.01 | 0.14 | 7 | 1 | 249 | M phase |
| GO:0016044 | 0.01 | 0.00 | 5 | 0 | 173 | membrane organization and biogenesis |


Krogan2004: Viable Prey Gene to GO BP Conditional test for under-representation

| GOBPID | Pvalue | OddsRatio | ExpCount | Count | Size | Term |
| GO:0007047 | 0.00 | 0.17 | 16 | 3 | 194 | cell wall organization and biogenesis |
| GO:0006811 | 0.00 | 0.00 | 9 | 0 | 110 | ion transport |
| GO:0048193 | 0.00 | 0.21 | 13 | 3 | 161 | Golgi vesicle transport |
| GO:0045045 | 0.00 | 0.33 | 20 | 7 | 238 | secretory pathway |
| GO:0006629 | 0.00 | 0.34 | 19 | 7 | 232 | lipid metabolic process |
| GO:0009100 | 0.00 | 0.00 | 6 | 0 | 78 | glycoprotein metabolic process |
| GO:0019752 | 0.00 | 0.44 | 25 | 12 | 307 | carboxylic acid metabolic process |
| GO:0006486 | 0.00 | 0.00 | 6 | 0 | 72 | protein amino acid glycosylation |
| GO:0006807 | 0.00 | 0.42 | 20 | 9 | 242 | nitrogen compound metabolic process |
| GO:0044262 | 0.00 | 0.36 | 15 | 6 | 187 | cellular carbohydrate metabolic process |
| GO:0006839 | 0.00 | 0.00 | 5 | 0 | 63 | mitochondrial transport |
| GO:0048522 | 0.00 | 0.20 | 9 | 2 | 110 | positive regulation of cellular process |
| GO:0051234 | 0.00 | 0.69 | 79 | 59 | 961 | establishment of localization |
| GO:0042221 | 0.00 | 0.52 | 29 | 16 | 347 | response to chemical stimulus |
| GO:0009893 | 0.01 | 0.22 | 9 | 2 | 104 | positive regulation of metabolic process |
| GO:0006066 | 0.01 | 0.35 | 13 | 5 | 160 | alcohol metabolic process |
| GO:0008652 | 0.01 | 0.22 | 8 | 2 | 103 | amino acid biosynthetic process |
| GO:0030001 | 0.01 | 0.00 | 5 | 0 | 57 | metal ion transport |
| GO:0008610 | 0.01 | 0.28 | 10 | 3 | 121 | lipid biosynthetic process |
| GO:0016044 | 0.01 | 0.39 | 14 | 6 | 173 | membrane organization and biogenesis |
| GO:0006643 | 0.01 | 0.28 | 10 | 3 | 119 | membrane lipid metabolic process |
| GO:0045935 | 0.01 | 0.23 | 8 | 2 | 99 | positive regulation of nucleobase, nucleoside, nucleotide and nucleic acid metabolic process |


Krogan2004: Viable Baits Gene to GO MF Conditional test for under-representation

| GOMFID | Pvalue | OddsRatio | ExpCount | Count | Size | Term |
| GO:0005215 | 0.00 | 0.08 | 11 | 1 | 408 | transporter activity |
| GO:0003735 | 0.00 | 0.00 | 6 | 0 | 216 | structural constituent of ribosome |
| GO:0016491 | 0.01 | 0.13 | 7 | 1 | 264 | oxidoreductase activity |
| GO:0005515 | 0.01 | 0.32 | 12 | 4 | 443 | protein binding |


Krogan2004: Viable Prey Gene to GO MF Conditional test for under-representation

| GOMFID | Pvalue | OddsRatio | ExpCount | Count | Size | Term |
| GO:0005215 | 0.00 | 0.24 | 34 | 9 | 408 | transporter activity |
| GO:0016491 | 0.00 | 0.38 | 22 | 9 | 264 | oxidoreductase activity |
| GO:0016758 | 0.00 | 0.00 | 6 | 0 | 76 | transferase activity, transferring hexosyl groups |
| GO:0003700 | 0.01 | 0.00 | 5 | 0 | 60 | transcription factor activity |
| GO:0008324 | 0.01 | 0.27 | 10 | 3 | 125 | cation transporter activity |
| GO:0051082 | 0.01 | 0.00 | 5 | 0 | 58 | unfolded protein binding |
| GO:0016563 | 0.01 | 0.00 | 4 | 0 | 54 | transcriptional activator activity |
