# Supplementary material for: Coverage and error models of protein-protein interaction data by directed graph analysis
Source: Genome Biol. 2007 Sep 10;8(9):R186. doi: 10.1186/gb-2007-8-9-r186 (PMC2375024; doi:10.1186/gb-2007-8-9-r186)
Supplement: Additional data file 2 — Presented is the Bioconductor package ppiStats (version 1.3.5 of 22 June 2007) in 'source' format. ppiStats contains the novel methods developed in this paper. [file gb-2007-8-9-r186-S2.gz › ppiStats/inst/Scripts/Krogan2006.html]

Krogan2006: Viable Baits Gene to GO CC Conditional test for over-representation

| GOCCID | Pvalue | OddsRatio | ExpCount | Count | Size | Term |
| GO:0005622 | 0.00 | 8.47 | 1774 | 2141 | 4563 | intracellular |
| GO:0005634 | 0.00 | 4.02 | 705 | 1118 | 1814 | nucleus |
| GO:0005623 | 0.00 | 8.36 | 1926 | 2185 | 4954 | cell |
| GO:0043233 | 0.00 | 3.14 | 286 | 465 | 736 | organelle lumen |
| GO:0043227 | 0.00 | 1.88 | 1330 | 1537 | 3423 | membrane-bound organelle |
| GO:0043234 | 0.00 | 1.96 | 478 | 638 | 1519 | protein complex |
| GO:0005730 | 0.00 | 3.39 | 88 | 152 | 226 | nucleolus |
| GO:0044446 | 0.00 | 1.61 | 793 | 945 | 2078 | intracellular organelle part |
| GO:0044451 | 0.00 | 3.90 | 57 | 103 | 307 | nucleoplasm part |
| GO:0043232 | 0.00 | 1.69 | 254 | 328 | 931 | intracellular non-membrane-bound organelle |
| GO:0005681 | 0.00 | 4.35 | 26 | 49 | 78 | spliceosome |
| GO:0000794 | 0.00 | 3.46 | 30 | 52 | 76 | condensed nuclear chromosome |
| GO:0000779 | 0.00 | 4.53 | 19 | 37 | 50 | condensed chromosome, pericentric region |
| GO:0030863 | 0.00 | 4.21 | 20 | 37 | 51 | cortical cytoskeleton |
| GO:0005737 | 0.00 | 1.29 | 1301 | 1385 | 3346 | cytoplasm |
| GO:0000776 | 0.00 | 3.78 | 21 | 38 | 54 | kinetochore |
| GO:0005732 | 0.00 | 3.45 | 22 | 39 | 57 | small nucleolar ribonucleoprotein complex |
| GO:0015629 | 0.00 | 2.69 | 27 | 44 | 77 | actin cytoskeleton |
| GO:0005938 | 0.00 | 2.11 | 39 | 57 | 100 | cell cortex |
| GO:0016585 | 0.00 | 4.48 | 9 | 17 | 78 | chromatin remodeling complex |
| GO:0000228 | 0.00 | 4.43 | 7 | 14 | 183 | nuclear chromosome |
| GO:0016591 | 0.00 | 3.95 | 8 | 15 | 72 | DNA-directed RNA polymerase II, holoenzyme |
| GO:0005935 | 0.00 | 1.71 | 44 | 58 | 112 | bud neck |
| GO:0043229 | 0.01 | 1.17 | 609 | 652 | 3755 | intracellular organelle |
| GO:0044454 | 0.01 | 2.33 | 16 | 25 | 153 | nuclear chromosome part |
| GO:0005829 | 0.01 | 1.33 | 128 | 150 | 330 | cytosol |
| GO:0005654 | 0.01 | 3.42 | 7 | 13 | 326 | nucleoplasm |


Krogan2006: Viable Prey Gene to GO CC Conditional test for over-representation

| GOCCID | Pvalue | OddsRatio | ExpCount | Count | Size | Term |
| GO:0005623 | 0.00 | 7.39 | 4291 | 4528 | 4954 | cell |
| GO:0043227 | 0.00 | 2.56 | 2965 | 3120 | 3423 | membrane-bound organelle |
| GO:0005737 | 0.00 | 2.34 | 2898 | 3039 | 3346 | cytoplasm |
| GO:0031974 | 0.00 | 4.22 | 637 | 707 | 736 | membrane-enclosed lumen |
| GO:0044422 | 0.00 | 2.15 | 1800 | 1906 | 2078 | organelle part |
| GO:0031981 | 0.00 | 16.08 | 258 | 295 | 524 | nuclear lumen |
| GO:0043229 | 0.00 | 1.85 | 2446 | 2547 | 3755 | intracellular organelle |
| GO:0043234 | 0.00 | 2.17 | 1022 | 1092 | 1519 | protein complex |
| GO:0005634 | 0.00 | 2.39 | 765 | 824 | 1814 | nucleus |
| GO:0005730 | 0.00 | 7.10 | 196 | 221 | 226 | nucleolus |
| GO:0005667 | 0.00 | 6.69 | 113 | 127 | 130 | transcription factor complex |
| GO:0016585 | 0.00 | Inf | 68 | 78 | 78 | chromatin remodeling complex |
| GO:0005694 | 0.00 | 3.07 | 193 | 212 | 223 | chromosome |
| GO:0016591 | 0.00 | Inf | 62 | 72 | 72 | DNA-directed RNA polymerase II, holoenzyme |
| GO:0005759 | 0.00 | 3.52 | 141 | 156 | 163 | mitochondrial matrix |
| GO:0005840 | 0.00 | 2.10 | 294 | 315 | 339 | ribosome |
| GO:0005829 | 0.00 | 2.03 | 286 | 306 | 330 | cytosol |
| GO:0044454 | 0.00 | 3.29 | 133 | 146 | 153 | nuclear chromosome part |
| GO:0005732 | 0.00 | Inf | 49 | 57 | 57 | small nucleolar ribonucleoprotein complex |
| GO:0044448 | 0.00 | 6.82 | 77 | 87 | 89 | cell cortex part |
| GO:0043232 | 0.00 | 4.32 | 99 | 110 | 931 | intracellular non-membrane-bound organelle |
| GO:0044430 | 0.00 | 4.00 | 92 | 102 | 190 | cytoskeletal part |
| GO:0044451 | 0.00 | 9.85 | 55 | 63 | 307 | nucleoplasm part |
| GO:0044428 | 0.00 | 2.20 | 181 | 195 | 931 | nuclear part |
| GO:0005935 | 0.00 | 3.36 | 97 | 107 | 112 | bud neck |
| GO:0005681 | 0.00 | 3.91 | 68 | 75 | 78 | spliceosome |
| GO:0044424 | 0.00 | Inf | 32 | 37 | 4527 | intracellular part |
| GO:0015629 | 0.01 | 3.85 | 67 | 74 | 77 | actin cytoskeleton |
| GO:0005643 | 0.01 | 7.64 | 43 | 49 | 50 | nuclear pore |
| GO:0005934 | 0.01 | 7.64 | 43 | 49 | 50 | bud tip |
| GO:0005819 | 0.01 | 3.12 | 73 | 80 | 84 | spindle |


Krogan2006: Viable Baits Gene to GO BP Conditional test for over-representation

| GOBPID | Pvalue | OddsRatio | ExpCount | Count | Size | Term |
| GO:0044238 | 0.00 | 2.38 | 921 | 1210 | 2763 | primary metabolic process |
| GO:0032774 | 0.00 | 3.38 | 185 | 314 | 476 | RNA biosynthetic process |
| GO:0006350 | 0.00 | 3.14 | 201 | 332 | 517 | transcription |
| GO:0065007 | 0.00 | 2.33 | 304 | 445 | 783 | biological regulation |
| GO:0044237 | 0.00 | 1.82 | 875 | 1073 | 2988 | cellular metabolic process |
| GO:0006259 | 0.00 | 5.64 | 73 | 146 | 503 | DNA metabolic process |
| GO:0006996 | 0.00 | 2.08 | 370 | 512 | 1272 | organelle organization and biogenesis |
| GO:0006338 | 0.00 | 7.83 | 49 | 105 | 149 | chromatin remodeling |
| GO:0006325 | 0.00 | 6.96 | 49 | 102 | 238 | establishment and/or maintenance of chromatin architecture |
| GO:0031497 | 0.00 | 8.22 | 38 | 81 | 97 | chromatin assembly |
| GO:0016481 | 0.00 | 4.82 | 57 | 109 | 146 | negative regulation of transcription |
| GO:0031324 | 0.00 | 3.86 | 70 | 127 | 181 | negative regulation of cellular metabolic process |
| GO:0016569 | 0.00 | 9.30 | 31 | 69 | 81 | covalent chromatin modification |
| GO:0016070 | 0.00 | 2.77 | 115 | 185 | 918 | RNA metabolic process |
| GO:0040029 | 0.00 | 7.54 | 33 | 70 | 94 | regulation of gene expression, epigenetic |
| GO:0019219 | 0.00 | 2.39 | 141 | 214 | 396 | regulation of nucleobase, nucleoside, nucleotide and nucleic acid metabolic process |
| GO:0006355 | 0.00 | 2.49 | 124 | 191 | 327 | regulation of transcription, DNA-dependent |
| GO:0000398 | 0.00 | 4.77 | 37 | 71 | 95 | nuclear mRNA splicing, via spliceosome |
| GO:0000375 | 0.00 | 4.12 | 40 | 74 | 103 | RNA splicing, via transesterification reactions |
| GO:0044267 | 0.00 | 1.58 | 432 | 531 | 1143 | cellular protein metabolic process |
| GO:0006730 | 0.00 | 7.64 | 20 | 43 | 52 | one-carbon compound metabolic process |
| GO:0006348 | 0.00 | 7.46 | 20 | 42 | 51 | chromatin silencing at telomere |
| GO:0065004 | 0.00 | 4.56 | 28 | 54 | 73 | protein-DNA complex assembly |
| GO:0000278 | 0.00 | 2.69 | 59 | 95 | 244 | mitotic cell cycle |
| GO:0000723 | 0.00 | 2.09 | 105 | 151 | 269 | telomere maintenance |
| GO:0009056 | 0.00 | 1.81 | 157 | 212 | 404 | catabolic process |
| GO:0042254 | 0.00 | 2.98 | 45 | 76 | 321 | ribosome biogenesis and assembly |
| GO:0006365 | 0.00 | 3.83 | 30 | 55 | 78 | 35S primary transcript processing |
| GO:0006519 | 0.00 | 2.22 | 77 | 115 | 199 | amino acid and derivative metabolic process |
| GO:0044271 | 0.00 | 2.85 | 43 | 71 | 111 | nitrogen compound biosynthetic process |
| GO:0006366 | 0.00 | 2.05 | 89 | 128 | 308 | transcription from RNA polymerase II promoter |
| GO:0009308 | 0.00 | 1.99 | 86 | 122 | 221 | amine metabolic process |
| GO:0043285 | 0.00 | 2.84 | 38 | 62 | 268 | biopolymer catabolic process |
| GO:0006399 | 0.00 | 2.61 | 44 | 70 | 113 | tRNA metabolic process |
| GO:0007059 | 0.00 | 3.99 | 22 | 40 | 115 | chromosome segregation |
| GO:0006402 | 0.00 | 4.21 | 20 | 37 | 60 | mRNA catabolic process |
| GO:0050794 | 0.00 | 2.05 | 72 | 103 | 678 | regulation of cellular process |
| GO:0006950 | 0.00 | 1.88 | 92 | 128 | 464 | response to stress |
| GO:0019752 | 0.00 | 1.74 | 119 | 159 | 307 | carboxylic acid metabolic process |
| GO:0006281 | 0.00 | 2.25 | 53 | 80 | 181 | DNA repair |
| GO:0022402 | 0.00 | 3.13 | 28 | 47 | 399 | cell cycle process |
| GO:0044265 | 0.00 | 2.95 | 29 | 48 | 284 | cellular macromolecule catabolic process |
| GO:0043543 | 0.00 | 3.45 | 22 | 39 | 57 | protein amino acid acylation |
| GO:0009889 | 0.00 | 3.04 | 25 | 42 | 64 | regulation of biosynthetic process |
| GO:0043412 | 0.00 | 1.50 | 187 | 231 | 569 | biopolymer modification |
| GO:0007001 | 0.00 | 1.67 | 100 | 131 | 551 | chromosome organization and biogenesis (sensu Eukaryota) |
| GO:0006342 | 0.00 | 20.57 | 5 | 13 | 89 | chromatin silencing |
| GO:0000279 | 0.00 | 1.65 | 97 | 126 | 249 | M phase |
| GO:0006260 | 0.00 | 6.73 | 8 | 17 | 112 | DNA replication |
| GO:0008652 | 0.00 | 2.57 | 26 | 42 | 103 | amino acid biosynthetic process |
| GO:0030029 | 0.00 | 2.06 | 43 | 62 | 110 | actin filament-based process |
| GO:0009451 | 0.00 | 2.54 | 25 | 40 | 65 | RNA modification |
| GO:0005975 | 0.00 | 1.68 | 79 | 104 | 203 | carbohydrate metabolic process |
| GO:0000074 | 0.00 | 2.03 | 42 | 61 | 162 | regulation of progression through cell cycle |
| GO:0006333 | 0.00 | 10.28 | 6 | 13 | 112 | chromatin assembly or disassembly |
| GO:0009653 | 0.00 | 1.57 | 96 | 122 | 247 | anatomical structure morphogenesis |
| GO:0051329 | 0.00 | 2.07 | 36 | 52 | 92 | interphase of mitotic cell cycle |
| GO:0009628 | 0.00 | 1.97 | 41 | 58 | 105 | response to abiotic stimulus |
| GO:0044275 | 0.00 | 2.53 | 22 | 35 | 57 | cellular carbohydrate catabolic process |
| GO:0007067 | 0.00 | 1.90 | 45 | 63 | 125 | mitosis |
| GO:0022618 | 0.00 | 3.96 | 11 | 20 | 130 | protein-RNA complex assembly |
| GO:0006468 | 0.00 | 2.02 | 36 | 52 | 93 | protein amino acid phosphorylation |
| GO:0006006 | 0.00 | 3.17 | 14 | 24 | 62 | glucose metabolic process |
| GO:0040007 | 0.00 | 1.77 | 53 | 72 | 137 | growth |
| GO:0031323 | 0.00 | 2.71 | 18 | 29 | 459 | regulation of cellular metabolic process |
| GO:0043632 | 0.00 | 1.70 | 59 | 79 | 153 | modification-dependent macromolecule catabolic process |
| GO:0007015 | 0.00 | 2.29 | 24 | 36 | 61 | actin filament organization |
| GO:0051168 | 0.00 | 1.92 | 38 | 53 | 97 | nuclear export |
| GO:0019954 | 0.00 | 2.04 | 31 | 45 | 80 | asexual reproduction |
| GO:0006974 | 0.00 | 2.61 | 17 | 28 | 226 | response to DNA damage stimulus |
| GO:0042255 | 0.00 | 2.20 | 24 | 36 | 62 | ribosome assembly |
| GO:0007163 | 0.00 | 1.77 | 44 | 60 | 114 | establishment and/or maintenance of cell polarity |
| GO:0008152 | 0.00 | 3.02 | 12 | 21 | 3064 | metabolic process |
| GO:0006897 | 0.00 | 1.93 | 32 | 45 | 82 | endocytosis |
| GO:0030163 | 0.00 | 1.58 | 66 | 85 | 171 | protein catabolic process |
| GO:0006261 | 0.00 | 2.14 | 24 | 35 | 91 | DNA-dependent DNA replication |
| GO:0030468 | 0.00 | 1.79 | 40 | 54 | 102 | establishment of cell polarity (sensu Fungi) |
| GO:0006403 | 0.00 | 1.87 | 34 | 47 | 87 | RNA localization |
| GO:0043283 | 0.00 | 2.21 | 21 | 32 | 1800 | biopolymer metabolic process |
| GO:0000075 | 0.00 | 2.23 | 21 | 31 | 53 | cell cycle checkpoint |
| GO:0006913 | 0.00 | 3.36 | 10 | 17 | 122 | nucleocytoplasmic transport |
| GO:0051603 | 0.00 | 1.59 | 58 | 74 | 148 | proteolysis involved in cellular protein catabolic process |
| GO:0006310 | 0.00 | 1.71 | 43 | 57 | 110 | DNA recombination |
| GO:0006511 | 0.00 | 1.59 | 57 | 73 | 146 | ubiquitin-dependent protein catabolic process |
| GO:0007017 | 0.00 | 1.76 | 38 | 52 | 99 | microtubule-based process |
| GO:0006796 | 0.00 | 1.49 | 77 | 96 | 199 | phosphate metabolic process |
| GO:0008361 | 0.00 | 1.62 | 50 | 65 | 129 | regulation of cell size |
| GO:0006725 | 0.00 | 2.22 | 19 | 28 | 56 | aromatic compound metabolic process |
| GO:0000819 | 0.01 | 2.01 | 23 | 33 | 59 | sister chromatid segregation |
| GO:0000122 | 0.01 | 2.20 | 17 | 25 | 51 | negative regulation of transcription from RNA polymerase II promoter |
| GO:0006364 | 0.01 | 1.95 | 23 | 32 | 166 | rRNA processing |
| GO:0022403 | 0.01 | 1.96 | 22 | 31 | 328 | cell cycle phase |
| GO:0048523 | 0.01 | 2.32 | 14 | 22 | 218 | negative regulation of cellular process |
| GO:0006397 | 0.01 | 2.59 | 11 | 18 | 139 | mRNA processing |
| GO:0019222 | 0.01 | 2.59 | 11 | 18 | 488 | regulation of metabolic process |


Krogan2006: Viable Prey Gene to GO BP Conditional test for over-representation

| GOBPID | Pvalue | OddsRatio | ExpCount | Count | Size | Term |
| GO:0065007 | 0.00 | 2.80 | 678 | 738 | 783 | biological regulation |
| GO:0044238 | 0.00 | 2.06 | 1053 | 1121 | 2763 | primary metabolic process |
| GO:0050794 | 0.00 | 2.61 | 587 | 637 | 678 | regulation of cellular process |
| GO:0007001 | 0.00 | 2.88 | 477 | 521 | 551 | chromosome organization and biogenesis (sensu Eukaryota) |
| GO:0006350 | 0.00 | 3.00 | 448 | 490 | 517 | transcription |
| GO:0006996 | 0.00 | 2.11 | 791 | 845 | 1272 | organelle organization and biogenesis |
| GO:0006396 | 0.00 | 3.91 | 303 | 336 | 350 | RNA processing |
| GO:0032774 | 0.00 | 2.97 | 412 | 451 | 476 | RNA biosynthetic process |
| GO:0006950 | 0.00 | 3.40 | 340 | 375 | 464 | response to stress |
| GO:0006323 | 0.00 | 6.22 | 206 | 232 | 238 | DNA packaging |
| GO:0009719 | 0.00 | 6.14 | 204 | 229 | 235 | response to endogenous stimulus |
| GO:0065003 | 0.00 | 4.17 | 255 | 284 | 295 | macromolecule complex assembly |
| GO:0016072 | 0.00 | 9.21 | 152 | 173 | 176 | rRNA metabolic process |
| GO:0042254 | 0.00 | 4.47 | 224 | 250 | 321 | ribosome biogenesis and assembly |
| GO:0044237 | 0.00 | 1.96 | 736 | 783 | 2988 | cellular metabolic process |
| GO:0019222 | 0.00 | 2.50 | 423 | 458 | 488 | regulation of metabolic process |
| GO:0006338 | 0.00 | 11.69 | 129 | 147 | 149 | chromatin remodeling |
| GO:0048519 | 0.00 | 3.93 | 199 | 221 | 230 | negative regulation of biological process |
| GO:0006139 | 0.00 | 2.44 | 361 | 391 | 1402 | nucleobase, nucleoside, nucleotide and nucleic acid metabolic process |
| GO:0019219 | 0.00 | 2.51 | 343 | 372 | 396 | regulation of nucleobase, nucleoside, nucleotide and nucleic acid metabolic process |
| GO:0009056 | 0.00 | 2.35 | 350 | 378 | 404 | catabolic process |
| GO:0044249 | 0.00 | 1.77 | 632 | 668 | 841 | cellular biosynthetic process |
| GO:0006365 | 0.00 | Inf | 68 | 78 | 78 | 35S primary transcript processing |
| GO:0031324 | 0.00 | 3.95 | 157 | 174 | 181 | negative regulation of cellular metabolic process |
| GO:0019752 | 0.00 | 2.43 | 266 | 288 | 307 | carboxylic acid metabolic process |
| GO:0006355 | 0.00 | 2.34 | 283 | 306 | 327 | regulation of transcription, DNA-dependent |
| GO:0006261 | 0.00 | 14.16 | 79 | 90 | 91 | DNA-dependent DNA replication |
| GO:0016481 | 0.00 | 4.46 | 126 | 141 | 146 | negative regulation of transcription |
| GO:0043283 | 0.00 | 2.59 | 224 | 244 | 1800 | biopolymer metabolic process |
| GO:0016071 | 0.00 | 5.01 | 113 | 127 | 191 | mRNA metabolic process |
| GO:0006403 | 0.00 | 13.52 | 75 | 86 | 87 | RNA localization |
| GO:0009653 | 0.00 | 2.65 | 214 | 233 | 247 | anatomical structure morphogenesis |
| GO:0006366 | 0.00 | 2.30 | 267 | 288 | 308 | transcription from RNA polymerase II promoter |
| GO:0043632 | 0.00 | 3.88 | 132 | 147 | 153 | modification-dependent macromolecule catabolic process |
| GO:0016070 | 0.00 | 12.72 | 71 | 81 | 918 | RNA metabolic process |
| GO:0031497 | 0.00 | 7.47 | 84 | 95 | 97 | chromatin assembly |
| GO:0040007 | 0.00 | 4.17 | 119 | 132 | 137 | growth |
| GO:0022613 | 0.00 | Inf | 55 | 63 | 384 | ribonucleoprotein complex biogenesis and assembly |
| GO:0051603 | 0.00 | 3.74 | 128 | 142 | 148 | proteolysis involved in cellular protein catabolic process |
| GO:0030163 | 0.00 | 3.23 | 148 | 163 | 171 | protein catabolic process |
| GO:0042255 | 0.00 | Inf | 54 | 62 | 62 | ribosome assembly |
| GO:0006511 | 0.00 | 3.69 | 126 | 140 | 146 | ubiquitin-dependent protein catabolic process |
| GO:0040029 | 0.00 | 7.23 | 81 | 92 | 94 | regulation of gene expression, epigenetic |
| GO:0006468 | 0.00 | 7.15 | 81 | 91 | 93 | protein amino acid phosphorylation |
| GO:0050658 | 0.00 | 11.93 | 67 | 76 | 77 | RNA transport |
| GO:0051329 | 0.00 | 7.07 | 80 | 90 | 92 | interphase of mitotic cell cycle |
| GO:0009308 | 0.00 | 2.54 | 191 | 208 | 221 | amine metabolic process |
| GO:0006519 | 0.00 | 2.71 | 172 | 188 | 199 | amino acid and derivative metabolic process |
| GO:0008361 | 0.00 | 3.91 | 112 | 124 | 129 | regulation of cell size |
| GO:0000279 | 0.00 | 2.31 | 216 | 233 | 249 | M phase |
| GO:0008652 | 0.00 | 5.24 | 89 | 100 | 103 | amino acid biosynthetic process |
| GO:0006342 | 0.00 | 6.83 | 77 | 87 | 89 | chromatin silencing |
| GO:0007059 | 0.00 | 4.37 | 100 | 111 | 115 | chromosome segregation |
| GO:0000074 | 0.00 | 3.04 | 140 | 154 | 162 | regulation of progression through cell cycle |
| GO:0006970 | 0.00 | 10.97 | 61 | 70 | 71 | response to osmotic stress |
| GO:0044271 | 0.00 | 4.21 | 96 | 107 | 111 | nitrogen compound biosynthetic process |
| GO:0051168 | 0.00 | 4.92 | 84 | 94 | 97 | nuclear export |
| GO:0044262 | 0.00 | 2.53 | 162 | 176 | 187 | cellular carbohydrate metabolic process |
| GO:0016570 | 0.00 | 6.19 | 70 | 79 | 81 | histone modification |
| GO:0022403 | 0.00 | 6.03 | 68 | 77 | 328 | cell cycle phase |
| GO:0006406 | 0.00 | 9.86 | 55 | 63 | 64 | mRNA export from nucleus |
| GO:0000375 | 0.00 | 3.89 | 89 | 99 | 103 | RNA splicing, via transesterification reactions |
| GO:0051641 | 0.00 | 1.57 | 484 | 507 | 559 | cellular localization |
| GO:0006464 | 0.00 | 1.81 | 279 | 296 | 496 | protein modification |
| GO:0006281 | 0.00 | 3.73 | 86 | 95 | 181 | DNA repair |
| GO:0030029 | 0.00 | 3.30 | 95 | 105 | 110 | actin filament-based process |
| GO:0015931 | 0.00 | 4.34 | 74 | 83 | 86 | nucleobase, nucleoside, nucleotide and nucleic acid transport |
| GO:0007165 | 0.00 | 2.19 | 167 | 180 | 193 | signal transduction |
| GO:0000278 | 0.00 | 2.50 | 132 | 143 | 244 | mitotic cell cycle |
| GO:0016043 | 0.00 | 1.74 | 290 | 307 | 2008 | cell organization and biogenesis |
| GO:0051321 | 0.00 | 2.62 | 122 | 133 | 141 | meiotic cell cycle |
| GO:0007126 | 0.00 | 2.62 | 122 | 133 | 141 | meiosis |
| GO:0000398 | 0.00 | 3.57 | 82 | 91 | 95 | nuclear mRNA splicing, via spliceosome |
| GO:0006457 | 0.00 | 4.18 | 72 | 80 | 83 | protein folding |
| GO:0006412 | 0.00 | 1.72 | 287 | 303 | 372 | translation |
| GO:0045045 | 0.00 | 1.93 | 206 | 220 | 238 | secretory pathway |
| GO:0006352 | 0.00 | 8.59 | 48 | 55 | 56 | transcription initiation |
| GO:0006897 | 0.00 | 4.12 | 71 | 79 | 82 | endocytosis |
| GO:0043412 | 0.00 | 8.44 | 48 | 54 | 569 | biopolymer modification |
| GO:0007114 | 0.00 | 4.02 | 69 | 77 | 80 | cell budding |
| GO:0043170 | 0.00 | 1.56 | 373 | 391 | 2355 | macromolecule metabolic process |
| GO:0000723 | 0.00 | 1.78 | 233 | 247 | 269 | telomere maintenance |
| GO:0000910 | 0.00 | 3.01 | 87 | 96 | 101 | cytokinesis |
| GO:0043284 | 0.00 | 7.96 | 45 | 51 | 52 | biopolymer biosynthetic process |
| GO:0044267 | 0.01 | 1.92 | 183 | 195 | 1143 | cellular protein metabolic process |
| GO:0051234 | 0.01 | 1.33 | 832 | 857 | 961 | establishment of localization |
| GO:0044265 | 0.01 | 2.41 | 113 | 123 | 284 | cellular macromolecule catabolic process |
| GO:0006302 | 0.01 | 7.80 | 44 | 50 | 51 | double-strand break repair |
| GO:0046907 | 0.01 | 1.47 | 450 | 469 | 520 | intracellular transport |
| GO:0007124 | 0.01 | 4.77 | 55 | 61 | 63 | pseudohyphal growth |
| GO:0043285 | 0.01 | 2.88 | 84 | 92 | 268 | biopolymer catabolic process |
| GO:0007015 | 0.01 | 4.61 | 53 | 59 | 61 | actin filament organization |
| GO:0006402 | 0.01 | 4.53 | 52 | 58 | 60 | mRNA catabolic process |
| GO:0016568 | 0.01 | 4.53 | 52 | 58 | 209 | chromatin modification |
| GO:0007067 | 0.01 | 2.29 | 108 | 117 | 125 | mitosis |


Krogan2006: Viable Baits Gene to GO MF Conditional test for over-representation

| GOMFID | Pvalue | OddsRatio | ExpCount | Count | Size | Term |
| GO:0030528 | 0.00 | 2.20 | 124 | 183 | 320 | transcription regulator activity |
| GO:0003676 | 0.00 | 7.29 | 19 | 41 | 505 | nucleic acid binding |
| GO:0045182 | 0.00 | 4.37 | 22 | 41 | 56 | translation regulator activity |
| GO:0008092 | 0.00 | 4.33 | 20 | 38 | 52 | cytoskeletal protein binding |
| GO:0005488 | 0.00 | 1.58 | 194 | 246 | 1056 | binding |
| GO:0016251 | 0.00 | 5.10 | 16 | 32 | 62 | general RNA polymerase II transcription factor activity |
| GO:0031202 | 0.00 | 4.22 | 20 | 37 | 51 | RNA splicing factor activity, transesterification mechanism |
| GO:0003723 | 0.00 | 2.27 | 50 | 75 | 236 | RNA binding |
| GO:0016787 | 0.00 | 1.55 | 178 | 223 | 734 | hydrolase activity |
| GO:0003677 | 0.00 | 1.77 | 84 | 113 | 226 | DNA binding |
| GO:0003824 | 0.00 | 1.33 | 388 | 445 | 1907 | catalytic activity |
| GO:0016772 | 0.00 | 2.30 | 37 | 56 | 295 | transferase activity, transferring phosphorus-containing groups |
| GO:0016741 | 0.00 | 2.40 | 33 | 51 | 85 | transferase activity, transferring one-carbon groups |
| GO:0004674 | 0.00 | 2.39 | 27 | 42 | 70 | protein serine/threonine kinase activity |
| GO:0016791 | 0.00 | 2.10 | 34 | 50 | 88 | phosphoric monoester hydrolase activity |
| GO:0016773 | 0.00 | 1.68 | 67 | 88 | 172 | phosphotransferase activity, alcohol group as acceptor |
| GO:0016853 | 0.00 | 2.46 | 22 | 34 | 56 | isomerase activity |
| GO:0016817 | 0.00 | 1.49 | 107 | 133 | 276 | hydrolase activity, acting on acid anhydrides |
| GO:0016462 | 0.00 | 1.49 | 107 | 133 | 276 | pyrophosphatase activity |
| GO:0016301 | 0.00 | 1.57 | 77 | 98 | 198 | kinase activity |
| GO:0004518 | 0.00 | 1.87 | 39 | 54 | 100 | nuclease activity |
| GO:0016887 | 0.00 | 1.52 | 76 | 96 | 197 | ATPase activity |
| GO:0008094 | 0.00 | 2.27 | 20 | 30 | 51 | DNA-dependent ATPase activity |
| GO:0016407 | 0.00 | 2.19 | 19 | 29 | 50 | acetyltransferase activity |
| GO:0008757 | 0.01 | 2.05 | 21 | 31 | 62 | S-adenosylmethionine-dependent methyltransferase activity |
| GO:0030234 | 0.01 | 1.47 | 73 | 90 | 188 | enzyme regulator activity |
| GO:0016874 | 0.01 | 1.54 | 50 | 63 | 128 | ligase activity |


Krogan2006: Viable Prey Gene to GO MF Conditional test for over-representation

| GOMFID | Pvalue | OddsRatio | ExpCount | Count | Size | Term |
| GO:0016772 | 0.00 | 5.82 | 255 | 287 | 295 | transferase activity, transferring phosphorus-containing groups |
| GO:0003723 | 0.00 | 10.70 | 176 | 200 | 236 | RNA binding |
| GO:0016817 | 0.00 | 5.42 | 239 | 268 | 276 | hydrolase activity, acting on acid anhydrides |
| GO:0016462 | 0.00 | 5.42 | 239 | 268 | 276 | pyrophosphatase activity |
| GO:0016887 | 0.00 | 10.36 | 171 | 194 | 197 | ATPase activity |
| GO:0030528 | 0.00 | 2.40 | 277 | 300 | 320 | transcription regulator activity |
| GO:0004674 | 0.00 | Inf | 61 | 70 | 70 | protein serine/threonine kinase activity |
| GO:0016787 | 0.00 | 1.95 | 397 | 423 | 734 | hydrolase activity |
| GO:0005198 | 0.00 | 2.09 | 293 | 314 | 338 | structural molecule activity |
| GO:0016251 | 0.00 | Inf | 54 | 62 | 62 | general RNA polymerase II transcription factor activity |
| GO:0008094 | 0.00 | Inf | 44 | 51 | 51 | DNA-dependent ATPase activity |
| GO:0016746 | 0.00 | 3.96 | 91 | 101 | 105 | transferase activity, transferring acyl groups |
| GO:0016740 | 0.00 | 2.03 | 216 | 231 | 647 | transferase activity |
| GO:0005488 | 0.00 | 2.71 | 126 | 138 | 1056 | binding |
| GO:0004518 | 0.00 | 3.76 | 87 | 96 | 100 | nuclease activity |
| GO:0003677 | 0.00 | 2.07 | 196 | 210 | 226 | DNA binding |
| GO:0004672 | 0.00 | 8.91 | 50 | 57 | 128 | protein kinase activity |
| GO:0016829 | 0.00 | 4.17 | 72 | 80 | 83 | lyase activity |
| GO:0008415 | 0.00 | 3.37 | 78 | 86 | 90 | acyltransferase activity |
| GO:0003676 | 0.01 | 7.64 | 43 | 49 | 505 | nucleic acid binding |
| GO:0016879 | 0.01 | 3.75 | 65 | 72 | 75 | ligase activity, forming carbon-nitrogen bonds |
| GO:0005386 | 0.01 | 3.70 | 64 | 71 | 74 | carrier activity |
| GO:0005515 | 0.01 | 1.48 | 384 | 400 | 443 | protein binding |
| GO:0003824 | 0.01 | 1.45 | 411 | 428 | 1907 | catalytic activity |


Krogan2006: Viable Baits Gene to GO CC Conditional test for under-representation

| GOCCID | Pvalue | OddsRatio | ExpCount | Count | Size | Term |
| GO:0005783 | 0.00 | 0.28 | 133 | 55 | 343 | endoplasmic reticulum |
| GO:0005886 | 0.00 | 0.19 | 76 | 22 | 255 | plasma membrane |
| GO:0005740 | 0.00 | 0.27 | 100 | 39 | 288 | mitochondrial envelope |
| GO:0031975 | 0.00 | 0.40 | 152 | 83 | 391 | envelope |
| GO:0005743 | 0.00 | 0.18 | 48 | 13 | 161 | mitochondrial inner membrane |
| GO:0005789 | 0.00 | 0.25 | 50 | 18 | 128 | endoplasmic reticulum membrane |
| GO:0005773 | 0.00 | 0.37 | 75 | 38 | 194 | vacuole |
| GO:0016020 | 0.00 | 0.12 | 26 | 5 | 1071 | membrane |
| GO:0031090 | 0.00 | 0.53 | 151 | 100 | 620 | organelle membrane |
| GO:0000324 | 0.00 | 0.29 | 32 | 13 | 152 | vacuole (sensu Fungi) |
| GO:0005777 | 0.00 | 0.19 | 21 | 6 | 54 | peroxisome |
| GO:0030312 | 0.00 | 0.34 | 38 | 18 | 99 | external encapsulating structure |
| GO:0009277 | 0.00 | 0.34 | 38 | 18 | 99 | cell wall (sensu Fungi) |
| GO:0005739 | 0.00 | 0.74 | 402 | 342 | 1035 | mitochondrion |
| GO:0016021 | 0.00 | 0.49 | 69 | 43 | 269 | integral to membrane |
| GO:0044459 | 0.00 | 0.31 | 23 | 10 | 60 | plasma membrane part |
| GO:0012505 | 0.00 | 0.65 | 115 | 88 | 296 | endomembrane system |
| GO:0005624 | 0.00 | 0.43 | 32 | 18 | 83 | membrane fraction |
| GO:0044455 | 0.00 | 0.38 | 24 | 12 | 98 | mitochondrial membrane part |
| GO:0005774 | 0.00 | 0.26 | 14 | 5 | 105 | vacuolar membrane |
| GO:0031301 | 0.00 | 0.28 | 13 | 5 | 64 | integral to organelle membrane |
| GO:0044425 | 0.00 | 0.72 | 127 | 104 | 611 | membrane part |
| GO:0000329 | 0.01 | 0.50 | 27 | 17 | 70 | vacuolar membrane (sensu Fungi) |


Krogan2006: Viable Prey Gene to GO CC Conditional test for under-representation

| GOCCID | Pvalue | OddsRatio | ExpCount | Count | Size | Term |
| GO:0005740 | 0.00 | 0.58 | 249 | 229 | 288 | mitochondrial envelope |
| GO:0044455 | 0.00 | 0.44 | 74 | 64 | 98 | mitochondrial membrane part |


Krogan2006: Viable Baits Gene to GO BP Conditional test for under-representation

| GOBPID | Pvalue | OddsRatio | ExpCount | Count | Size | Term |
| GO:0015849 | 0.00 | 0.03 | 20 | 1 | 51 | organic acid transport |
| GO:0051234 | 0.00 | 0.66 | 373 | 296 | 961 | establishment of localization |
| GO:0006812 | 0.00 | 0.14 | 24 | 5 | 92 | cation transport |
| GO:0009101 | 0.00 | 0.26 | 30 | 11 | 77 | glycoprotein biosynthetic process |
| GO:0043413 | 0.00 | 0.25 | 28 | 10 | 72 | biopolymer glycosylation |
| GO:0006875 | 0.00 | 0.29 | 25 | 10 | 64 | metal ion homeostasis |
| GO:0044255 | 0.00 | 0.54 | 67 | 45 | 219 | cellular lipid metabolic process |
| GO:0007047 | 0.00 | 0.57 | 75 | 52 | 194 | cell wall organization and biogenesis |
| GO:0006626 | 0.00 | 0.30 | 19 | 8 | 50 | protein targeting to mitochondrion |
| GO:0009060 | 0.00 | 0.45 | 31 | 18 | 80 | aerobic respiration |
| GO:0006811 | 0.00 | 0.09 | 7 | 1 | 110 | ion transport |
| GO:0046467 | 0.00 | 0.37 | 20 | 10 | 71 | membrane lipid biosynthetic process |
| GO:0006644 | 0.00 | 0.53 | 35 | 23 | 91 | phospholipid metabolic process |
| GO:0006873 | 0.00 | 0.56 | 41 | 28 | 106 | cell ion homeostasis |
| GO:0048878 | 0.00 | 0.57 | 44 | 30 | 112 | chemical homeostasis |


Krogan2006: Viable Prey Gene to GO BP Conditional test for under-representation

| GOBPID | Pvalue | OddsRatio | ExpCount | Count | Size | Term |
| GO:0006839 | 0.00 | 0.41 | 55 | 46 | 63 | mitochondrial transport |


Krogan2006: Viable Baits Gene to GO MF Conditional test for under-representation

| GOMFID | Pvalue | OddsRatio | ExpCount | Count | Size | Term |
| GO:0005215 | 0.00 | 0.21 | 52 | 16 | 408 | transporter activity |
| GO:0008324 | 0.00 | 0.23 | 33 | 11 | 125 | cation transporter activity |
| GO:0015075 | 0.00 | 0.00 | 8 | 0 | 146 | ion transporter activity |
| GO:0042626 | 0.00 | 0.26 | 22 | 8 | 56 | ATPase activity, coupled to transmembrane movement of substances |
| GO:0016757 | 0.00 | 0.40 | 38 | 20 | 97 | transferase activity, transferring glycosyl groups |
| GO:0005386 | 0.00 | 0.40 | 29 | 15 | 74 | carrier activity |
| GO:0015078 | 0.00 | 0.36 | 21 | 10 | 54 | hydrogen ion transporter activity |
| GO:0003735 | 0.00 | 0.67 | 84 | 65 | 216 | structural constituent of ribosome |


Krogan2006: Viable Prey Gene to GO MF Conditional test for under-representation

| GOMFID | Pvalue | OddsRatio | ExpCount | Count | Size | Term |
| GO:0015077 | 0.00 | 0.38 | 51 | 42 | 59 | monovalent inorganic cation transporter activity |
