# Supplementary material for: Coverage and error models of protein-protein interaction data by directed graph analysis
Source: Genome Biol. 2007 Sep 10;8(9):R186. doi: 10.1186/gb-2007-8-9-r186 (PMC2375024; doi:10.1186/gb-2007-8-9-r186)
Supplement: Additional data file 2 — Presented is the Bioconductor package ppiStats (version 1.3.5 of 22 June 2007) in 'source' format. ppiStats contains the novel methods developed in this paper. [file gb-2007-8-9-r186-S2.gz › ppiStats/inst/Scripts/Tong2002.html]

Tong2002: Viable Baits Gene to GO CC Conditional test for over-representation

| GOCCID | Pvalue | OddsRatio | ExpCount | Count | Size | Term |
| GO:0005938 | 0.00 | 41.30 | 0 | 8 | 100 | cell cortex |
| GO:0015629 | 0.00 | 44.02 | 0 | 7 | 77 | actin cytoskeleton |
| GO:0030863 | 0.00 | 54.73 | 0 | 6 | 51 | cortical cytoskeleton |
| GO:0005935 | 0.00 | 22.99 | 0 | 6 | 112 | bud neck |
| GO:0043232 | 0.01 | 3.52 | 3 | 8 | 931 | intracellular non-membrane-bound organelle |


Tong2002: Viable Prey Gene to GO CC Conditional test for over-representation

| GOCCID | Pvalue | OddsRatio | ExpCount | Count | Size | Term |
| GO:0015629 | 0.00 | 17.68 | 1 | 10 | 77 | actin cytoskeleton |
| GO:0030863 | 0.00 | 21.25 | 1 | 8 | 51 | cortical cytoskeleton |
| GO:0005938 | 0.00 | 13.11 | 1 | 10 | 100 | cell cortex |
| GO:0030427 | 0.00 | 6.37 | 1 | 8 | 149 | site of polarized growth |
| GO:0044431 | 0.00 | 5.11 | 1 | 6 | 133 | Golgi apparatus part |
| GO:0005933 | 0.00 | 4.59 | 1 | 6 | 147 | bud |


Tong2002: Viable Baits Gene to GO BP Conditional test for over-representation

| GOBPID | Pvalue | OddsRatio | ExpCount | Count | Size | Term |
| GO:0009653 | 0.00 | 23.44 | 1 | 10 | 247 | anatomical structure morphogenesis |
| GO:0030468 | 0.00 | 32.30 | 0 | 7 | 102 | establishment of cell polarity (sensu Fungi) |
| GO:0007163 | 0.00 | 28.61 | 0 | 7 | 114 | establishment and/or maintenance of cell polarity |
| GO:0006897 | 0.00 | 24.74 | 0 | 5 | 82 | endocytosis |
| GO:0007010 | 0.00 | 12.11 | 1 | 6 | 220 | cytoskeleton organization and biogenesis |
| GO:0007015 | 0.00 | 25.16 | 0 | 4 | 61 | actin filament organization |
| GO:0006970 | 0.00 | 21.37 | 0 | 4 | 71 | response to osmotic stress |
| GO:0016044 | 0.00 | 11.16 | 1 | 5 | 173 | membrane organization and biogenesis |
| GO:0000910 | 0.00 | 14.68 | 0 | 4 | 101 | cytokinesis |
| GO:0030036 | 0.00 | 24.16 | 0 | 3 | 106 | actin cytoskeleton organization and biogenesis |
| GO:0007242 | 0.00 | 11.92 | 0 | 4 | 123 | intracellular signaling cascade |
| GO:0007154 | 0.00 | 8.57 | 1 | 5 | 222 | cell communication |
| GO:0007105 | 0.00 | 16.31 | 0 | 3 | 65 | cytokinesis, site selection |
| GO:0032505 | 0.00 | 6.74 | 1 | 5 | 278 | reproduction of a single-celled organism |
| GO:0019954 | 0.00 | 13.10 | 0 | 3 | 80 | asexual reproduction |
| GO:0050896 | 0.01 | 3.88 | 2 | 7 | 713 | response to stimulus |
| GO:0016043 | 0.01 | 3.27 | 6 | 12 | 2008 | cell organization and biogenesis |


Tong2002: Viable Prey Gene to GO BP Conditional test for over-representation

| GOBPID | Pvalue | OddsRatio | ExpCount | Count | Size | Term |
| GO:0030036 | 0.00 | 20.65 | 0 | 7 | 106 | actin cytoskeleton organization and biogenesis |
| GO:0007163 | 0.00 | 6.03 | 1 | 6 | 114 | establishment and/or maintenance of cell polarity |
| GO:0016043 | 0.00 | 2.35 | 20 | 32 | 2008 | cell organization and biogenesis |
| GO:0009653 | 0.00 | 3.69 | 2 | 8 | 247 | anatomical structure morphogenesis |
| GO:0007015 | 0.00 | 7.40 | 1 | 4 | 61 | actin filament organization |
| GO:0000910 | 0.00 | 5.56 | 1 | 5 | 101 | cytokinesis |
| GO:0030468 | 0.00 | 5.50 | 1 | 5 | 102 | establishment of cell polarity (sensu Fungi) |
| GO:0007105 | 0.00 | 6.91 | 1 | 4 | 65 | cytokinesis, site selection |
| GO:0007010 | 0.00 | 3.87 | 2 | 7 | 220 | cytoskeleton organization and biogenesis |
| GO:0019954 | 0.01 | 5.54 | 1 | 4 | 80 | asexual reproduction |
| GO:0006512 | 0.01 | 5.46 | 1 | 4 | 81 | ubiquitin cycle |
| GO:0006897 | 0.01 | 5.39 | 1 | 4 | 82 | endocytosis |


Tong2002: Viable Baits Gene to GO MF Conditional test for over-representation

| GOMFID | Pvalue | OddsRatio | ExpCount | Count | Size | Term |
| GO:0008092 | 0.00 | 29.86 | 0 | 4 | 52 | cytoskeletal protein binding |
| GO:0005488 | 0.00 | 4.53 | 4 | 10 | 1056 | binding |


Tong2002: Viable Prey Gene to GO MF Conditional test for over-representation

| GOMFID | Pvalue | OddsRatio | ExpCount | Count | Size | Term |
| GO:0008092 | 0.00 | 11.43 | 1 | 5 | 52 | cytoskeletal protein binding |
| GO:0005515 | 0.00 | 2.93 | 4 | 10 | 443 | protein binding |


Tong2002: Viable Baits Gene to GO CC Conditional test for under-representation

| GOCCID | Pvalue | OddsRatio | ExpCount | Count | Size | Term |
| GO:0043227 | 0.00 | 0.17 | 12 | 4 | 3423 | membrane-bound organelle |
| GO:0043234 | 0.00 | 0.00 | 5 | 0 | 1519 | protein complex |
| GO:0005634 | 0.01 | 0.12 | 6 | 1 | 1814 | nucleus |


Tong2002: Viable Baits Gene to GO BP Conditional test for under-representation

| GOBPID | Pvalue | OddsRatio | ExpCount | Count | Size | Term |
| GO:0044237 | 0.00 | 0.05 | 10 | 1 | 2988 | cellular metabolic process |
| GO:0043283 | 0.01 | 0.12 | 6 | 1 | 1800 | biopolymer metabolic process |
| GO:0019538 | 0.01 | 0.00 | 4 | 0 | 1194 | protein metabolic process |


Tong2002: Viable Prey Gene to GO BP Conditional test for under-representation

| GOBPID | Pvalue | OddsRatio | ExpCount | Count | Size | Term |
| GO:0008152 | 0.01 | 0.51 | 31 | 21 | 3064 | metabolic process |


Tong2002: Viable Baits Gene to GO MF Conditional test for under-representation

| GOMFID | Pvalue | OddsRatio | ExpCount | Count | Size | Term |
| GO:0003824 | 0.00 | 0.00 | 7 | 0 | 1907 | catalytic activity |


Tong2002: Viable Prey Gene to GO MF Conditional test for under-representation

| GOMFID | Pvalue | OddsRatio | ExpCount | Count | Size | Term |
| GO:0016740 | 0.01 | 0.14 | 6 | 1 | 647 | transferase activity |
