# Supplementary material for: Coverage and error models of protein-protein interaction data by directed graph analysis
Source: Genome Biol. 2007 Sep 10;8(9):R186. doi: 10.1186/gb-2007-8-9-r186 (PMC2375024; doi:10.1186/gb-2007-8-9-r186)
Supplement: Additional data file 2 — Presented is the Bioconductor package ppiStats (version 1.3.5 of 22 June 2007) in 'source' format. ppiStats contains the novel methods developed in this paper. [file gb-2007-8-9-r186-S2.gz › ppiStats/inst/Scripts/Uetz2000-1.html]

Uetz2000-1: Viable Baits Gene to GO CC Conditional test for over-representation

| GOCCID | Pvalue | OddsRatio | ExpCount | Count | Size | Term |
| GO:0005623 | 0.00 | 2.07 | 423 | 456 | 4954 | cell |
| GO:0044424 | 0.00 | 1.76 | 385 | 424 | 4527 | intracellular part |
| GO:0000776 | 0.00 | 4.22 | 5 | 15 | 54 | kinetochore |
| GO:0000779 | 0.00 | 4.26 | 4 | 14 | 50 | condensed chromosome, pericentric region |
| GO:0005634 | 0.00 | 1.55 | 75 | 104 | 1814 | nucleus |
| GO:0043234 | 0.00 | 1.41 | 127 | 159 | 1519 | protein complex |
| GO:0000794 | 0.00 | 2.92 | 6 | 16 | 76 | condensed nuclear chromosome |
| GO:0005681 | 0.00 | 4.07 | 3 | 9 | 78 | spliceosome |
| GO:0015630 | 0.00 | 2.32 | 9 | 18 | 103 | microtubule cytoskeleton |
| GO:0044428 | 0.00 | 1.42 | 73 | 95 | 931 | nuclear part |
| GO:0005816 | 0.00 | 2.67 | 5 | 12 | 61 | spindle pole body |


Uetz2000-1: Viable Prey Gene to GO CC Conditional test for over-representation

| GOCCID | Pvalue | OddsRatio | ExpCount | Count | Size | Term |
| GO:0005634 | 0.00 | 1.76 | 189 | 259 | 1814 | nucleus |
| GO:0000776 | 0.00 | 3.70 | 6 | 16 | 54 | kinetochore |
| GO:0005623 | 0.00 | 1.64 | 515 | 544 | 4954 | cell |
| GO:0044424 | 0.00 | 1.47 | 465 | 499 | 4527 | intracellular part |
| GO:0005681 | 0.00 | 2.83 | 8 | 19 | 78 | spliceosome |
| GO:0005816 | 0.00 | 2.61 | 6 | 14 | 61 | spindle pole body |
| GO:0000779 | 0.00 | 2.76 | 5 | 12 | 50 | condensed chromosome, pericentric region |
| GO:0044451 | 0.00 | 1.61 | 30 | 44 | 307 | nucleoplasm part |
| GO:0005819 | 0.01 | 2.22 | 9 | 17 | 84 | spindle |
| GO:0005856 | 0.01 | 1.70 | 21 | 33 | 204 | cytoskeleton |


Uetz2000-1: Viable Baits Gene to GO BP Conditional test for over-representation

| GOBPID | Pvalue | OddsRatio | ExpCount | Count | Size | Term |
| GO:0065007 | 0.00 | 1.84 | 67 | 105 | 783 | biological regulation |
| GO:0022402 | 0.00 | 2.26 | 28 | 55 | 399 | cell cycle process |
| GO:0000278 | 0.00 | 2.42 | 21 | 43 | 244 | mitotic cell cycle |
| GO:0000398 | 0.00 | 3.54 | 8 | 23 | 95 | nuclear mRNA splicing, via spliceosome |
| GO:0043283 | 0.00 | 1.54 | 154 | 198 | 1800 | biopolymer metabolic process |
| GO:0000375 | 0.00 | 3.18 | 9 | 23 | 103 | RNA splicing, via transesterification reactions |
| GO:0016071 | 0.00 | 2.33 | 16 | 33 | 191 | mRNA metabolic process |
| GO:0051327 | 0.00 | 2.50 | 12 | 26 | 141 | M phase of meiotic cell cycle |
| GO:0007165 | 0.00 | 2.21 | 16 | 32 | 193 | signal transduction |
| GO:0006511 | 0.00 | 2.40 | 12 | 26 | 146 | ubiquitin-dependent protein catabolic process |
| GO:0006468 | 0.00 | 2.82 | 8 | 19 | 93 | protein amino acid phosphorylation |
| GO:0051603 | 0.00 | 2.36 | 13 | 26 | 148 | proteolysis involved in cellular protein catabolic process |
| GO:0000074 | 0.00 | 2.36 | 13 | 26 | 162 | regulation of progression through cell cycle |
| GO:0044238 | 0.00 | 1.39 | 236 | 273 | 2763 | primary metabolic process |
| GO:0016043 | 0.00 | 1.40 | 171 | 207 | 2008 | cell organization and biogenesis |
| GO:0043632 | 0.00 | 2.26 | 13 | 26 | 153 | modification-dependent macromolecule catabolic process |
| GO:0007127 | 0.00 | 3.10 | 6 | 15 | 68 | meiosis I |
| GO:0030163 | 0.00 | 2.16 | 15 | 28 | 171 | protein catabolic process |
| GO:0044248 | 0.00 | 1.73 | 33 | 52 | 390 | cellular catabolic process |
| GO:0009057 | 0.00 | 1.77 | 27 | 43 | 314 | macromolecule catabolic process |
| GO:0050794 | 0.00 | 1.62 | 39 | 58 | 678 | regulation of cellular process |
| GO:0051325 | 0.00 | 2.48 | 8 | 17 | 92 | interphase |
| GO:0007049 | 0.00 | 5.91 | 1 | 6 | 417 | cell cycle |
| GO:0050896 | 0.00 | 1.47 | 61 | 82 | 713 | response to stimulus |
| GO:0006913 | 0.00 | 2.15 | 10 | 20 | 122 | nucleocytoplasmic transport |
| GO:0009653 | 0.00 | 1.76 | 21 | 34 | 247 | anatomical structure morphogenesis |
| GO:0048523 | 0.00 | 1.81 | 17 | 29 | 218 | negative regulation of cellular process |
| GO:0006796 | 0.01 | 1.80 | 17 | 28 | 199 | phosphate metabolic process |
| GO:0040007 | 0.01 | 1.98 | 12 | 21 | 137 | growth |
| GO:0008361 | 0.01 | 2.01 | 11 | 20 | 129 | regulation of cell size |
| GO:0000746 | 0.01 | 2.07 | 10 | 18 | 113 | conjugation |
| GO:0019953 | 0.01 | 2.07 | 10 | 18 | 113 | sexual reproduction |
| GO:0045892 | 0.01 | 1.91 | 12 | 21 | 141 | negative regulation of transcription, DNA-dependent |
| GO:0051169 | 0.01 | 2.03 | 10 | 18 | 115 | nuclear transport |
| GO:0007017 | 0.01 | 2.10 | 8 | 16 | 99 | microtubule-based process |
| GO:0007067 | 0.01 | 1.96 | 11 | 19 | 125 | mitosis |


Uetz2000-1: Viable Prey Gene to GO BP Conditional test for over-representation

| GOBPID | Pvalue | OddsRatio | ExpCount | Count | Size | Term |
| GO:0065007 | 0.00 | 1.96 | 81 | 132 | 783 | biological regulation |
| GO:0007049 | 0.00 | 2.61 | 18 | 39 | 417 | cell cycle |
| GO:0006139 | 0.00 | 1.53 | 146 | 191 | 1402 | nucleobase, nucleoside, nucleotide and nucleic acid metabolic process |
| GO:0051726 | 0.00 | 2.37 | 17 | 34 | 162 | regulation of cell cycle |
| GO:0000398 | 0.00 | 2.82 | 10 | 23 | 95 | nuclear mRNA splicing, via spliceosome |
| GO:0006351 | 0.00 | 1.69 | 49 | 74 | 471 | transcription, DNA-dependent |
| GO:0043285 | 0.00 | 1.90 | 28 | 47 | 268 | biopolymer catabolic process |
| GO:0006623 | 0.00 | 3.18 | 7 | 17 | 64 | protein targeting to vacuole |
| GO:0044265 | 0.00 | 1.87 | 30 | 49 | 284 | cellular macromolecule catabolic process |
| GO:0016071 | 0.00 | 2.34 | 13 | 27 | 191 | mRNA metabolic process |
| GO:0000375 | 0.00 | 2.54 | 11 | 23 | 103 | RNA splicing, via transesterification reactions |
| GO:0000278 | 0.00 | 1.91 | 25 | 43 | 244 | mitotic cell cycle |
| GO:0006402 | 0.00 | 3.32 | 5 | 14 | 60 | mRNA catabolic process |
| GO:0051276 | 0.00 | 34.72 | 1 | 4 | 556 | chromosome organization and biogenesis |
| GO:0050794 | 0.00 | 2.59 | 9 | 20 | 678 | regulation of cellular process |
| GO:0043283 | 0.00 | 1.36 | 159 | 193 | 1800 | biopolymer metabolic process |
| GO:0031323 | 0.00 | 1.56 | 48 | 68 | 459 | regulation of cellular metabolic process |
| GO:0019222 | 0.00 | 3.92 | 3 | 9 | 488 | regulation of metabolic process |
| GO:0009056 | 0.00 | 1.56 | 42 | 60 | 404 | catabolic process |
| GO:0000075 | 0.00 | 2.84 | 6 | 13 | 53 | cell cycle checkpoint |
| GO:0000226 | 0.00 | 2.37 | 8 | 17 | 80 | microtubule cytoskeleton organization and biogenesis |
| GO:0019236 | 0.00 | 2.26 | 9 | 18 | 88 | response to pheromone |
| GO:0007010 | 0.00 | 1.73 | 23 | 36 | 220 | cytoskeleton organization and biogenesis |
| GO:0006357 | 0.00 | 1.75 | 21 | 34 | 206 | regulation of transcription from RNA polymerase II promoter |
| GO:0007001 | 0.00 | 1.43 | 57 | 76 | 551 | chromosome organization and biogenesis (sensu Eukaryota) |
| GO:0007088 | 0.01 | 2.69 | 5 | 12 | 51 | regulation of mitosis |
| GO:0000279 | 0.01 | 1.64 | 26 | 39 | 249 | M phase |
| GO:0032446 | 0.01 | 2.26 | 8 | 15 | 79 | protein modification by small protein conjugation |
| GO:0016043 | 0.01 | 1.24 | 209 | 236 | 2008 | cell organization and biogenesis |


Uetz2000-1: Viable Baits Gene to GO MF Conditional test for over-representation

| GOMFID | Pvalue | OddsRatio | ExpCount | Count | Size | Term |
| GO:0016773 | 0.00 | 2.35 | 15 | 30 | 172 | phosphotransferase activity, alcohol group as acceptor |
| GO:0016301 | 0.00 | 2.23 | 17 | 33 | 198 | kinase activity |
| GO:0031202 | 0.00 | 3.36 | 4 | 12 | 51 | RNA splicing factor activity, transesterification mechanism |
| GO:0004674 | 0.00 | 2.79 | 5 | 13 | 70 | protein serine/threonine kinase activity |
| GO:0003676 | 0.00 | 1.51 | 43 | 60 | 505 | nucleic acid binding |
| GO:0003924 | 0.01 | 2.79 | 5 | 11 | 54 | GTPase activity |
| GO:0005515 | 0.01 | 1.53 | 37 | 52 | 443 | protein binding |
| GO:0004871 | 0.01 | 2.97 | 4 | 9 | 57 | signal transducer activity |


Uetz2000-1: Viable Prey Gene to GO MF Conditional test for over-representation

| GOMFID | Pvalue | OddsRatio | ExpCount | Count | Size | Term |
| GO:0005515 | 0.00 | 1.73 | 45 | 70 | 443 | protein binding |
| GO:0003702 | 0.00 | 2.37 | 13 | 26 | 123 | RNA polymerase II transcription factor activity |
| GO:0005200 | 0.00 | 3.31 | 5 | 14 | 51 | structural constituent of cytoskeleton |
| GO:0003676 | 0.00 | 1.57 | 53 | 75 | 505 | nucleic acid binding |
| GO:0030234 | 0.00 | 1.88 | 20 | 33 | 188 | enzyme regulator activity |
| GO:0031202 | 0.00 | 2.99 | 5 | 13 | 51 | RNA splicing factor activity, transesterification mechanism |
| GO:0030528 | 0.00 | 1.77 | 21 | 33 | 320 | transcription regulator activity |


Uetz2000-1: Viable Baits Gene to GO CC Conditional test for under-representation

| GOCCID | Pvalue | OddsRatio | ExpCount | Count | Size | Term |
| GO:0005739 | 0.00 | 0.53 | 88 | 53 | 1035 | mitochondrion |
| GO:0005740 | 0.00 | 0.33 | 25 | 9 | 288 | mitochondrial envelope |
| GO:0005743 | 0.00 | 0.20 | 14 | 3 | 161 | mitochondrial inner membrane |
| GO:0005842 | 0.00 | 0.12 | 7 | 1 | 87 | cytosolic large ribosomal subunit (sensu Eukaryota) |
| GO:0030312 | 0.01 | 0.22 | 8 | 2 | 99 | external encapsulating structure |
| GO:0009277 | 0.01 | 0.22 | 8 | 2 | 99 | cell wall (sensu Fungi) |
| GO:0044455 | 0.01 | 0.22 | 8 | 2 | 98 | mitochondrial membrane part |


Uetz2000-1: Viable Prey Gene to GO CC Conditional test for under-representation

| GOCCID | Pvalue | OddsRatio | ExpCount | Count | Size | Term |
| GO:0005739 | 0.00 | 0.46 | 108 | 58 | 1035 | mitochondrion |
| GO:0005740 | 0.00 | 0.39 | 30 | 13 | 288 | mitochondrial envelope |
| GO:0005743 | 0.00 | 0.27 | 17 | 5 | 161 | mitochondrial inner membrane |
| GO:0005840 | 0.00 | 0.47 | 35 | 18 | 339 | ribosome |
| GO:0044455 | 0.01 | 0.27 | 10 | 3 | 98 | mitochondrial membrane part |
| GO:0005761 | 0.01 | 0.22 | 8 | 2 | 81 | mitochondrial ribosome |
| GO:0031090 | 0.01 | 0.68 | 64 | 47 | 620 | organelle membrane |
| GO:0031980 | 0.01 | 0.44 | 17 | 8 | 163 | mitochondrial lumen |
| GO:0031975 | 0.01 | 0.62 | 41 | 27 | 391 | envelope |


Uetz2000-1: Viable Baits Gene to GO BP Conditional test for under-representation

| GOBPID | Pvalue | OddsRatio | ExpCount | Count | Size | Term |
| GO:0006412 | 0.00 | 0.46 | 32 | 16 | 372 | translation |
| GO:0009100 | 0.00 | 0.00 | 7 | 0 | 78 | glycoprotein metabolic process |
| GO:0006486 | 0.00 | 0.00 | 6 | 0 | 72 | protein amino acid glycosylation |
| GO:0008610 | 0.01 | 0.27 | 10 | 3 | 121 | lipid biosynthetic process |
| GO:0006629 | 0.01 | 0.47 | 20 | 10 | 232 | lipid metabolic process |


Uetz2000-1: Viable Prey Gene to GO BP Conditional test for under-representation

| GOBPID | Pvalue | OddsRatio | ExpCount | Count | Size | Term |
| GO:0006412 | 0.00 | 0.47 | 39 | 20 | 372 | translation |
| GO:0006812 | 0.00 | 0.09 | 10 | 1 | 92 | cation transport |
| GO:0044255 | 0.00 | 0.40 | 23 | 10 | 219 | cellular lipid metabolic process |
| GO:0044249 | 0.01 | 0.73 | 87 | 68 | 841 | cellular biosynthetic process |


Uetz2000-1: Viable Baits Gene to GO MF Conditional test for under-representation

| GOMFID | Pvalue | OddsRatio | ExpCount | Count | Size | Term |
| GO:0005215 | 0.00 | 0.42 | 35 | 16 | 408 | transporter activity |
| GO:0008324 | 0.00 | 0.17 | 11 | 2 | 125 | cation transporter activity |
| GO:0003735 | 0.00 | 0.35 | 18 | 7 | 216 | structural constituent of ribosome |
| GO:0015078 | 0.01 | 0.00 | 5 | 0 | 54 | hydrogen ion transporter activity |
| GO:0016758 | 0.01 | 0.14 | 6 | 1 | 76 | transferase activity, transferring hexosyl groups |


Uetz2000-1: Viable Prey Gene to GO MF Conditional test for under-representation

| GOMFID | Pvalue | OddsRatio | ExpCount | Count | Size | Term |
| GO:0005215 | 0.00 | 0.40 | 42 | 19 | 408 | transporter activity |
| GO:0008324 | 0.00 | 0.21 | 13 | 3 | 125 | cation transporter activity |
| GO:0003735 | 0.00 | 0.41 | 22 | 10 | 216 | structural constituent of ribosome |
| GO:0003824 | 0.01 | 0.79 | 199 | 171 | 1907 | catalytic activity |
