# Supplementary material for: Coverage and error models of protein-protein interaction data by directed graph analysis
Source: Genome Biol. 2007 Sep 10;8(9):R186. doi: 10.1186/gb-2007-8-9-r186 (PMC2375024; doi:10.1186/gb-2007-8-9-r186)
Supplement: Additional data file 2 — Presented is the Bioconductor package ppiStats (version 1.3.5 of 22 June 2007) in 'source' format. ppiStats contains the novel methods developed in this paper. [file gb-2007-8-9-r186-S2.gz › ppiStats/inst/Scripts/Uetz2000-2.html]

Uetz2000-2: Viable Baits Gene to GO CC Conditional test for over-representation

| GOCCID | Pvalue | OddsRatio | ExpCount | Count | Size | Term |
| GO:0030863 | 0.00 | 18.94 | 1 | 15 | 51 | cortical cytoskeleton |
| GO:0015629 | 0.00 | 13.04 | 2 | 17 | 77 | actin cytoskeleton |
| GO:0005935 | 0.00 | 7.36 | 2 | 14 | 112 | bud neck |
| GO:0005934 | 0.00 | 9.51 | 1 | 9 | 50 | bud tip |
| GO:0005623 | 0.00 | 4.74 | 118 | 134 | 4954 | cell |
| GO:0043228 | 0.00 | 2.01 | 22 | 38 | 931 | non-membrane-bound organelle |
| GO:0005856 | 0.00 | 3.68 | 3 | 10 | 204 | cytoskeleton |
| GO:0044448 | 0.00 | 6.38 | 1 | 5 | 89 | cell cortex part |
| GO:0005622 | 0.00 | 1.99 | 109 | 122 | 4563 | intracellular |


Uetz2000-2: Viable Prey Gene to GO CC Conditional test for over-representation

| GOCCID | Pvalue | OddsRatio | ExpCount | Count | Size | Term |
| GO:0005935 | 0.00 | 5.04 | 7 | 25 | 112 | bud neck |
| GO:0030863 | 0.00 | 7.35 | 3 | 17 | 51 | cortical cytoskeleton |
| GO:0015629 | 0.00 | 5.91 | 5 | 20 | 77 | actin cytoskeleton |
| GO:0005934 | 0.00 | 5.11 | 3 | 13 | 50 | bud tip |
| GO:0005622 | 0.00 | 1.69 | 301 | 329 | 4563 | intracellular |
| GO:0044448 | 0.00 | 4.80 | 2 | 9 | 89 | cell cortex part |
| GO:0005623 | 0.00 | 1.73 | 327 | 348 | 4954 | cell |


Uetz2000-2: Viable Baits Gene to GO BP Conditional test for over-representation

| GOBPID | Pvalue | OddsRatio | ExpCount | Count | Size | Term |
| GO:0030468 | 0.00 | 21.41 | 2 | 30 | 102 | establishment of cell polarity (sensu Fungi) |
| GO:0007163 | 0.00 | 18.32 | 3 | 30 | 114 | establishment and/or maintenance of cell polarity |
| GO:0009653 | 0.00 | 9.84 | 6 | 38 | 247 | anatomical structure morphogenesis |
| GO:0065007 | 0.00 | 4.04 | 19 | 52 | 783 | biological regulation |
| GO:0000278 | 0.00 | 6.06 | 6 | 27 | 244 | mitotic cell cycle |
| GO:0007264 | 0.00 | 14.01 | 1 | 14 | 59 | small GTPase mediated signal transduction |
| GO:0000910 | 0.00 | 9.27 | 2 | 17 | 101 | cytokinesis |
| GO:0007105 | 0.00 | 12.35 | 2 | 14 | 65 | cytokinesis, site selection |
| GO:0050794 | 0.00 | 6.40 | 4 | 22 | 678 | regulation of cellular process |
| GO:0022402 | 0.00 | 4.62 | 8 | 29 | 399 | cell cycle process |
| GO:0000003 | 0.00 | 17.33 | 1 | 11 | 306 | reproduction |
| GO:0030029 | 0.00 | 7.72 | 3 | 16 | 110 | actin filament-based process |
| GO:0007165 | 0.00 | 6.24 | 3 | 17 | 193 | signal transduction |
| GO:0050896 | 0.00 | 3.00 | 17 | 40 | 713 | response to stimulus |
| GO:0051325 | 0.00 | 7.31 | 2 | 13 | 92 | interphase |
| GO:0000075 | 0.00 | 10.15 | 1 | 10 | 53 | cell cycle checkpoint |
| GO:0006974 | 0.00 | 10.84 | 1 | 9 | 226 | response to DNA damage stimulus |
| GO:0007015 | 0.00 | 8.55 | 1 | 10 | 61 | actin filament organization |
| GO:0000279 | 0.00 | 4.00 | 6 | 20 | 249 | M phase |
| GO:0022414 | 0.00 | 3.69 | 6 | 20 | 267 | reproductive process |
| GO:0016043 | 0.00 | 2.64 | 16 | 34 | 2008 | cell organization and biogenesis |
| GO:0007114 | 0.00 | 11.09 | 1 | 7 | 80 | cell budding |
| GO:0022413 | 0.00 | 3.67 | 6 | 18 | 239 | reproductive process in single-celled organism |
| GO:0006996 | 0.00 | 2.18 | 30 | 52 | 1272 | organelle organization and biogenesis |
| GO:0007067 | 0.00 | 4.65 | 3 | 12 | 125 | mitosis |
| GO:0006970 | 0.00 | 6.27 | 2 | 9 | 71 | response to osmotic stress |
| GO:0016044 | 0.00 | 3.88 | 4 | 14 | 173 | membrane organization and biogenesis |
| GO:0000074 | 0.00 | 4.98 | 2 | 10 | 162 | regulation of progression through cell cycle |
| GO:0006511 | 0.00 | 3.91 | 3 | 12 | 146 | ubiquitin-dependent protein catabolic process |
| GO:0051603 | 0.00 | 3.85 | 4 | 12 | 148 | proteolysis involved in cellular protein catabolic process |
| GO:0043632 | 0.00 | 3.71 | 4 | 12 | 153 | modification-dependent macromolecule catabolic process |
| GO:0000746 | 0.00 | 4.19 | 3 | 10 | 113 | conjugation |
| GO:0019953 | 0.00 | 4.19 | 3 | 10 | 113 | sexual reproduction |
| GO:0006897 | 0.00 | 4.62 | 2 | 8 | 82 | endocytosis |
| GO:0030163 | 0.00 | 3.28 | 4 | 12 | 171 | protein catabolic process |
| GO:0051301 | 0.00 | 2.91 | 5 | 14 | 325 | cell division |
| GO:0008361 | 0.00 | 3.62 | 3 | 10 | 129 | regulation of cell size |
| GO:0019236 | 0.00 | 4.27 | 2 | 8 | 88 | response to pheromone |
| GO:0031323 | 0.00 | 2.25 | 11 | 22 | 459 | regulation of cellular metabolic process |
| GO:0045449 | 0.00 | 2.39 | 8 | 18 | 350 | regulation of transcription |
| GO:0006468 | 0.00 | 4.02 | 2 | 8 | 93 | protein amino acid phosphorylation |
| GO:0048523 | 0.00 | 2.75 | 5 | 13 | 218 | negative regulation of cellular process |
| GO:0046903 | 0.00 | 2.64 | 6 | 14 | 245 | secretion |
| GO:0007017 | 0.00 | 3.75 | 2 | 8 | 99 | microtubule-based process |
| GO:0007001 | 0.00 | 2.04 | 13 | 24 | 551 | chromosome organization and biogenesis (sensu Eukaryota) |
| GO:0032446 | 0.00 | 4.13 | 2 | 7 | 79 | protein modification by small protein conjugation |
| GO:0006796 | 0.00 | 2.77 | 5 | 12 | 199 | phosphate metabolic process |
| GO:0007124 | 0.00 | 4.45 | 2 | 6 | 63 | pseudohyphal growth |
| GO:0006281 | 0.00 | 2.78 | 4 | 11 | 181 | DNA repair |
| GO:0043283 | 0.00 | 1.62 | 43 | 58 | 1800 | biopolymer metabolic process |
| GO:0006310 | 0.00 | 3.34 | 3 | 8 | 110 | DNA recombination |
| GO:0000723 | 0.00 | 2.38 | 6 | 14 | 269 | telomere maintenance |
| GO:0051704 | 0.00 | 10.41 | 0 | 3 | 128 | multi-organism process |
| GO:0044267 | 0.01 | 1.67 | 27 | 40 | 1143 | cellular protein metabolic process |
| GO:0051169 | 0.01 | 3.18 | 3 | 8 | 115 | nuclear transport |
| GO:0007049 | 0.01 | 8.92 | 0 | 3 | 417 | cell cycle |
| GO:0031497 | 0.01 | 3.29 | 2 | 7 | 97 | chromatin assembly |
| GO:0006913 | 0.01 | 2.98 | 3 | 8 | 122 | nucleocytoplasmic transport |


Uetz2000-2: Viable Prey Gene to GO BP Conditional test for over-representation

| GOBPID | Pvalue | OddsRatio | ExpCount | Count | Size | Term |
| GO:0065007 | 0.00 | 2.23 | 52 | 94 | 783 | biological regulation |
| GO:0007163 | 0.00 | 4.64 | 8 | 27 | 114 | establishment and/or maintenance of cell polarity |
| GO:0030468 | 0.00 | 4.84 | 7 | 25 | 102 | establishment of cell polarity (sensu Fungi) |
| GO:0051704 | 0.00 | 3.60 | 8 | 25 | 128 | multi-organism process |
| GO:0000910 | 0.00 | 3.87 | 7 | 21 | 101 | cytokinesis |
| GO:0051726 | 0.00 | 3.11 | 11 | 28 | 162 | regulation of cell cycle |
| GO:0048610 | 0.00 | 2.66 | 16 | 36 | 239 | reproductive cellular process |
| GO:0019236 | 0.00 | 4.04 | 6 | 19 | 88 | response to pheromone |
| GO:0009653 | 0.00 | 2.48 | 16 | 34 | 247 | anatomical structure morphogenesis |
| GO:0016043 | 0.00 | 1.57 | 133 | 171 | 2008 | cell organization and biogenesis |
| GO:0000278 | 0.00 | 3.18 | 8 | 21 | 244 | mitotic cell cycle |
| GO:0000075 | 0.00 | 4.24 | 4 | 12 | 53 | cell cycle checkpoint |
| GO:0048523 | 0.00 | 2.26 | 14 | 29 | 218 | negative regulation of cellular process |
| GO:0007114 | 0.00 | 4.30 | 3 | 11 | 80 | cell budding |
| GO:0022402 | 0.00 | 6.06 | 2 | 8 | 399 | cell cycle process |
| GO:0007105 | 0.00 | 3.62 | 4 | 13 | 65 | cytokinesis, site selection |
| GO:0007015 | 0.00 | 3.54 | 4 | 12 | 61 | actin filament organization |
| GO:0030036 | 0.00 | 4.12 | 3 | 10 | 106 | actin cytoskeleton organization and biogenesis |
| GO:0006974 | 0.00 | 2.08 | 15 | 28 | 226 | response to DNA damage stimulus |
| GO:0006468 | 0.00 | 2.79 | 6 | 15 | 93 | protein amino acid phosphorylation |
| GO:0000747 | 0.00 | 2.78 | 6 | 14 | 113 | conjugation with cellular fusion |
| GO:0019219 | 0.00 | 1.71 | 26 | 41 | 396 | regulation of nucleobase, nucleoside, nucleotide and nucleic acid metabolic process |
| GO:0051640 | 0.00 | 3.27 | 4 | 10 | 54 | organelle localization |
| GO:0006897 | 0.00 | 2.72 | 5 | 13 | 82 | endocytosis |
| GO:0032505 | 0.00 | 2.01 | 13 | 24 | 278 | reproduction of a single-celled organism |
| GO:0051325 | 0.00 | 2.60 | 6 | 14 | 92 | interphase |
| GO:0007010 | 0.00 | 2.41 | 7 | 15 | 220 | cytoskeleton organization and biogenesis |
| GO:0000087 | 0.00 | 2.26 | 8 | 17 | 126 | M phase of mitotic cell cycle |
| GO:0050794 | 0.00 | 1.73 | 22 | 35 | 678 | regulation of cellular process |
| GO:0006351 | 0.00 | 1.60 | 31 | 46 | 471 | transcription, DNA-dependent |
| GO:0007017 | 0.01 | 2.38 | 7 | 14 | 99 | microtubule-based process |
| GO:0043283 | 0.01 | 1.33 | 119 | 142 | 1800 | biopolymer metabolic process |
| GO:0007154 | 0.01 | 1.85 | 15 | 25 | 222 | cell communication |
| GO:0022403 | 0.01 | 1.80 | 16 | 26 | 328 | cell cycle phase |
| GO:0007242 | 0.01 | 2.16 | 8 | 16 | 123 | intracellular signaling cascade |
| GO:0009892 | 0.01 | 1.89 | 13 | 22 | 191 | negative regulation of metabolic process |
| GO:0016481 | 0.01 | 2.04 | 10 | 18 | 146 | negative regulation of transcription |
| GO:0050896 | 0.01 | 1.47 | 40 | 55 | 713 | response to stimulus |
| GO:0009628 | 0.01 | 2.22 | 7 | 14 | 105 | response to abiotic stimulus |


Uetz2000-2: Viable Baits Gene to GO MF Conditional test for over-representation

| GOMFID | Pvalue | OddsRatio | ExpCount | Count | Size | Term |
| GO:0003924 | 0.00 | 11.41 | 1 | 11 | 54 | GTPase activity |
| GO:0008092 | 0.00 | 9.19 | 1 | 9 | 52 | cytoskeletal protein binding |
| GO:0004871 | 0.00 | 7.11 | 1 | 8 | 57 | signal transducer activity |
| GO:0008047 | 0.00 | 5.99 | 2 | 8 | 66 | enzyme activator activity |
| GO:0004672 | 0.00 | 4.14 | 3 | 11 | 128 | protein kinase activity |
| GO:0005515 | 0.00 | 2.37 | 9 | 19 | 443 | protein binding |
| GO:0003677 | 0.00 | 2.68 | 5 | 13 | 226 | DNA binding |
| GO:0005488 | 0.00 | 1.93 | 14 | 25 | 1056 | binding |
| GO:0005200 | 0.01 | 4.63 | 1 | 5 | 51 | structural constituent of cytoskeleton |
| GO:0005083 | 0.01 | 5.65 | 1 | 4 | 54 | small GTPase regulator activity |


Uetz2000-2: Viable Prey Gene to GO MF Conditional test for over-representation

| GOMFID | Pvalue | OddsRatio | ExpCount | Count | Size | Term |
| GO:0008092 | 0.00 | 4.96 | 3 | 12 | 52 | cytoskeletal protein binding |
| GO:0005200 | 0.00 | 3.51 | 3 | 10 | 51 | structural constituent of cytoskeleton |
| GO:0005515 | 0.00 | 1.69 | 24 | 38 | 443 | protein binding |
| GO:0004672 | 0.00 | 2.21 | 8 | 17 | 128 | protein kinase activity |
| GO:0030234 | 0.01 | 1.92 | 12 | 22 | 188 | enzyme regulator activity |


Uetz2000-2: Viable Baits Gene to GO CC Conditional test for under-representation

| GOCCID | Pvalue | OddsRatio | ExpCount | Count | Size | Term |
| GO:0005739 | 0.00 | 0.12 | 14 | 2 | 1035 | mitochondrion |
| GO:0043233 | 0.00 | 0.31 | 18 | 6 | 736 | organelle lumen |
| GO:0044429 | 0.00 | 0.18 | 10 | 2 | 438 | mitochondrial part |
| GO:0005773 | 0.01 | 0.00 | 5 | 0 | 194 | vacuole |


Uetz2000-2: Viable Prey Gene to GO CC Conditional test for under-representation

| GOCCID | Pvalue | OddsRatio | ExpCount | Count | Size | Term |
| GO:0005739 | 0.00 | 0.54 | 39 | 23 | 1035 | mitochondrion |
| GO:0044429 | 0.00 | 0.52 | 29 | 16 | 438 | mitochondrial part |
| GO:0005840 | 0.00 | 0.46 | 22 | 11 | 339 | ribosome |


Uetz2000-2: Viable Baits Gene to GO BP Conditional test for under-representation

| GOBPID | Pvalue | OddsRatio | ExpCount | Count | Size | Term |
| GO:0044249 | 0.00 | 0.35 | 20 | 8 | 841 | cellular biosynthetic process |
| GO:0019752 | 0.00 | 0.13 | 7 | 1 | 307 | carboxylic acid metabolic process |
| GO:0015980 | 0.01 | 0.00 | 5 | 0 | 197 | energy derivation by oxidation of organic compounds |


Uetz2000-2: Viable Prey Gene to GO BP Conditional test for under-representation

| GOBPID | Pvalue | OddsRatio | ExpCount | Count | Size | Term |
| GO:0006082 | 0.00 | 0.32 | 20 | 7 | 307 | organic acid metabolic process |
| GO:0006732 | 0.00 | 0.10 | 9 | 1 | 135 | coenzyme metabolic process |
| GO:0006412 | 0.00 | 0.41 | 25 | 11 | 372 | translation |
| GO:0006807 | 0.00 | 0.35 | 16 | 6 | 242 | nitrogen compound metabolic process |
| GO:0051188 | 0.00 | 0.00 | 5 | 0 | 78 | cofactor biosynthetic process |
| GO:0044249 | 0.00 | 0.63 | 50 | 34 | 841 | cellular biosynthetic process |
| GO:0006520 | 0.01 | 0.31 | 12 | 4 | 184 | amino acid metabolic process |


Uetz2000-2: Viable Baits Gene to GO MF Conditional test for under-representation

| GOMFID | Pvalue | OddsRatio | ExpCount | Count | Size | Term |
| GO:0016491 | 0.00 | 0.00 | 6 | 0 | 264 | oxidoreductase activity |
