# Supplementary material for: Coverage and error models of protein-protein interaction data by directed graph analysis
Source: Genome Biol. 2007 Sep 10;8(9):R186. doi: 10.1186/gb-2007-8-9-r186 (PMC2375024; doi:10.1186/gb-2007-8-9-r186)
Supplement: Additional data file 3 — Presented is the Bioconductor package ppiStats in 'Windows binary' format. [file gb-2007-8-9-r186-S3.zip › ppiStats/html/00Index.html]

R: Protein-Protein Interaction Statistical Package

# Protein-Protein Interaction Statistical Package

---

## Documentation for package `ppiStats' version 1.3.5

## User Guides and Package Vignettes

Read overview or browse directory.

## Help Pages

|  |  |
| --- | --- |
| assessSymmetry | A function that calcualtes some directed degree statistics on graphs using a binomial error model |
| bpMatrix | This function generates the Bait-Prey Matrix for an protein-protein interaction (ppi) experiment |
| calcInOutDegStats | This function calculates the various degree statistic for a Protein-Protein Interaction (PPI) Graph |
| estErrProbMethodOfMoments | Estimate false positive and false negative error probabilities by method moments. |
| genBPGraph | A function to generate the protein-protein interaction (ppi) induced (un)directed graph |
| idHomodimers | A function to determine viable baits, viable preys, or homodimers within experimental data-sets. |
| idStochastic | A function to identify those proteins affected by either stochastic or systematic errors |
| idSystematic | A function to identify those proteins affected by either stochastic or systematic errors |
| idViableProteins | A function to determine viable baits, viable preys, or homodimers within experimental data-sets. |
| makeBinVect | A function to compute a permutation test on a two way table.h |
| nullDistDoublyTestedEdges | Null distribution of number of reciprocated, unreciprocated and missing edges in stochastic model. |
| ppiBuildParams4GO | A wrapper function to build a parameter class for the input of the HyperGTest. |
| ppiBuildParams4PFAM | A wrapper function to build a parameter class for the input of the HyperGTest. |
| ppiHGTest4GO | A wrapper function to implement the Hypergeometric test, HyperGTest found withing the Category and GOstats packages. |
| ppiHGTest4PFAM | A wrapper function to implement the Hypergeometric test, HyperGTest found withing the Category and GOstats packages. |
| twowayPERM | A function to compute a permutation test on a two way table.h |
| twPERM | A function to compute a permutation test on a two way table.h |
