# Supplementary material for: Coverage and error models of protein-protein interaction data by directed graph analysis
Source: Genome Biol. 2007 Sep 10;8(9):R186. doi: 10.1186/gb-2007-8-9-r186 (PMC2375024; doi:10.1186/gb-2007-8-9-r186)
Supplement: Additional data file 3 — Presented is the Bioconductor package ppiStats in 'Windows binary' format. [file gb-2007-8-9-r186-S3.zip › ppiStats/html/assessSymmetry.html]

R: A function that calcualtes some directed degree statistics on
graphs using a binomial error model

|  |  |
| --- | --- |
| assessSymmetry {ppiStats} | R Documentation |

## A function that calcualtes some directed degree statistics on graphs using a binomial error model

### Description

This function takes in a bait to prey protein-protein interaction
graph (undirected) and calculates the reciprocated degree, the
unreciprocated in and out degrees. Using this information and binomial
error model, it asses the p-value for the in and out degree of each
protein. Lastly, it plots the countour curves for these p-values

### Usage

```
assessSymmetry(bpMat, bpGraph = FALSE,
  prob=0.5, pLevels = 1e-4)
```

### Arguments

|  |  |
| --- | --- |
| `bpMat` | Either a bait to prey directed graphNEL or its corresponding adjacency matrix. |
| `bpGraph` | A logical. If TRUE, than bpMat is passed in by the user as a graphNEL. |
| `pLevels` | A numeric vector. It gives the levels to calculate the countours of the function in p in the (n-in, n-out)-plane |
| `prob` | A numeric. The bias of the coin used in the function pbinom call. |

### Value

A list:

|  |  |
| --- | --- |
| `deg` | A 3xn matrix. The rows are indexed by each protein. Column one gives the number of reciprocated edges; column two gives the number of unreciprocated out edges; colunm three gives the number of unreciprocated in-edges |
| `p` | The p-value for each protein with experimental in and out degrees |
| `countours` | The countours as a function of p |

### Author(s)

W Huber

### Examples

```
library(ppiData)
assessSymmetry(Ito2001BPGraph, bpGraph=TRUE)
```

---

[Package *ppiStats* version 1.3.5 Index]
