# Supplementary material for: Coverage and error models of protein-protein interaction data by directed graph analysis
Source: Genome Biol. 2007 Sep 10;8(9):R186. doi: 10.1186/gb-2007-8-9-r186 (PMC2375024; doi:10.1186/gb-2007-8-9-r186)
Supplement: Additional data file 3 — Presented is the Bioconductor package ppiStats in 'Windows binary' format. [file gb-2007-8-9-r186-S3.zip › ppiStats/html/bpMatrix.html]

R: This function generates the Bait-Prey Matrix for an
protein-protein interaction (ppi) experiment

|  |  |
| --- | --- |
| bpMatrix {ppiStats} | R Documentation |

## This function generates the Bait-Prey Matrix for an protein-protein interaction (ppi) experiment

### Description

This function takes in experimental ppi data and generates the bait to
prey adjacency matrix.

### Usage

```
bpMatrix(y2h, symMat = TRUE, homodimer = FALSE, baitAsPrey = FALSE, 
              unWeighted=TRUE, onlyRecip=FALSE, baitsOnly=FALSE)
```

### Arguments

|  |  |
| --- | --- |
| `y2h` | A named list of character vectors. The names of the list correspond to the baits used in the experimental technology. The entries of the character vectors are those proteins found as prey or a character(0) if the bait did not find any prey. |
| `symMat` | A logical, if TRUE, the matrix will be square with all the proteins documented in the experiment indexing both the row and column; if FALSE, only the baits index the rows, preys the columns. |
| `homodimer` | A logical. If TRUE, the matrix will record the presence of homodimers; if FALSE, all homodimers data will be deleted. |
| `baitAsPrey` | A logical; if TRUE, the columns will be indexed by both the bait and prey population while the rows will remain indexed by the baits exclusively. |
| `unWeighted` | A logical. If TRUE, the entries of the adjacency will be binary (0,1) which records the presence of interactions or not. If FALSE, the entries of the matrix will be a natrual number to record the multiplicity of the interaction found by the experiment. |
| `onlyRecip` | A logical. If TRUE, the adjacency matrix will be restricted to only those interactions which are symmetric. |
| `baitsOnly` | A logical. If TRUE, the matrix will be indexed by the baits for both the rows and columns. If baitsOnly is TRUE, then baitsAsPrey must also be TRUE and symMat must be FALSE |

### Details

It is important to note that the weight of each directed edge is
recorded by the number of instances bait b finds prey p.

### Value

An adjacency matrix with weighting. The rows are indexed by those
proteins sampled as baits (if symMat is true, only those rows with
non-trivial row sums were sampled as baits) and the colunms are
indexed by proteins detected as baits (et cetera).

### Author(s)

T Chiang

### Examples

```
library(ppiData)
data(y2hSysGW)
eg <- y2hSysGW[2:4]
lapply(eg, bpMatrix)
```

---

[Package *ppiStats* version 1.3.5 Index]
