# Supplementary material for: Coverage and error models of protein-protein interaction data by directed graph analysis
Source: Genome Biol. 2007 Sep 10;8(9):R186. doi: 10.1186/gb-2007-8-9-r186 (PMC2375024; doi:10.1186/gb-2007-8-9-r186)
Supplement: Additional data file 3 — Presented is the Bioconductor package ppiStats in 'Windows binary' format. [file gb-2007-8-9-r186-S3.zip › ppiStats/html/calcInOutDegStats.html]

R: This function calculates the various degree statistic for a
Protein-Protein Interaction (PPI) Graph

|  |  |
| --- | --- |
| calcInOutDegStats {ppiStats} | R Documentation |

## This function calculates the various degree statistic for a Protein-Protein Interaction (PPI) Graph

### Description

This function takes a graphNEL argument and calculates several degree
statistics for which further analysis can be made.

### Usage

```
calcInOutDegStats(graphObj, homodimer=FALSE)
```

### Arguments

|  |  |
| --- | --- |
| `graphObj` | The argument `graphObj` is an instance of the class graphNEL. |
| `homodimer` | A logical. If FALSE, the function removes all homodimer relationships. It retains them otherwise. |

### Value

The return value is a list of various degree statistics of the PPI directed
graph:

|  |  |
| --- | --- |
| `inDegree` | A named numeric vector. The names corresponds to the particular yeast Open Reading Frame (ORF), and each entry details how many edges flow towards that node, i.e. how many times each node was detected as a prey. |
| `outDegree` | A named numeric vector. The names corresponds to the particular yeast Open Reading Frame (ORF), and each entry details how many edges flow out from that node, i.e. how many prey was detected by each node as a bait. |
| `inDegreeMinusOutDegree` | A named numeric vector. This entry is the inDegree minus the outDegree entries, or the signed difference for each node as a prey to each node as a bait. |
| `outDegreeMinusInDegree` | A named numeric vector. This entry is the outDegree minus the inDegree entries, or the signed difference for each node as a bait to each node as a prey. |
| `recipInDegree` | A named numeric vector. Again the names represents the ORFs, while each entry corresponds to how many times each node was found as a prey in a reciprocated interaction. |
| `recipOutDegree` | A named numeric vector. Again the names represents the ORFs, while each entry corresponds to how many times each node was found as a bait in a reciprocated interaction. |
| `totalRecipDegree` | A numeric. For the PPI graph given, this argument gives the total number of reciprocated interactions found. |
| `unrecipInDegree` | A named numeric vector. The names corresponds ORF and each entry details how many edges flow towards each node modulo the reciprocated interactions, i.e. for each node $p$, how many baits $b$ detected $p$ as a prey with the condition that $p$ never detects $b$ as a prey. |
| `unrecipOutDegree` | A named numeric vector. The names corresponds ORF and each entry details how many edges flow out from each node modulo the reciprocated interactions, i.e. for each node $b$, how many prey $p$ does $b$ detect with the condition that $b$ is never detected by $p$. |
| `totalUnrecipDegree` | A numeric. The total number of unreciprocated interactions for the PPI graph given. |

...

### Author(s)

T Chiang

### Examples

```
        library(ppiData)
        degStat <- calcInOutDegStats(Cagney2001BPGraph)
        degStat$recipOutDeg
```

---

[Package *ppiStats* version 1.3.5 Index]
