# Supplementary material for: Coverage and error models of protein-protein interaction data by directed graph analysis
Source: Genome Biol. 2007 Sep 10;8(9):R186. doi: 10.1186/gb-2007-8-9-r186 (PMC2375024; doi:10.1186/gb-2007-8-9-r186)
Supplement: Additional data file 3 — Presented is the Bioconductor package ppiStats in 'Windows binary' format. [file gb-2007-8-9-r186-S3.zip › ppiStats/html/estErrProbMethodOfMoments.html]

R: Estimate false positive and false negative error probabilities
by method moments.

|  |  |
| --- | --- |
| estErrProbMethodOfMoments {ppiStats} | R Documentation |

## Estimate false positive and false negative error probabilities by method moments.

### Description

Estimate false positive and false negative error probabilities
by method moments.

### Usage

```
estErrProbMethodOfMoments(nint, nrec, nunr, ntot)
```

### Arguments

|  |  |
| --- | --- |
| `nint` | Integer vector. True number of interactions. Typically, the function is called for a range of these, returning all possible solutions for that range. |
| `nrec` | Integer scalar. Observed number of reciprocated edges. |
| `nunr` | Integer scalar. Observed number of unreciprocated edges. |
| `ntot` | Integer scalar. Number of proteins which were tested twice (e.g. both as viable bait and as viable prey). |

### Details

The model is described in the vignette
*Stochastic and systematic errors in PPI data, by looking
at unreciprocated in- or out-edges*
by W. Huber, T. Chiang and R. Gentleman.

### Value

Matrix with 5 columns `nint` (a copy of the input argument),
`pfp1`, `pfn1`, `pfp2` and `pfn2`, and as many
rows as the length of `nint`.

### Author(s)

Wolfgang Huber http://www.ebi.ac.uk/huber

### Examples

```
est = estErrProbMethodOfMoments(nint=seq(8000, 40000, by=100), nrec=9722, nunr=15856, ntot=2000)
if(interactive()) {
  plot(est[, c("pfp2", "pfn2")], type="l", col="blue", lwd=2,
       xlab=expression(p[FP]), ylab=expression(p[FN]))
  abline(h=0, v=0, lty=2)
}
```

---

[Package *ppiStats* version 1.3.5 Index]
