# Supplementary material for: Coverage and error models of protein-protein interaction data by directed graph analysis
Source: Genome Biol. 2007 Sep 10;8(9):R186. doi: 10.1186/gb-2007-8-9-r186 (PMC2375024; doi:10.1186/gb-2007-8-9-r186)
Supplement: Additional data file 3 — Presented is the Bioconductor package ppiStats in 'Windows binary' format. [file gb-2007-8-9-r186-S3.zip › ppiStats/html/genBPGraph.html]

R: A function to generate the protein-protein interaction (ppi)
induced (un)directed graph

|  |  |
| --- | --- |
| genBPGraph {ppiStats} | R Documentation |

## A function to generate the protein-protein interaction (ppi) induced (un)directed graph

### Description

This function will take the ppi data and generate instance of the
class graph.

### Usage

```
genBPGraph(bpMat, directed=TRUE, bp=TRUE)
```

### Arguments

|  |  |
| --- | --- |
| `bpMat` | An adjacency matrix of PPI. If the matrix is obtained by empirical data, or bait to prey, then the rows are indexed by the baits and the columns indexed by the preys. If the rownames are not the same as the column names (i.e. a generic bait to prey matrix) the argument bp must be set to TRUE. |
| `directed` | A logical - if TRUE, the object will be a directed graph rather than an ordinary graph. For bait to prey interactions, this parameter must always be set to TRUE. |
| `bp` | A logical - if TRUE, it signifies that the adjacency matrix is a bait to prey empirically derived matrix so that the bait population (rownames) is usually different from the prey population (colnames). |

### Value

An instance of the class graph.

### Author(s)

T Chiang

### Examples

```
library(ppiData)
library(graph)
data(y2hSysGW)
eg=y2hSysGW[[3]]
egMat = bpMatrix(eg)
genBPGraph(egMat)
```

---

[Package *ppiStats* version 1.3.5 Index]
