# Supplementary material for: Coverage and error models of protein-protein interaction data by directed graph analysis
Source: Genome Biol. 2007 Sep 10;8(9):R186. doi: 10.1186/gb-2007-8-9-r186 (PMC2375024; doi:10.1186/gb-2007-8-9-r186)
Supplement: Additional data file 3 — Presented is the Bioconductor package ppiStats in 'Windows binary' format. [file gb-2007-8-9-r186-S3.zip › ppiStats/html/hgParams.html]

R: A wrapper function to build a parameter class for the
input of the HyperGTest.

|  |  |
| --- | --- |
| hgParams {ppiStats} | R Documentation |

## A wrapper function to build a parameter class for the input of the HyperGTest.

### Description

This function takes a gene set and conducts test for either over or under
representation of some category using the Hypergeometric distribution.

The two differences when building the parameter classes are that a conditional
test can be performed on the GO dag but not on PFAM categories and an ontology
can be assigned to GO but not to PFAM.

### Usage

```
ppiBuildParams4GO(geneSet, universe, direction="over", annot="YEAST",
                           ontology = "CC", cond=TRUE, pThresh = 0.01)
ppiBuildParams4PFAM(geneSet, universe, annot ="YEAST",
                              direction = "over", pThresh=0.01)
```

### Arguments

|  |  |
| --- | --- |
| `geneSet` | A character vector of genes given by the gene locus name. |
| `universe` | The set of genes by which the geneSet is tested against for over/under representation. The genes are also given by the gene locus names. |
| `direction` | A character. This parameter can be either set to over or under when testing for GO categories. |
| `annot` | A character. The annotation package used. |
| `ontology` | A character: either CC, MF, or BP to describe the GO ontology. |
| `cond` | A logical. To test within the CO dag, a conditional hypergeometric test can be conducted. |
| `pThresh` | A numeric. A p-value threshold by which the null hypothesis is rejected. |

### Value

A object of class hyperGParams.

### Author(s)

T Chiang

### References

### Examples

```

```

---

[Package *ppiStats* version 1.3.5 Index]
