# Supplementary material for: Coverage and error models of protein-protein interaction data by directed graph analysis
Source: Genome Biol. 2007 Sep 10;8(9):R186. doi: 10.1186/gb-2007-8-9-r186 (PMC2375024; doi:10.1186/gb-2007-8-9-r186)
Supplement: Additional data file 3 — Presented is the Bioconductor package ppiStats in 'Windows binary' format. [file gb-2007-8-9-r186-S3.zip › ppiStats/html/hgTests.html]

R: A wrapper function to implement the Hypergeometric test,
HyperGTest found withing the Category and GOstats packages.

|  |  |
| --- | --- |
| hgTests {ppiStats} | R Documentation |

## A wrapper function to implement the Hypergeometric test, HyperGTest found withing the Category and GOstats packages.

### Description

This function takes the instances of the hyperGParams and conducts
a (conditional) test for over/under representation of some category
under the Hypergeometric distribution.

### Usage

```
ppiHGTest4GO(parameter, filename, append=TRUE,
                      label = "Experiment name here",
                      typeGeneSet = "Describe the gene set here",
                      cs=50)

ppiHGTest4PFAM(parameter, filename, append = TRUE,
                           label = "Experiment Name Here",
                           typeGeneSet = "Describe the Gene Set Here",
                           cs = 50)
```

### Arguments

|  |  |
| --- | --- |
| `parameter` | An object of hyperGParams. |
| `filename` | A character vector. The name given to the .html file produced. |
| `append` | A logical. If multiple tests are conducted using the same filename, then the .html file will be appended if TRUE or over-written if FALSE. |
| `label` | A character. The should give a description of the experiment used to obtain the gene set. |
| `typeGeneSet` | A character: this character vector should adequately describe the gene set itself. |
| `cs` | A numeric. This gives a cut-off for the size of the categories in which we conduct the test. |

### Value

A instance of the class hyperGTest as well as an .html file.

### Author(s)

T Chiang

### References

### Examples

```

```

---

[Package *ppiStats* version 1.3.5 Index]
