# Supplementary material for: Coverage and error models of protein-protein interaction data by directed graph analysis
Source: Genome Biol. 2007 Sep 10;8(9):R186. doi: 10.1186/gb-2007-8-9-r186 (PMC2375024; doi:10.1186/gb-2007-8-9-r186)
Supplement: Additional data file 3 — Presented is the Bioconductor package ppiStats in 'Windows binary' format. [file gb-2007-8-9-r186-S3.zip › ppiStats/html/idProteinErrorType.html]

R: A function to identify those proteins affected by either
stochastic or systematic errors

|  |  |
| --- | --- |
| idProteinErrorType {ppiStats} | R Documentation |

## A function to identify those proteins affected by either stochastic or systematic errors

### Description

This function takes in either a bait to prey Graph (matrix) and,
based on a binomial error model, partitions proteins identified as
either affected by systematic or stochastic error. It is a wrapper
function that will eventually call the qbinom function.

### Usage

```
idSystematic(bpMat, viable, bpGraph = FALSE, pThresh = 0.01, pLevels =
1e-4, prob=0.5)
idStochastic(bpMat, bpGraph = FALSE, pThresh = 0.01, pLevels =
1e-4, prob=0.5)
```

### Arguments

|  |  |
| --- | --- |
| `bpMat` | Either a bait to prey directed graphNEL or its corresponding adjacency matrix. |
| `viable` | This is a character vector of viable proteins. It is only used in the idSystematic function. |
| `bpGraph` | A logical. If TRUE, than bpMat is passed in by the user as a graphNEL. |
| `pThresh` | The p-value threshold for which to partition stochastic or systematic errors |
| `pLevels` | A numeric. It gives the levels to calculate the countours of the function in p in the (n-in, n-out)-plane |
| `prob` | A numeric. The probability parameter in the call to the qbinom function. |

### Value

A character vector of proteins either affected by systematic or
stochastic errors.

### Author(s)

T Chiang

### References

~put references to the literature/web site here ~

### Examples

```
library(ppiData)
idSystematic(Ito2001BPGraph, viableBaits[[1]], bpGraph=TRUE)
```

---

[Package *ppiStats* version 1.3.5 Index]
