# Supplementary material for: Coverage and error models of protein-protein interaction data by directed graph analysis
Source: Genome Biol. 2007 Sep 10;8(9):R186. doi: 10.1186/gb-2007-8-9-r186 (PMC2375024; doi:10.1186/gb-2007-8-9-r186)
Supplement: Additional data file 3 — Presented is the Bioconductor package ppiStats in 'Windows binary' format. [file gb-2007-8-9-r186-S3.zip › ppiStats/html/idProteinType.html]

R: A function to determine viable baits, viable preys, or
homodimers within experimental data-sets.

|  |  |
| --- | --- |
| idProteinType {ppiStats} | R Documentation |

## A function to determine viable baits, viable preys, or homodimers within experimental data-sets.

### Description

These functions take a bait to prey directed graphNEL and returns
either a character vector of all proteins which participates in
homodimer relationships or a list of two character vectors: a vector
of viable baits as well as a vector of viable prey.

### Usage

```
idViableProteins(bpGraph)
idHomodimers(bpGraph)
```

### Arguments

|  |  |
| --- | --- |
| `bpGraph` | A direced graphNEL |

### Value

The return value for idHomodimers is a character vector of those
proteins which participates in homodimer relationships.
  
The return value for idViableProteins is a list of two character
vectors:

|  |  |
| --- | --- |
| `viableBaits` | A vector of baits that finds at least one prey in the experimental graphNEL, i.e. the nodes with out-degree at greater than 0 |
| `viablePrey` | A vector of prey which is found by at least one bait in the experimental graphNEL, i.e. the nodes with in-degree at greater than 0 |

### Author(s)

T Chiang

### Examples

```
library(ppiData)
idViableProteins(Ito2001BPGraph)
idHomodimers(Ito2001BPGraph)
```

---

[Package *ppiStats* version 1.3.5 Index]
