# Supplementary material for: Coverage and error models of protein-protein interaction data by directed graph analysis
Source: Genome Biol. 2007 Sep 10;8(9):R186. doi: 10.1186/gb-2007-8-9-r186 (PMC2375024; doi:10.1186/gb-2007-8-9-r186)
Supplement: Additional data file 3 — Presented is the Bioconductor package ppiStats in 'Windows binary' format. [file gb-2007-8-9-r186-S3.zip › ppiStats/html/nullDistDoublyTestedEdges.html]

R: Null distribution of number of reciprocated,
unreciprocated and missing edges in stochastic model.

|  |  |
| --- | --- |
| nullDistDoublyTestedEdges {ppiStats} | R Documentation |

## Null distribution of number of reciprocated, unreciprocated and missing edges in stochastic model.

### Description

Calculate the null distribution of the number of reciprocated,
unreciprocated and missing edges in a stochastic model where each
edge is tested twice.

### Usage

```
nullDistDoublyTestedEdges(deltaMax, n, pFP, pFN)
```

### Arguments

|  |  |
| --- | --- |
| `deltaMax` | Integer. Distributions will be calculated for model parameter *delta*`=0, 1, 2, ..., deltaMax`. |
| `n` | Integer. The parameter *n* of the model. |
| `pFP` | Numeric. The parameter *pFP* of the model. |
| `pFN` | Numeric. The parameter *pFN* of the model. |

### Details

The model is described in the vignette
*Stochastic and systematic errors in PPI data, by looking
at unreciprocated in- or out-edges*
by W. Huber, T. Chiang and R. Gentleman.

This function can be quite slow, its runtime grows quickly
with `deltaMax` (and is roughly independent of `n`, `pFP`,
`pFN`). The example below should take only a few seconds on a reasonable
computer, though.

### Value

3d array with dimensions `nMax+1` x `nMax+1` x
`deltaMax+1` whose element `p[nr+1, nu+1, delta+1]`
is the corresponding joint probability. `nMax+1` is calculated
(probably too conservatively) by the function to make sure that no
probability leaks out of the array.

### Author(s)

Wolfgang Huber http://www.ebi.ac.uk/huber

### Examples

```
p = nullDistDoublyTestedEdges(32, 1000, pFP=0.001, pFN=0.15)

if(interactive() && require("RColorBrewer"))
  for(k in 1:dim(p)[3]) {
    image(sqrt(p[,,k]), xlab=expression(N[rec]), ylab=expression(N[unrec]),
        main = expression(P(N[rec], N[unrec]~";"~ delta^"*", n, p[FP], p[FN])),
        x = 1:dim(p)[1], y = 1:dim(p)[2], 
        col = colorRampPalette(brewer.pal(9, "GnBu"))(256))
    text(35, 35, paste("delta", k, sep="="))
  }
```

---

[Package *ppiStats* version 1.3.5 Index]
