# Supplementary material for: Coverage and error models of protein-protein interaction data by directed graph analysis
Source: Genome Biol. 2007 Sep 10;8(9):R186. doi: 10.1186/gb-2007-8-9-r186 (PMC2375024; doi:10.1186/gb-2007-8-9-r186)
Supplement: Additional data file 3 — Presented is the Bioconductor package ppiStats in 'Windows binary' format. [file gb-2007-8-9-r186-S3.zip › ppiStats/html/twPERM.html]

R: A function to compute a permutation test on a two way table.h

|  |  |
| --- | --- |
| twPERM {ppiStats} | R Documentation |

## A function to compute a permutation test on a two way table.h

### Description

This function takes the entries of a two-way table and creates
two binary logical vectors upon which it computes a permutation
test to check for independence.

### Usage

```
twowayPERM( v1, v2, NPERM, stat, seed = 123)
makeBinVect(n11, n12, n21, n22)
```

### Arguments

|  |  |
| --- | --- |
| `v1` | A logical vector. |
| `v2` | A logical vector the same length as v1. |
| `NPERM` | A positive integer. The number of tests to be conducted. |
| `stat` | A statistical function passed into to test the permutation on two vectors. |
| `seed` | A positive integer. To set the seed for the random number generator. |
| `n11` | A postive integer. The value of (1,1) in the two way table. |
| `n12` | A postive integer. The value of (1,2) in the two way table. |
| `n21` | A postive integer. The value of (2,1) in the two way table. |
| `n22` | A postive integer. The value of (2,2) in the two way table. |

### Value

A numeric vector containing the p-values for the test that
the permutation of the vector v1 is independent from v2.

### Author(s)

T Chiang

### Examples

```
x <- makeBinVect(13, 17, 23, 71)
```

---

[Package *ppiStats* version 1.3.5 Index]
